# New Dromaeosaurid Dinosaur (Theropoda, Dromaeosauridae) from New Mexico and Biodiversity of Dromaeosaurids at the end of the Cretaceous

**DOI:** 10.1038/s41598-020-61480-7

**Published:** 2020-03-26

**Authors:** Steven E. Jasinski, Robert M. Sullivan, Peter Dodson

**Affiliations:** 1 University of Pennsylvania, Department of Earth and Environmental Science, Philadelphia, PA 19104-6316 USA; 2 State Museum of Pennsylvania, Section of Paleontology and Geology, 300 North Street, Harrisburg, PA 17120-0024 USA; 3 Don Sundquist Center of Excellence in Paleontology, Johnson City, TN 37614-1709 USA; 4 New Mexico Museum of Natural History and Science, 1801 Mountain Road N.W., Albuquerque, NM 87104 USA; 5 School of Veterinary Science, University of Pennsylvania, Philadelphia, PA 19104-6316 USA

**Keywords:** Palaeontology, Taxonomy, Phylogenetics

## Abstract

Dromaeosaurids (Theropoda: Dromaeosauridae), a group of dynamic, swift predators, have a sparse fossil record, particularly at the time of their extinction near the Cretaceous-Paleogene boundary. Here we report on a new dromaeosaurid, *Dineobellator notohesperus*, gen. and sp. nov., consisting of a partial skeleton from the Upper Cretaceous (Maastrichtian) of New Mexico, the first diagnostic dromaeosaurid to be recovered from the latest Cretaceous of the southern United States (southern Laramidia). The holotype includes elements of the skull, axial, and appendicular skeleton. The specimen reveals a host of morphologies that shed light on new behavioral attributes for these feathered dinosaurs. Unique features on its forelimbs suggest greater strength capabilities in flexion than the normal dromaeosaurid condition, in conjunction with a relatively tighter grip strength in the manual claws. Aspects of the caudal vertebrae suggest greater movement near the tail base, aiding in agility and predation. Phylogenetic analysis places *Dineobellator* within Velociraptorinae. Its phylogenetic position, along with that of other Maastrichtian taxa (*Acheroraptor* and *Dakotaraptor*), suggests dromaeosaurids were still diversifying at the end of the Cretaceous. Furthermore, its recovery as a second North American Maastrichtian velociraptorine suggests vicariance of North American velociraptorines after a dispersal event during the Campanian-Maastrichtian from Asia. Features of *Dineobellator* also imply that dromaeosaurids were active predators that occupied discrete ecological niches while living in the shadow of *Tyrannosaurus rex*, until the end of the dinosaurs’ reign.

## Introduction

Dromaeosaurids (Theropoda: Dromaeosauridae) have been found in North America from the Early to Late Cretaceous, from as far west as Alaska to as far east as Maryland^1–3^. However, their fossil record is very poor near the time of their extinction prior to the Cretaceous-Paleogene boundary in North America. Additional taxa have been named from the Early Cretaceous, including *Yurgovuchia doellingi*^4^, *Utahraptor ostrommayorum*^5^ and *Deinonychus antirrhopus*^6,7^. Several taxa are known from the Late Cretaceous, but almost all are from the Campanian^1,3,8–12^. Recently, two taxa (*Acheroraptor temertyorum* and *Dakotaraptor steini*) were named from the upper Maastrichtian Hell Creek Formation, but, aside from these two skeletal fossil specimens, non-tooth material of Maastrichtian taxa is rare^3,13,14^. Although isolated dromaeosaurid teeth are somewhat common in Campanian age strata of North America, these teeth reveal little ecological information about this group.

Here we report on a new dromaeosaurid dinosaur, *Dineobellator notohesperus*, gen. and sp. nov., discovered in 2008, and briefly mentioned by Jasinski *et al*.^15^, from the Naashoibito Member (Ojo Alamo Formation), San Juan Basin, New Mexico. The holotype specimen, SMP VP-2430 (Vertebrate Paleontology Collection, State Museum of Pennsylvania, Harrisburg, Pennsylvania, USA), consists of at least 20 identifiable skeletal elements, including parts of the skull, fore- and hindlimbs, and axial skeleton. These skeletal remains are complete enough to compare to other known dromaeosaurids, assess its phylogenetic position, and infer additional aspects of their life history and predatory behavior. This specimen constitutes the first significant skeletal remains of a Maastrichtian dromaeosaurid from south of North American 43^rd^ latitude (South Dakota) in North America.

### Systematic paleontology

Dinosauria Owen, 1842; Theropoda Marsh, 1881; Coelurosauria Huene, 1914; Dromaeosauridae Matthew and Brown, 1922; *Dineobellator notohesperus* gen. et sp. nov.

### Etymology

The generic name is derived from *Diné*, the Navajo word in reference to the people of the Navajo Nation, and the Latin suffix *bellator*, meaning warrior. The specific epithet *noto* is from the Greek, meaning southern, or south; and the Greek *hesper* meaning western, in reference to the American Southwest. Additionally, Hesperus refers to a Greek god, namely the personification of the evening star and, by extension, “western.” Pronounced “dih NAY oh - BELL a tor” “Noh toh – hes per us.”

### Holotype

**Figure 1:**
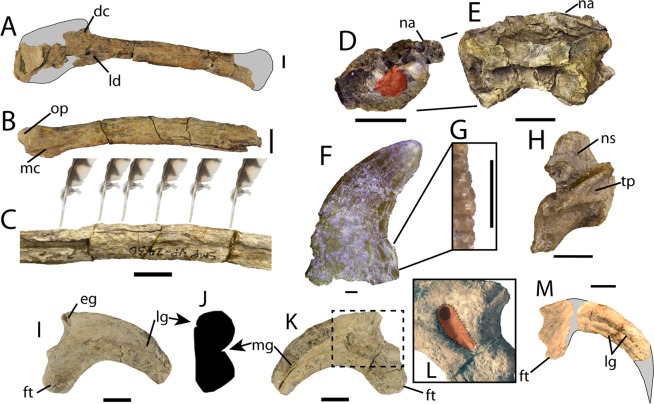
Selected elements and features of the holotype of *Dineobellator notohesperus* (SMP VP-2430), gen. et sp. nov., including: right humerus, posterior (**A**) view; right ulna, medial (**B**) view; close up of ulna showing feathers where ulnar papillae are located along the ulnar ridge, feathers used are from *Megascops kennicottii* (**C**); middle caudal vertebra (**D**,**E**), distal (**D**) and (**E**) lateroventral (**E**) views, with red highlighting circular indent on centrum surface; tooth, lateral (**F**) view; magnification of distal basal denticles (**G**); anterior caudal vertebra 1, right lateral (**H**) view; right manual ungual II (**I**–**L**), lateral (**I**) view, silhouette of transverse plane of right manual ungual II near distal end (**J**), medial (**K**) view, and with area shown in dashed box in K highlighting abnormal oblong concavity in red (**L**); right pedal ungual III, partially reconstructed, lateral (**M**) view. Abbreviations: cc, central concavity; dc, deltopectoral crest; eg, digital extensor groove; ft, flexor tubercle; ld, latissimus dorsi scar; lg, lateral groove; mc, medial crest; mg, medial groove; na, neural arch; ns, neural spine; op, olecranon process; tp, transverse process. Scale bars, 1 cm for (**A**–**E**) and (**H**–**M**), 1 mm for (**F**,**G**). (**L**) not to scale.

**Figure 2:**
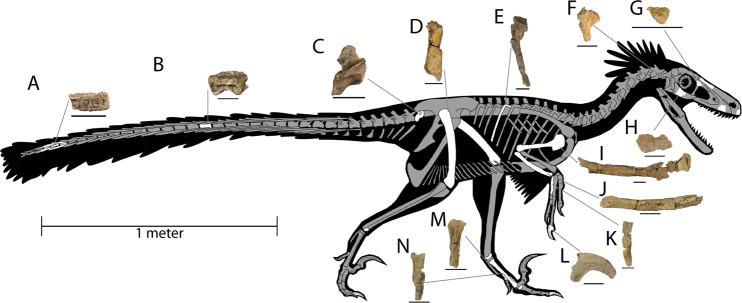
Skeletal reconstruction of *Dineobellator notohesperus* gen. et sp. nov., SMP VP-2430, with known elements colored in white. Figured bones are as follows: fused distal caudal vertebra (**A**); middle caudal vertebra (**B**); caudal vertebra 1 (**C**); right femur (**D**); rib (**E**); right basipterygoid (**F**); left lacrimal (reversed) (**G**); right jugal (**H**); right humerus (**I**); right ulna (**J**); right metacarpal III (**K**); right manual ungual II (**L**); right metatarsal II (**M**); right metatarsal III. (**N**) Individual scale bars, 2 cm. Skeletal drawing based off work of Scott Hartman.

SMP VP-2430 is a disarticulated, associated individual consisting of a rostromedial portion of right premaxilla, left maxilla fragment, ?maxillary tooth, dorsolateral process of left lacrimal, left ?nasal fragment, incomplete right jugal, incomplete right basipterygoid, incomplete occipital condyle, isolated prezygopophyses, isolated vertebral processes, caudal vertebra 1, middle caudal vertebra, four fused distal caudal vertebrae, several vertebral fragments, nearly complete rib and rib fragments, nearly complete right humerus, nearly complete right ulna, incomplete right metacarpal III, nearly complete right manual ungual II, incomplete right femur, incomplete right metatarsals I, II and III, incomplete left ?astragalus, nearly complete right pedal ungual III, and various other cranial and post-cranial bone fragments (Figs. 1–2). Portions of the specimen were first found and collected by Robert M. Sullivan, Steven E. Jasinski, and James Nikas in 2008, and more material was subsequently collected from the same individual by Sullivan and Jasinski in 2009 and Jasinski in 2015 and 2016.

### Type locality and horizon

The type locality, SMP 410b, Bisti/De-na-zin Wilderness, New Mexico. Precise locality information is on file at the State Museum of Pennsylvania, Section of Paleontology and Geology, and is available to qualified researchers. The holotype (SMP VP-2430) was collected within a few meters above the base of the Naashoibito Member (Ojo Alamo Formation) in relatively poorly consolidated sandstone. ^40^Ar/^39^Ar dates acquired from detrital sanidines give a maximum depositional age for the Naashoibito Member at 66.5 ± 0.2 Ma (upper Maastrichtian)^16–20^. Biostratigraphy, however, seems to suggest an early late Maastrichtian age, approximately 70.0–68.0 Ma^21^.

### Diagnosis

A mid-sized dromaeosaurid theropod that differs from other eudromaeosaurs by the following characters: offset of lateral grooves on manual ungual; distinct and conspicuous dorsomedial groove proximally dorsal to the articular surface on the manual ungual; sharp angle of distal deltopectoral crest of the humerus; opisthocoelous proximal caudal vertebrae; short and robust neural spines on proximal caudal vertebrae; gracile and subrectangular transverse processes on proximal caudal vertebrae; proximal caudal vertebrae with curved ventral surface and oval to subrectangular cranial and caudal centrum surfaces; distinct round concavities on cranial and caudal centrum surfaces in mid-caudal vertebrae; enlarged flexor tubercles on manual ungual II and pedal ungual III; and secondary lateral grooves ventral on pedal unguals.

### Description

The type specimen of *Dineobellator notohesperus* (SMP VP-2430) is an animal similar in size to *Velociraptor* and *Saurornitholestes* based on the similar sizes of comparable elements. A few small fragments of SMP VP-2430 are from the skull of *Dineobellator notohesperus*. A rostromedial portion of the right premaxilla is preserved without teeth, but with portions of two alveoli. The alveoli are relatively inconspicuous (see SI Tables 1–2 for all measurements) and closely spaced. A subrectangular fragment of the left maxilla is preserved with two partial alveoli. The dorsolateral process of the left lacrimal is subtriangular with a rounded point laterally, a conspicuous lacrimal fenestra, and is similar to those in other dromaeosaurids ^see refs.^ ^22,23^ (Fig. 2G). Another subrectangular fragment, this one of the left nasal, has an enlarged medial sutural surface and a flat dorsal surface. A flat, trapezoidal portion of the right jugal is slightly curved laterally toward its rostral and caudal ends, suggesting a relatively deep jugal (Fig. 2H). The braincase is incomplete with only the condylar portion of the basioccipital preserved. The caudal portion of the braincase is subcircular and obliquely twisted. The right basipterygoid process of the basisphenoid is prominent medially and externally, directed caudodorsally, and possesses a thin canal internally, inferred to represent a neurovascular groove, as for the palatine ramus of the facial nerve + palatine artery (Fig. 2F). The right basal tuber is robust but incomplete medially. Its rostral edge is directed rostrolaterally with a deep U-shaped notch between the processes. Portions of the carotid canal are present on the medial edge of the basipterygoid recess and run rostrocaudally. Ventrally, the ovoid opening for the carotid canal is 6.5 mm long.

#### Teeth

A gracile, ziphodont,?maxillary tooth measures 12.0 mm in apical length (referring to the total height of the tooth including the preserved root), with a crown height of 11.3 mm (Fig. 1F). There are approximately 18–20 denticles per 5 mm (3.7 to 4.3 denticles per mm) on the distal carina (distal basal denticles) (Fig. 1G), and no denticles on the mesial carina. The angle between the lines of 10% and 90% of the length of the exposed denticles ^see ref.^ ^24^ normally falls between 86°–95° for well-preserved denticles, with most falling just under 90°. The denticles are nearly rounded with no indication of a hook and are short. A wear facet on the distal end of the mesial edge of the tooth measures 6.7 mm along the curvature. The tooth curves (concave) caudally and would be perpendicular to the alveolar margin (not strongly raked [including caudally] in the alveolus).

#### Axial

Several vertebrae and vertebral fragments are preserved in SMP VP-2430, including a nearly complete proximal caudal vertebra, the first postsacral vertebra (Figs. 1H, 2C). Its neural arch and spine are robust but short. The cranial and caudal faces of the centrum are subrectangular, and wider than tall. While the cranial surface is flat, the caudal is concave, making it opisthocoelous. The transverse processes project laterally, and are subrectangular, short, and gracile. The ventral surface is distinctly downcurved toward the caudal end. Another nearly complete vertebra represents an amphicoelous caudal vertebra approximately midway through the caudal series (~#8–~#12) (Figs. 1D,E, 2B). The cranial and caudal centrum surfaces are subrectangular to subtrapezoidal with well-defined and conspicuous circular indentations on both the cranial and caudal ends. These concavities of the centrum are symmetrical and both lie near the center of the centrum on their respective surfaces. A limited series of fused caudal vertebrae is preserved in SMP VP-2430 representing portions of at least four caudal vertebrae (Fig. 2A). These are not distal caudal vertebrae and, therefore, not representative of a pygostyle. Two of the vertebrae are complete, having lengths of 4.1 mm and 5.1 mm, respectively. Several fragmentary bones are identified as parts of ribs. One represents a nearly complete left dorsal rib that exhibits some taphonomic distortion distally and a long, thin depression laterally (Fig. 2E). The bone has an irregular surface in places, mostly proximally, with areas of slight expansion or depression along the rib shaft. This irregular morphology suggests bone remodeling and therefore is likely pathologic.

#### Forelimb

The nearly complete right humerus measures 186 mm, with an estimated total length of 215 mm (Figs. 1A, 2I). The proximal portion is thin and gracile with the proximal edge mostly flat with a slight bend medially versus sigmoidal in other dromaeosaurid humeri. The deltopectoral crest is thinner and more gracile than the shaft, projects cranially, lies approximately perpendicular to the long axis of the humeral head, and is approximately 31% the total length of the humerus. The distal edge of the crest forms a sharp, acute angle with the shaft of the humerus. Distally, the shaft is sub-round to oval and hollow in cross-section at the broken distal end. The incomplete right ulna is a long, thin, bowed bone with the preserved portion having a length of 101 mm, yielding a total estimated length of 140 mm (Figs. 1B, 2J). It has a shallow trochlear notch proximally and an inconspicuous, transversely broad, subtriangular olecranon process. At least six protuberances, identified as ulnar papillae or quill knobs, lie along the ventral ulnar ridge (Fig. 1C), which suggests a total estimate of 12–14 secondary remiges. The right incomplete metacarpal III is slightly ventrally-curved and tapers distally (Fig. 2K). On the proximal surface, the dorsal portion of the bone is wider than the ventral portion, giving it a generalized backwards-“P” shape.

#### Hindlimb

The incomplete right femur is robust, with a preserved length of 69 mm, and a rough estimate of a total length of 275 mm (Fig. 2D). The preserved portion of the femoral head suggests the shaft is twisted as in other dromaeosaurid femora. Right metatarsal I is slightly twisted about its shaft and is missing part of its proximal end. There is a relatively large foramen at its distal end with a pronounced, rounded rim. Right metatarsal II, represented by the proximal (Fig. 2M) and distal portions, is thin and gracile. Proximally, the bone is subtriangular, with a distinct groove from the proximal edge that runs halfway down the preserved proximal portion. The distal portion is also subtriangular and flares out to the condyles. The right metatarsal III is thinner proximally and extends toward the distally preserved surface (Fig. 2N).

#### Ungual

A nearly complete right manual ungual II, missing only the tip, measures 45.6 mm long from the ventral edge of the articular surface to the preserved distal end. The complete ungual would have a total length of approximately 50 mm (Figs. 1I–L, 2L). It has a pronounced flexor tubercle along its proximoventral edge, and a significant arched profile, with the dorsal surface approximately 114 ° in relation to the articulation surface. A lateral groove (or depression) runs along its length toward the distal tip. This groove lies between the articular surface and flexor tubercle and extends the length of the claw toward the dorsal surface distally. A similar groove is present medially. The two grooves are offset as the medial groove does not converge with the dorsal surface, unlike the lateral groove. On the medial surface near the proximal end of the groove lies a prominent gouge mark (Fig. 1L). This mark, or furrow, extends proximoventrally and terminates in a prominent, but small, depression close to the dorsal edge. The gouge has an approximate width of 3 mm and extends for a length of 9 mm. This feature does not exhibit any abnormal morphology, suggesting it is not due to an infection or pathology associated with the keratinous sheath. Its presence on only one side of the element suggests it is not diagenetic. The flexor tubercle is large (93% of the size of the articular surface) and perpendicular to the articular surface. Dorsally adjacent to the articular surface is a ridge extending to the middle of the ungual, with grooves on both sides. On its proximodorsal surface is a faint, slight lip. Directly ventral to the articular surface is another slight lip, or ridge, that comes to two lateral points or projections. Proximal and distal portions of right pedal ungual III are preserved, with only a small, middle portion missing (Fig. 1M). The proximal fragment is 13.2 mm long (proximodistally) and the distal fragment is 32.0 mm long, with a total estimated length of 55 mm. The flexor tubercle is significantly smaller and less pronounced than that of the manual ungual II, but relatively large compared to other pedal unguals, with the tubercle 67% the length of the articular surface. There is a slight concave curvature below the articular surface, with the flexor tubercle perpendicular to the articular surface. Grooves are present on both the lateral and medial surfaces of the claw and are offset from each other, like those of the manual ungual. The lateral groove encroaches toward the dorsal surface as it extends distally, while the medial one does not, as in manual ungual II. There is a second, less conspicuous depression, or groove, ventral to the main one on both the lateral and medial surfaces. The pedal claw is thinner in profile than the manus claw and lacks a pronounced curvature.

### Remarks

The distinct offset nature of the longitudinal grooves of the manual ungual are often found on the pedal unguals of several dromaeosaurid taxa and are barely offset in the manual ungual in one other taxon (*Boreonykus certekorum*), but not in other dromaeosaurids. The distinct dorsomedial groove proximally near the articulation surface is not seen in other dromaeosaurid taxa. The flattened proximal edge of the humerus of *Dineobellator* is distinct from the sigmoidal shape in other dromaeosaurids (e.g., *Saurornitholestes*, *Bambiraptor*, *Deinonychus*). The sharp, acutely angled curvature of the distal portion of the deltopectoral crest is unique among dromaeosaurids, although it is less smooth in *Deinonychus* (AMNH 3015, American Museum of Natural History, New York, New York, USA). The deltopectoral crest is relatively larger in *Dineobellator* [estimated 31% of total humeral length in *Dineobellator* compared to other dromaeosaurids with preserved humeri (e.g., 20.5% in *Bambiraptor feinborgorum*, 23.5% in *Dakotaraptor steini*, 25% in *Saurornitholestes langstoni*, and 28% in *Deinonychus antirrhopus*)]. In other dromaeosaurids, proximal caudal vertebrae are acoelous or amphiplatyan. The opisthocoelous proximal caudal vertebrae of *Dineobellator* are unknown in other dromaeosaurids, although they have been found in the caenagnathid theropod *Gigantoraptor erlianensis*^25^. The ventral surface of the proximal caudal is curved ventrally, while those of other dromaeosaurids (e.g., *Deinonychus*) are angled, but not curved, in lateral view. The transverse processes of the proximal caudal vertebra I is subrectangular, distinct from *Deinonychus* where they are subtriangular and *Velociraptor* where they are enlarged and fan out distally. The centrum surfaces, particularly on the posterior (=caudal) end, are distinctly oval to subrectangular in *Dineobellator* rather than rounded as in other dromaeosaurids. The subcircular concavities on the cranial and caudal surfaces of the centra of the mid-caudal vertebrae are symmetrical and not seen in other dromaeosaurid caudal vertebrae. While the flexor tubercle is smaller in the pedal ungual than in the manual ungual, it is still enlarged compared to those of other dromaeosaurid taxa (e.g., *Bambiraptor*, *Deinonychus*, *Utahraptor*), and most similar in relative size to *Dakotaraptor* pedal unguals. Additionally, the smaller secondary grooves ventral to the main lateral grooves on the pedal ungual are unique among dromaeosaurids. While the late Campanian *Saurornitholestes sullivani* (holotype frontal SMP VP-1270) is from the older Kirtland Formation (De-na-zin Member) of the San Juan Basin^11,26–28^_,_ it lacks corresponding elements that would permit comparison. However, isolated dromaeosaurid teeth from the De-na-zin Member have been collected (SMP VP-1901), and these differ from those of *Dineobellator*. Teeth of *S*. *sullivani* are gently curved, have slightly apically hooked denticles, less dense denticles (14–15 denticles per 5 mm compared to 18–20 in *Dineobellator*), and possess mesial denticles. It is also noted that one of the diagnostic features of *S*. *langstoni* are distal denticles that are strongly hooked apically^22^. Additionally, the maxillary and dentary teeth of *S*. *langstoni* are vertical and perpendicular to the alveolar margin and possess mesial carinae (although these tend to be mainly or completely proximal on the teeth) that are distinctly smaller than distal carinae.

### Phylogenetic analysis

**Figure 3:**
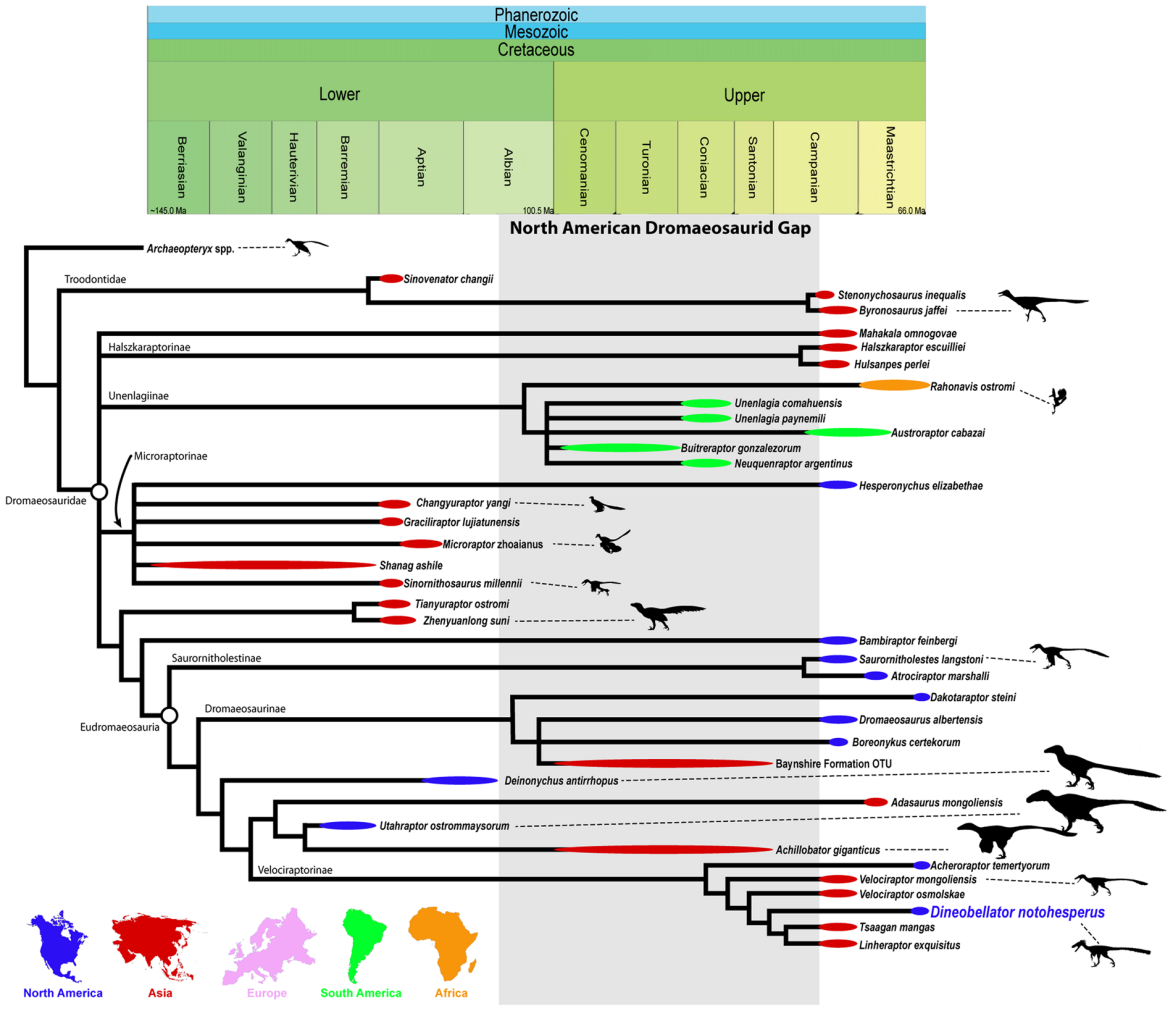
Time-calibrated phylogeny of dromaeosaurid relationships illustrating the major relationships within the family including their paleobiogeography. Strict consensus phylogenetic tree resulting in 32 most parsimonious trees, each with a tree length of 416 steps, a Consistency Index of 0.466, and a Retention Index of 0.640. *Archaeopteryx* is the outgroup. Temporal positions and biogeographic locations of dromaeosaurid taxa are provided. Silhouettes are taken from phylopic.org and are freely available for reuse under a Public Domain or Creative Commons license (www.phylopic.org), see SI for more information about individual silhouettes. Credits for silhouettes and references for temporal positions and biogeographic locations provided in SI.

The first of the two phylogenetic analyses resulted in a strict consensus tree that recovered Eudromaeosauria with several previously identified clades within, including Saurornitholestinae, Dromaeosaurinae, and Velociraptorinae (Fig. 3). Derived within Eudromaeosauria is Velociraptorinae with a mix of Campanian taxa from Asia and Maastrichtian taxa from North America, including *Dineobellator notohesperus*. Sister to Velociraptorinae lies a clade with the large-bodied taxa *Achillobator* and *Utahraptor* (and *Adasaurus*), with *Deinonychus antirrhopus* as the sister taxon to these groups. Moving further down the tree is Dromaeosaurinae, recovered as a mainly North American clade, although it includes the Baynshire dromaeosaurid as well. Saurornitholestinae was recovered as the basal-most subfamily, with *Bambiraptor feinbergi* as the sister taxon to Eudromaeosauria. *Tianyuraptor* + *Zhenyuanlong* are found to be sister to *Bambiraptor* + Eudromaeosauria. In addition to the Eudromaeosauria + *Bambiraptor* + (*Tianyuraptor* + *Zhenyuanlong*) clade, Dromaeosauridae includes an unresolved, polytomic Microraptorinae + a mostly unresolved Unenlagiinae + Halszkaraptorinae + *Mahakala*. Previously recovered clades include the mainly Asian Microraptorinae with *Hesperonychus* as the sister taxon to the polytomic Asian microraptorines and the Gondwanan Unenlagiinae with *Rahonavis* from Madagascar as sister to South American dromaeosaurids. Similar to Currie and Evans^26^, Haszkaraptorinae was recovered as *Halszkraptor* + *Hulsanpes*, with *Mahakala* recovered outside this clade, contra Cau *et al*.^29^. As found in several previous studies, *Acheroraptor* was recovered basally in the mostly Asian Velociraptorinae^13,26,30^. *Dineobellator* represents the second North American member of this clade, and a more derived member than *Acheroraptor* as *Dineobellator* is the sister taxon to *Tsaagan mangas* + *Linheraptor exquisitus*. *Dineobellator notohesperus* was also run through an established theropod dataset (Theropod Working Group dataset) to further gather insight into its phylogenetic placement. While many theropod groups had higher resolution, intrafamilial relationships of the Dromaeosauridae were poorly resolved, resulting in *Dineobellator notohesperus* forming part of a large polytomy with other dromaeosaurids. While this helps confirm the dromaeosaurid affinities of *Dineobellator*, it does not provide further information on its relationships within Dromaeosauridae. The strict consensus majority rule tree for this dataset can be found with the Supplemental Information.

## Discussion

*Dineobellator notohesperus* represents the most complete theropod skeleton recovered from the late Maastrichtian Naashoibito Member and one of the most complete dromaeosaurids from the Maastrichtian of North America. *Dineobellator* co-existed with numerous other theropods, including caenagnathids, ornithomimids, troodontids, and tyrannosaurids^15,31–33^. The presence of *Dineobellator* suggests that dromaeosaurid dinosaurs continued to diversify into the late Maastrichtian. Because these taxa do not form a monophyletic clade, multiple lineages of dromaeosaurids are inferred to have been present during Campanian and Maastrichtian time, including at least two in the northern and one in the southern reaches of Laramidia. These lineages followed distinct evolutionary paths, while presumably filling similar ecological niches in their respective ecosystems. Our phylogenetic analysis suggests potentially at least four lineages during the Campanian, and at least two to three remaining into the Maastrichtian in North America. The recovery of *Dineobellator* as a second Maastrichtian North American velociraptorine further suggests vicariance in this group after a dispersal from Asian ancestors.

For nearly a century, since the recognition of the early late Campanian *Dromaeosaurus albertensis*, only indeterminate teeth and fragmentary dromaeosaurid remains had been recovered from Maastrichtian age strata in North America^12^. However, recently, Evans *et al*.^13^ reported on the first diagnostic late Maastrichtian North American dromaeosaurid, *Acheroraptor temertyorum*, consisting of a nearly complete right maxilla and potentially associated, nearly complete left dentary from the Hell Creek Formation of Montana. Soon after, a second dromaeosaurid, *Dakotaraptor steini*, was named by DePalma *et al*.^14^ from the Hell Creek Formation of South Dakota based on material from a larger individual and represented by portions of the fore- and hindlimbs and axial skeleton. It is noted that *Dakotaraptor* is likely a chimera and portions of the described skeleton have already been shown to not represent a dromaeosaurid, namely with the “furcula” reidentified as part of a turtle plastron ^see ref.^ ^34^. *Dineobellator notohesperus* represents the first diagnostic dromaeosaurid known from the Maastrichtian of southern North America, and only the third late Maastrichtian dromaeosaurid known from North America.

### Inferred behavior

Some aspects of the paleobiology of *Dineobellator*, including its inferred behavior, can be hypothesized based on morphological evidence. The deltopectoral crest of the humerus is the attachment site for several muscles in the forelimb ^see ref.^ ^35^. The *m*. *brachialis*, which originates on the distal edge of the deltopectoral crest^35^, would aid in the flexion of the forearm in *Dineobellator*. Enlarging the distal portion of the crest would result in the enlargement of the origin of this muscle. The change in the angle of the distal portion of the deltopectoral crest may have also allowed for the origin of the *m*. *brachialis* to shift, creating a more parallel orientation for the muscle in relation to the long axis of the radius and ulna. This orientation could have resulted in lower muscular forces necessary for flexion of the forearm, and similar or larger muscle sizes based on the enlarged deltopectoral crest could have provided greater strength capabilities of this movement. The enlarged dorsomedial groove on the manual ungual suggests larger digital extensors (*m*. *extensor digitorum brevis*). This could be counteracted by tighter grip strength of the manus, as evidenced by the enlarged flexor tubercle on the manual ungual relative to other dromaeosaurids (flexor tubercle approximately 93% height of articular surface in *Dineobellator*), including those of *Microraptor* (56%), *Bambiraptor* (55%), *Deinonychus* (55%), *Boreonykus* (60%), and *Velociraptor mongoliensi*s (77%). This tighter grip strength is also seen in the hindfeet relative to other eudromaeosaurs (67% in *Dineobellator*, 50% in *Dakotaraptor*, 40% in *Utahraptor*, 36% in *Deinonychus*, 30% in *Dromaeosaurus*, 22% in *Boreonykus*, 20% in *Velociraptor mongoliensis*, and 17% in *Bambiraptor*).

The possession of opisthocoelous proximal caudal vertebrae may have allowed more mobility and range of movement near the base, while keeping the rest of the tail stiff could allow it to act as a rudder or counterweight ^e.g.,^^36–39^. This may have increased the agility of *Dineobellator* and thus may have implications for its predatory behavior, particularly with respect to the pursuit of prey.

A gouge and depression on the manual ungual are inferred to be the result of an external force from a single event. These features are only present on the medial side of the ungual suggesting it is not due to postmortem deformation. They do not appear to be the result of an infection or disease causing a pathology. The absence of remodeling or retexturing of the bone suggests external trauma caused these features, and that the inflicted trauma that resulted in these marks occurred close to, or at, the time of death. The size of this groove is consistent with the morphology of the ungual of an animal of similar size to SMP VP-2430 (Fig. 1L). We speculate an altercation with another *Dineobellator* or other predatory theropod resulted in these marks. Additionally, there is a deformed and remodeled rib, suggesting a break that healed, indicating that the animal survived for a while after suffering the injury ^e.g.,^^40–43^. More evidence is needed to confirm or refute these features as pathologic features.

### Feathers

Several dromaeosaurid taxa have been found to possess feathers, or feather-like structures, such as the Barremian–early Aptian *Changyuraptor*^44^, the Aptian *Sinornithosaurus*^45,46^, *Zhenyuanlong*^47^, and *Wulong*^48^, and the Albian *Microraptor*^49–51^. Some of these also possess feathers on their hindlimbs and most are confined to smaller body sizes and classified within Microraptorinae, although *Zhenyuanlong* is larger than the others and has recently been recovered as the sister taxon to Microraptorinae + Eudromaeosauria^29^. In addition to exceptional preservation leading to the discovery of feathers in theropods, some taxa have been found with structures similar to the quill knobs (or ulnar papillae) in extant birds. Among these taxa are the Campanian Asian velociraptorine *Velociraptor mongoliensis*^52^ and the Maastrichtian North American dromaeosaurine *Dakotaraptor*^14^. The discovery of ulnar papillae in *Dineobellator* adds a third member of Eudromaeosauria to this group (Fig. 1C). With approximately 12–14 secondary feathers, based on the number of quill knobs, *Dineobellator* is similar to that of *V*. *mongoliensis* having 14 secondaries^52^ and lies between the estimates for the Maastrichtian *Rahonavis* (10 secondaries^53^), the Tithonian *Archaeopteryx* (12 or more secondaries^54^) and the Albian *Microraptor* (18 secondaries^50^). The presence of quill knobs in *Dineobellator* provides further evidence for feathers throughout Dromaeosauridae, which have been documented in the three major clades, and from the Barremian through the Maastrichtian. It seems likely that feathers were present in the earliest dromaeosaurids, and potentially all members thereafter, based on the widespread occurrence of quill knobs and feathers in microraptorines. Their presence in non-volant dromaeosaurids of varying sizes further supports the notion that these feathers did not evolve exclusively for flight. While there have been suggestions of the winged forelimbs being used for stabilization during predatory attack^55^, this would have been less important for larger-bodied taxa such as *Dakotaraptor*. It has been shown that coloration and patterns highly discernible within taxa may not have the same effect on prey ^e.g.^^56^. This implies that feathers can act as bright markers, species-recognition markers, and/or sexual display features without being visual signals that call attention of predators or prey. Modern raptorial birds show that color patterns can still be intricate and serve to both camouflage the predator and be part of the sexual selection process ^e.g.^^57–59^, and similar feather styles may have been present in dromaeosaurids.

### Dromaeosauridae hiatus in North America

While North American dromaeosaurids are known from the Barremian by multiple taxa (*Yurgovuchia* and *Utahraptor*)^4,5^, following *Deinonychus* in the early Albian^6,7^ there is a significant hiatus in their fossil record (Fig. 3). This hiatus (or gap) lasts until the middle to late Campanian with the appearance of *Dromaeosaurus*^1–3,23^. This approximately 30-million-year hiatus may be due, in part, to bias (e.g., preservational, collecting, sampling) against small and rarer taxa, making it difficult to determine if their absence is real or an artefact of the fossil record^12,60^. Any dromaeosaurids from this hiatus would be of extreme importance in understanding their evolution.

### Phylogenetic relationships

While Gondwanan dromaeosaurids are recovered as a monophyletic group, Laurasian dromaeosaurids are recovered in several different clades and most clades have both Asian and North American members (Fig. 3). *Dineobellator* and *Acheroraptor* are members of the mostly Asian Velociraptorinae. Dromaeosaurinae has Asian and North American members. DePalma *et al*.^14^. found that large-bodied dromaeosaurid taxa formed a monophyletic clade. However, our analysis suggests large-bodied taxa (e.g., *Deinonychus*, *Utahraptor*, *Achillobator*, *Dakotaraptor*) are represented in several clades of eudromaeosaurs, although *Utahraptor* and *Achillobator* are sister taxa in a small clade. The basal position of *Dakotaraptor* in Dromaeosaurinae suggests small-bodied size may be a derived trait in this group. The two upper Maastrichtian Hell Creek Formation taxa (*Dakotaraptor* and *Acheroraptor*) are found within the two distinct subfamilies of eudromaeosaurs. However, it is noted that the likely chimeric status of *Dakotaraptor* likely leads, at least partially, to the more basal position among dromaeosaurids in the phylogenetic analysis. The presence of Campanian velociraptorines in Asia and Maastrichtian velociraptorines in North America also suggests migration between Asia and North America sometime before the Maastrichtian. The phylogenetic analysis further suggests there were multiple lineages of dromaeosaurids in North America during Campanian and Maastrichtian time, including two in the northern and at least one in the southern portions of Laramidia (present day southwestern North America). *Dineobellator*, recovered as a velociraptorine, also suggests vicariance in late Maastrichtian North American dromaeosaurids, with different taxa in the northern and southern United States. Close morphological relationships between Asian and San Juan Basin taxa has previously been noted for ankylosaurids^61^ and pachycephalosaurids^62–65^. This suggests movement of dinosaur species, with vicariance occurring between biogeographical regions. These latest dromaeosaurid lineages followed distinct evolutionary paths, while presumably filling similar ecological niches in their respective ecosystems. New discoveries of dromaeosaurids during the Late Cretaceous will provide further clarity as to whether distinct lineages lived throughout this time. Our analysis suggests multiple faunal interchanges and migrations between Asia and North America in the Late Cretaceous, with potential vicariance occurring into the very end of the Cretaceous in dromaeosaurids, particularly in North America.

## Methods

### Phylogenetic analysis

Phylogenetic analyses were run on two datasets used to explore relationships of the Dromaeosauridae and Theropoda. The first phylogenetic analysis used 38 operational taxonomic units (34 ingroup OTUs) and 180 characters. These data were based on the study of Currie and Evans^22^, which was, in turn, based on the studies of Bell and Currie^30^, Evans *et al*.^13^, and Longrich and Currie^10^. Data were run with TNT version 1.5^66^. This analysis resulted in 32 most parsimonious trees, each with a tree length of 416 steps, a Consistency Index of 0.466, and a Retention Index of 0.640 (Fig. 3). The Theropod Working Group dataset was also used, mainly from Brusatte *et al*.^67^ and recently updated by Cau *et al*.^29,68^. This analysis includes 157 OTUs and 860 characters. It was also run in TNT version 1.5^66^ and resulted in 11,590 most parsimonious trees, each with a tree length of 3317 steps, a Consistency Index of 0.328, and a Retention Index of 0.7612. Both datasets were subjected to a New Technology search (with default parameters for sectorial search, ratchet, tree drift, and tree fusion). While many theropod groups had higher resolution in the second phylogenetic analysis, intrafamilial relationships of Dromaeosauridae were poorly resolved. The strict consensus majority rule tree for this dataset can be found with the Supplemental Information.

### Nomenclatural acts

This published work and its nomenclatural acts have been registered in ZooBank which is a proposed online registration system for the International Code of Zoological Nomenclature (IZCN). The LSID (Life science identifiers) for this publication is urn:lsid:zoobank.org:pub:B32D7094-1A0E-400B-89A4-036640F3ED63. The associated information can also be viewed through any standard web browser by appending the LSID to the prefix “http://zoobank.org/”.

## Supplementary information

Supplementary information 1.

Supplementary information 2.
