# Supplementary material for: New Dromaeosaurid Dinosaur (Theropoda, Dromaeosauridae) from New Mexico and Biodiversity of Dromaeosaurids at the end of the Cretaceous
Source: Sci Rep. 2020 Mar 26;10:5105. doi: 10.1038/s41598-020-61480-7 (PMC7099077; doi:10.1038/s41598-020-61480-7)
Supplement: Supplementary file 1 — Supplementary information 1. [file 41598_2020_61480_MOESM1_ESM.pdf]

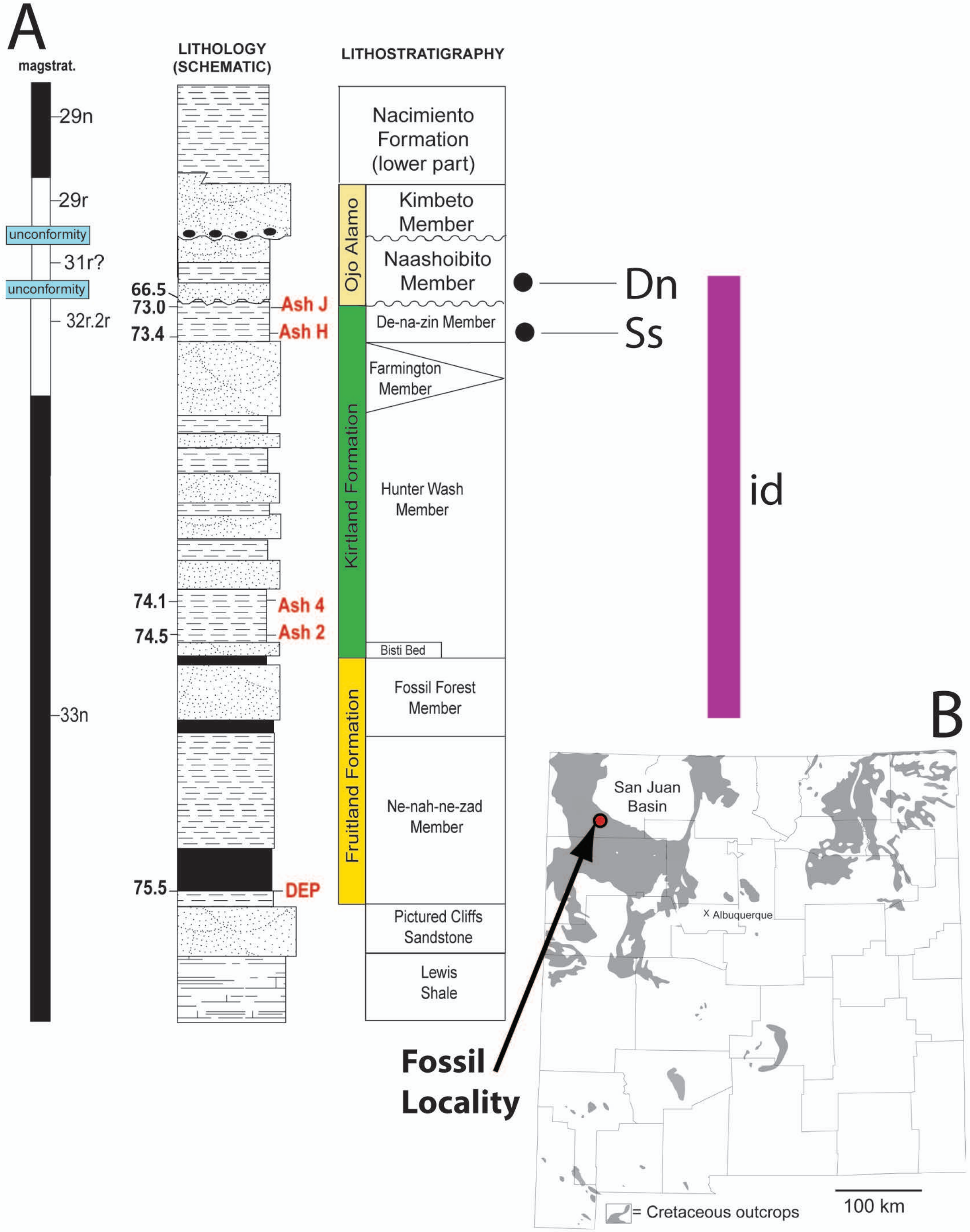

SUPP FIGURE 1. Stratigraphic and geographic position of the specimens and taxa described from New Mexico in the present study. A, stratigraphic schematic of the dromaeosaurids from the Late Cretaceous Fruitland, Kirtland, and Ojo Alamo formations, San Juan Basin, New Mexico. B, geographic location of the field localities in San Juan County, New Mexico. The numbers on the left side of the lithology schematic refer to millions of years ago. Ash dates show for the Fruitland and Kirtland formations are from Fassett and Steiner (1997), and the 66.5 Ma date (base of the Naashoibito Member) is from Mason et al. (2013). Abbreviations: Dn, *Dineobellator notohesperus*; id, indeterminate dromaeosaurids; Ss, *Saurornitholestes sullivani*. It is noted that indeterminate dromaeosaurids have also been found throughout the Fossil Forest, Hunter Wash, Farmington, De-na-zin, and Naashoibito members, although these may be referable to *D. notohesperis*, *S. sullivani*, or another taxon.

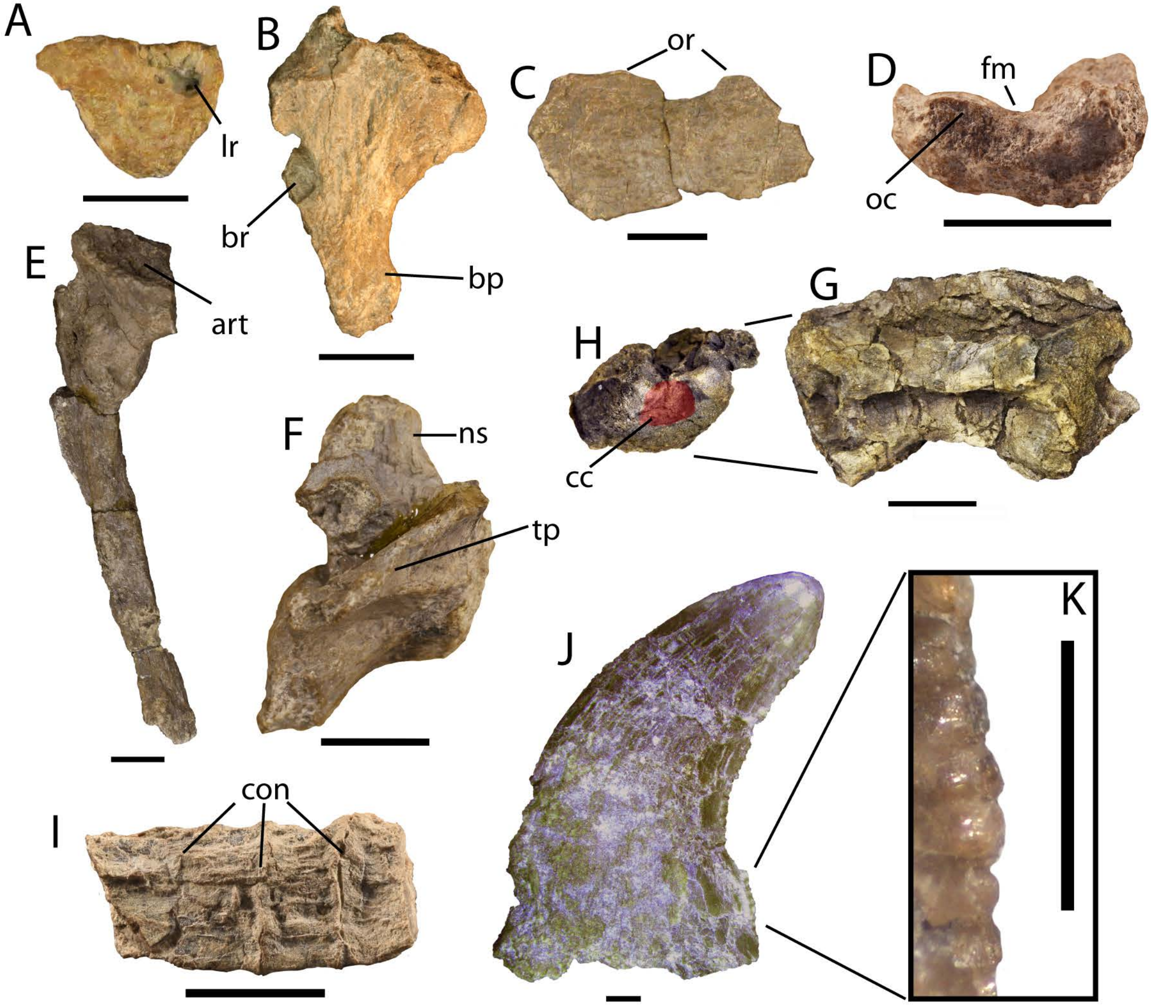

SUPP FIGURE 2. Cranial and axial elements of *Dineobellator notohesperus*, including: A, left lacrimal, ventrolateral view; B, right basipterygoid, caudoventral view; C, incomplete right jural, lateral view; D, incomplete basioccipital, caudal view; E, nearly complete rib, anterior view; F, caudal vertebra 1, right lateral view; G–H, middle caudal vertebra, (G) lateroventral view and (H) distal view; I, distal caudal vertebrae, lateral view; J, tooth, lateral view; K, magnification of distal basal denticles. Abbreviations are as follows: art, articulation surface; bp, basipterygoid process; br, basipterygoid recess; cc, central concavity; con, centrum contact surface; fm, foramen magnum; lr, lacrimal recess; ns, neural spine; oc, occipital condyle; or, orbital rim; tp, transverse process. Scale bars, 1 cm for A–I, 1 mm for J–K.

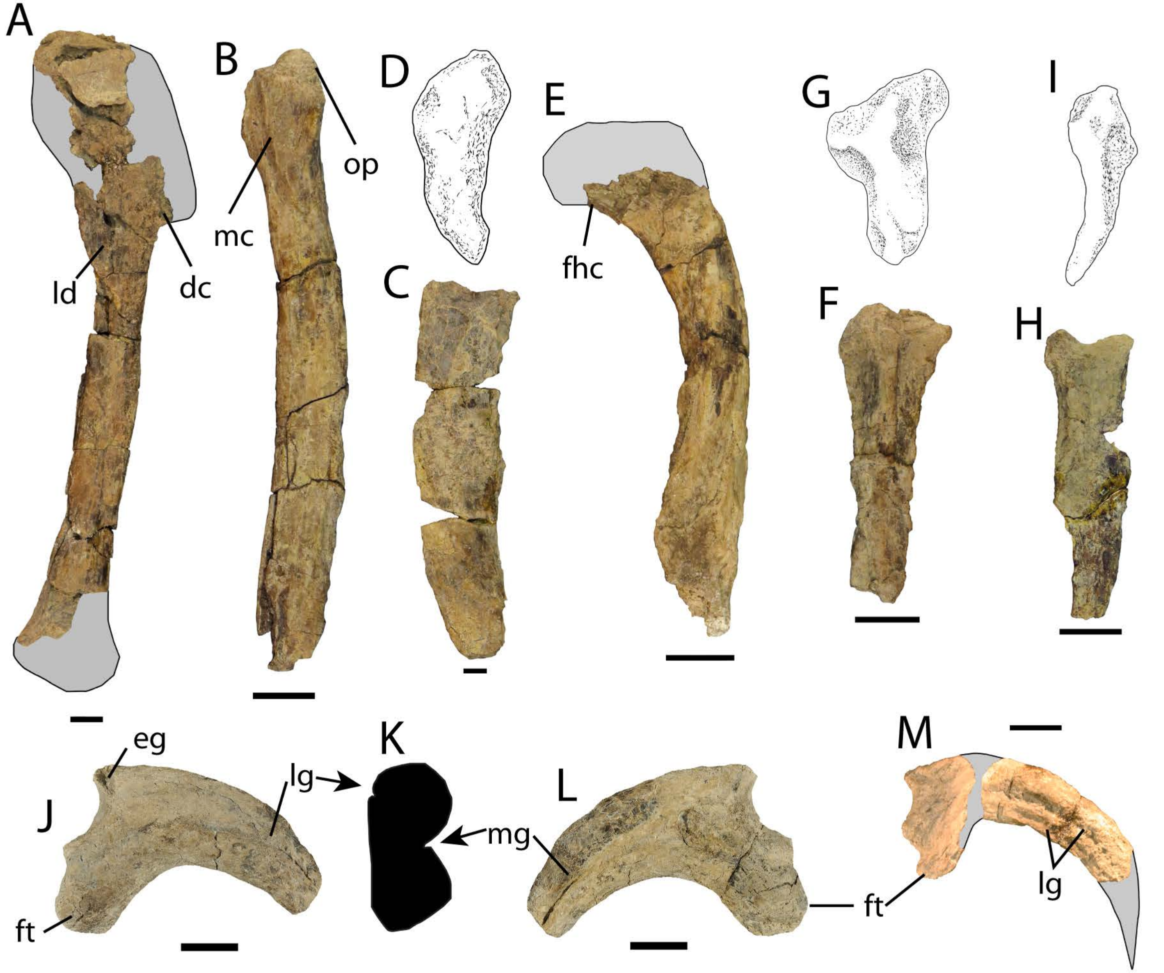

SUPP FIGURE 3. Appendicular elements of *Dineobellator notohesperus*, including: A, right humerus, posterior view; B, right ulna, medial view; C, incomplete right metacarpal III, medial view; D, illustration of proximal end of right metacarpal III, dorsal to top of page; E, incomplete right femur, posterior view; F, incomplete right metatarsal II, medial view; G, illustration of proximal end of right metatarsal II, dorsal to top of page; H, incomplete right metatarsal III, medial view; I, illustration of proximal end of right metatarsal III, dorsal to top of page; J, right manual ungual II, lateral view; K, silhouette of transverse plane of right manual ungual II near distal end; L, right manual ungual II, medial view; M, right pedal ungual III, partially reconstructed, lateral view. Abbreviations are as follows: dc, deltopectoral crest; eg, digital extensor groove; fhc, curvature for femoral head; ld, latissimus dorsi scar; lg, lateral groove; mc, medial crest; mg, medial groove; op, olecranon process. Scale bars, 1 cm for A–C, E–F, H, J, L–M. D, G, I, K not to scale.

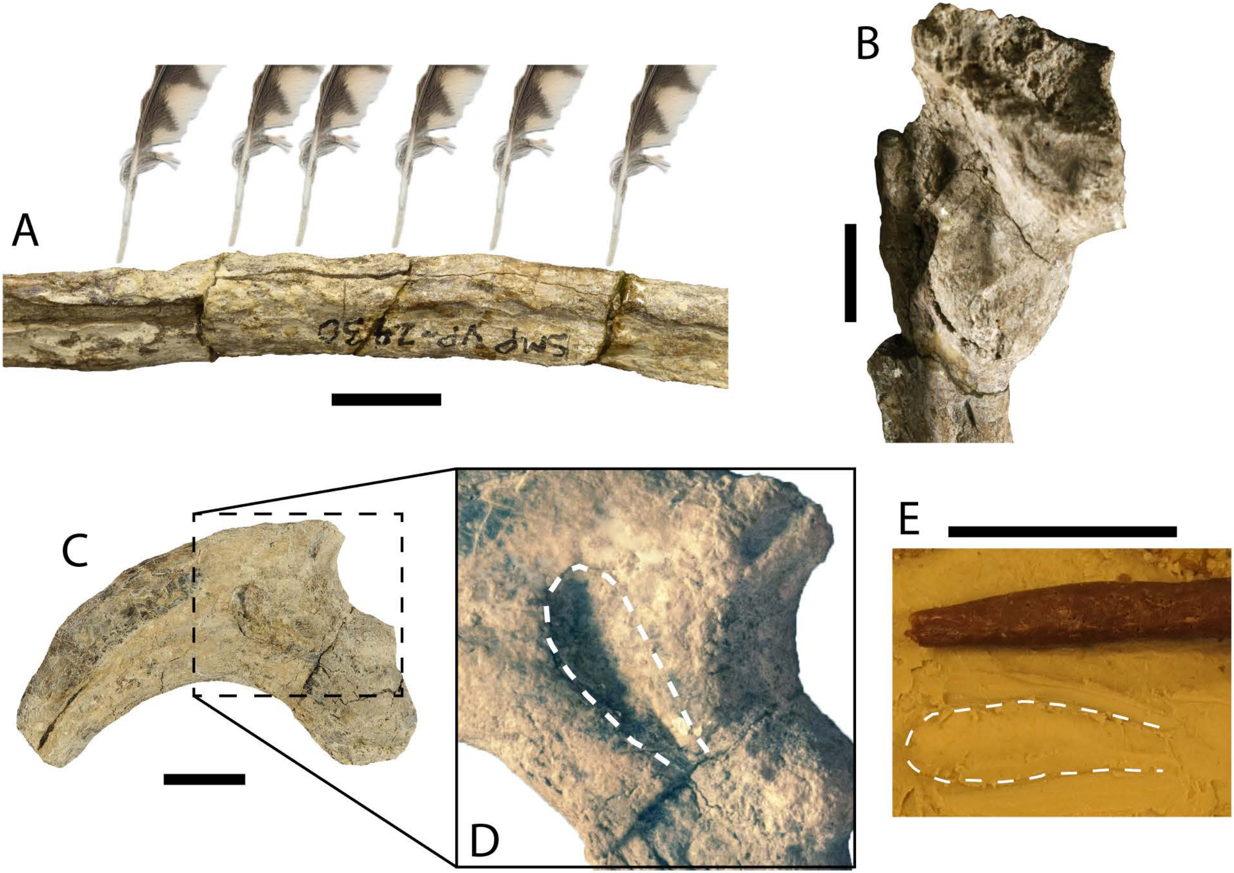

SUPP FIGURE 4. Other significant features of *Dineobellator notohesperus*, including; A, close up of ulna showing feathers where ulnar papillae are located along the ulnar ridge, feathers used are from *Megascops kennicottii*; B, proximal end of rib showing abnormal bone growth due to pathology; C, right manual ungual II, medial view, with close up, D, showing abnormal oblong concavity outlined in white dashed line; E, mold of the cavity, outlined in white dashed line, showing similarity with the distal portion of the ungual of a dromaeosaurid (*Deinonychus antirrhopus*, OMNH 50268, pedal ungual).

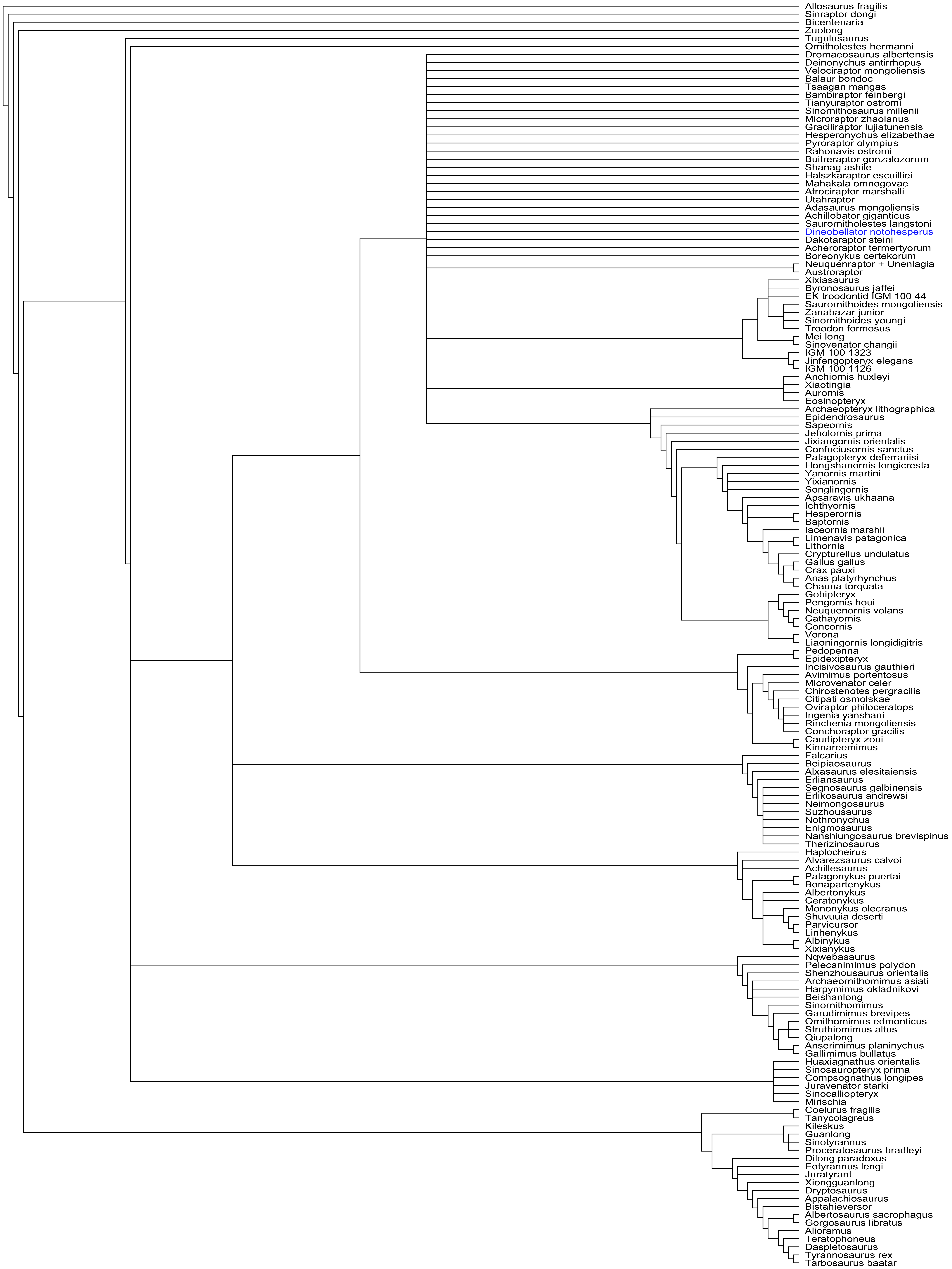

SUPP FIGURE 5. Strict consensus majority rule tree of the phylogenetic relationships of Coelurosauria, resulting in a mainly polytomic Dromaeosauridae. Phylogenetic analysis based on an updated version of the Theropod Working Group dataset, based particularly on the studies of Brusatte et al. (2014) and Cau et al. (2015, 2017). This results in 11590 most parsimonious trees with a length of 3317 steps, a Consistency Index of 0.328, and a Retention Index of 0.7612.

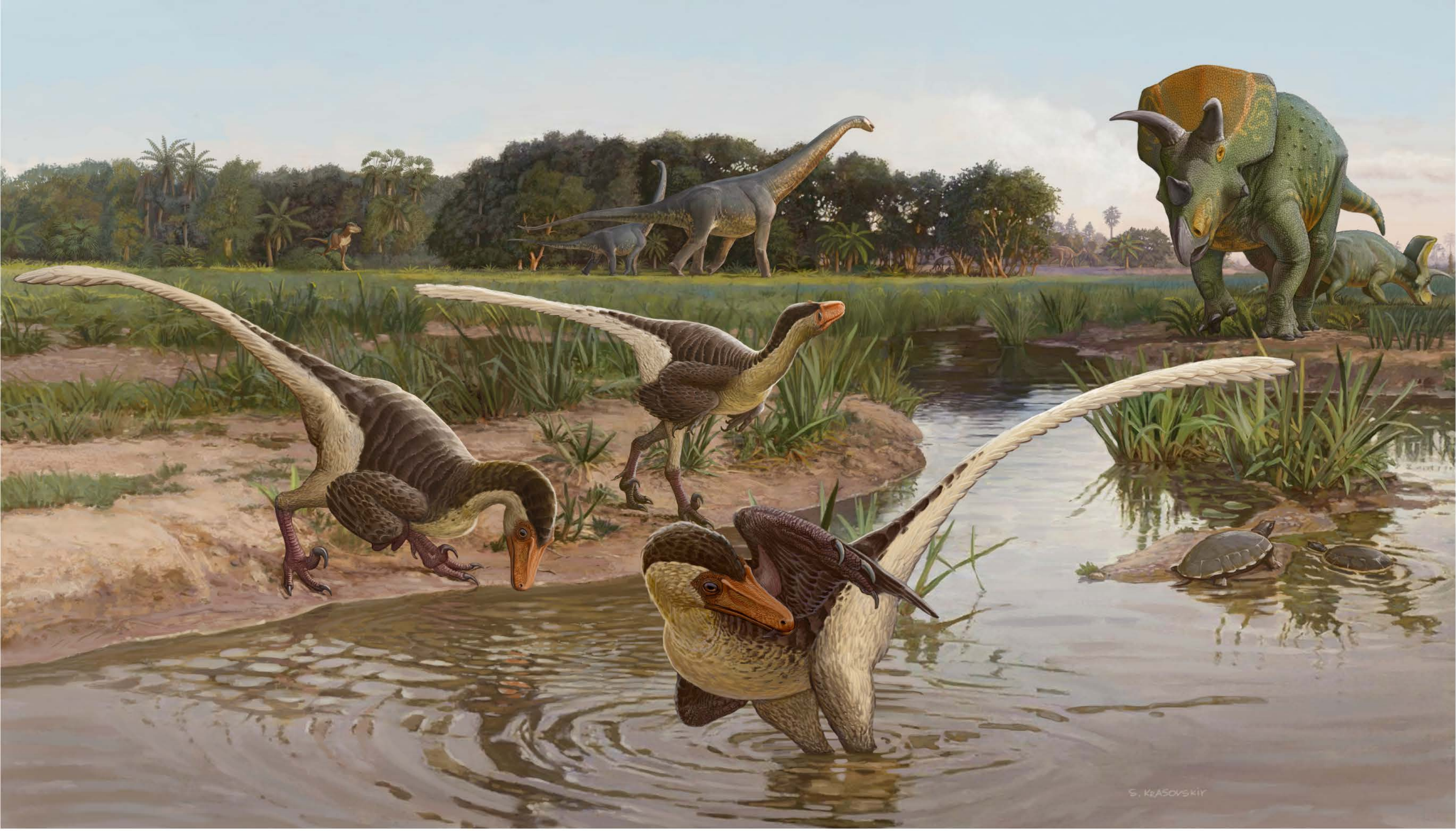

SUPP FIGURE 6. Life reconstruction of *Dineobellator notohesperus* depicting a possible scene from the Maastrichtian (approximately 66.5 Ma) in the San Juan Basin, New Mexico. Three *D. notohesperus* standing at the edge of a water source, with one in the foreground preening its feathers and another watching an *Ojoceratops fowleri* coming to the water's edge. Two *Alamosaurus sanjuanensis* are in the background, with a small tyrannosaurid watching from the forest edge. Artwork by Sergey Krasovskiy, with permission.
