# Supplementary material for: New Dromaeosaurid Dinosaur (Theropoda, Dromaeosauridae) from New Mexico and Biodiversity of Dromaeosaurids at the end of the Cretaceous
Source: Sci Rep. 2020 Mar 26;10:5105. doi: 10.1038/s41598-020-61480-7 (PMC7099077; doi:10.1038/s41598-020-61480-7)
Supplement: Supplementary file 2 — Supplementary information 2. [file 41598_2020_61480_MOESM2_ESM.pdf]

NEW DROMAEOSAURID DINOSAUR (THEROPODA, DROMAEOSAURIDAE) FROM NEW MEXICO AND BIODIVERSITY OF DROMAEOSAURIDS AT THE END OF THE CRETACEOUS

STEVEN E. JASINSKI, ROBERT M. SULLIVAN, and PETER DODSON

SUPPLEMENTAL INFORMATION

Supplementary Information Text

I. Abstract

II. Institutional Abbreviations

III. Description

IV. Apomorphies

V. Comparisons

VI. Table S1. Selected measurements of *Dineobellator notohesperus*

VII. Table S2. Tooth measurements of New Mexican dromaeosaurids

VIII. San Juan Basin Late Cretaceous Vertebrate Diversity

IX. Dromaeosauridae Hiatus in North America

X. Maastrichtian Dromaeosaurids

XI. Phylogenetic Analyses supplemental information

XII. Supplemental Character List for first data set (via Currie & Evans, 2019)

XIII. Character-Taxon Matrix for first data set

XIV. Supplemental Character List for second data set (via Cau et al., 2017)

XV. Character-Taxon Matrix for second data set

XVI. Phylogenetic Results

XVII. Geologic Ages used for Operational Taxonomic Units in the Phylogenetic Analyses

XVIII. Phylopics Sources

XIX. Summary

XX. Supplemental Literature Cited

I. ABSTRACT. Dromaeosaurids (Theropoda: Dromaeosauridae), a group of dynamic, swift predators, have a sparse fossil record, particularly at the time of their extinction near the Cretaceous-Paleogene boundary. Here we report on a new dromaeosaurid, *Dineobellator notohesperis*, gen. and sp. nov., consisting of a partial skeleton from the Upper Cretaceous (Maastrichtian) of New Mexico, the first diagnostic dromaeosaurid to be recovered from the latest Cretaceous of the southern United States (southern Laramidia). The holotype includes elements of the skull, axial, and appendicular skeleton. The specimen reveals a host of morphologies that shed light on new behavioral attributes for these feathered dinosaurs. Observed pathologies, in the form of potential wounds or injuries, are consistent with an active predatory lifestyle. Unique features on its forelimbs suggest greater strength capabilities in flexion, in conjunction with a relatively tighter grip strength in the manual claws. Aspects of the caudal vertebrae suggest greater movement near the tail base, aiding in agility and predation. Phylogenetic

analysis places *Dineobellator* within the Velociraptorinae. Its phylogenetic position, along with that of other Maastrichtian taxa (*Acheroraptor* and *Dakotaraptor*), suggests dromaeosaurids were still diversifying at the end of the Cretaceous. Furthermore, its recovery as second North American Maastrichtian velociraptorine suggests vicariance of North American velociraptorines after a dispersal eventduring the Campanian-Maastrichtian from Asia. Features of *Dineobellator* also imply that dromaeosaurids were active predators who occupied discrete ecological niches while living in the shadow of *Tyrannosaurus rex*, until the end of the dinosaurs' reign.

II. Institutional abbreviations. AMNH, American Museum of Natural History, New York, New York, USA; **GIN (IGM)**, Mongolian Institute of Geology, Ulanbataar, Mongolia; **HIII**, Henan Geological Museum, Zhengzhou, Henan Province, China; **IVPP**, Institute of Vertebrate Paleontology and Paleoanthropology; Beijing, China; **MPCA**, Museo Carlos Ameghino, Cipolletti, Rio Negro Province, Argentina; **OMNH**, Sam Noble Oklahoma Museum of Natural History, Norman, Oklahoma, USA; **PBMNH**, Palm Beach Museum of Natural History, Ft. Lauderdale, Florida, USA; **ROM**, Royal Ontario Museum, Toronto, Ontario, Canada; **SMP**, State Museum of Pennsylvania, Harrisburg, Pennsylvania, USA; **TMP**, Royal Tyrrell Museum of Paleontology, Drumheller, Alberta, Canada; **UALVP**, University of Alberta Laboratory for Vertebrate Paleontology, Edmonton, Alberta, Canada.

III. Description. A few small fragments of SMP VP-2430 are from the skull of *Dineobellator notohesperis*. A small rostromedial fragment of the right premaxilla is preserved, although it contains no teeth. It does, however, have portions of two alveoli, which are visible caudomedially (posteromedially). The element is fragmentary, and most of the outer lateral surface is incomplete. There is a smooth portion anteriorly where the anterior part of the left and right premaxillae would be in contact, roughly parallel with the alveoli orientation. The remnants of the alveoli medially are quite small (see SI tables 1–2 for all measurements) and relatively closely spaced, similar to the condition found in the anterior teeth of other dromaeosaurids. Portions of the outer, lateral surface are not smooth and may be pathologic. The left maxilla is represented by a small, sub-rectangular fragment. While a few small, questionable, inconsistent foramina are present, it is otherwise smooth on its lateral surface. Two partial alveoli are present medially. The more complete alveolus on the maxilla fragment is larger and more prominent than any preserved on the premaxilla fragment. Regardless, the small size of the alveoli suggests the fragment is from the posterior or caudal portion of the left maxilla.

A ?maxillary tooth is quite small and relatively gracile (Fig. 2F, Supp. Fig. 2J). It measures 12.0 mm total apical length, with a crown height of 11.3 mm. There are approximately 18–20 denticles per 5 mm on the distal (or posterior) carina (distal basal denticles), but no denticles on the mesial (or anterior) carina. There are 3.7 to 4.3 denticles per mm, most easily seen close to the base of the distal carina where the denticles are most well preserved (Fig. 2G, Supp. Fig. 2K), and the angle between the lines of 10% and 90% of the length of the exposed denticles normally falls between 86°–95° for denticles that are not worn down, broken, or deformed, with most falling just

under 90°. The denticles on the posterior carina are nearly rounded and show no traces of a hook, and the denticles tend to be shallow in relation to depth. On the distal end of the anterior edge of the tooth there is a wear facet that measures 6.70 mm along the curvature of the tooth from the distal tooth tip where it would occlude with a dentary tooth. The tooth does not constrict where it lies in the alveolus, has a concave-curve caudally, and would not be strongly raked in the alveolus.

A fragment of the left lacrimal is small, robust and sub-triangular with a rounded point laterally (Fig. 1G, Supp. Fig. 2A). It represents the lateral process with a small concavity on the caudodorsal portion of the medial edge (lacrimal fenestra) and a smooth surface along the rostroventral edge. The element projects rostrally (anteriorly) beyond the extent of the "sub-triangular" form, which presumably would have been 'T'-shaped if complete. The bone is incomplete with no sutural surfaces preserved.

Another small fragment is tentatively identified as part of the left nasal. It is sub-rectangular, with an enlarged medial surface, believed to be the sutural surface between the two nasals, and a flat dorsal surface. Several other problematic, and often gracile, bone fragments are tentatively identified as coming from the skull, but their exact identifications are uncertain.

A flat, trapezoidal fragment is identified as part of the right jugal (Fig. 1H, Supp. Fig. 2C). The entire element is slightly curved laterally toward its rostral (anterior) and caudal (posterior) ends. The element is quite flat and any medial or lateral projections that may have been present would have been on portions of the element that are not preserved. Preserved portions suggest a circular orbit. The majority of the margins of the

bone are incomplete, although caudodorsally the margin is complete and becomes rather thin and gracile. Dorsocaudally, along the postorbital process, a portion of the sutural surface between the jugal and the postorbital is preserved. This great extent of the sutural surface suggests a relatively deep jugal. There is also a partial foramen present anteriorly (rostrally) along the broken surface. This foramen may make up part of the contact between the jugal and the maxilla, although that is uncertain due to its preservation.

The braincase is incomplete. The condylar portion of the basioccipital is preserved (Supp. Fig. 2D). As only the posterior-most portion of the braincase is preserved, no cranial nerve passages are present, including the passages for cranial nerves XII and XIII. Thus, it is inferred that all cranial nerve passages are present anterior (or rostral) to the preserved portion. It is subcircular and the entire element is obliquely twisted. A portion of the basisphenoid is preserved, namely a portion including the right basipterygoid process (Fig. 1F, Supp. Fig. 2B). It preserves a small but prominent basipterygoid recess medially. The right basal tubera is present and robust, but incomplete medially. The hypophyseal fossa may be present but incomplete, as breakage makes its identification tentative. The basisphenoid extends anteriorly with a portion of the well-developed right basipterygoid process preserved. The anterior-most edge of this process is directed rostrolaterally. The caudal surface is smooth and the processes would have been separated by a deep U-shaped notch. Dorsomedial to this lies a portion of the basipterygoid recess. This recess is prominent externally, continues as a thin canal internally and is directed caudodorsally. Portions of the carotid canal are present on the medial edge of the basipterygoid recess and extends rostrocaudally. Dorsomedially, this

carotid canal contains multiple inconspicuous foramina. Ventrally, the opening for the carotid canal is 6.52 mm long and appears oval to sub-oval.

Several vertebrae and vertebral fragments are preserved in SMP VP-2430. One represents a nearly complete anterior caudal vertebra, the 1st caudal posterior to the sacrum (Fig. 1C, Supp. Fig. 2F). The centrum of the vertebra is complete, and portions of the diapophysis and parapophysis are preserved. The neural arch and spine are nearly complete and robust, albeit short. The anterior and posterior centrum surfaces are both oval to sub-rectangular, with both being wider then high. While the anterior surface is flat, the posterior is concave, making it opisthocoelous. Laminae are present on the lateral surfaces of the vertebra, with more on the right side, probably due to it being the better preserved of the two sides. The ventral surface has a distinct curvature when viewed laterally. Dorsal on the centrum, the transverse processes project laterally more than dorsolaterally, are sub-rectangular, short and gracile, and are midway through to just caudal to the midpoint of the centrum. On the dorsal surface at the neural canal lies a depression between the cranial and caudal surfaces of the centrum. A medial depression on the ventral surface of the centrum has a width of 5.10 mm. Craniolaterally there is a shallow depression on the transverse process, which becomes more inconspicuous laterally on the process. There is a medial depression running dorsoventrally on the cranial surface of the neural spine. The neural spine is low and robust, and flares laterally toward its posterior portion. Caudally there is a deep foramen just dorsal to the neural canal.

Another nearly complete vertebra represents a caudal vertebra approximately midway through the caudal series (~#8 – ~#12) (Fig. 1B, Supp. Fig. 2G–H). The centrum is complete and a portion of the neural arch is preserved, although none of the neural spine is preserved. The neural arch travels the majority of the anteroposterior length of the centrum. The centrum has undergone some shear deformation. It is amphicoelous with well-defined and conspicuous circular indents on both the cranial and caudal ends. These concavities on the cranial and caudal surfaces of the centrum are symmetrical and both lie near the dorsal center of the centrum on their respective surfaces. Both the anterior and posterior centrum faces are sub-rectangular to sub-trapezoidal, and the centrum is thinner midway between the two centrum faces.

A small section of fused caudal vertebrae is preserved with SMP VP-2430 (Fig. 1A, Supp. Fig. 2I). This fragment appears to represent complete and incomplete portions of at least four caudal vertebrae. The lateral surface of the vertebrae is generally smooth to slightly convex. Ridges that lie perpendicular to the vertebrae represent the contact surfaces between adjacent caudals. Two of the vertebrae are complete with lengths of 4.12 mm and 5.10 mm, respectively. Thin transverse 'lines' are present towards the dorsal half of the caudal section that may represent the prezygopophyseal rods found in various dromaeosaurid caudal sections that help give the tail added strength. Nevertheless, the vertebrae are quite small and it is believed they may have been posteriorly distal in the tail. Their fusion may be pathologic as they do not resemble a pygostyle and the tail does not seem to be shortened compared to other eudromaeosaurs. Several additional small elements and fragments are identified as portions of vertebrae but are too incomplete to offer further information.

Several bone fragments are identified as rib fragments. Two smaller fragments are fairly inconspicuous and do not offer much morphology. The larger of these two bones exhibits a long, thin depression that runs its length on two opposite sides. The other fragment appears to be taphonomically deformed and does not have any depressions. There is one larger and nearly complete left dorsal rib (Fig. 1E, Supp. Fig. 2E). Distally, this bone appears similar to other theropod ribs, and, while it is incomplete distally, it exhibits some folding and a long, thin depression laterally. Proximal to this depression is a ridge that wraps toward the posterolateral surface and continues to the proximal edge. The proximal surface is expanded and has a "D", or semi-circular, shape to it (Supp. Fig. 4B). Just ventrolateral to this proximal expansion is another expansion of the element, which wraps medially to the proximal edge. The bone preserves several areas of irregular morphology on its surface, with areas of slight expansion or depression along the rib shaft. This irregular morphology shows bone restructuring and is thought to be pathologic in origin.

The nearly complete right humerus (Fig. 1I, Supp Fig. 3A) lacks small portions proximally, including parts of the deltopectoral crest, and the distal end. The preserved portion of the humerus measures 185.78 mm, with an estimated total length of 215 mm. What is preserved of the humerus indicates that the proximal portion is quite thin and gracile. Its proximal edge is bent somewhat medially. The deltopectoral crest is thinner and more gracile than the shaft, projects anteriorly, and lies approximately perpendicular to the long axis of the humeral head. It is relatively elongate at approximately 31% the total length of the humerus. While not complete, the distal edge of the crest creates a sharp angle with the shaft of the humerus. The muscle scar of the latissimus dorsi is

present proximally on the lateral surface of the humerus. The medial depression near this muscle attachment area is pronounced and continues distally from the proximal portion for approximately 1/3 the humerus length. On the lateral surface, and anterior to the depression, lies a ridge that runs longitudinally down the bone. The raised ridge on the proximolateral surface of the bone is approximately 93.03 mm long. It terminates just distal to the distal extent of the deltopectoral crest. There is some slight bend midway along the humeral shaft that is inferred to be, at least partially, due to taphonomic deformation. Distal to this bending, the shaft is sub-round to oval in cross-section. Distally, the humerus expands mediolaterally and there is a depression running longitudinally down the shaft on the caudal surface. The humerus is also hollow in cross-section. There is some slight longitudinal crushing of the shaft towards its middle on the distal surface, but this is undoubtedly due to the hollow nature of the bone.

The right ulna is preserved as a long, thin, bowed bone (Fig. 1J, Supp. Fig. 3B). The distal edge is broken, but the proximal portion is complete. The preserved portion has a total proximodistal length of 100.96 mm, indicating a total estimated length of approximately 140 mm. The ulna appears to have a shallow trochlea and fairly inconspicuous, transversely broad olecranon process. The process is nearly complete and sub-triangular. The ulna flares out mediolaterally just distal to the olecranon process and the sigmoid notch of the ulna. The shaft of the ulna is curved and flares to become thin and wide distally. There is an uneven texture along the ventral ulnar ridge that bears at least 6 protuberances, identified as ulnar papillae or quill knobs (Supp. Fig. 4A). This suggests approximately 12–14 secondary remiges. These protuberances start approximately 4.70 mm from the proximal end.

The proximal right metacarpal III is preserved, although the shaft has been partially crushed mediolaterally (Fig. 1K, Supp. Fig. 3C–D). Nevertheless, the shaft becomes thinner mediolaterally away from the proximal edge. The proximal edge is slightly concavely-curved. On the distally preserved surface, the dorsal portion of the bone is wider (mediolaterally) than the ventral portion, giving the bone a generalized "P" shape. On the lateral surface of the shaft, there is a narrow groove, although this may be due to deformation. The entire element is slightly curved with the proximal and distal ends being slightly more anterior than the rest of the shaft.

The nearly complete right manual ungual II is preserved, missing only the distal-most tip (Fig. 1L, Supp. Fig. 3J–L). It measures 45.64 mm long from the ventral edge of the articular surface to the preserved distal tip, with an estimated total length of approximately 50 mm when complete. The claw is strongly curved, with a significantly-pronounced flexor tubercle along its proximoventral edge. However, when viewed medially or laterally, the claw exhibits a significant arch, with the dorsal surface approximately 114° in relation to the articular surface. There is a lateral depression or groove that runs along its length toward the distal tip. This groove starts between the articular surface and flexor tubercle, and travels along the length of the claw toward the dorsal surface distally. Medially a similar groove is present, although there is a prominent foramen in the groove just distal to the articular surface. The two grooves are offset on the two sides of the ungual as the groove on the medial surface also does not get closer to the dorsal surface, unlike the lateral groove. Near the proximal end of the groove on the medial surface lies a distinct and prominent gouge mark (Supp. Fig. 4C). This mark starts as a furrow proximoventrally and terminates in a prominent, but small, depression closer

to the dorsal edge (Supp. Fig. 4D). The gouge has an approximate width of 3 mm and extends for a length of 9 mm. This feature does not show abnormal morphology suggesting it is not due to infection or pathology on and under the keratinous sheath. The flexor tubercle is perpendicular to, angled almost straight down (ventrally) from, the articular surface of the ungual. Immediately dorsal to the articular surface is a ridge running up the middle of the ungual, with a depression present on either side (medially and laterally). On its proximodorsal-most surface, there is a faint, slight lip. Directly ventral to the articular surface is another slight lip or ridge that comes to two lateral points or projections.

The right femur is a robust, incomplete element missing its proximal-most (including most of the femoral head) and distal portions (Fig. 1D, Supp. Fig. 3E). The preserved portion of the element is 68.83 mm long, with a total estimated length of approximately 275 mm. In the preserved portion the bone projects medially making up the medioventral portion of the femoral head. The shaft is sub-oval in cross-section. The projection infers that the head would have been twisted on the shaft of the femur. The shaft begins to flare anteroposteriorly toward its distal end. A slight raised portion of the bone posteromedially is inferred to represent part of the fourth trochanter.

Several metatarsals are present, albeit incomplete. Right metatarsal I is incomplete and somewhat twists about its shaft. There is also a relatively large foramen present towards its distal end, measuring 3.55 mm by 2.30 mm. Around this foramen is a relatively pronounced and rounded rim. The outer surface near the proximal end is eroded and incomplete. The right metatarsal II is represented by the proximal (Fig. 1M, Supp.

Fig. 3F–G) and distal portions, with the middle part of the shaft missing. The preserved shaft of metatarsal II is relatively thin and gracile and is inferred to have been sub-circular to sub-rectangular in cross-section. Proximally, the bone is sub-triangular, with a distinct groove (or furrow) from the proximal edge and extending approximately halfway down the proximal section. Posterolaterally, there is a depression near the distal end. On the anterior portion of the distal end, the bone flares out anteriorly to a relatively sharp point. The distal end did not undergo any significant deformation. There is a depression on the dorsal surface of the proximal end that runs distally from between the two condyles. Another depression is present on the distomedial surface of the bone, lying just proximal to the left condyle. The distal portion of the right metatarsal II is incomplete due to deformation and erosion and is sub-rectangular to sub-triangular. Distally the element flares out into two separated projections, or condyles. On the medial surface, just proximal to the distal end, lies a prominent foramen. On the lateral surface, approximately even with the foramen, lies a small, raised protuberance. There are no traces of a tuber where the extensor surface lies. Right metatarsal III is represented by the proximal end, with the shaft thinner proximally and flares out steadily toward the distally preserved surface (Fig. 1N, Supp. Fig. 3H–I). The dorsal edge, while having been crushed somewhat mediolaterally, is still wider than the ventral edge. The dorsal and ventral edges of the proximal surface also taper to a ridge that runs mediolaterally along the middle of this surface. The bone flares slightly toward the dorsolateral and ventromedial regions on the distal surface.

A small bone, questionably identified as a left astragalus, is incomplete and, distally, projects anteriorly to form a "basin" that would contact the distal tibia. A distal

projection gives the element an anteroposterior depth of 5.35 mm. Proximally the bone projects to a sub-circular proximal edge, which is relatively robust. The entire anterior surface has an inconspicuous depression, with the outer edges flaring more anteriorly, forming the tibial facet. Calcaneum and fibular facets are not preserved.

Two bone fragments are identified as portions of a pedal ungual, namely from right digit III (Fig. 2M, Supp. Fig. 3M). A small, middle portion is missing, but the proximal edge and most of the distal portions are present. The proximal fragment is 13.23 mm long (proximodistally) and the distal fragment is 32.03 mm long, allowing us to estimate a total length of a little over approximately 55 mm long when complete. The dorsal portion of the proximal surface shows the articular surface. Ventral to the articular surface lies the flexor tubercle, which is significantly smaller and less pronounced than that of the manual ungual II. While there is a slight concave curvature below the articular surface, the flexor tubercle again projects perpendicular to the articular surface. The flexor tubercle, while smaller than that of the manual ungual, is relatively large. The distal portion of the pedal ungual is missing the tip. Grooves are present on both the lateral and medial surfaces of the claw and offset from each other, like those of the manual ungual. There is also a ridge dorsal to the lateral depression, and the depression proceeds toward the dorsal surface as it moves distally, while the medial depression or groove does not, as in manual ungual II. There is a second, less conspicuous depression or groove ventral to the main one on both the lateral and medial surfaces. The pedal claw itself is far thinner (dorsoventrally) than the manus claw and is not nearly as strongly curved. Numerous bone fragments have been left unidentified as they are too fragmentary to allow for identification.

IV. Apomorphies. *Dineobellator notohesperis* is distinguished from other dromaeosaurids based on several apomorphies throughout various portions of its skeleton. These apomorphies include: an offset of lateral grooves on the manual ungual with a conspicuous mediodorsal groove proximally, a sharp angle of the distal deltopectoral crest of the humerus, opisthocoelous anterior caudal vertebrae, short and robust neural spines on the anterior caudal vertebrae, gracile and sub-rectangular transverse processes on the anterior caudal vertebrae, distinct round concavities on the cranial and caudal centrum surfaces in mid-caudal vertebrae, and an enlarged flexor tubercle on the pedal ungual. The distinct offset nature of the longitudinal grooves of the manual ungual are often found on the pedal unguals of various dromaeosaurid taxa and are barely offset in one other taxon (*Boreonykus certekorum*), but not in other dromaeosaurids. The distinct mediodorsal groove proximally near the articular surface is not present in other dromaeosaurid taxa and suggests enlarged areas for the digit extensors (extensor digitorum brevis). The flattened proximal edge of the humerus of *Dineobellator* is distinct from the sigmoidal shape in other dromaeosaurids (e.g., *Saurornitholestes*, *Bambiraptor*, *Deinonychus*). The sharp angled curvature of the distal portion of the deltopectoral crest is unique among dromaeosaurids and, indeed, all theropods, although it is less gentle in *Deinonychus* (AMNH 3015). The gentle curvature of the deltopectoral crest allows for muscle attachment to be more fan-shaped. However, the sharp bend in the distal deltopectoral crest may be due to an overall enlargement of the deltopectoral crest in *Dineobellator*. Indeed, the deltopectoral crest is relatively larger in *Dineobellator* [estimated 31% of total humeral length, than several other dromaeosaurids with preserved humeri (e.g., 20.5% in *Bambiraptor feinborgorum*, 23.5%

in *Dakotaraptor steini*, 25% in *Saurornitholestes langstoni* and 28% in *Deinonychus antirhoppus*)]. It is unknown if the relative length of the deltopectoral crest changes through ontogeny, although if *Bambiraptor* is a juvenile, it may suggest a relatively smaller deltopectoral crest in juveniles, implying an older ontogenetic age for *Dineobellator*. In dromaeosaurids, anterior caudal vertebrae are acoelous or amphiplatyan, making the concave posterior centrum surface distinct among known dromaeosaurids. While opisthocoelous anterior caudal vertebrae are not known in other dromaeosaurids, they have been found in the caenagnathid theropod *Gigantoraptor erlianensis* (Xu et al., 2007). The ventral surface is curved ventrally, while those of other dromaeosaurids (e.g., *Deinonychus*) are angled but not curved in lateral view. The neural spine is short and robust while the transverse processes are thin and gracile in caudal vertebra 1 of *Dineobellator*. The transverse processes are sub-rectangular, distinct from *Deinonychus* where they are sub-triangular and *Velociraptor* where they are enlarged and fan out distally. Additionally, the centrum surfaces, particularly those posteriorly, are rounded rather than distinctly oval to sub-rectangular in *Dineobellator*. The sub-circular concavities on the cranial and caudal surfaces of the centra of the midcaudal vertebrae are symmetrical and have not yet been seen in other dromaeosaurid caudal vertebrae. While the flexor tubercle is smaller in the pedal ungual than in the manual ungual, it is still enlarged in comparison to those in other dromaeosaurid taxa (e.g., *Boreonykus*, *Dakotaraptor*, *Deinonychus*), and potentially most similar in relative size to *Utahraptor* pedal unguals. Additionally, the smaller secondary grooves ventral to the main lateral grooves on the pedal ungual are not present in other dromaeosaurids.

V. Comparisons. The vertical orientation of the premaxilla suggested by the premaxilla fragment helps distinguish *Dineobellator* from several other dromaeosaurids with posterodorsally angled premaxilla, including *Sinornithosaurus*, *Halszkaraptor*, and *Utahraptor*. The nasals are flat dorsally, similar to *Bambiraptor* and *Deinonychus*, while the jugal would have been dorsoventrally deep, similar to most other dromaeosaurids other than *Bambiraptor* and *Halszkaraptor*. The flat caudal surface of the basal tubera distinguishes it from *Velociraptor mongoliensis* (Norell and Makoviky, 2004; Norell et al., 2004) and *Tsaagan mangas* (Norell et al., 2006), while the U-shaped notch between the tubera distinguishes it from *Dromaeosaurus* (Currie, 1995). The subcircular opening for the foramen magnum helps distinguish *Dineobellator* from *Tsaagan mangas*. The teeth would not have been strongly raked posteriorly, distinct from *Atrociraptor*, *Bambiraptor*, and *Deinonychus*. The posterior denticles are not apically hooked, making them similar to those of *Acheroraptor* and *Tsaagan*, but distinct from *Atrociraptor marshalli* and *Saurornitholestes langstoni* (Larson, 2008). The presence or absence of denticles on the anterior carina is highly variable among dromaeosaurids, but, in addition to *Dineobellator*, they are also absent in *Bambiraptor* and several Asian taxa including *V. osmolskae*, *Tsaagan*, and *Linheraptor*. The relatively elongate humerus in *Dineobellator* distinguishes it from most other known eudromaeosaurs except *Saurornitholestes langstoni* and *Bambiraptor*, although this condition is also present in some microraptorines and unenlagiines. The orientation and gracility of the deltopectoral crest in relation to the humeral shaft also help distinguish *Dineobellator* from the unenlagiines. In addition, a few other dromaeosaurid specimens have been reported from the San Juan Basin and the Naashoibito Member in particular. Jasinski et al. (2011) reported on two

isolated dromaeosaurid teeth from the Naashoibito Member. SMP VP-2505 was reported as a large dromaeosaurid tooth by Jasinski et al. (2011), but referred to a tyrannosaurid by Williamson and Brusatte (2014), and we agree with the latter study here. SMP VP-2595, an isolated tooth, was referred to Dromaeosauridae by Jasinski et al. (2011). This tooth does represent a dromaeosaurid, with rounded denticles, slightly less dense denticles than *Dineobellator notohesperis* with approximately 17 per 5 mm on the posterior carina, and lacking denticles on the anterior carina. While the tip is missing, SMP VP-2595 is larger than the tooth preserved with SMP VP-2430, with an apical length of 14.25 mm, a crown height of 12.59 mm, a fore-aft basal length of 8.41 mm, and a basal width of 4.71 mm. Williamson and Brusatte (2014) reported the presence of dromaeosaurids in the member as well based on NMMNH P-32814, which they referred to their 'Dromaeosauridae Morphotype A'. This tooth has rounded denticles that are not hooked, as in *Acheroraptor*, has 25 denticles per 5 mm on the posterior carina, and does have small anterior denticles. It has an apical length of 6.86 mm, a crown height of 5.13 mm, a fore-aft basal length of 3.46, and a basal width of 1.66 mm. While SMP VP-2595 may represent *Dineobellator notohesperis*, NMMNH P-32814 appears distinct, and suggests the presence of at least two dromaeosaurid taxa in the Naashoibito Member. While the late Campanian *Saurornitholestes sullivani* was also collected in the San Juan Basin (De-na-zin Member, Kirtland Formation), it was based on a holotype frontal (SMP VP-1270, Sullivan and Lucas, 2000; Jasinski, 2015). Isolated dromaeosaurid teeth from the De-na-zin Member have been collected (SMP VP-1901), but these also differ from *Dineobellator*. They are gently curved, have slightly apically hooked denticles, less dense denticles (14–15 denticles per 5 mm), and possess anterior denticles. It has an apical length of 14.85 mm, a

crown height of 12.62 mm, a fore-aft basal length of 6.75, and a basal width of 3.36 mm. The teeth are believed to be from *S. sullivani*, making the two taxa distinct based on tooth morphologies.

VI. Table S1. Selected measurements of *Dineobellator notohesperus* (SMP VP-2430).

Element	length (in mm)	notes
**Premaxilla (right)**		
length	*8.86	
depth	*12.38	
alveolus 'a' length	2.28	
alveolus 'b' length	1.81	
**Maxilla**		
length (rostrocaudal)	*6.34	
depth (dorsoventral)	*9.09	
alveolus 'a' length	3.03	
**?Maxillary Tooth**		
apical length	12	
crown height	11.3	
fore-aft basal length	7.78	
basal width	4.15	
wear facet length	6.7	
**Lacrimal (Left)**		
length	*9.00	
depth	*6.96	
**Nasal (left)**		
length	*18.51	
width	*6.71	
**Jugal (right)**		
length (craniocaudal)	*37.17	
depth (dorsoventral)	*20.66	maximum preserved depth measured near posterior of bone
**Basioccipital**		
length (anteroposterior)	*14.79	
width (mediolateral)	*16.04	
**Basipterygoid Process (right)**		
length (anteroposterior)	31.67	between the basal tubera and the basipterygoid process
height (dorsoventral)	13.8	measured posteriorly at the basal tubera
width A (mediolateral)	22.06	measured across the posterior portion with the basal tubera
width B (mediolateral)	11.58	measured across the middle constriction just anterior to the carotid canal
width C (mediolateral)	8.81	measured across the anterior portion with the basipterygoid process
**Anterior Caudal Vertebra #1**		
length (anteroposterior)	26.4	
height (dorsoventral)	30.32	from ventral edge to dorsal edge of neural spine
width (mediolateral)	15.9	
anterior centrum surface height (dorsoventral)	12.75	
anterior centrum surface width (mediolateral)	19.3	
posterior centrum surface height (dorsoventral)	14.1	
posterior centrum surface width (mediolateral)	21.95	
neural spine height (dorsoventral)	15.3	
**Midcaudal Vertebra (~8–~12)**		
length (anteroposterior)	32.56	
anterior width (mediolateral)	15.23	
posterior width (mediolateral)	14.71	
mid-length width (mediolateral)	10.86	
neural arch length (anteroposterior)	28.33	
**Distal Caudal Vertebrae**		
fused section length (anteroposterior)	*19.95	
fused section height (dorsoventral)	9.5	maximum height
Caudal vertebra A length (anteroposterior)	4.12	this represents one of the two complete distal caudal vertebrae in the fused section
Caudal vertebra B length (anteroposterior)	5.1	this represents one of the two complete distal caudal vertebrae in the fused section
**Rib A**		largest incomplete rib
length (proximodistal)	*100.55	
width A (anteroposterior)	25.12	at proximal end across the "D"
width B (anteroposterior)	10.35	at distally preserved end
**Humerus (right)**		
length (proximodistal)	*185.78	
shaft diameter	17.25 to 18.52	
shaft width	17.1	proximal, distal to deltopectoral crest
shaft length	*91.27	distal to deltopectoral crest
**Ulna (right)**		
length (proximodistal)	*100.96	
width	15.71	maximum width measured across trochlea and olecranon process
shaft width	12	average shaft width
**Metacarpal III (right)**		
length (proximodistal)	*72.75	
width (anteroposterior)	*16.68	measured at proximal end
**Manual Ungual II (right)**		
length (proximodistal)	*45.64	measured from ventral edge of proximal articulartion surface to preserved distal tip
length (proximodistal)	*46.97	measured from ventroproximal edge of flexor tubercle to preserved distal tip
height (dorsoventral)	29.95	measured at the proximal end (from dorsal edge of articulation surface to ventral edge of flexor tubercle)
height (dorsoventral)	16.46	measured at the midlength of the claw
height (dorsoventral)	9.1	measured at the preserved distal tip
articulation surface height (dorsoventral)	18	
articulation surface width (mediolateral)	8.33	
flexor tubercle height (dorsoventral)	16.75	
flexor tubercle width (mediolateral)	6.7	

Element	length (in mm)	notes
**Femur (right)**		
length (proximodistal)	*68.83	
width	*20.18	
**Metatarsal I (right)**		
length (proximodistal)	*11.43	
width	7.2	maximum width measured distally
**Metatarsal II (right)**		
length (proximal section, proximodistal)	*46.79	
shaft width (proximodistal section)	18.14	maximum width measured at preserved distal end
shaft width (proximodistal section)	8.75	proximal-most shaft width
length (distal section, proximodistal)	*33.3	
shaft width (distal section)	11.52	maximum width measured at distal end
**Metatarsal III (right)**		
length (proximodistal)	*47.14	
width	14.04	maximum width measured at preserved distal end
**?Astragalus (left)**		
length (proximodistal)	*10.6	
width	*6.82	
**Pedal Ungual III (right)**		
length (proximal section, proximodistal)	*13.23	
height (proximal section, dorsoventral)	21.3	
articulation surface height (dorsoventral)	10.65	
articulation surface width (mediolateral)	4.12	
flexor tubercle height (dorsoventral)	7.1	
flexor tubercle width (mediolateral)	3.53	
length (distal section, proximodistal)	*32.03	
height (distal section, dorsoventral)	11.95	measured at preserved proximal end
height (distal section, dorsoventral)	9.05	measured at preserved distal end

*incomplete element, measurement as preserved

VII. Table S2. Tooth measurements of dromaeosaurid teeth from the Late Cretaceous of the San Juan Basin, New Mexico.

Specimen number	Taxonomic identity	element identity	apical length	crown height	fore-aft basal length	basal width	denticles per 5 mm
SMP VP-2430	*Dineobellator notohesperus*	?maxillary tooth	12	11.3	7.78	4.15	18–20
SMP VP-2595	Dromaeosauridae indeterminate	tooth	14.25	12.59	8.41	4.71	17
NMMNH P-32814	Dromaeosauridae morphotype A	tooth	6.86	5.13	3.46	1.66	25
SMP VP-1901	cf. Saurornitholestes sullivani	tooth	14.85	12.62	6.75	3.36	14–15

*measurements in mm
Identity of NMMNH P-32814 from Williamson and Brusatte (2014)

VIII. San Juan Basin Late Cretaceous vertebrate diversity. The diversity of Late Cretaceous vertebrates from the San Juan Basin of New Mexico has undergone significant revision and been increasing over the last approximately two decades. This increase in diversity has been driven by consistent collecting in the Upper Cretaceous strata of the region. Newly named species from the Fruitland, Kirtland, and Ojo Alamo formations include the nanhsiungchelyid turtle *Basilemys gaffneyi* (Sullivan et al., 2013); the ?azhdarchid pterosaur *Navajodactylus boerei* (Sullivan and Fowler, 2011); and several dinosaurs including the tyrannosaurid *Bistahieversor sealeyi* (Carr and Williamson, 2010); the dromaeosaurid *Saurornitholestes sullivani* (Jasinski, 2015) and proposed dromaeosaurid "*Saurornitholestes*" *robustus* (Sullivan, 2006a; now believed to be a troodontid by Evans et al., 2014); the caenagnathid *Ojoraptorsaurus boerei* (Sullivan et al., 2011); the hadrosaurine hadrosaurids *Anasazisaurus horneri* and *Naashoibitosaurus ostromi* (Hunt and Lucas, 1993); the ankylosaurids *Nodocephalosaurus kirtlandensis* (Sullivan, 1999), *Ahshislepelta minor* (Burns and Sullivan, 2011), and *Ziapelta sanjuanensis* (Arbour et al., 2014); the nodosaurids *Edmontonia australis* and *Glyptodontopelta mimus* (Ford, 2000); the ceratopsids *Ojoceratops fowleri* (Sullivan and Lucas, 2010) and *Titanoceratops ouranos* (Longrich, 2011); and the pachycephalosaurids *Sphaerotholus goodwini* (Williamson and Carr, 2002) and *Stegoceras novomexicanum* (Jasinski and Sullivan, 2011; 2016). Newly named genera for existing species include *Denazinemys* for the baenid turtle *D. nodosa* (Lucas and Sullivan, 2006); *Scabremys* for the baenid turtle *S. ornata* (Sullivan et al., 2013); and *Denazinosuchus* for the goniopholidid mesoeucrocodylian *D. kirtlandicus* (Lucas and Sullivan, 2003).

The Fruitland through Ojo Alamo formations span the Upper Campanian through near the very end of the Cretaceous in northwestern New Mexico (Supp. Fig. 1). The Ojo Alamo Formation spans the Cretaceous–Paleocene boundary and has Paleocene sediments as well (Jasinski et al., 2011), although these younger strata are not believed to contain in-situ dinosaur fossils (Lucas et al., 2009; Koenig et al., 2012; contra Fassett and Lucas, 2000; Fassett et al., 2002, 2011; Fassett, 2009). Even so, most of the recently named taxa have come from the Upper Campanian Fruitland and Kirtland formations, as these are more richly fossiliferous than the younger Maastrichtian Naashoibito Member of the Ojo Alamo Formation (Jasinski et al., 2011).

Armstrong-Ziegler (1978, 1980) was the first to report material potentially referable to the Dromaeosauridae from the San Juan Basin, although it was identified as an indeterminate ?dromaeosaurid. Lehman (1981) noted a complete right metatarsal I similar to *Stenonychosaurus* that he identified as dromaeosaurid or troodontid. However, the specimen is part of a private collection and was not considered further by Jasinski et al. (2011), nor will it be in the present study. Lucas et al. (1987) and Hunt and Lucas (1993) listed the presence of indeterminate dromaeosaurids in the Fruitland, Kirtland, and Ojo Alamo formations, although this material is made up almost exclusively of isolated teeth. Sullivan and Lucas (2000) described the first potentially diagnostic material, an isolated left frontal (SMP VP-1270) from the Late Campanian De-na-zin Member (Kirtland Formation), which they attributed to *Saurornitholestes langstoni*. This specimen was later re-studied and determined to represent a distinct dromaeosaurid, which Jasinski (2015) named *S. sullivani*. Sullivan (2006a) later described another isolated left frontal from the De-na-zin Member which he referred to a new species, *S.*

robustus. However, this was restudied by Evans et al. (2014), who determined it represented an indeterminate troodontid. Jasinski et al (2011) reviewed the fossil vertebrates from the Maastrichtian Ojo Alamo Formation and reported the presence of indeterminate dromaeosaurids from both tooth and non-tooth material. SMP VP-2430, the specimen described herein, was also first reported by Jasinski et al. (2011) as an indeterminate dromaeosaurid. Jasinski et al. (2011) noted that there may be multiple dromaeosaurid taxa present, although they were focused on the Naashoibito Member. Williamson and Brusatte (2014) studied theropod teeth recovered from the Upper Cretaceous strata of the San Juan Basin. They determined there were at least two types of dromaeosaurids based on differing tooth morphotypes, although they noted only one of these types ("Dromaeosauridae Morphotype A") in the Naashoibito Member.

IX. Dromaeosauridae Hiatus in North America. While North American dromaeosaurids are known from the Barremian in multiple taxa (*Yurgovuchia* and *Utahraptor*) (Senter et al., 2012; Kirkland et al., 1993), following *Deinonychus* in the early Albian (Ostrom, 1969; Brinkman et al., 1998) there is a significant hiatus in their fossil record. This hiatus or gap lasts until the middle to late Campanian with the appearance of *Dromaeosaurus* (Turner et al., 2012; Jasinski and Dodson, 2015). This approximately 30-million-year hiatus may be due, in part, to preservational bias against small and rarer taxa, making it difficult to determine if their absence is real or an artefact of the fossil record (Jasinski et al., 2015, Lucas et al. 2016). While their presumed extinction may be due to lack of preservation or rarity in Cretaceous ecosystems, they are not the only dinosaurs presumed missing from North America during this time range only to return near the end of the Cretaceous. This gap has been investigated in the Sauropoda

as well, with their reappearance in North America during the Maastrichtian with *Alamosaurus* (D'Emic et al., 2010; Jasinski et al., 2011; Jasinski and Dodson, 2015; Jasinski et al., 2015). Part of this is undoubtedly due to a lack of stratigraphy and fossils within this time range, and so any fossils dating to this range could be important for helping understand what was happening in their evolution during this hiatus.

X. Maastrichtian Dromaeosaurids: *Dineobellator* is the first diagnostic dromaeosaurid from the Maastrichtian of New Mexico (southern Laramidia). Features of the forelimb, unguals, and caudal vertebrae distinguish it from other known North American Maastrichtian dromaeosaurids. *Dineobellator* is similar size to *Acheroraptor*, but distinct from the far larger *Dakotaraptor*. While DePalma et al. (2015) note the presence of two different-sized individuals of *Dakotaraptor*, they attributed this to sexual dimorphism. The only potential overlap in material between *Acheroraptor* and *Dakotaraptor* are teeth, with estimates of 22–25 and 22–23 denticles per 5 mm for the posterior denticles of each, respectively. This still leaves open the possibility of them being different ontogenetic stages of the same species, although little work has investigated ontogeny in dromaeosaurids (e.g., Parsons and Parsons, 2015), and lack of overlapping material makes this difficult to determine in the case of these two taxa. Additionally, based on the current available material and phylogenetic analysis, these two taxa are found in two distinct areas of the phylogeny, with *Dakotaraptor* recovered as a basal member of the Dromaeosaurinae and *Acheroraptor* as a basal member of the Velociraptorinae. Smaller body-sized dromaeosaurids clearly persisted into the late Maastrichtian throughout Laramidia. Large body-sized dromaeosaurids first appeared in southern North America during the Barremian and were found throughout much of what is today the United States

by the Aptian-Albian (Jasinski and Dodson, 2015; Jasinski et al., 2015), but fossil evidence is missing after this time until the Maastrichtian. However, *Achillobator giganticus*, a large dromaeosaur from the Cenomanian-Santonian of Mongolia falls in this gap in Asia (Hicks et al., 1999, Perle et al., 1999; Makovicky et al., 2005). The Unenlagiinae from South America have several large-bodied taxa, including the Turonian-Coniacian *Unenlagia* (Novas and Puerta, 1997; Calvo et al., 2004) and the early Maastrichtian *Austroraptor* (Novas et al., 2009). Large-bodied dromaeosaurids may have gone extinct in North America during this interval while they remained in other regions of the world. They may have then returned to North America during the Maastrichtian. It may be that large-bodied dromaeosaurids migrated back to North America during the Maastrichtian, after the potential reappearance of dromaeosaurids in North America during the middle to late Campanian, with tyrannosaurs, in particular, filling some of the available ecological niches of medium- to large-bodied terrestrial predators during this time.

XI. Phylogenetic Analyses supplemental information.

We included *Dineobellator notohesperis* in two well sampled data sets focusing on the relationships of coelurosaurian theropod dinosaurs and dromaeosaurid theropods. Multiple taxa were added to the used datasets, or new characters were scored if the taxa had previously not been scored for all available characters. In addition to *Dineobellator notohesperis* (SMP VP-2430) were *Dakotaraptor steini*, *Boreonykus certekorum*, and *Acheroraptor termertyorum* were added to the second, larger dataset (TWG dataset). *Dakotaraptor* was scored based on descriptions and figures from DePalma et al. (2015),

using mainly the holotype (PBMNH.P.10.113.T). *Boreonykus certekorum* was scored based on specimens described and figures presented by Bell and Currie (2016). *Acheroraptor termertyorum* was scored based on material described and figured by Evans et al. (2013).

The first data set was originally developed by Longrich and Currie (2009) to investigate the relationships of *Hesperonykus elizabethae*. This data set was then further edited and updated by Evans et al. (2013) to investigate the relationships of *Acheroraptor temertyorum*. Following this the data set was edited and updated by Bell and Currie (2016) to investigate the relationships of *Boreonykus certekorum*. Finally, the data set was most recently edited by Currie and Evans (2019) with numerous rescorings and character additions based on the study of new specimens of *Saurornitholestes langstoni*. The current study added both *Dineobellator notohesperis* (SMP VP-2430). The second data set is based mainly off work by Turner et al. (2012) and Brusatte et al. (2014). This data set was further updated by Cau et al. (2015, 2017), who added several characters, rescored *Balaur bondoc*, and added *Halskaraptor escuilliei*. To this data set we also added *Boreonykus certekorum*, *Dakotaraptor steini*, and *Dineobellator notohesperis*, along with further scoring *Acheroraptor temertyorum* for characters added after the Turner et al. (2012) study. Both data sets were analyzed using TNT version 1.5 (Goloboff and Catalano, 2016). In both analyses, we performed 100 replications of the “New Technology” search analysis with default parameters, and further explored the shortest tree islands found performing “Traditional Search” analyses using default parameters and saving all shortest trees found. Nodal support was also calculated in TNT saving all trees up to 10 steps longer than the most parsimonious results.

First data set. The first analysis includes 38 operational taxonomic units and 180 morphological characters focused on the interrelationships of the Dromaeosauridae. *Dineobellator notohesperis*, The analysis resulted in 32 most parsimonious trees, with a tree length of 416 steps, a Consistency Index of 0.466 and a Retention Index of 0.640. As has been the case with other studies investigating the phylogenetic relationships of dromaeosaurids, support throughout the tree was low. The only clades with Bremer support values greater than 1 was *Saurornitholestes langstoni* + *Atriciraptor marshalli* (=2) and the Eudromaeosauria + *Bambiraptor feinbergi* (=2). Similar to the results of Currie and Evans (2019), bootstrap values were also low throughout the tree. None were over 50% and the only ones over 25% within Eudromaeosauria were *Tsaagan mangas* + *Linheraptor exquisitus* (=45%) and *Saurornitholestes langstoni* + *Atrociraptor marshalli* (=29%). Among the Velociraptorinae, a key synapomorphy is a distinctly longer than tall rostral ramus of the maxilla. Additionally, most North American taxa possess a short rostral ramus of the maxilla (at least as tall as long). *Dineobellator* further groups as derived within the Velociraptorinae and sister to *Tsaagan mangas* + *Linheraptor exquisitus* due to the lack of mesial denticles on the maxillary and dentary teeth.

Second data set. The second analysis includes 157 operational taxonomic units and 860 morphological characters focused on the interrelationships of the Coelurosauria. The Theropod Working Group dataset was used, mainly from Turner et al. (2012), Brusatte et al. (2014), and recently updated by Cau et al. (2017). A character state, namely character state 2, was added to character 758 for the opisthocoelous anterior caudal vertebra in

Dineobellator notohesperis. *Gigantoraptor erlianensis*, while not currently scored for this dataset, would also be scored as character state 2 for this character. The analysis resulted in 11,590 most parsimonious trees, with a tree length of 3,317 steps, a Consistency Index of 0.328 and a Retention Index of 0.7612. While many theropod groups had higher resolution, intrafamilial relationships of the Dromaeosauridae were poorly resolved. The majority of dromaeosaurids in the dataset resulted in a unresolved polytomy. The only clade among dromaeosaurids was *Neuquenraptor*+*Unenlagia* and *Austroraptor*. All these dromaeosaurids were part of a larger polytomy with other eumaniraptorans.

XII. Supplemental character list for first data set. Characters from the first data set used in the phylogenetic analysis. 180 total characters were taken from Currie & Evans (2019), which, in turn, were mostly derived from Longrich & Currie (2009), with additional characters from Turner et al (2012), Evans et al. (2013), Bell & Currie (2015), and Kubota (2015). All characters were treated as unordered.

1. Skull length: 0, less than 125% length of femur: 1, at least 125% length of femur

2. Skull height ratio, mid-naris level compared with mid-orbital level: 0, more than half; 1, less than half (233. Senter 2007; 3, Kubota 2015)

3. Antorbital skull length to femur length ratio: 0, less than 60%; 1, more than 60%. (new character).

4. Narial opening, caudal margin; 0, caudal to PM-Max suture on alveolar margin; 1, well caudal (new character). *Bambiraptor*, *Deinonychus*, *Linheraptor*, *Tsaagan*, *Velociraptor* as coded as 0. *Atrociraptor* and *Saurornitholestes* are 1.

5. Narial opening, caudal margin; 0, rostral to rostral border of antorbital fossa; 1, nearly reaching rostral part of antorbital fossa. (19. Maryańska et al. 2002; 23*. Senter 2007; 23*. Turner et al. 2007; 16, Kubota 2015)

6. Antorbital fossa, rostral margin is level with: 0, 3rd maxillary alveolus; 1, 4th alveolus; 2, level with or caudal to the 5th alveolus (new character).

7. Antorbital fossa, ventral margin: 0, extends onto caudal half of the maxilla (jugal ramus) and is visible in lateral view; 1, does not extend onto the jugal process, antorbital fossa restricted ventrally.

8. Antorbital fenestra; 0, longer than tall; 1, subequal or taller than long (new character).

9. Promaxillary fenestra of maxilla: 0, subcircular and broadly exposed in lateral view; 1, slitlike, largely concealed in the rostroventral margin of antorbital fossa.

10. Maxillary fenestra: 0, developed as a simple perforation; 1, positioned in a distinct fossa caudodorsal to the promaxillary fenestra. This character is rescored as a binary character by combining character states 1 and 2, as they form a continuum, following the argumentation of character 239 in Turner et al. (2012).

11. Maxillary fenestra: 0, large and subcircular: 1, small and subcircular; 2 rostrocaudally elongate

12. Maxillary fenestra position: 0, low in antorbital fossa with ventral margin at level of ventral margin of antorbital fossa; 1, low in fossa but above ventral margin; 2, in upper half of antorbital fossa. This character is scored "2" for the Bayanshiree Formation new taxon based on IGB 100/23. The preserved portion of the maxilla in this specimen allows inference of a dorsally positioned accessory antorbital fenestra. *Acheroraptor temertyorum* has an accessory antorbital fenestra positioned very low in the antorbital fossa compared to other dromaeosaurids, and is scored as [0].

13. Maxillary fenestra, ratio of distance between lower edge of fenestra and alveolar margin divided by length of tallest tooth: 0, less than 2.0; 1, greater than 2.0

14. Maxillary fenestra: 0, situated caudal to rostral border of antorbital fossa; 1, situated close to rostral border [from Turner et al. (2012): character 28, reversed polarity]

15. Orbit, margin: 0, smooth; 1, with raised rim. (24. Currie and Varricchio, 2004; 61, Kubota 2015)

16. Supratemporal fossa, extension onto frontal: 0, rostral emargination of fossa straight or slightly curved; 1, sinusoidal and reaching onto the postorbital process, often with a deep pit. (3. Currie 1995; 11. Currie and Varricchio, 2004; 43. Senter 2007; 42. Turner et al. 2007; 63, Kubota 2015)

17. Supratemporal fossa on frontal: 0, restricted to the lateral half of the frontal; 1, supratemporal fossa extends medially.

18. Quadratic foramen/fenestra between quadrate and quadratojugal: 0, small foramen; 1, large fenestra.

19. External mandibular fenestra, size; 0, small and slit-like; 1, large and rounded. (22. Currie 1995; 74. Senter 2007; 73. Turner et al. 2007; 119, Kubota 2015)

20. Premaxilla shape: 0, elongate; 1, body of premaxilla short, no more than 15% length of maxilla

21. Premaxilla, maxillary process: 0, short; 1, elongate process separating nasal and maxilla

22. Premaxilla, main body below naris: 0, longer than tall; 1, at least as tall as long. (5. Holtz, 1998; 4*. Maryańska et al. 2002; 30. Currie and Varricchio, 2004; 237*. Senter 2007; 10, Kubota 2015)

23. Premaxilla, nasal process: 0, projects caudodorsally; 1, projects caudally.

24. Premaxilla, narial fossa: 0, limited exposure on lateral surface; 1, prominent rostroventral extension of narial fossa onto lateral surface of premaxilla

25. Maxilla, contribution to border of naris: 0, excluded from border; 1, narial fossa extends onto rostral ramus of maxilla

26. Maxilla, postantral wall: 0, concealed in lateral view; 1, projects caudally into antorbital fenestra and exposed in lateral view

27. Maxilla, palatal shelf: 0, concealed in lateral view; 1, palatal shelf projects dorsally into the antorbital fenestra and visible in lateral view.

28. Maxilla, rostral ramus: 0, elongate, 25% or more of the length of maxilla; 1, short, less than 25% length. Character scored for Baynshire new taxon based on MPC-D100/23.

29. Maxilla, rostral ramus: 0, longer than tall; 1, short, at least as tall as long.

30. Maxilla, lateral surface: 0, smooth; 1, maxilla with a distinct lip (ridge) bounding the ventral margin of the antorbital fossa. Character definition modified to point out that the ridge at the margin of the fossa protrudes from the lateral surface of the maxilla.

31. Maxilla, interfenestral bar between maxillary and antorbital fenestrae: 0, narrow; 1, rostrocaudally broad.

32. Maxilla, jugal process ventral to the antorbital fossa: 0, dorsoventrally low; 1, dorsoventrally tall. [from Turner et al. (2012): character 238]

33. Nasal, dorsal outline in lateral view: 0, concave; 1, straight to convex. *Bambiraptor* scored as 0 on basis of holotype.

34. Nasal participation in margin of antorbital fossa: and has pneumatopores: 0, no; 1, yes, and has pneumatopores. (new character).

35. Lacrimal shape: 0, T-shaped with subequal rostrodorsal and caudodorsal processes; 1, T-shaped with rostrodorsal process much longer than caudodorsal process. (7*. Currie, 1995; 32*. Holtz, 1998 ; 95*. Sereno, 1999; 18*. Currie and Varricchio, 2004; 40*. Senter 2007; 39*. Turner et al. 2007; 36, Kubota 2015)

36. Prefrontal; 0, present as separate bone; 1, lost as a separate bone. (new character).

37. Frontonasal suture: 0, transverse orientation; 1, frontal narrows rostrally into a wedge between nasals; 2, complex W-shaped suture. (39*. Holtz, 1998; 25*. Maryańska et al. 2002; 12*. Currie and Varricchio, 2004; 42*. Senter 2007; 41*. Turner et al. 2007; 59, Kubota 2015)

38. Frontal, orbital margin in dorsal view: 0, straight or smoothly concave; 1, postorbital process sharply offset, and orbital margin L-shaped in dorsal view

39. Frontal notched to receive lacrimal: 0, absent; 1, present. Frontal edge smooth in region of lacrimal suture; 1, notched (1). (8. Currie, 1995; 13. Currie and Varricchio, 2004; 45. Senter 2007; 44. Turner et al. 2007; 60, Kubota 2015)

40. Parietal fusion: 0, unfused. 1, fused. *Bambiraptor* is 0 (probably because of immaturity).

41. Jugal pneumatic: 0, no; 1, yes

42. Jugal, suborbital process. 0, dorsal and ventral borders subparallel; 1, distinctly deeper at the back of the orbit than front, triangular. (new character).

43. Jugal, suborbital process: 0, dorsoventrally slender; 1, dorsoventrally deep and robust.

44. Jugal, postorbital process: 0, slender triradiate jugal; 1, postorbital process broad so that jugal is triangular.

45. Postorbital in lateral view: 0, has a straight rostral (frontal) process; 1, frontal process curves rostrodorsally and dorsal border of temporal bar is dorsally concave. (10. Currie, 1995; 16. Senter et al. 2004; 4. Senter 2007; 4. Turner et al. 2007; 69, Kubota 2015)

46. Postorbital, contribution to lateral temporal fenestra: 0, contributes to the dorsolateral margin in lateral view; 1, does not contribute to lateral temporal fenestra, excluded by jugal-squamosal contact.

47. Squamosal, tab-like process invades caudolateral corner of upper temporal fenestra: 0, no; 1, yes.

48. Quadratojugal: 0, without horizontal process caudal to ascending process (reversed L-shape); 1, with process (inverted T- or Y-shape). (11. Currie, 1995; 63. Holtz, 1998; 33. Currie and Varricchio, 2004; 36. Senter 2007; 35. Turner et al. 2007; 56, Kubota 2015)

49. Quadratojugal, ascending (squamosal) ramus: 0, straight ascending ramus; 1, ascending ramus bowed rostrally.

50. Quadrate shaft pierced by large, pneumatic foramen: 0, present; 1, absent.

51. Quadrate shaft in lateral view: 0, straight or weakly curved; 1, strongly bowed rostrally.

52. Occipital condyle: 0, lacks constricted neck; 1, subspherical with constricted neck. (21. Makovicky and Sues, 1998; 103. Holtz, 1998; 56. Senter 2007; 55. Turner et al. 2007)

53. Exoccipital, caudal surface with a bowl-like depression containing the exits of cranial nerves X and XII: 0, absent; 1, present. (19. Senter 2007; 19. Turner et al. 2007)

54. Exoccipital-opisthotic, paroccipital processes: 0, projects ventrolaterally; 1, projects laterally.

55. Exoccipital-opisthotic, paroccipital process: 0, elongate and slender, with dorsal and ventral edges nearly parallel; 1) short, deep with convex distal end. (57. Senter 2007; 56. Turner et al. 2007)

56. Exoccipital-opisthotic, paroccipital processes, occipital surface of distal end: 0, oriented more caudally than dorsally; 1, conspicuous twist in the distal end orients distal surface more dorsally than proximal region. (14. Currie 1995; 9. Currie and Varricchio, 2004; 59. Senter 2007; 58. Turner et al. 2007; 94, Kubota 2015)

57. Paroccipital process plus squamosal: 0, does not extend beyond level of intertemporal bar of postorbital and squamosal; 1, ventrolateral process of squamosal and lateral extension of paroccipital process beyond head of quadrate. (13, Currie 1995)

58. Basioccipital tubera, caudal surfaces: 0, flat or smoothly concave; 1, with distinct, ovoid depressions

59. Basioccipital tubera: 0, separated by weak notch; 1, separated by a deep, broad, U-shaped ventral notch.

60. Basisphenoid recess with paired openings: 0, absent; 1, present

61. Lateral depression of braincase bounded by otosphenoidal crest prominent: 0, absent; 1, present.

62. Parasphenoid recess (parabasisphenoidal recess): 0, caudal to cultriform process; 1, adjacent to base of cultriform process. (81, Kubota 2015)

63. Accessory tympanic recess dorsal to crista interfenestralis: 0, absent; 1, small pocket present; 2, extensive with indirect pneumatization. (17. Senter 2007; 17. Turner et al. 2007)

64. Foramen magnum: 0, subcircular; 1, distinctly taller than wide.

65. Ectopterygoid, dorsal recess: 0, absent; 1, present. (61. Senter 2007; 60. Turner et al. 2007)

66. Dentary symphysis, ventral deflection with lateral parapet centered on third tooth: 0, present; 1, absent.

67. Dentary, shape of dorsal symphysial region rostral to the third dentary tooth: 0, dorsal margin at same level and continuous with alveolar ramus; 1, alveolar margin concave.

68. Dentary, shape of symphysis, ventral margin: 0, at same level and continuous with ventral ramus of the dentary; 1, symphyseal region extends ventrally below the level of the dentary margin forming a symphyseal ‘chin’.

69. Dentary curvature in lateral view: 0, straight or weakly curved; 1, strongly bowed, with curved dorsal and ventral margins. *Deinonychus* and *Shanag* are considered to have relatively straight dentaries, and are scored as “0”.

70. Dentary curvature in dorsal view: 0, straight; 1, curves medially towards symphysis. (107, Holtz, 1998; 190, Sereno 1999; 66, Senter 2007b; 65, Turner et al. 2007)

71. Dentary shape: 0, subtriangular, with dorsal and ventral margins diverging caudally; 1, dorsal and ventral margins subparallel. *Velociraptor mongoliensis* is considered to have subparallel dentary margins in lateral view.

72. Dentary with distinct midlength constriction and terminal expansion: 0, absent; 1, present.

73. Dentary, nutrient foramina: 0, inset into prominent groove along length of the lateral surface; 1, lateral groove reduced rostrally or absent. (72. Senter 2007; 71. Turner et al. 2007; 115, Kubota 2015).

74. Splenial: 0, limited or no exposure on lateral surface of mandible; 1, conspicuous triangular exposure between dentary and angular. (21. Currie, 1995; Holtz, 1998; 36. Currie and Varricchio, 2004; 76. Senter 2007; 75. Turner et al. 2007; 133, Kubota 2015)

75. Articular with tall, columnar process on retroarticular process: 0, absent; 1, present.

76. Interdental plates on premaxilla, maxilla, and dentary: 0, distinct and separate; 1, fused to each other and jaw margin; 2, no interdental plates (labial wall of each tooth socket is significantly higher than the lingual one). This character is modified from Longrich and Currie (2009) with the addition of one character state [2] that distinguishes troodontids, which lack interdental plates, from dromaeosaurids, in which they are fused to the dentigerous bones. Interdental bone can also be distinguished histologically from the dentary, and by the presence of foramina between the bases of interdental plates. Separation and fusion are distinguished from absence by having nearly equal heights of the labial and lingual walls of the tooth sockets. (Currie, 1987b. 6, Currie, 1995; 135. Holtz, 1998; 15. Currie and Varricchio, 2004; 90*. Senter 2007; 90*. Turner et al. 2007; 122, Kubota 2015).

77. Teeth, premaxillary teeth; 0, 2–4 subequal in size; 1, second premaxillary tooth larger than third and fourth.

78. Teeth, cross-section at base of premaxillary tooth #2: 0, J-shaped in cross-section. 1) flattened lingual surface with longitudinal striations. (new character).

79. Teeth, premaxillary and rostral dentary: 0, lack serrations; 1, have denticles on distal carina.

80. Teeth: 0, constricted between root and crown: 1, root and crown confluent. (130*. Holtz, 1998; 21*. Senter et al. 2004; 88. Senter 2007; 88. Turner et al. 2007; 140, Kubota 2015) Only caudal teeth of *Microraptor zhaoianus* are constricted between root and crown, which is scored as "0" by Kubota and scored as multistate here.

81. Teeth, maxillary tooth counts: 0, fewer than 20; 1, numbering at least 20

82. Teeth, maxillary teeth: 0, subequal in length along the jaw; 1, caudal maxillary teeth elongate and fanglike, approximately 200% the length of most rostral maxillary teeth.

83. Teeth, maxillary: 0, more or less perpendicular to jaw margin; 1, strongly raked caudally.

84. Teeth, roots of maxillary and dentary teeth: 0, mediolaterally compressed; 1, circular in cross-section. (228. Senter 2007; 150, Kubota 2015)

85. Teeth, denticles: 0, large; 1, small; 2, absent. Farlow et al. (1991) quantify this difference. (128*. Holtz, 1998; 86*. Senter 2007; 86*. Turner et al. 2007; 141, Kubota 2015).

86. Teeth, mesial carina on maxillary and dentary teeth is close to midline of tooth near tip but twists toward the lingual surface: 0, absent; 1, present. (2. Currie, 1995; 155, Kubota 2015)

87. Teeth, denticle shape: 0, convex; 1, pointed at tip and hooked apically; 2, absent. (23*. Senter et al. 2004; 87*. Senter 2007; 87*. Turner et al. 2007; 142, Kubota 2015)

88. Teeth, denticle orientation: 0, perpendicular to carina. 1, orientated toward the tip of the crown; 2, absent. (143, Kubota 2015).

89. Teeth, maxillary and dentary: 0, teeth large; 1, more than 25 small teeth. (85*. Senter 2007; 84*. Turner et al. 2007; 151, Kubota 2015)

90. Teeth, maxillary and dentary: 0, subequal in number and size; 1, dentary teeth more numerous and smaller than maxillary teeth. (127. Holtz, 1998; 157, Kubota 2015)

91. Teeth, maxillary and caudal dentary teeth: 0, not serrated; 1, denticles on distal carina only; 2, denticles on mesial and distal carinae.

92. Teeth, relative denticle size of mesial and distal carinae on maxillary and dentary teeth: 0, subequal: 1, distal denticles much larger than mesial ones: 2, no denticles; 3, mesial denticles absent. (5*. Currie, 1995; 129. Holtz, 1998; 38*. Currie and Varricchio, 2004; 20*. Senter et al. 2004; 247*. Turner et al. 2007; 258*. Senter 2007; 156, Kubota 2015).

93. Teeth, dentary: 0, in separate alveoli; 1, set in open groove. (260. Senter 2007; 85. Turner et al. 2007; 160, Kubota 2015)

94. Teeth, dentary: 0, evenly spaced; 1, mesial dentary teeth smaller, more numerous, and more closely appressed than those in middle of tooth row. (89. Senter 2007; 89. Turner et al. 2007; 159, Kubota 2015)

95. Vertebrae, epipophyses on axis: 0, short; 1, elongate, project laterally beyond postzygapophyses.

96. Vertebrae, rostral cervical centra: 0, level with or shorter than caudal extent of neural arch; 1, extends beyond caudal extent of neural arch. (33. Makovicky and Sues, 1998; 158. Holtz, 1998; 102. Maryańska et al. 2002; 96. Senter 2007; 96. Turner et al. 2007; 167, Kubota 2015)

97. Vertebrae, carotid process on rostroventral margin of caudal cervical vertebrae: 0, absent; 1, present. (40. Makovicky and Sues, 1998; 162. Holtz, 1998; 97. Senter 2007; 97. Turner et al. 2007; 169, Kubota 2015)

98. Vertebrae, cervical: 0, with low neural spines; 1, cervical neural spines at least as tall as long rostrocaudally.

99. Vertebrae, cervical prezygapophysis:0, unflexed; 1, flexed. (263. Senter 2007; 171, Kubota 2015)

100. Vertebrae, transverse processes of rostral dorsal vertebrae: 0, long and thin; 1, short, wide, and only slightly inclined. (107. Senter 2007; 107. Turner et al. 2007; 185, Kubota 2015)

101. Vertebrae, dorsal: 0, parapophyses short; 1, parapophyses on elongate pedicels.

102. Vertebrae, dorsal: 0, no pneumatopores; 1, pneumatopores present on dorsal centra.

103. Vertebrae, dorsal, neural spine height: 0, low, height does not exceed rostrocaudal length; 1, taller than long rostrocaudally (≥ x 1.5 rostrocaudal length).

104. Vertebrae, dorsal; 0, neural arch does not have prominent rostral fossae on either side of neural canal; 1, present.

105. Vertebrae, caudal dorsals: 0, centra elongate; 1, short and massive, length of centrum less than diameter.

106. Vertebrae, caudal dorsals; 0, no expansion of neural spines distally; 1, distal end of neural spines transversely expanded by at least 200% to form a distinct spine table (47. Makovicky and Sues, 1998; 168. Holtz, 1998; 108. Senter 2007; 108. Turner et al. 2007; 182, Kubota, 2015). Recoded as ? in *Bambiraptor* because of its juvenile (and broken) condition.

107. Vertebrae, sacral; 0, five vertebrae incorporated into sacrum; 1, sacrum incorporating at least 6 vertebrae. (185*. Holtz, 1998; 39*. Sereno, 1999; 109*. Maryańska et al. 2002; 110. Senter 2007; 110*. Turner et al. 2007; 186, Kubota 2015)

108. Vertebrae, sacral: 0, lack pneumatopores; 1, pneumatopores present in one or more sacral vertebrae. Velociraptor is recoded as '1' on basis of pneumatopore in 100/986.

109. Vertebrae, caudal, distal caudal centra: 0, have prominent lateral depressions; 1, lateral surfaces of centra flat or convex.

110. Vertebrae, caudal, distal: 0, greatly elongated, more than 200% the length of proximal caudals; 1, moderately elongate, no more than 200% the length of the proximal caudals. *Bambiraptor* recoded as '1'

111. Vertebrae, caudal, distal: 0, with a convex or flat dorsal surface; 1, with a prominent dorsal groove (119. Senter 2007; 119. Turner et al. 2007; 198, Kubota 2015)

112. Vertebrae, caudal: 0, prezygapophyses short; 1, elongate; 2, extended by ossified tendons of caudal epaxial muscles.

113. Cervical ribs, shafts: 0, slender and longer than vertebrae to which they articulate; 1, broad and shorter than vertebra. (51*. Sereno, 1999; 106. Maryańska et al. 2002; 124. Senter 2007; 124. Turner et al. 2007; 200, Kubota, 2015)

114. Sternal plates: 0, unossified; 1, ossified.

115. Furcula: 0, interclavicular angle less than 90°; 1, at least 90°.

116. Scapula, acromion margin: 0, continuous with blade; 1, rostral edge laterally everted. (133. Senter 2007; 133. Turner et al. 2007; 208, Kubota 2015)

117. Coracoid: 0, highly flexed in lateral view, with dorsal and ventral rami of coracoid forming an angle of 90°–100°; 1, coracoids weakly flexed, forming an angle larger than 100°.

118. Coracoid: 0, elongate, taller than wide; 1, short, at least as wide as tall.

119. Humerus length: 0, elongate and at least 75% length of femur; 1, humerus less than 75% length of the femur.

120. Humerus, internal tuberosity: 0, proximodistally short; 1, proximodistally elongate, about 50% the length of deltopectoral crest.

121. Humerus, proximal shaft with prominent longitudinal ridge on caudal surface: 0, absent; 1, present.

122. Radius shaft diameter: 0, greater than; 1, less than or subequal to 0.5× ulna in diameter. (278. Senter 2007; 241, Kubota 2015)

123. Ulna, length compared to scapular length: 0, shorter; 1, longer. (242, Kubota 2015).

124. Ulnar/femoral length ratio: 0, significantly less than one; 1, equal or greater than one. (236. Turner et al. 2007; 243, Kubota 2015)

125. Metacarpal I length compared with Mtc II length: 0, no more than 33%; 1, more than a third. *Bambiraptor* recoded as '1'.

126. Metacarpal II, length compared with Mc I plus manual phalanx 1-1: 0, shorter; 1, subequal to or longer. (54*. Senter et al. 2004; 285. Senter 2007; 257, Kubota 2015)

127. Manual phalanx I-1: 0, strongly bowed in medial view; 1, weakly curved or straight.

128. Manual phalanx I-2 (ungual): 0, proximodorsal 'lip' absent; 1, present. (153. Senter 2007; 279, Kubota 2015). Rescored in *Saurornitholestes* and *Velociraptor* as '0' from Kubota. *Deinonychus* changed to 0

129. Manual phalanx II-1: 0, caudal flange absent; 1, present. (62. Senter et al. 2004; 290. Senter 2007; 283, Kubota 2015)

130. Manual phalanx III-1 length compared to length of manual phalanx III-2: 0, less than twice the length; 1, more than double the length. (57. Senter et al. 2004; 294. Senter 2007; 292, Kubota 2015)

131. Manual phalanx III-1: 0, short; 1, elongate, at least 75% length of III-3.

132. Ilium, rostral wing: 0, 200% length of caudal wing; 1, short, less than twice length of caudal blade.

133. Ilium, rostral end of rostral wing: 0, rounded or straight; 1, with notched rostral margin. *Bambiraptor* recoded as '?' because rostral margin broken. (156*. Senter 2007; 156*. Turner et al. 2007; 299, Kubota 2015)

134. Ilium, cuppedicus ridge ends on pubic peduncle; 1, extends caudally to acetabulum.

135. Ilium, medial antiliac shelf: 0, short; 1, elongate, approaching length of caudal wing.

136. Ilium, caudal wing: 0, slender, shallower than rostral wing; 1, caudal wing as deep or deeper than rostral wing. Recoded as 0 in Baynshire nov. sp (MPC-D100/23).

137. Ilium, caudal wing: 0, longer than tall; 1, at least as tall as long. Recoded as 0 in Baynshire nov. sp (MPC-D100/23).

138. Ilium: 0, has acuminate caudal margin; 1, brevis shelf lobate and projects caudally beyond postacetabular lamina; 2, brevis shelf notched in lateral view. Recoded as 0 in Baynshire nov. sp (MPC-D100/23).

139. Ilium, caudal wing: 0, with straight or convex dorsal margin; 1, concave dorsal margin. Recoded as 0 in Baynshire nov. sp (MPC-D100/23) by Kubota 2015. Dorsal margin of ilium along central portion of blade nearly straight; 1, arched (1). (151. Maryańska et al. 2002; 304, Kubota 2015)

140. Ilium, pubic peduncle in lateral view: 0, rostrocaudally narrow; 1, broad rostrocaudal length, approximately 200% of height.

141. Pubis, distal end: 0, with prominent caudal expansion; 1, distal end spatulate, both rostral and caudal expansions absent; 2, with prominent rostral and caudal expansions.

142. Pubis, shaft: 0, straight or gently curved; 1, distal end strongly bent caudally.

143. Pubis, lateral surface of shaft: 0, smooth or bearing a ridge; 1, with enlarged tubercle or process.

144. Pubis, pubic apron: 0, extends less than 50% length of pubis; 1, extends at least 50% length of pubis.

145. Pubis orientation: 0, propubic; 1, vertical; 2, caudally oriented (opisthopubic). (309*, Holtz, 1998; Maryańska et al. 2002 ; 175*. Senter 2007; 177. Turner et al. 2007; 317, Kubota 2015)

146. Ischium length: 0, no more than 50% length of pubis; 1, elongate, more than 50% length of pubis. Although there is no pubis in MPC-D100/22, the comparison of ischium length with other limb bones shows that it is elongate and is therefore coded as 1.

147. Ischium, proximodorsal process: 0, present; 1, absent; 2, hypertrophied, hooked and separated from iliac peduncle of ischium by a notch. (325*. Holtz, 1998; 192*. Rauhut, 2003; 72. Senter et al. 2004; 230*. Turner et al. 2007; 332, Kubota 2015)

148. Ischium, lateral ridge on shaft: 0, absent; 1, present. Recoded as 1 for Baynshire nov. sp (MPC-D100/23) by Kubota 2015.

149. Caudal edge of ischium: 0, straight; 1, with median or distal caudal process, except for proximodorsal process. (334*. Senter 2007; 165 and 232*. Turner et al. 2007)

150. Ischium, distal dorsal process: 0, prominent; 1, highly reduced or absent. Recoded as 1 for Baynshire nov. sp. (MPC-D200/23) by Kubota 2015. (334*. Senter 2007; 165 and 232*. Turner et al. 2007; 333, Kubota 2015)

151. Ischium, obturator process position: 0, at distal end of shaft; 1, at midshaft; 2, proximal in position (323*. Holtz, 1998; 170*. Maryańska et al. 2002; 169. Turner et al. 2007; 336, Kubota 2015)

152. Ischium, obturator process: 0, elongate and spur-like; 1, broad and flange-like. (70. Senter et al. 2004; 305*. Senter 2007; 234*. Turner et al. 2007; 337, Kubota 2015)

153. Ischium, obturator process: 0, separated from ischial shaft by caudal notch; 1, confluent. Recoded as 1 for Baynshire nov. sp. (MPC-D200/23) by Kubota 2015.

154. Ischium, distal end: 0, tapers to a narrow point; 1, broadly expanded, blunt or spatulate.

155. Ischium, shaft: 0, mediolaterally compressed; 1, subcircular in section. Recoded as 0 for Baynshire nov. sp. (MPC-D200/23) by Kubota 2015.

156. Ischium, ridge on medial surface connecting proximodorsal process and iliac peduncle: 0, absent; 1, present.

157. Femur, angle of femoral head: 0, projecting dorsomedially or medially relative to shaft; 1, projects ventromedially.

158. Metatarsal II length compared to that of Mt IV: 0, subequal; 1, markedly shorter.

159. Metatarsal II tuber along rostral surface: 0, absent. 1, present. (Chiappe, 2002; 235. Turner et al. 2007; 371, Kubota 2015)

160. Metatarsal II, proximal end size compared to proximal end of Mt III: 0, as deep as and deeper than; 1, shallower; 2, no exposure of metatarsal III. (370, Kubota 2015)

161. Metatarsal II, distal end: 0, smooth; 1, with well-developed ginglymus. (86. Senter et al. 2004; 198. Senter 2007; 201. Turner et al. 2007; 373, Kubota 2015)

162. Metatarsal II, width of distal condyles: 0, broad, subequal to Mt III; 1, more narrow than Mt III.

163. Metatarsal II, distal end compared with width of distal end of Mt IV: 0, wider than or as wide as; 1, narrower. (372, Kubota 2015)

164. Metatarsal III, plantar surface: 0, broadly exposed; 1, largely covered by Metatarsals III and IV. Recoded as 0 for Baynshire nov. sp. (MPC-D200/23) by Kubota 2015.

165. Metatarsal III, rostrodorsal surface of shaft: 0, flat or rounded; 1, has a prominent longitudinal sulcus. Recoded as 0 for Baynshire nov. sp. (MPC-D200/23) by Kubota 2015.

166. Metatarsal IV, lateral flange on proximal end of shaft: 0, absent; 1, present.

167. Metatarsal IV, prominent flange on caudolateral surface of shaft: 0, present; 1, highly reduced or absent.

168. Metatarsal IV, ventral surface proximal to distal articular surface: 0, no tuber; 1, has a prominent tuber.

169. Metatarsal IV, distal condylar surface: 0, trochlear to planar; 1, strongly ball-shaped.

170. Foot symmetry: 0, symmetrical; 1, asymmetrical with slender metatarsal II and robust metatarsal IV, excluding flange. (205. Senter 2007; 208. Turner et al. 2007; 363, Kubota 2015)

171. Pedal phalanx II-1 length: 0, elongate; 1, short and robust, shaft length does not exceed 200% the diameter of the distal condyle. (96. Senter et al. 2004; Senter 2007; 394, Kubota 2015)

172. Pedal phalanx II-2 length: 0, subequal to or longer than II-1; 1, significantly shorter than II-1. Recoded for Deinonychus and Dromaeosaurus as '0'.

173. Pedal phalanx II-2 shaft: 0, slender to moderately robust; 1, massive, shaft diameter at least 50% of shaft length.

174. Pedal phalanx II-2 proximoventral flexor heel: 0, short; 1, elongate caudal projection. (228. Turner et al. 2007; 387, Kubota 2015)

175. Pedal phalanx II-2, ventromedial keel ventral to flexor heel: 0, absent; 1, present (388, Kubota 2015).

176. Pedal phalanx II-2, collateral ligament pits: 0, deep; 1, shallow.

177. Pedal phalanx II-3, lateral and medial vascular grooves: 0, at same level; 1, lateral groove more dorsal and medial groove more ventral.

178. Pedal phalanx IV-4: 0, shorter than or subequal to pedal phalanx IV-3; longer than pedal phalanx IV-3. (97. Senter et al. 2004; 395, Kubota 2015)

179. Vaned feathers on forelimb: 0, symmetric; 1, asymmetric. (1. Senter et al. 2007b; 1. Turner et al. 2007; 1, Kubota 2015).

180. Vaned feathers on tarsus: 0, present; 1, absent.

XIII. Character-taxon matrix for first data set. Character-taxon matrix based on the first data set, including 180 total characters and 38 taxa, based mainly off the dataset of Currie & Evans (2019), which was previously based on studies by Bell & Currie (2016), Kubota (2015), Evans et al. (2013), and Longrich & Currie (2009).

Acheroraptor_temertyorum
?????20?111100??????????01?00001?????????????????????????????????1111?101??1???10000?0010021
00??

Achillobator_giganticus
?????00??????0??????1???0?001001???1???1000?1?0?0?20??
??0100111?1???1?01???0???????????????110011200200111000121111100???10??0???????1?11????

Adasaurus_mongoliensis
??????????????001????????????????????1?1?11110?1?01??100??1????????????????????????????????0??
10011?1???0?11?1?10???01?????????????110010000200121 1??1111001?11110100110111 1?011?0??

Atrociraptor_marshalli
???11111011211?????0110000101001??????????????????????????????00000101??11?1100101011002
100???

Austroraptor_cabazai
1???0200002000??1???0???010000100?0?11????00?0????????????????????0??1?110?????0110012?22100
200?11???11??01?????????????11??1?00????

Bambiraptor_feinbergi
0??011110112001 11??011000001 10000?010?00?0001011111 00????010?1???0?1101011?111?100101011
?011001001?011111?011102?1101101001010100111?000000000012001 1101100101?0100000001010011
011??

Baynshire_nov._sp.
????11?????2?0?????011?10?011001??????????1??????????????????????00??101011?11?11000011010020
00??????111110??1102??????0100??1010000????0000??????1111111001?1?0100001101 0000?0100??

Boreonykus_certekorum
???????????????01????????????????????211???1??0???????20????
????????????1?0??1????

Buitreraptor_gonzalezorum
1???0????20?001?1??????10??00000??0001??0010???000?????????????0??00110??0??01??002221002
00011??1100?00?00?011?010?011010????????????011??1??1?010110000?100?1101?00??0?????????

Byronosaurus_jaffei
????1200?02000?????100111???00100?1?1???????????????0?10?00?1?11?001110001?2?0001100202211
0211???01?00??????0?1??0??????

Changyuraptor_yangi
0?????????2?????????0???
??????????????2??????0??????1???????????????11??????????????1????????????????????0

Dakotaraptor_steini
??1?00??21????
?????1??????1??2????????00?0????????????????????????????????010?0????0??0???????0???

Deinonychus_antirrhopus
???00210011200??1?10110000001001110??1?1??1010110??01?01????????10000010111101100101001
0021001001101110110?11021??111110000101000011000000002001?011011111000110100000101001001
1010??

Dineobellator_notohesperis

????????????????????????????0??1?????????1????????1?????01????0???????????????1??0?10000?130??
?????????????110???????010??0??????????????????????????????????0?10?0?00???????????1???

Dromaeosaurus_albertensis
???010000???0000111?11??0?0110?1????2111?010???10101010 1?000000000000010111100110000 1101
002000??10???1?11?101?11????

Graciliraptor_lujiatensis
???????????????????????????????????1???01???01???0?21????
????????0???0?02?????????0?001?1111???????????????????????????1??1101?????0000?00????

Halzskaraptor_escuilliei
0101?2????1210100??00010?0?00?000?01?0110000100?????????????????0000010????0?0110002?2210
020?00?00?100???1????011???????0??111??011?0?0000??????????????????00?110?0???000101?0?0??

Hesperonychus_elizabethae
??
????????????????????????????????????0???0001110????????????????????????????????????

Hulsanpes_perlei
???
??00?1??00?000?0?????????

Linheraptor_exquisitus
00?00210011101?11??010110100001100011111?1111111001?10???0110??1?1111010111?1?110000100
000130010?1????1??????1?2?1????1??????????????????????00?2??????????????????????????0?????????

Mahakala_omnogovae
??????????????100??????????????????000???????????0?0001????0?00???????????????1???02?22??02??
0???????????10?100???????????0?????????0??0000????????????????0?0?10?00??000??01001???

Microraptor_zhaoianus
01?0100?0????0????001000???1?1?0??00?01??????0?1?????????????????000101011??100[01]0100100?0
?1300?01010100?000?00?211[01]1??00011001011111?0?000?01110200010000000?01001111?00?11000?
00?110

Neuquenraptor_argentinus
???
??0??1??11000??010?00????

Rahonavis_ostromi
???
???110000110001??????0??011???????0011000110000010201?00??00011011001000100 10?001???

Saurornitholestes_langstoni
00010101011210011110110000101001010101111010101111????1????????1001101011111111 0000101
100210010011011111101110211 1?1101000010101011100000000200120010101100101001000000010100
11010??

Shanag_ashile
??????11?112?0??????1???00010100??????????????????????????????????0?000001?????01?100100?0111
01???

Sinornithosaurus_millenii
0??000?101111??11??010??0??1?1?00?000011????10?10?????????????1???0001010111111?010100100100
2100???????0??000????2?1??0001?1100101111111?0?00??11102000100000 0??00?1?11?00?10?00?00?1?
?

Sinovenator_changii
?1?0120000200010 0??1000010000000??1?100???00?????1000010?00?0111?00110000???0?00100?1?00?
013?1?1??11000?0000????????11?????0???0??????0??0?0?0012000100 1??00010100?1010001??0?000??
?

Tianyuraptor_ostromi
00???0?10?1200???????????0?01000?????0?1?????????????????????????????0?????????0???01?0?00??00
?0?????????00???01?1?0?011010000?1?1?0???00?01000?101???111000?0??????0????0???????1??

Tsaagan_mangas
00?00210011101?11??0101101000011000111 11?1111111001?10???0110??1?1111010111?1?110000100
000130010?1??

Stenonychosaurus_inequalis

?????2001022??100???0???10000010??1?1[01]01????1????0110010?0001001?00101000??200101000001
1112111?110110010?1101?111??????000??????????????????2000??1001111000010?0011000001011010
01??

Unenlagia_comahuensis
??
???1111001????????0??111??????????0011011110101 10200?11??001?0??????????????????0??

Unenlagia_paynemili
??
???????0??????????????11???????????????111?2101??????????????????????????0?????????

Utahraptor_ostrommaysi
????????????????????100??1011????1?010??0???
00???11101???1?02????11???????????????00?0200??????00?1211?100???10?00111?01?????1???

Velociraptor_mongoliensis
00100200011100011110101101000010000101111110101110001101?1110100111110101 1?11111000010
010021001001101[01]1?1111110201111111000010100001100100002001211101111100011110100 11110
10011010??

Velociraptor_osmolskae
?????21?011101??????????0?000010???1???1000010??0?110
??

Zhenyuanlong_suni
000000?10?12001????0100?00?01?001?01???1???????1?????????????????0?0?0???1?????????0????00??
00????????????1???????????1???00?0???0???????????10?2?????????????0????????????????????11

XIV. Supplemental character list for second data set. Characters from the second data set used in the phylogenetic analysis are part of the Theropod Working Group data set. 860 total characters were taken from Cau et al. (2017), which, in turn, were mostly derived from Brusatte et al. (2014) and Turner et al. (2012), with additional characters from Cau et al. (2015). Characters 1–853 are mainly from Brusatte et al. (2014), with characters 854–860 from Cau et al. (2015). Characters were left unaltered from those of Cau et al. (2015, 2017), with the alterations they had done to the Brusatte et al. (2014) data set. The only change other than the addition of *Dineobellator notohesperis*, *Dakotaraptor steini*, *Boreonykus certekorum*, and *Acheroraptor temertyorum* was the addition of character state 2 (opisthocoelous anterior caudal vertebrae) for character 758. For further notes on the characters and character states, including changes from the original and earlier versions of the Theropod Working Group data set, see Brusatte et al. (2014).

Character 1: Feathers, vaned feathers on forelimb, form:

0: symmetric

1: asymmetric

Character 2: Orbit, shape

0: circular in lateral or dorsolateral view

1: dorsoventrally elongate

Note (via Brusatte et al., 2014): A "dorsoventrally elongate" orbit was not explicitly defined by Turner et al. (2012) or previous versions of the TWiG dataset, but is here held to be any orbit that is greater than 1.5 times deeper dorsoventrally than long anterposteriorly at its midpoint. This character state is only

present in some adult tyrannosauroid specimens among coelurosaurs. The presence of a circular orbit in *Alioramus* may be a consequence of the juvenile status of the one quality specimen of this taxon (IGM 100/1844), or alternatively, it could be a product of the dorsoventrally shallow and anteroposteriorly elongated snout of this taxon.

Character 3: Postorbital, ventral ramus, projection into orbit: (ORDERED)

0: does not project into orbit, suborbital process absent

1: projects into orbit, suborbital process present and large in adults and small and unpronounced in sub-adults

2: projects into orbit, suborbital process present and large in sub-adults and adults

Note (via Brusatte et al., 2014): Turner et al. (2012) and previous TWiG datasets utilized a binary character relating to the presence/absence of an anterior process projecting into the orbit. We here modify this character by splitting the "presence" state into two states, relating to the varying degree of postorbital projection in tyrannosauroids (to distinguish the hypertrophied condition in *Tyrannosaurus* and *Tarbosaurus* in which the postorbital projects strongly into the orbit in both sub-adults and adults). We have also reversed the 0 and 1/2 designation relative to Turner et al. (2012) so that "0" now refers to the lack of postorbital projection and "1" and "2" refer to the two conditions of postorbital projection, following Brusatte et al. (2010a). We have changed the score of *Haplocheirus* from present to absent based on Choiniere et al. (2010a).

Character 4: Postorbital, shape of anterior (frontal) process in lateral view:

0: straight

1: curving anterodorsally, such that dorsal border of temporal bar is dorsally concave

Character 5: Postorbital, ventral ramus, orientation: (UNORDERED)

0: parallels quadrate, lower temporal fenestra rectangular in shape

1: oriented strongly obliquely relative to quadrate, jugal and postorbital approach or contact quadratojugal to constrict lower temporal fenestra

2: oriented anteroventrally relative to the long axes of the quadrate and lacrimal, angle of postorbital ventral ramus long axis with the lacrimal long axis (if lacrimal is approximately vertical) greater than 30 degrees

Note (via Brusatte et al., 2014): This is a modified version of the original TWiG character, which adds a third state denoting the anteroventrally oriented postorbital ventral rami of some basal coelurosaurs (e.g., tyrannosauroids).

Character 6: Braincase, otosphenoidal crest, form and position:

0: vertical on basisphenoid and prootic, and does not border an enlarged pneumatic recess

1: well developed, crescent shaped, thin crest forms anterior edge of enlarged pneumatic recess

Character 7: Braincase, crista interfenestralis, location:

0: confluent with lateral surface of prootic and opisthotic

1: distinctly depressed within middle ear opening

Character 8: Braincase, subotic recess (pneumatic fossa ventral to fenestra ovalis):

0: absent

1: present

Note (via Brusatte et al., 2014): Carr et al. (2011) described a "subotic fossa and recesses" in *Teratophoneus*, but these are not similar to the large, invasive subotic recess of ornithomimosaurs and troodontids.

Character 9: Basisphenoid recess: (UNORDERED)

0: present between basisphenoid and basioccipital

1: entirely within basisphenoid

2: absent

Note (via Brusatte et al., 2014): This multistate character is not considered ordered because there is not a clear nested set of primary homologies. The basisphenoid recess is primitive for coelurosaurs, so it is not clear whether the reduced and absent conditions form a transformational sequence. Therefore, the character is left unordered. The reduction of the basisphenoid recess to lie entirely within the basisphenoid in *Tyrannosaurus* and *Tarbosaurus* is most likely due to the extreme anterposterior foreshortening of the recess in these taxa (character 158 in Brusatte et al. 2010a).

Character 10: Basisphenoid recess, posterior opening, form:

0: single opening

1: divided into two small, circular foramina by a thin bar of bone

Character 11: Parasphenoid, base of cultriform process (parasphenoid rostrum), pneumatization:

0: not highly pneumatized

1: expanded and pneumatic (parasphenoid bulla present)

Character 12: Braincase, basipterygoid processes, direction of projection: (UNORDERED)

0: ventral or anteroventrally projecting

1: lateroventrally projecting

2: laterally projecting

Character 13: Braincase, basipterygoid processes, form:

0: well developed, extending as a distinct process from the base of the basisphenoid

1: processes abbreviated or absent

Character 14: Braincase, basipterygoid processes, pneumatization:

0: absent or very subtle, process solid

1: processes hollow, invaded by pneumatic recess (anterior tympanic recess and/or basisphenoid recess)

Note (via Brusatte et al., 2014): Turner et al. (2012) scored hollow basipterygoid processes as absent in several tyrannosauroids. However, these are indeed present in taxa that have been CT scanned, including *Alioramus* (Bever et al. 2011), *Gorgosaurus* (Witmer and Ridgely 2009), and *Tyrannosaurus* (Witmer and Ridgely 2009). Therefore, we have scored the character as present in these tyrannosauroids and considered it questionable "?" in all tyrannosauroids (and other basal coelurosaurs) that have not been studied using CT. We anticipate that this character state will eventually be identified in many taxa once they have been the subject of CT study. We also suggest that there may be a difference between the extensively hollow basipterygoid processes of ornithomimosaurs and troodontids (which have long been noted without CT) and more subtle, less invasive recesses in other taxa (such as tyrannosauroids). For the time being, however, we consider these conditions homologous because it is difficult to differentiate them in a discrete manner.

Character 15: Basisphenoid, basipterygoid recesses on dorsolateral surfaces of basipterygoid processes:

0: absent

1: present

Note (via Brusatte et al., 2014): This character refers only to the presence of discrete external pneumatic foramina or fenestrae on the lateral surface of the basipterygoid processes (or immediately above). Brusatte et al. (2010a, character 157) utilized a different character relevant to the ingroup relationships of tyrannosauroids, concerning the presence or absence of a single large, window-like fenestra in this region, which is present in juveniles of *Bistahieversor*, *Alioramus*, and *Daspletosaurus* (but not adults of *Bistahieversor* and *Daspletosaurus*). Because of its ontogenetically variable nature, we do not use the Brusatte et al. (2010a) character here, although we incorporate it into a character relating to the anterior tympanic recess below. Rather, we retain the original character of the TWiG dataset which refers to the simple presence or absence of pneumatic foramina on the basipterygoid processes.

Character 16: Prootic, depression for pneumatic recess (Dorsal Tympanic Recess): (ORDERED)

0: absent

1: present as dorsally open fossa on prootic/opisthotic

2: present as deep, posterolaterally directed concavity

Note (via Brusatte et al., 2014): Brusatte et al. (2010a, character 163) utilized a similar character for tyrannosauroids, and we use those character scores here. Most notably, *Dilong* is scored as uncertain (not as possessing a dorsally open recess as in Turner et al. [2012]). *Guanlong*, however, does possess a dorsal tympanic recess.

Character 17: Braincase, accessory tympanic recess dorsal to crista interfenestralis: (ORDERED)

0: absent

1: small pocket present

2: extensive with indirect pneumatization

Character 18: Braincase, caudal (posterior) tympanic recess: (ORDERED)

0: absent

1: present as opening on anterior surface of paroccipital process

2: extends into opisthotic posterodorsal to fenestra ovalis, confluent with this fenestra

Note (via Brusatte et al., 2014): Derived tyrannosauroids possess state “2”, as can be seen by their inflated and heavily pneumatized paraoccipital processes (Witmer and Ridgely 2009; Bever et al. 2011,

2013). *Guanlong* clearly does not possess such extensively inflated paroccipital processes, but in the absence of CT data it is uncertain whether it possesses state 0 or state 1. Because of this uncertainty we conservatively score it as "[01]".

Character 19: Braincase, exits of cranial nerves X-XII, location and form: (ORDERED)

0: flush with surface of exoccipital

1: located together in a shallow bowl-like depression

2: located together in a deep, funnel-like depression

Note (via Brusatte et al., 2014): We have modified the original binary character of Turner et al. (2012) and previous TWiG versions into an ordered multistate, to differentiate between two conditions of the "bowl-like depression". The first condition, a shallow depression, is commonly seen in coelurosaurs. The second, an extremely deep funnel-like morphology, is present in some derived tyrannosauroids (Brusatte et al. 2010a, character 153). *Bistahieversor* and *Teratophoneus*, which were scored as "?" in Brusatte et al. (2010a), are now scored as possessing the funnel-like morphology based on recent reexaminations of both specimens by Thomas Carr (as noted in Bever et al. 2013).

Character 20: Premaxilla, maxillary process, extent and articulation: (UNORDERED)

0: contacts nasal to form posterior border of nares

1: reduced so that maxilla participates broadly in external naris

2: extends posteriorly to separate maxilla from nasal posterior to nares

Character 21: Premaxilla and nasal, internarial bar, morphology of external surface:

0: rounded

1: flat

Character 22: Premaxilla, crenulated margin on buccal edge of bone:

0: absent

1: present

Character 23: External naris, position of posterior margin:

0: farther anterior than antorbital fossa

1: nearly reaching or overlapping the anterior border of the antorbital fossa

Character 24: Premaxilla, shape of conjoined left and right bones in ventral view: (ORDERED)

0: acute, V-shaped

1: rounded, U-shaped, first two teeth oriented mediolaterally and third and fourth teeth oriented parasagitally

2: rounded, U-shaped, entire tooth row oriented mediolaterally

Note (via Brusatte et al., 2014): We have expanded this character into consider this character into an ordered multistate to take into account variation in the "rounded" condition in tyrannosauroids, as denoted by Brusatte et al. (2010a, character 10). All non-tyrannosauroids with rounded snouts are scored for state 1, as are basal tyrannosauroids. Derived tyrannosauroids in which the entire premaxillary tooth row is oriented mediolaterally are scored for state 2.

Character 25: Maxilla, anteromedial process (= "secondary palate"): (ORDERED)

0: short

1: long, with extensive palatal shelves on maxilla

2: extremely elongated, extending back at least to the level of alveolus 4

Note (via Brusatte et al., 2014): We have added a second derived state to the character of Turner et al. (2012) and previous TWiG analyses, to differentiate the extremely elongated anteromedial processes of tyrannosauroids, which extend back to at least the level of alveolus 4. The second derived state is present in all score-able tyrannosauroids, as well as ornithomimosaurs (e.g., *Struthiomimus*, *Pelecanimimus*, *Nqwebasaurus*) and the long-snouted dromaeosaurid *Austroraptor*). It is possible that this character may eventually diagnose a more inclusive clade of basal coelurosaurs, given that elongate anteromedial processes are present in nearly all coelurosaurs. However, it is currently difficult to distinguish between the long and extremely long processes of many coelurosaurs, because many specimens are preserved on slabs and therefore fine details of the medial maxilla are not visible. It is clear, however, that derived paravians such as *Dromaeosaurus* and *Deinonychus*, while possessing long anteromedial processes, do not possess the extremely elongate morphology of tyrannosauroids (e.g.., Currie 1995). This is also true of basal coelurosaurs such as *Zuolong* (Choiniere et al. 2010b). Whether compsognathids may have possessed the tyrannosauroid condition is somewhat unclear, because most preserved specimens are either preserved on slabs or are not preserved in disarticulation (precluding observation of the medial surface of the maxilla). However, high resolution photographs of *Compsognathus* published by Göhlich et al. (2006) indicate that

this taxon had a somewhat elongate palatal process, and definitely did not possess the extremely elongated condition of tyrannosauroids (therefore, we score it for state 1).

Character 26: Maxilla, palatal shelf, midline ventral "tooth-like" projection:

0: absent, palatal shelf flat

1: present

Character 27: Maxilla, maxillary fenestra: (ORDERED)

0: absent

1: present, fenestra occupies less than half of the depressed area between the anterior margins of the antorbital fossa and antorbital fenestra

2: present, fenestra large and takes up most of the space between the anterior margins of the antorbital fenestra and fossa

Note (via Brusatte et al., 2014): the second derived state refers only to the huge fenestrae of some troodontids, which are so large that they take up nearly the entire antorbital fossa region anterior to the antorbital fenestra, with only a thin strut of bone separating the two openings. Other characters referring to nuances of the size and position of the fenestrae are given below.

Character 28: Maxilla, maxillary fenestra, location:

0: situated at anterior border of antorbital fossa

1: situated posterior to anterior border of fossa

Note (via Brusatte et al., 2014): This is equivalent to character 17 in Brusatte et al. (2010a). Among tyrannosauroids, *Daspletosaurus*, *Tarbosaurus*, *Tyrannosaurus*, and *Sinotyrannus* have state 0. We here score *Dilong* for state 1, contra Turner et al. (2012). In *Dilong* the maxillary fenestra approaches the anterior border of the antorbital fossa, but it doesn't reach the border.

Character 29: Maxilla, promaxillary fenestra:

0: absent

1: present

Note (via Brusatte et al., 2014): We score *Eotyrannus* as possessing the fenestra, based on personal observation of the holotype (MIWG 1997.550)

Character 30: Nasal, pneumatization: (UNORDERED)

0: apneumatic or poorly pneumatized

1: with extensive pneumatic fossae, especially along posterodorsal rim of naris

2: with 2-3 large pneumatic openings, set into a fossa, on the lateral surface above the antorbital fenestra, leading into an extensive internal pneumatic cavity

Note (via Brusatte et al., 2014): Turner et al. (2012) utilized a binary character, and we added a new state based on Brusatte et al. (2010a, character 43). We do not consider the two pneumatic states to be directly homologous or form a nested series of homology, because of distinct differences in the region of the external pneumatopores (posterodorsal rim of naris vs. midpoint of bone) and the form of the pneumatopores (several pneumatopores posterodorsal to the rim of the naris and within the narial fossa vs. 2-3 discrete pneumatic openings centered in a fossa). The outgroups and some basal tyrannosauroids possess condition 2, whereas oviraptorosaurs possess condition 1. Turner et al. (2012) scored the outgroups (*Allosaurus* and *Sinraptor*) for state 0, and this was correct when the character was a simple binary distinction between no/poor pneumaticity and the extreme pneumaticity of oviraptorosaurs. The new condition added here recognizes morphological similarity between the outgroups and some basal coelurosaurs (basal tyrannosauroids), and probably optimizes as the ancestral condition for Coelurosauria.

Character 31: Jugal and postorbital, contribution to postorbital bar:

0: contribute equally to postorbital bar

1: ascending process of jugal reduced and descending process of postorbital ventrally elongate

Character 32: Jugal, dorsoventral height beneath lower temporal fenestra:

0: tall, twice or more as tall dorsoventrally as it is wide transversely

1: very short, jugal rod-like

Character 33: Jugal, pneumatic recess in posteroventral corner of antorbital fossa:

0: present

1: absent

Note (via Brusatte et al., 2014): This character refers to an internal pneumatic recess of the jugal, which hollows out some or all of the bone internally. As shown by Tahara and Larsson (2011), the presence of a discrete jugal diverticulum (located in the jugal portion of the antorbital fossa) does not always equate to the presence of an internal recess hollowing the jugal. The presence of an internal recess can be

confidently assessed by the presence of a discrete pneumatopore externally, which leads into the recess. *Pelecanimimus* possesses this pneumatopore (LH 7777) and is here scored for state 0, whereas all other ornithomimosaurs are scored for state 1 because they lack the external pneumatopore, and in the case of *Ornithomimus* are known to lack the internal recess based on CT scans (Tahara and Larsson 2011).

Character 34: Jugal, medial jugal foramen:

0: present on medial surface ventral to postorbital bar

1: absent

Note (via Brusatte et al., 2014): Turner et al. (2012) scored the foramen as absent in *Dilong*, but the medial surface of the jugal is obscured in the holotype specimen, which has not yet been the subject of CT study. Therefore, we have changed the score to "?".

Character 35: Quadratojugal, shape:

0: without horizontal process posterior to ascending process (reversed "L" shape)

1: with horiziontal posterior process (i.e., inverted 'T' or 'Y' shape)

Character 36: Jugal and quadratojugal, fusion:

0: absent

1: present, the two bones are not distinguishable from one another

Character 37: Lacrimal, supraorbital crests in adult individuals: (UNORDERED)

0: absent

1: dorsal crest above orbit

2: lateral expansion anterior and dorsal to orbit

Note (via Brusatte et al., 2014): Following Turner et al. (2012), we consider this an unordered character because we do not hypothesize primary homology between state 1 (discrete crests or tubercles along the dorsal margin of the lacrimal, as present in outgroups and tyrannosauroids) and state 2 (a lamina-like triangular sheet that expands laterally above the orbit, as present in troodontids). Turner et al. (2012) scored *Shuvuuia* for state 1, but we have rescored this as state 0 because this taxon does not possess the discrete crest/tubercle along the dorsal margin of the lacrimal as in outgroups and tyrannosauroids.

Character 38: Lacrimal, pneumatic foramina opening laterally at the junction of the anterior and ventral processes above the antorbital fenestra: (ORDERED)

0: absent

1: present, extent of pneumaticity limited to partially hollowing the bone in the region where the anterior and ventral rami meet

2: present and extensive, completely hollowing the bone where the anterior and ventral rami meet

Note (via Brusatte et al., 2014): Turner et al. (2012) used a binary character to distinguish between the presence/absence of lacrimal pneumaticity, and we add a third state to distinguish the greatly extensive pneumatic recess of some tyrannosauroids. Therefore, this is now an ordered character. Turner et al. (2012) scored *Eotyrannus* as lacking lacrimal pneumaticiy, but observation of the type specimen shows that it has a shallow pneumatic recess with external pneumatopores, as in *Guanlong*, *Dilong*, and other basal tyrannosauroids.

Character 39: Lacrimal, posterodorsal process: (ORDERED)

0: absent, lacrimal is inverted ‘L’ shaped or ‘7’ shaped in lateral view

1: present, lacrimal ‘T’ shaped in lateral view

2: present but reduced, anterodorsal process much longer than posterodorsal process

Note (via Brusatte et al., 2014): Turner et al. (2012) and previous TWiG datasets have referred to the presence of an “anterodorsal process”, which creates a T-shaped lacrimal, in this character. This T-shaped condition contrasts with the “inverted L” shape of most theropod lacrimals. However, the inverted L shape is due to a pronounced anterior process along the dorsal margin of the bone (the anterodorsal process) and the lack of any posterior process along the dorsal margin (the posterodorsal process). Therefore, we have reworded the character to refer specifically to the posterodorsal process. We also consider this an ordered character, because state 2 (the presence of a posterodorsal process that is much shorter than the anterodorsal process) is a sub-condition of state 1, meaning that there is a nested primary homology hypothesis. Brusatte et al. (2010a, character 48) utilized a character distinguishing the T-shaped and 7-shaped lacrimals of tyrannosauroids. We utilize this as a separate character below. *Zulong*, which possesses a small posterodorsal process that appears large because of the presence of an autapomorphic notch ventral to it, is scored for state 0.

Character 40: Prefrontal, exposure in dorsal view: (ORDERED)

0: large, dorsal exposure similar to that of lacrimal, forms much of orbital rim and usually separates or nearly separates frontal and lacrimal

1: greatly reduced in size, not exposed widely along the orbital rim and allows for wide contact between frontal and lacrimal

2: absent

Note (via Brusatte et al., 2014): We have slightly reworded this character based on language in Brusatte et al. (2010a, character 111). Turner et al. (2012) scored *Dilong* for a reduced frontal, based on the description in Xu et al. (2004). However, personal observation of the holotype (IVPP V14243) reveals that it is difficult to distinguish the prefrontal from surrounding bones, so we conservatively score this taxon as "?" following Brusatte et al. (2010a).

Character 41: Frontals, shape of conjoined left and right elements in dorsal view:

0: triangular, narrowing anteriorly as a wedge between nasals

1: rectangular, end abruptly anteriorly, suture with nasal transversely oriented

Note (via Brusatte et al., 2014): Brusatte et al. (2010a, character 113) distinguished between the broad triangular shape of most tyrannosauroid frontals and the anteroposteriorly foreshortened shape of derived tyrannosaurid frontals. However, the frontals of all tyrannosauroids are triangular, so we retain the current character and add a new character below to distinguish between the tyrannosauroid morphologies.

Character 42: Frontal, outline of anterior emargination of supratemporal fossa:

0: straight or slightly curved

1: strongly sinusoidal and reaching onto postorbital process

Character 43: Frontal, form of articulation between postorbital process and frontal orbital margin in dorsal view:

0: smooth transition from orbital margin

1: sharply demarcated from orbital margin

Note (via Brusatte et al., 2014): Turner et al. (2012) scored some derived tyrannosaurids for state "1", but we here rescore these as state "0," because they do not possess the strongly demarcated and discrete postorbital processes that are seen in some dromaeosaurids (which state "1" refers to).

Character 44: Frontal, form of lateral margin of lacrimal (or prefrontal) suture:

0: smooth

1: with discrete notch

Character 45: Parietals, form of dorsal surface and presence of sagittal crest(s): (ORDERED)

0: flat, lateral ridge borders supratemporal fenestra, sagittal crest (or crests) absent

1: convex with very low sagittal crest (or crests) along midline

2: convex with well-developed sagittal crest (or crests)

Note (via Brusatte et al., 2014): This character was unordered in Turner et al. (2012), but we here make it an ordered character because states 1 and 2 are a nested series of homologies defining progressively more prominent sagittal crests, whereas state 0 refers to the absence of a crest. This character subsumes character 122 in Brusatte et al. (2010a), which referred to the presence/absence of sagittal crests in tyrannosauroids.

Character 46: Parietals, fusion of left and right elements:

0: unfused

1: fused on the midline in sub-adults and adults (when known)

Note (via Brusatte et al., 2014): This character is equivalent to character 127 in Brusatte et al. (2010a).

Character 47: Squamosal, quadratojugal (descending) process, orientation of long axis:

0: dorsoventral or slightly oblique, essentially parallels quadrate shaft

1: anteroposterior, nearly perpendicular to quadrate shaft

Note (via Brusatte et al., 2014): This character is equivalent to character 92 of Brusatte et al. (2010a). Brusatte et al. (2010a) scored *Bistahieversor* for state 0, but we here change this to state 1. This miscoding was an error in the Brusatte et al. (2010a) analysis.

Character 48: Squamosal, contact of descending (ventral) process with quadratojugal:

0: present

1: absent, squamosal does not contact quadratojugal

Character 49: Squamosal, posterolateral shelf overhanging quadrate head:

0: absent

1: present

Character 50: Quadrate, orientation of shaft when in articulation:

0: vertical

1: strongly inclined anteroventrally so that ventral end lies far forward of dorsal end

Character 51: Quadrate shaft, form of lateral surface:

0: straight, without any prominent processes projecting laterally; quadrate foramen not visible in lateral view

1: with broad, triangular process along lateral edge of shaft contacting squamosal and quadratojugal above an enlarged quadrate foramen, which is often partially or largely visible in lateral view

Note (via Brusatte et al., 2014): We have slightly reworded the character to explain it more clearly.

Character 52: Foramen magnum, shape:

0: subcircular, slightly wider than tall

1: oval, taller than wide

Character 53: Occipital condyle, mediolateral width and dorsoventral height of neck linking condyle to remainder of braincase:

0: approximately the same width and height as the condyle

1: constricted in width and height relative to the condyle

Note (via Brusatte et al., 2014): We have reworded this character to make it clearer here, so that it refers specifically to a discrete neck that is constricted in width and height relative to the width of the condyle.

Character 54: Paroccipital processes, shape:

0: elongate mediolaterally and slender dorsoventrally compared to mediolateral length, with dorsal and ventral edges nearly parallel

1: process short mediolaterally, deep dorsoventrally compared to mediolateral length, with convex distal end

Character 55: Paroccipital processes, orientation in posterior view:

0: straight, projects laterally or slightly posterolaterally

1: downturned, distal end curves ventrally and is pendant

Note (via Brusatte et al., 2014): This character is not equivalent to character 150 in Brusatte et al. (2010a), which refers to a distinct ventral flange at the distal end of the paroccipital processes of some tyrannosauroids. This character is utilized below.

Character 56: Paroccipital process, orientation of dorsal edge:

0: straight

1: twisted anterolaterally at distal end

Character 57: Ectopterygoid, form of the opening into the pneumatic fossa on the ventral surface:

0: constricted opening relative to fossa

1: widely opened fossa

Note (via Brusatte et al., 2014): Turner et al. (2012) scored *Albertosaurus* and *Gorgosaurus* for state "0", which is unusual among coelurosaurs. Our observations of tyrannosauroids do not reveal any discrete differences between these taxa and other tyrannosauroids, both basal (e.g., *Guanlong*, *Xiongguanlong*) and derived (e.g., *Alioramus*, *Tarbosaurus*, *Tyrannosaurus*) forms. Therefore, all tyrannosauroids are scored "1" here.

Character 58: Ectopterygoid: dorsal recess:

0: absent

1: present

Note (via Brusatte et al., 2014): This character does not refer to the principal pneumatic recess of the ectopterygoid, which hollows out much of the bone and opens via a large pneumatopore on the ventral surface. Rather, it refers to a separate dorsal recess present in some dromaeosaurids.

Character 59: Pterygoid, morphology of the posterior flange for articulation with quadrate and epipterygoid:

0: well developed

1: reduced in size or absent

Note (via Brusatte et al., 2014): This character has been reworded here for clarity. See Balanoff et al. (2009) for a good description of state 1, which is present in oviraptorosaurs and a handful of other coelurosaurs.

Character 60: Palatine and ectopterygoid, articulation between the two bones:

0: absent, the two bones separated from each other by the pterygoid

1: present, the two bones contact each other

Character 61: Palatine, jugal process:

0: present, palatine tetraradiate in shape

1: absent, palatine triradiate in shape

Character 62: Skull, suborbital fenestra, size:

0: large, similar in anteroposterior length to the anteroposterior length of the orbit

1: reduced in size (less than one quarter orbital length) or absent

Character 63: Dentary, form of symphyseal region in dorsal or ventral view: (ORDERED)

0: approximately straight anteroposteriorly, paralleling lateral margin of the remainder of bone; conjoined dentaries narrow

1: recurved slightly medially relative to remainder of bone, conjoined dentaries U-shaped

2: recurved strongly medially relative to remainder of bone, conjoined dentaries forming broad U-shaped muzzle

Note (via Brusatte et al., 2014): This character has been reworded for clarity.

Character 64: Dentary, orientation of dorsal margin of symphyseal region in lateral view:

0: in line with the remainder of the dorsal (alveolar) margin of the dentary

1: downturned relative to the remainder of the dorsal margin

Character 65: Lower Jaw, coronoid prominence (usually located on surangular):

0: absent

1: present

Character 66: Dentary, contribution to the dorsal margin of the external mandibular fenestra: (ORDERED)

0: absent or slight, without discrete posterodorsal process

1: present, with discrete posterodorsal process above anterior end of fenestra

2: present and extensive, with elongate posterodorsal process extending over most of fenestra

Character 67: Dentary, morphology of lateral surface:

0: flat, tooth row approximately in line with the remainder of the lateral surface

1: bearing lateral ridge, tooth row inset from remainder of lateral surface

Character 68: Dentary, shape:

0: subtriangular in lateral view, with dorsoventral depth expanding posteriorly

1: strap-like in lateral view, with subparallel dorsal and ventral edges and a consistent dorsoventral depth across its length

Note (via Brusatte et al., 2014): Turner et al. (2012) scored some derived tyrannosauroids (*Eotyrannus*, *Gorgosaurus*, *Tyrannosaurus*, *Daspletosaurus*) for state “1”, whereas the basal tyrannosauroids *Dilong* and *Proceratosaurus* were scored for state “0”. This character was introduced to the TWiG matrix based on the descriptions of Currie (1995), who noted that some dromaeosaurids (and other coelurosaurs) had dentaries that maintained an approximately even depth across their entire lengths, unlike the dentaries of most theropods that funnel out in depth posteriorly (so-called “subtriangular” dentaries). The subtriangular condition is indeed present in basal tyrannosauroids, but also in more derived tyrannosauroids, contra Turner et al. (2012) (e.g., Brochu 2003; Currie 2003; Carr et al. 2011; Brusatte et al. 2012a). We here score all tyrannosauroids for state “0”. Turner et al. (2012) scored *Eotyrannus* for state “1”, seemingly because the preserved portions of the left and right dentaries of the holotype (MIWG 1997.550) are gracile and do not expand posteriorly. However, neither of these dentaries are complete posteriorly, so the degree of expansion cannot be assessed. Therefore, we here score *Eotyrannus* as “?”.

Character 69: Dentary, primary neurovascular foramina on lateral surface, arrangement:

0: distinct but superficial foramina

1: distinct foramina lie within a deep and sharp groove across the middle and posterior regions of the dentary

Note (via Brusatte et al., 2014): This character is equivalent to character 176 in Brusatte et al. (2010a). It has long been utilized in the TWiG matrix, with state 1 scored for a handful of coelurosaurs. Brusatte et al. (2010a) recognized that a distinct and sharp groove is also present in the basal tyrannosauroids *Guanlong*, *Proceratosaurus*, and *Sinotyrannus*.

Character 70: Lower jaw, external mandibular fenestra, shape

0: oval

1: subdivided by a spinous anterior process of the surangular

Character 71: Lower jaw, internal mandibular fenestra (opening between splenial and prearticular on medial surface of mandible), size and shape:

0: small and slit-like

1: large and rounded

Note (via Brusatte et al., 2014): This character was introduced into the TWiG dataset based on Currie (1995), who describe an unusual morphology of the internal mandibular fenestra in some dromaeosaurids, in which this opening is large and circular. This contrasts with the smaller and more ovoid opening of most theropods, which is often reduced to a small slit. Turner et al. (2012) scored *Tyrannosaurus* for a large and rounded fenestra, but as shown by Brochu (2003) this opening, although absolutely large due to the large size of Tyrannosaurus, is not circular and is smaller in relation to the size of the dentary than in dromaeosaurids. Therefore, *Tyrannosaurus* and all other tyrannosauroids with known splenials are here scored for state 0. This state can be inferred in isolated splenials by the size and shape of the notch along the posterior margin, which forms the anterior margin of the internal mandibular fenestra.

Character 72: Surangular, foramen in lateral surface of surangular anterior to the mandibular articulation: (ORDERED)

0: absent

1: present but small

2) present and large, approximately 30% of the dorsoventral depth of the posterior surangular

Note (via Brusatte et al., 2014): This multistate ordered character combines two characters in the Turner et al. (2012) dataset: character 74 that scores the presence or absence of the surangular foramen and character 258 that scores variability in size of the foramen. The latter character is also used by Brusatte et al. (2010a, character 179) to distinguish between the small and extremely large foramina of some tyrannosauroids. This ordered multistate better encapsulates the full range of variation among not only coelurosaurs, but also tyrannosauroids (basal taxa such as *Guanlong* lack the foramen all together, whereas intermediate taxa such as *Eotyrannus* have a small foramen and derived tyrannosauroids have a derived enlarged foramen). Turner et al. (2012) scored *Eotyrannus* as “?”, but the recent realization that the holotype (MIWG 1997.550) includes surangular material shows that *Eotyrannus* has a small foramen.

Character 73: Splenial, exposure in lateral view:

0: not widely exposed on lateral surface of mandible

1: exposed as a broad triangle between dentary and angular on lateral surface of mandible

Character 74: Lower jaw, coronoid ossification, presence and shape: (ORDERED)

0: present as a large, triangular bone (fused with the supradentary)

1: present but reduced to a thin splint

2: absent

Character 75: Articular, elongate and slender medial process emanating from retroarticular process (can project medially, posteromedially, or dorsomedially):

0: absent

1: present

Note (via Brusatte et al., 2014): Turner et al. (2012) scored *Dilong* and *Daspletosaurus* as lacking the process, and *Gorgosaurus* and *Tyrannosaurus* as possessing it. We could not confirm any observation of the articular medial process on *Dilong*, so we score this taxon as "?". *Daspletosaurus* has the same general morphology of *Tyrannosaurus* and *Gorgosaurus*, which a pronounced dorsally and medially extending process at the posteromedial corner of the retroarticular process (Currie 2003). Therefore, we score it for state 1. We also have identified this process in *Tarbosaurus* and *Proceratosaurus*.

Character 76: Articular, retroarticular process, presence and shape: (UNORDERED)

0: present, short and stout, with a distinct region between the glenoid and the portion of the retroarticular process to which the jaw depressors attached

1: present, elongate and slender

2: present but extremely reduced, with no (or only a very short) margin between the glenoid and the muscle attachment region

Note (via Brusatte et al., 2014): We have modified this character to include an additional state signifying the extremely reduced retroarticular processes of tyrannosauroids. In tyrannosauroids, including basal taxa such as *Guanlong* and *Dilong*, the retroarticular process is so short that there is essentially no separation between the mandibular glenoid and the attachment site (on the retroarticular process) for the jaw depressor muscles. This is not the case in *Tanycolagreus*, which has a longer retroarticular process and a wider margin between the glenoid and muscle attachment site (Carpenter et al. 2005a, fig. 2.4). In

tyrannosauroids lacking an articular, the short retroarticular process can be inferred with a high degree of confidence by the short retroarticular region of the surangular, which articulates with the angular. This inferral is based on the one-to-one correspondence of the short surangular process and short articular retroarticular process in all known tyrannosauroids.

Character 77: Lower jaw, glenoid articular surface for mandible, anteroposterior length:

0: approximately as long as distal quadrate condyles

1: twice or more as long as distal quadrate condyles, allowing anteroposterior movement of mandible

Note (via Brusatte et al., 2014): This character can usually be confidently inferred from the shape of all or part of the glenoid articular surface on the surangular and articular. Oviraptorosaurs, which possess state 1, have a radically different morphology compared to other theropods. In oviraptorosaurs, the quadrate condyles do not tightly articulate with the mandibular glenoid, and the lack of a clear, corresponding fit can be seen by observing the surangular and articular.

Character 78: Premaxilla, teeth:

0: present

1: absent

Character 79: Premaxillary dentition, size of second premaxillary tooth:

0: approximately equivalent in size to other premaxillary teeth

1: markedly larger than third and fourth premaxillary teeth

Character 80: Maxilla, teeth:

0: present

1: absent

Character 81: Maxillary and dentary teeth, serrations: (UNORDERED)

0: present on all known teeth

1: some teeth without serrations on mesial (anterior) carina (except at base in *S. mongoliensis*)

2: all known teeth without serrations on both mesial and distal carinae

Note (via Brusatte et al., 2014): This character is considered unordered, because we do not hypothesize a nested sequence of primary homology. In other words, we do not hypothesize that there is a

clear transformational sequence from serrations to only distal serrations to no serrations, although we recognize that this is a possibility that needs testing without assuming that the character is ordered. We score *Zuolong* for state 1. Choiniere et al. (2010b) described the mesial carina of an isolated lateral tooth as lacking serrations, but considered it difficult to rule out the absence of serrations across the entire carina because of the missing apical tip of the tooth. We feel that enough of the tooth is preserved to show that serrations certainly weren't present across most of the length of the carina, and if were present, were restricted to the very apical tip of the tooth. We consider this equivalent to state 1.

Character 82: Maxillary and dentary teeth, size:

0: large

1: small (25-or more teeth in dentary when complete series is observable)

Note (via Brusatte et al., 2014): The "large" condition is the normal condition for theropods in which 10-20 distinct maxillary teeth and 10-20 distinct dentary teeth are present. The "small" condition refers to the higher number of tightly packed teeth in the maxillae and dentaries of some coelurosaurs. Although a complete or near-complete maxilla and/or dentary are needed to score this character confidently, usually the size of individual teeth can be used to predict the state of this character.

Character 83: Dentary teeth, implantation:

0: in separate alveoli

1: set in open groove

Character 84: Maxillary and dentary teeth, serrations (denticles):

0: large

1: small

Note (via Brusatte et al., 2014): Turner et al. (2012) and previous versions of the TWiG matrix have followed Farlow et al. (1991) in quantifying this difference. In the current dataset, along with previous TWiG matrices, the "large" condition refers only to the pronounced, coarse denticles of therizinosauroids and troodontids (~1-5 serrations per millimeter), whereas other coelurosaurs are scored for the "small" condition (~more than 5 serrations per millimeter).

Character 85: Maxillary and dentary teeth, serrations, form:

0: simple, convex, and approximately perpendicular to the long axis of the tooth

1: large, hooked and pointing toward the tip of the crown on the distal and often mesial carinae

Character 86: Maxillary and dentary teeth, constriction between root and crown:

0: present

1: absent, root and crown confluent

Character 87: Dentary teeth, spacing between teeth:

0: evenly spaced across tooth row

1: anterior dentary teeth smaller, more numerous, and more closely appressed than those in middle of tooth row

Character 88: Dentary, interdental plates, form:

0: distinct internal plates absent (although a homologue to the plates is probably present)

1: distinct, clearly demarcated interdental plates medially between teeth

Note (via Brusatte et al., 2014): As reviewed by Turner et al. (2012), state 0 refers to the condition in dromaeosaurids and troodontids in which clear, distinct, unambiguously demarcated interdental plates are absent. It is likely, however, that bony tissue homologous to the interdental plates is present in this region but has been morphologically transformed.

Character 89: Premaxillary tooth crowns, position of mesial carina: (ORDERED)

0: along mesial margin of tooth, tooth cross section sub-oval to sub-circular

1: rotated distally on premaxillary teeth 1 and 2, such that anterior teeth have an asymmetrical cross section (D-shaped, with highly convex labial surface and flat lingual surface, with narrow spacing between mesial and distal carinae)

2: rotated distally on all premaxillary teeth, such that all teeth are D-shaped in cross section.

Note (via Brusatte et al., 2014): Turner et al. (2012) and previous TWiG matrices have utilized a binary character relating to tooth cross sectional shape, differentiating “sub-oval” and “sub-circular” teeth, which are present in the outgroups and most coelurosaurs, from “D-shaped” teeth, which are present in tyrannosauroids and a few other coelurosaurs. We note that some outgroups (allosauroids) and many coelurosaurs do possess premaxillary teeth that are roughly D-shaped in cross section, due to a labial margin that is somewhat convex and a lingual margin that is somewhat flat (and always flatter than the labial margin). For this character, however, we define D-shaped teeth based on the morphology of the

mesial carina. A “D-shaped” tooth is held to be a tooth in which the mesial carina is rotated strongly distally, such that it is present on the lingual surface of the tooth. This condition is seen almost exclusively in tyrannosauroids, and roughly follows the definition of D-shaped teeth by Currie et al. (1990), Choiniere et al. (2010b), and previous authors. This condition is always associated with a highly convex labial surface and a flat lingual surface, on which the two carinae are only very narrowly separated. In contrast, the somewhat D-shaped teeth of allosauroids and other coelurosaurs exhibit wider separation between both carinae on the lingual surface and a less convex labial surface. Therefore, these morphologies are not considered homologous to the “D-shaped” condition as defined here, although we recognize that there may be some homology shared between these taxa and coelurosaurs with true D-shaped teeth that is not present in more basal theropods such as coelophysoids, ceratosaurs, and some basal tetanurans (assessing this hypothesis awaits further study of theropod teeth more generally). Furthermore, following Brusatte et al. (2010a, character 196), we divide the “D-shaped” condition into two character states, which we consider to be nested homologies. Although these characters refer to the presence “D-shaped” morphology of either some (state 1) or all (state 2) of the tooth row, we here score all taxa lacking any sign of “D-shaped” teeth with rotated mesial carinae for state 0. It is possible that some of these taxa that do not preserve entire premaxillary tooth rows may eventually be scored for state 1, but we conservatively score them 0 here to minimize missing data.

Character 90: Cervical vertebrae, number:

0: $\leq$10

1: 12 or more

Character 91: Axis, epipophyses, form:

0: absent or poorly developed as a small pyramidal mound, not extending past posterior edge of postzygapophyses

1: large, rugose, and posteriorly directed flange, extending far beyond postzygapophyses

Note (via Brusatte et al., 2014): This character is equivalent to Brusatte et al. (2010a, character 210). The form of the axial epipophyses in tyrannosauroids conforms to the form of the epipophyses in the anterior-middle postaxial cervicals, so if these cervicals are well preserved they can be used as a confident

proxy for scoring this character. Because this correspondence is not necessarily clear for coelurosaurs as a whole, we only use anterior-middle cervicals as a proxy for tyrannosauroids.

Character 92: Axis, neural spine, form of dorsal portion:

0: flared transversely

1: compressed mediolaterally

Character 93: Cervical vertebrae, epipophyses, position:

0: placed distally on postzygapophyses, above postzygapophyseal facets

1: placed proximally, anterior to postzygapophyseal facets

Character 94: Cervical vertebrae, position of centrum in anterior cervical vertebrae:

0: terminates level with or anterior to the posterior extent of the neural arch

1: extending beyond the posterior limit of the neural arch

Character 95: Cervical vertebrae, carotid process on posterior cervical vertebrae:

0: absent

1: present

Character 96: Cervical vertebrae, shape of anterior articular surface of anterior cervical centra:

0: subcircular or square in anterior view

1: distinctly wider than high, kidney shaped

Character 97: Cervical vertebrae, neural spines, shape in dorsal view:

0: anteroposteriorly long

1: short and centered on neural arch, giving arch an "X" shape in dorsal view

Note (via Brusatte et al., 2014): This is equivalent to character 211 in Brusatte et al. (2010a).

Character 98: Cervical vertebrae, number of pneumatic foramina on lateral surface of centra:

0: one on each side

1: two on each side

Character 99: Cervical and anterior trunk vertebrae, form: (UNORDERED)

0: amphiplatyan or weakly opisthocoelous (anterior surface flat or weakly convex, posterior surface is flat or weakly concave)

1: strongly opisthocoelous (anterior surface is convex and posterior surface concave)

2: at least partially heterocoelous

Note (via Brusatte et al., 2014): Although the cervicals of tyrannosauroids and some other coelurosaurs are often referred to as "opisthocoelous," this character distinguishes between very weak opisthocoely in which the anterior surface is only very slightly convex (state 0, considered homologous to the amphiplatyan condition) and strong opisthocoely in which the anterior face is strongly convex and fits into a deep concave socket on the posterior surface of the preceding vertebra (state 1). This latter condition is present in *Dilong* and *Eotyrannus* among tyrannoauroids, *Compsognathus*, some alvarezsaurids, and rarely among other coelurosaurs.

Character 100: Dorsal vertebrae, hypapophyses in anterior trunk vertebrae:

0: absent or very small

1: large and pronounced

Character 101: Dorsal vertebrae, parapophyses in posterior trunk vertebrae, form:

0: flush with neural arch

1: distinctly projected on pedicels

Character 102: Dorsal vertebrae, hyposphene-hypantrum articulations:

0: absent

1: present

Character 103: Dorsal vertebrae, opposing zygapophyses on the same vertebra: (ORDERED)

0: abutting or nearly abutting one another above neural canal, opposite hyposphenes (if present) meet to form a single structure (lamina or rectangular projection)

1: zygapophyses placed distinctly lateral to neural canal and hyposphenes (if present) separated as two widely-spaced laminae, which are joined medially by an inset web of bone

2: zygapophyses placed distinctly lateral to neural canal and hyposphenes (if present) separated as two widely-spaced lamina, which are separated medially by a deep groove (the inset web of bone in state 1 is absent)

Note (via Brusatte et al., 2014): We have modified this character by dividing state 1 of Turner et al. (2012), which scores for the absence/presence of abutting postzygapophyses, into two states. Both of these states are applicable to taxa with widely separated postzygapophyses and hyposphenes on the

midline. Most coelurosaurs have this condition, in which separate left and right hyposphenes take the form of vertical sheets, which are distinctly separated from each other. However, the region between the separated hyposphenes can either house a web of bone, which is only slightly inset from the hyposphenes (state 1), or the web of bone is lost and the hyposphenes and zygapophyses are separated by a deep groove (state 2). Some coelurosaurs have left and right hyposphenes that are appressed to each other on the midline due to closely appressed left and right zygapophyses (*Alxasaurus*, derived tyrannosauroids). This is also the condition in outgroups (e.g., *Allosaurus*), and these taxa and the outgroups are scored for state 0. This character is ordered because the states describe a progressive sequence of conjoined-separation-loss of separating web.

Character 104: Cervical vertebrae, pneumaticity:

0: absent

1: present

Character 105: Dorsal vertebrae, transverse processes of anterior dorsals, form:

0: long (in mediolateral direction) and thin (in anteroposterior direction)

1: short (in mediolateral direction), wide (in anteroposterior direction), and only slightly inclined

Character 106: Dorsal vertebrae, neural spines, mediolateral expansion of dorsal end:

0: absent

1: present, expanded to form 'spine table'

Character 107: Dorsal vertebrae, scars for interspinous ligaments, position:

0: terminate at apex of neural spines

1: terminate below apex of neural spine

Character 108: Sacral vertebrae, number: (ORDERED)

0: 5 or less

1: 6

2: 7

3: 8

4: 9

5: 10

6: 11 or more

7: 15 or more

Character 109: Sacral vertebrae, fusion of zygapophyses:

0: absent, or zygapophyses partially fused to each other but still retaining the morphology of the original discrete structures

1: present, completely fused zygapophyses forming a sinuous ridge in dorsal view

Character 110: Sacral centra, ventral surface of posterior sacrals, form: (UNORDERED)

0: gently rounded, convex

1: ventrally flattened, sometimes with shallow sulcus

2: centrum strongly constricted transversely, ventral surface keeled

Character 111: Sacral vertebrae, pneumatic foramina on lateral surfaces of centra: (ORDERED)

0: absent on sacral vertebrae

1: present on anterior sacrals only

2: present on all sacrals

Character 112: Sacral vertebrae, morphology of the posterior articular face of the last sacral centrum:

0: flat or slightly concave

1: convex

Character 113: Caudal vertebrae, change in morphology of free caudals along the tail:

0: present, with distinct transition point from shorter centra with long transverse processes proximally to longer centra with small or no transverse processes distally

1: absent, vertebrae homogeneous in shape, without transition point

Character 114: Caudal vertebrae, location of transition point along the tail: (ORDERED)

0: begins distal to the 10th caudal vertebra

1: between the 7th and 10th caudal vertebra

2: or proximal to the 7th caudal vertebra

Character 115: Caudal vertebrae, morphology of anterior caudal centra: (UNORDERED)

0: tall, oval in cross section

1: with box-like centra in caudals I-V

2: anterior caudal centra laterally compressed with ventral keel

Character 116: Caudal vertebrae, neural spines, form:

0: simple, undivided

1: separated into anterior and posterior alae throughout much of caudal sequence

Character 117: Caudal vertebrae, neural spines on distal caudals (distal to ~caudal 15), form: (ORDERED)

0: present as a low ridge

1: absent

2: absent and location of spine replaced by midline sulcus in center of neural arch

Note (via Brusatte et al., 2014): Turner et al. (2012) scored *Dilong* for state 1, but we conservatively rescore this taxon as "?" because we have been unable to observe the caudal vertebrae directly, and also based on the illustrations in Xu et al. (2004), which seem to indicate that there may indeed be remnants of the neural spines on caudals in the vicinity of caudal 15 (state 0). Turner et al. (2012) may have scored this taxon as state 1 based on Xu et al.'s (2004) illustrations of extreme distal caudal vertebrae, which do lack neural spines. However, this is also the case in the extreme distal caudals of tyrannosaurids (e.g., *Tyrannosaurus*) and outgroups (*Allosaurus*), both of which Turner et al. (2012) scored for state 0. What we recognize as state 1 here is the condition in taxa such as *Compsognathus* (Peyer 2006), in which neural spines cease to be recognizable as discrete structures around ca. caudal 15 (in *Tyrannosaurus* and *Allosaurus* this happens much more posteriorly in the tail, ca. caudal 30: Brochu 2003).

Character 118: Caudal vertebrae, length of prezygapophyses of distal caudals: (UNORDERED)

0: between 1/3 and whole centrum length

1: extremely long (up to 10 vertebral segments long in some taxa)

2: strongly reduced or absent, terminate at approximately the anterior level of the centrum

3: prezygapophyses present but negligible in size, clasping the posterior surface of neural arch of preceding vertebrae, postzygapophyses negligible (this is an autapomorphy of *Ichthyornis dispar*)

Note (via Brusatte et al., 2014): Turner et al. (2012) scored *Dilong* for state 2, but personal observation of the holotype and the figures of Xu et al. (2004) show that state 0 is the correct score.

Character 119: Caudal vertebrae, number: (ORDERED)

0: more than 40

1: 35-40

2: 25-35

3: 8-25

4: less than 8 free caudal vertebrae, tail very short

Note (via Brusatte et al., 2014): We have modified this character by breaking down what was previously a single state (25-40 caudals) into two states, which better allows this character to differentiate the moderately elongate tails of ornithomimosaurs and alvarezsauroids (which have approximately 35 vertebrae) from the shortened tails of therizinosauroids, ornithomimosaurs, and many paravians (which have approximately 25 vertebrae).

Character 120: Chevrons, form of chevrons from proximal part of tail:

0: long and slender, proximal end short anteroposteriorly and shaft cylindrical

1: short and stout, proximal end elongate anteroposteriorly and shaft flattened and plate-like

Note (via Brusatte et al., 2014): Turner et al. (2012) scored various compsognathids for state 1, but we have rescored these taxa for state 0, as their proximal chevrons are elongate bones (with anteroposteriorly short proximal ends) that are proportionally similar to other taxa with state 0, such as outgroups (*Allosaurus*) and tyrannosauroids (e.g., *Tyrannosaurus*), and distinct from the short and stout proximal chevrons of other taa scored for state 1 (such as dromaeosaurids).

Character 121: Chevrons, form of chevrons from distal part of tail: (ORDERED)

0: simple

1: anteriorly bifurcate

2: bifurcate at both ends

Character 122: Cervical ribs, shaft: (UNORDERED)

0: slender and longer than vertebra to which they articulate

1: broad and shorter than vertebra

2: extremely thin and slender, hair-like

Note (via Brusatte et al., 2014): We have modified this character to include a third state designating the extremely thin and hair-like cervical ribs of compsognathid-grade theropods, as recognized by Göhlich and Chiappe (2006). This character is unordered because there is no clear set of nested homologies or no clear character transformation sequence.

Character 123: Ossified uncinate processes: (ORDERED)

0: absent

1: present and unfused to ribs

2: present and fused to ribs

Character 124: Ossified ventral (sternal) rib segments:

0: absent

1: present

Character 125: Gastralia, lateral gastral segment, size:

0: shorter than medial one in each lateral-medial gastralium set

1: longer than the medial one

Note (via Brusatte et al., 2014): We have modified this character following Claessens (2004). Claessens showed that in outgroups (*Allosaurus*) and tyrannosaurids (*Gorgosaurus*, *Tyrannosaurus*) the medial gastral segment is longer than the lateral gastral segment (state 0), contra the scores in Turner et al. (2012) and previous TWiG matrices. Furthermore, Norell and Makovicky (1997) showed that in *Velociraptor* the lateral segment is longer than the medial segment (state 1), which was affirmed by Claessens (2004) but contra the score in Turner et al. (2012). We suspect that scores 0 and 1 may have been swapped for some or all taxa accidentally in the TWiG matrix. Because of the confusion of scores in the original TWiG matrix, we conservatively take all character scores from Claessens (2004) and leave all other taxa as "?", unless observed personally by S. Brusatte or specifically reported in the literature.

Character 126: Sternum, ossified sternal plates, fusion:

0: absent, left and right plates separate in adults

1: present, left and right plates fused

Character 127: Sternum, lateral xiphoid process posterior to costal margin:

0: absent

1: present

Character 128: Sternum, groove on anterior edge for reception of coracoid:

0: present

1: absent

Character 129: Sternum, position of articular facet for coracoid (conditions may be determined by the position of the the articular facet on coracoid in taxa without ossified sternum):

0: anterolateral or more lateral than anterior

1: almost anterior

Character 130: Furcula, hypocledium: (**ORDERED)**

0: absent

1: present as tubercle

2: present as an elongate process

Character 131: Scapula, orientation of acromion margin:

0: continuous with blade

1: anterior edge laterally everted relative to blade

Character 132: Coracoid, expansion of posterolateral surface ventral to glenoid fossa:

0: unexpanded

1: posterolateral edge of coracoid expanded to form triangular subglenoid fossa bounded laterally by enlarged coracoid tuber

Character 133: Scapula and coracoid, fusion:

0: absent, two bones separate

1: present, two bones fused into scapulacoracoid

Character 134: Coracoid, shape in lateral view: (UNORDERED)

0: subcircular, with shallow ventral blade

1: subquadrangular with extensive ventral blade

2: shallow ventral blade with elongate posteroventral process

3: height more than twice width, coracoid strut-like

Character 135: Scapula and coracoid, form of their articulation:

0: form a continuous arc in posterior and anterior views

1: coracoid inflected medially relative to scapula, scapulocoracoid ‘L’ shaped in lateral view

Character 136: Scapula and coracoid, glenoid fossa, orientation of articular surface:

0: faces posteriorly or posterolaterally

1: faces laterally

Character 137: Scapula, length compared to length of humerus:

0: longer

1: approximately same length or shorter

Note (via Brusatte et al., 2014): In *Guanlong*, the scapula and humerus are approximately the same length (Xu et al. 2006), so this taxon is scored for state 1. The same scoring is used for all ornithomimosaurs, which have humeri that are slightly longer than the scapula (Osmólska et al. 1972) or have humeri and scapulae of approximately the same length (e.g., Nicholls and Russell 1985; Kobayashi and Lü 2003; Kobayashi and Barsbold 2005; Makovicky et al. 2010).

Character 138: Humerus, deltopectoral crest, extent and morphology: (UNORDERED)

0: large and distinct, proximal end of humerus quadrangular or triangular in anterior view

1: deltopectoral crest less pronounced, forming an arc rather than being quadrangular

2: deltopectoral crest very weakly developed, proximal end of humerus with rounded edges

3: deltopectoral crest extremely long and rectangular

Character 139: Humerus, deltopectoral crest, form of anterior surface:

0: smooth

1: with distinct muscle scar near lateral edge along distal end of crest for insertion of biceps muscle

Character 140: Ulna, olecranon process, size:

0: weakly developed

1: distinct and large

Character 141: Ulna, morphology of distal articular surface (dorsal condyle and dorsal trochlea in birds):

0: flat

1: convex, semilunate surface

Character 142: Ulna, morphology of proximal surface:

0: a single continuous articular facet

1: divided into two distinct fossae (one convex, the other concave) separated by a median ridge

Character 143: Lateral proximal carpal (ulnare?), shape in proximal view:

0: quadrangular

1: triangular

Character 144: Distal carpals in contact with metacarpals, number:

0: two separate carpals, one covering the base of metacarpal I (and perhaps contacting metacarpal II) the other covering the base of metacarpal II

1: a single distal carpal capping metacarpals I and II

Character 145: Semilunate carpal, placement relative to metacarpals: (UNORDERED)

0: medially placed, mostly overlapping metacarpals I and II, marginally or not overlapping metacarpal III

1: laterally shifted, marginally or not overlapping metacarpal I, significantly overlapping metacarpal III

Note (via Cau et al., 2015): Previously, the character included four states that partially overlapped, describing size and placement of the semilunate carpal, but was simplified to describe exclusively the relative placement of the semilunate carpal instead of the combination of size and position.

Character 146: Metacarpal I, length: (ORDERED)

0: less than half the length of metacarpal II

1: approximately half of the length (~50-70%) of metacarpal II

2: subequal in length to metacarpal II

Note (via Brusatte et al., 2014): We have modified the character of Turner et al. (2012) to include a new state, signifying the condition in some tyrannosauroids (and outgroups and other coelurosaurs) in which metacarpal I is approximately half of the length of metacarpal II. In essence, we have divided the original “half or less of the length of metacarpal II” character into two distinct states. This follows the character of Brusatte et al. (2010a, character 252). We have also removed the state in Turner et al.’s (2012)

character that refers to the autapomorphically short and robust metacarpals I of alvarezsaurids, which are wider transversely than long proximodistally. We now provide a separate character denoting the presence or absence of this peculiar morphology below.

Character 147: Third manual digit, number of phalanges: (ORDERED)

0: four

1: three

2: two

3: one

4: splint metacarpal bearing no phalanges

Note (via Cau et al., 2015): States redefined to better describe variation among theropods.

Character 148: Manual unguals, curvature: (ORDERED)

0: strongly curved, flexor margin deeply concave

1: weakly curved, flexor margin shallowly concave (third ungual may be straight)

2: all manual unguals straight

Note (via Brusatte et al., 2014): We have divided the original character of Turner et al. (2012) and previous TWiG matrices, which related to both the curvature and size of the flexor tubercle, into two separate characters. This character now refers to the degree of curvature, whereas a separate character has been added below for the size of the flexor tubercle. Furthermore, a separate character has also been added to simply denote the presence and absence of manual unguals (to distinguish the condition of derived avialans in which unguals are absent, which was originally one of several unordered character states in Turner et al.'s character). As constructed, this current character on manual ungual curvature subsumes c haracter 255 of Brusatte et al. (2010a). We here score *Dryptosaurus* for state 0 (strongly curved manual unguals) based on the recent redescription of this taxon by Brusatte et al. (2011c), contra the "weak curvature" score in Brusatte et al. (2010a). Many ornithomimosaurs have a straight manual ungual 3 but more curved manual unguals 1 and 2. These taxa are here scored for state 1, whereas those ornithomimosaurs with straight manual digits 1-3 are scored as state 2. We do not differentiate state 1 into multiple states for "weak curvature in all unguals" vs. "weak curvature in unguals 1 and 2 and a straight ungual 3," because derived tyrannosauroids do not possess a manual ungual 3, which would make it

impossible to score them for this character as currently constructed. Therefore, we conservatively lump together these two conditions into state 1, but recognize that future authors may advocate subdividing this character (but this would necessitate scoring derived tyrannosaurids as inapplicable, or dividing this character into multiple characters).

Character 149: Manual unguals, size of ungual on digit I compared to the other manual unguals in the hand:

0: generally similar in size

1: distinctly larger

Character 150: Manual unguals, a transverse ridge immediately dorsal to the articulating surface ("proximodorsal lip"):

0: absent

1: present

Character 151: Ilium, preacetabular process, anteroventral corner, form: (UNORDERED)

0: subtriangular, ventral margin of preacetabular process is shallowly concave

1: subquadrate with recurved anterior margin, ventral margin of preacetabular process is deeply concave such that the open region between the pubic peduncle and anteroventral region of the preacetabular process defines much of a circle

2: process strongly hooked

Note (via Brusatte et al., 2014): This character subsumes Brusatte et al. (2010a, character 259). State 1 is seen in some derived tyrannosauroids and ornithomimosaurs (and a few other coelurosaurs), and specifically refers to an anteroventral corner of the preacetabular process that is so recurved and ventrally projecting that the notch between it and the pubic peduncle is wide and essentially circular. Turner et al. (2012) and previous TWiG matrices scored the ougroups (*Allosaurus* and *Sinraptor*) as possessing state 1. However, although these taxa do have somewhat of a hooked anterior margin of the preactabular process (as do some compsognathids such as *Sinocalliopteryx*), they do not have the extremely pronounced condition of derived tyrannosauroids.

Character 152: Ilium, preacetabular process, length: (ORDERED)

0: roughly as long as postacetabular process

1: markedly longer (more than 2/3 of total ilium length) than postacetabular process

2: postacetabular blade much longer than postacetabular process

Character 153: Ilium, morphology of anterior margin of preacetabular process: (UNORDERED)

0: gently rounded or straight

1: anterior end strongly convex, lobate

2: pointed at anterodorsal corner with concave anteroventral edge

3: distinctly concave dorsally

Note (via Brusatte et al., 2014): Turner et al. (2012) scored *Dilong* for state 0, which is unexpected if it is a tyrannosauroid (as all known tyrannosauroids possess state 3). However, based on personal observation of the specimen (IVPP V14243) the anterodorsal margin of the ilium is broken, so we here score this taxon as "?". *Sinocallipteryx* is described as possessing a "slightly concave" anterodorsal margin of the preacetabular process, and this is clearly shown in an accompanying figure (Ji et al. 2007). Although we would prefer to confirm this score based on the original specimen, in order to determine whether it is genuine or a potential artifact of breakage, we here score *Sinocallipteryx* for state 3. Li et al. (2010) noted that *Mirischia* may possess an anterodorsal concavity, but personal observation of the specimen (SMNK 2349 PAL) shows that this region is broken, and therefore we score this taxon as "?".

Character 154: Ilium, supraacetabular crest on ilium, form: (ORDERED)

0: present as a separate process from the antitrochanter, and forming a "hood" over the femoral head

1: reduced but still separate from the antitrochanter, not forming "hood"

2: absent

Character 155: Ilium, postacetabular process, shape of distal end: (UNORDERED)

0: squared

1: acuminate

2: squared, with nearly vertical posterior margin that is nearly equivalent in depth to the anterior margin of the preacetabular process

Note (via Brusatte et al., 2014): We have expanded this character into an unordered multistate, by adding a third state referring to the extremely deep and squared-off postacetabular processes of some

derived tyrannosauroids such as *Alioramus* and *Tyrannosaurus*, as noted by Brusatte et al. (2010a, character 267). This condition differs from the shallower postacetabular processes of more basal tyrannosauroids such as *Guanlong*, *Dilong*, and *Juratyrant*. In these taxa the postacetabular process is squared off posteriorly, but is much shallower than the preacetabular process.

Character 156: Ilium, opposing postacetabular blades, orientation in dorsal view:

0: subparallel to each other

1: diverge posteriorly from each other

Note (via Brusatte et al., 2014): The "subparallel" condition includes those iliac blades which are oriented medially to contact each other (or nearly contact) above the sacrum (these blades are parallel to each other, but instead of their lateral surfaces being oriented straight laterally they are instead oriented dorsolaterally, hence the convergence above the midline). In some of these taxa, such as the ornithomimosaurs *Gallimimus* and *Garudimimus*, the postacetabular blades diverge from each other when seen in dorsal view, but this is a result of the reorientation of the lateral surface to face dorsolaterally, due to the medial orientation of the two blades to contact each other (or nearly contact) above the acetabulum. Without this medial rotation the postacetabular processes would be parallel or nearly parallel to each other. State 1 refers to the condition in some maniraptorans in which the two iliac blades clearly diverge from each other posteriorly when seen in dorsal view.

Character 157: Ilium, tuber along dorsal edge of ilium dorsal or slightly posterior to acetabulum:

0: absent

1: present

Character 158: Ilium, brevis fossa, form:

0: shallowly inset into bone, brevis shelf projects strongly medially

1: deeply inset into bone, with both lateral lamina and medial brevis shelf curving ventrally to demarcate the deeply concave fossa

Character 159: Ilium, antitrochanter, form:

0: absent or poorly developed

1: prominent

Note (via Brusatte et al., 2014): This character is equivalent to Brusatte et al. (2010a, character 261). In scoring this character, we hold that the large antitrochanters of derived tyrannosauroids, which are extensive flanges that are deeply inset from the remainder of the ischial peduncle, are primarily homologous to the prominent antitrochanters of most other coelurosaurs. Therefore, tyrannosauroids with flange-like antitrochanters and other coelurosaurs with prominent antitrochanters are both scored for state 1.

Character 160: Ilium, ridge bounding cuppedicus fossa, extension:

0: terminates anterior to acetabulum or curves ventrally onto anterior end of pubic peduncle

1: extends far posteriorly and to become confluent or almost confluent with acetabular rim

Character 161: Ilium, cuppedicus fossa, form: (ORDERED)

0: deeply inset into the anterior and lateral surfaces of the pubic peduncle, with a pronounced dorsal rim

1: reduced, fossa shallow or flat, with little or no overhanging dorsal rim

2: absent

Character 162: Ischium, prominent median posterior process along posterior edge of bone:

0: absent, posterior edge of bone straight

1: present

Character 163: Ischium, morphology of shaft distal to the acetabular (obturator) region: (ORDERED)

0: extremely rod-like, midshaft diameter 30-50% midshaft diameter of pubis

1: rod-like, midshaft diameter 60-100% midshaft diameter of pubis

2: wide, flat, and plate-like, midshaft diameter greater than midshaft diameter of pubis

Note (via Brusatte et al., 2014): This is a modified character that subsumes character 280 in Brusatte et al. (2010a). In essence, we here separate the “rod-like” character into two characters: extremely rod-like (in which the ischial shaft is less than 50% of the width of the pubic shaft, seen in some derived tyrannosauroids) and somewhat rod-like (in which the ischial shaft and pubic shaft are approximately the same diameter, seen in many coelurosaurs). We have rescored *Ornitholestes* for state 1 (somewhat rod-like), from Turner et al.’s (2012) polymorphic score for both a rod-like and a plate-like shaft in this taxon.

We also score the tyrannosauroids *Dryptosaurus* and *Alioramus* as polymorphic for states 0 and 1. This is a conservative score, because it is clear that the ischium of these taxa is rod-like but it is unclear how the midshaft diameter compares to the pubic midshaft diameter.

Character 164: Ischium, orientation of shaft: (UNORDERED)

0: straight

1: curved anteriorly at the ventral end

2: hooked posteriorly at the ventral end

Character 165: Ischium, surface morphology of lateral face of ischiadic blade: (UNORDERED)

0: flat or gently rounded

1: concave

2: with longitudinal ridge subdividing lateral surface into anterior (including obturator process) and posterior parts

Character 166: Ischium, obturator process of ischium: (ORDERED)

0: absent

1: proximal in position (midpoint of process positioned at less than 30% of the total length of the ischium)

2: located near middle of ischiadic shaft (midpoint of process positioned at approximately 40-50% of the total length of the ischium)

3: located at distal end of ischium (midpoint of process positioned distal to the midpoint of the ischium)

Note (via Brusatte et al., 2014): The language in this character has been modified following the quantitative character description of Brusatte et al. (2010a, character 281).

Character 167: Ischium, obturator process, contact with pubis:

0: absent

1: present

Character 168: Ischium, obturator foramen or notch in proximal portion of obturator process: (ORDERED)

0: enclosed foramen present

1: open notch present (i.e., a foramen that is no longer completely enclosed)

2: notch or foramen absent

Note (via Brusatte et al., 2014): This character has been modified to take into account the closed obturator foramen of *Guanlong* and *Mirischia*, which although unusual among coelurosaurs and proximal outgroups (allosauroids) is also seen in more distal basal theropod outgroups. Although Naish et al. (2004) described the left and right obturator foramina in *Mirischia* as asymmetrical (one open as a notch, one enclosed), we interpret the supposedly open notch as a closed foramen that has been broken, based on personal observation of the specimen (SMNK 2349 PAL). Turner et al. (2012) scored derived tyrannosauroids for "notch or foramen absent," whereas *Dilong*, compsognathids, and ornithomimosaurs were scored for "open notch present." However, all tyrannosauroids have an open obturator notch that is essentially identical in morphology to those of ornithomimosaurs and compsognathids. Therefore, these taxa are here scored for state 1.

Character 169: Ischium, ischial tubercle ("semicircular scar") ventral to iliac peduncle on the posterior margin of the proximal end of ischium: (ORDERED)

0: absent or potentially homologous structure present as a groove

1: present as a convex bulge on the posterior surface of the ischium

2: present as a rugose, ovoid or triangular flange whose lateral surface is depressed relative to the remainder of the ischium

Note (via Brusatte et al., 2014): This character has been modified, and an additional state added, based on Brusatte et al. (2010a, character 278). We consider the condition in ornithomimosaurs to be equivalent to the "convex bulge" condition of the tyrannosauroid *Juratyrant*, and score more derived tyrannosauroids for a hierarchically nested condition of an especially rugose, flange-like tubercle. We also consider the "groove-like" morphology of this region of the ischium to be homologous, at some level, to the ischial tubercle itself (see Hutchinson 2001). Because it is difficult to distinguish the groove-like condition from true absence of any muscular attachment in this region, we lump together both conditions as state 0 but recognize that future work may be able to subdivide these conditions into separate character states.

Character 170: Ischium, length of bone:

0: greater than two-thirds of pubis length

1: two-thirds or less of pubis length

Character 171: Ischium, morphology of distal ends of opposing ischia: (ORDERED)

0: form symphysis

1: approach one another but do not form symphysis

2: widely separated

Note (via Brusatte et al., 2014): We here score all tyrannosauroids for state 0, contra Turner et al. (2012) who score derived taxa for state 1. As discussed by Brusatte et al. (2012a), the distal ischia of tyrannosauroids do make contact with each other, although this contact is unfused. This is equivalent to the condition in ornithomimosaurs and compsognathids, and we here score all of these taxa for state 0.

Character 172: Ischium, distal end, expansion relative to midshaft:

0: present, resulting in an ischial "boot"

1: absent, ischial "boot" absent

Note (via Brusatte et al., 2014): This character is equivalent to Brusatte et al. (2010a, character 279).

Character 173: Ischium, tubercle on anterior edge of bone:

0: absent

1: present

Character 174: Pubis, orientation of shaft when in articulation: (UNORDERED)

0: projecting anteroventrally (propubic)

1: vertical

2: projecting posteroventrally (opisthopubic)

3: appressed to ischium

Note (via Brusatte et al., 2014): This character subsumes Brusatte et al. (2010a, character 275). We consider this character unordered because there is not a clear transformation sequence or nested set of homologies, although it is possible that future authors may argue for an anteroventral-vertical-posteroventral trend in theropod pelvic evolution.

Character 175: Pubis, pubic boot, anterior projection of boot relative to posterior projection: (ORDERED)

0: equal in size or anterior process larger than posterior process

1: anterior process small, approximately 10-40%

2: anterior process completely absent, posterior process large

3: both anterior and posterior processes absent (i.e., boot present but without distinct anterior and posterior projections)

Note (via Brusatte et al., 2014): This character subsumes Brusatte et al. (2010a, character 273). We have added an additional state here, to distinguish between taxa in which the anterior and posterior projections are equal in size (some derived tyrannosaurids) and those taxa in which anterior projections are present but are approximately 10-40% of the length of the posterior projection (many other coelurosaurs). Using our character scoring scheme, compsognathids and various paravians are scored for state 2, which refers to the complete lack of an anterior process and a pubic boot that is often “hook-like” in its morphology.

Character 176: Pubis, shelf on shaft proximal to symphysis (‘pubic apron’): (UNORDERED)

0: extends medially from middle of shaft

1: shelf extends medially from anterior edge of shaft

2: strongly reduced (restricted to distal end of pubis) or absent

Note (via Brusatte et al., 2014): Turner et al. (2012) scored tyrannosauroids (including *Dilong* and tyrannosaurids) for state 0, but we here rescore all score-able tyrannosauroids for state 1, based on personal observation of specimens. In all known tyrannosauroid pubes the pubic apron arises from the anterior margin of the shaft, not the middle of the shaft as in outgroups like *Allosaurus* (Madsen 1976). We have also removed the language in the original TWiG character relating to the cylindrical or anteroposteriorly flattened nature of the shafts, as these conditions do not correspond 1-to-1 with the location of the apron. Future authors may wish to reintroduce a character relating to the morphology of the shaft (cylindrical vs. flattened), but we could not identify clear, distinct categories of variation among taxa and hence do not utilize such a character here.

Character 177: Pubis, curvature of shaft: (UNORDERED)

0: absent, shaft straight

1: distal end curves anteriorly, anterior surface of shaft concave

2: distal end curves posteriorly, anterior surface of shaft convex

Note (via Brusatte et al., 2014): This character subsumes character 269 of Brusatte et al. (2010a). Derived tyrannosauroids are here scored for state 1, a concave anterior margin of the pubic shaft, based on Brusatte et al. (2010a).

Character 178: Pubis, length of pubic apron: (ORDERED)

0: about half of pubic shaft length

1: less than 1/3 of shaft length

2: greatly reduced, restricted to far distal end of pubis

3: absent

Note (via Brusatte et al., 2014): We modified this character to make it into an additive multistate referring to the presence/absence and size of the pubic apron. For all other pubic apron characters, those taxa without an apron are scored as inapplicable ("?").

Character 179: Pubic apron, form of contact between opposing pubes distally: (ORDERED)

0: both pubes meet extensively

1: contact between pubes disrupted by a slit (pubic foramen)

2: no contact between pubes distally, pubic apron absent in this part of pubis but present further proximally

Note (via Brusatte et al., 2014): The pubic fenestra is usually considered a synapomorphy of Tetanurae (Rauhut 2003; Benson et al. 2009). Turner et al. (2012) scored *Dilong* for state 0, but personal observation of the specimen indicates that the distal portion of the conjoined pubes is too damaged (and not complete enough) to permit observation of the fenestra. Therefore, it is here scored "?". Turner et al. (2012) also scored *Haplocheirus* for state 0, which would be unexpected, but because we cannot confirm this with a personal observation we conservatively score it as "?".

Character 180: Femur, head, fovea capitalis for attachment of capital ligament:

0: absent or subtle

1: present as a distinct circular fovea located in center of the medial surface of the head

Character 181: Femur, interaction of lesser and greater trochanters: (ORDERED)

0: separated from each other by deep cleft

1: separated from each other by small groove

2: completely fused to each other (absent as distinct structures), forming a trochanteric crest

Character 182: Femur, lesser trochanter, shape in lateral view:

0: alariform, projects anteriorly as a broad flange that is anteroposteriorly wider than the greater trochanter

1: reduced to approximately the same anteroposterior width as the greater trochanter, cylindrical in cross section

Character 183: Femur, ridge on lateral surface distal to lesser and greater trochanters (homologous to the trochanteric shelf):

0: absent or represented only by faint rugosity or bulge

1: distinctly raised from shaft as a pronounced, mound-like ridge

Character 184: Femur, fourth trochanter:

0: present

1: absent (or reduced to a subtle and barely distinguishable margin)

Character 185: Femur, accessory trochanteric crest distal to lesser trochanter:

0: absent

1: present

Note (via Brusatte et al., 2014): We consider the "accessory trochanteric crest" (identified by Makovicky and Sues [1998] in *Microvenator*) as equivalent to the accessory trochanter that is present in some tetanurans (including some coelurosaurs: e.g., Brusatte et al. 2008c), as both structures are accessory flanges or ridges along the anterior margin of the distl portion of the lesser trochanter. Therefore, this character is equivalent to Brusatte et al. (2010a, character 287). Accessory trochanters are large and prominent in basal tyrannosauroids (*Guanlong*, *Xiongguanlong*) but are lost in more derived tyrannosauroids. They are also large and prominent in several basal coelurosaurs (e.g., ornithomimosaurs, *Mirischia*, *Zuolong*, *Coelurus*, *Tanycolagreus*).

Character 186: Femur, mesiodistal crest, form:

0: absent or present as a subtle structure

1: present as a pronounced longitudinal crest extending proximally from medial condyle, which is seen to strongly overhang the remainder of the medial margin of the femur in posterior view (i.e., is visible as a broad flange in posterior view

Note (via Brusatte et al., 2014): We have reworded this character to make it refer specifically to the pronounced mesiodistal crest of some coelurosaurs. This crest is either located on the anterior surface of the femur (as described in the original TWiG character) or along the anteromedial corner of the femur, but in all cases extends proximally from the medial condyle.

Character 187: Femur, flexor groove, morphology of distal end:

0: open distally, smoothly confluent with distal articular surface

1: closed off distally by contact between distal condyles, separated from distal articular surface

Character 188: Fibula, distal extent:

0: reaches proximal tarsals

1: short, tapering distally, and not in contact with proximal tarsals

Character 189: Fibula, medial margin of proximal end in proximal view:

0: concave

1: flat

Character 190: Fibula, deep oval fossa, with well defined margins, on medial surface near proximal end:

0: absent

1: present

Note (via Brusatte et al., 2014): This character refers specifically to the discrete, ovoid fossa of some tyrannosauroids and ornithomimosaurs, as well as outgroup taxa. Some other basal coelurosaurs such as *Coelurus* and *Zuolong* have a depressed groove excavating much of the medial surface of the proximal fibula, but this is not present as a discrete, indented fossa with well defined margins (instead, the groove has a well defined anterior margin but opens posteriorly). The outgroups are scored for state 0, because they possess an open fossa (without a distinct posterior margin) as in most coelurosaurs.

Character 191: Astragalus and calcaneum, morphology of distal condyles:

0: condyles separated by shallow, indefinite sulcus

1: condyles separated by prominent tendoneal groove on anterior surface

Character 192: Tibia, number of cnemial crests:

0: single pronounced crest

1: two crests (main crest with accessory anterior crest)

Character 193: Astragalus, ascending process, form: (UNORDERED)

0: tall and broad, covering most of anterior surface of distal end of tibia (70% or more of mediolateral width of surface) and dorsoventral height greater than twice the height of the main body of the astragalus

1: process short and slender, covering only lateral half of anterior surface of tibia (includes derived therizinosauroid condition in which a lateral extension of the ascending process contacts the fibula) and dorsoventral height less than twice the height of the main body of the astragalus

2: ascending process tall, but with medial notch that restricts it to lateral side of anterior face of distal tibia

Note (via Brusatte et al., 2014): This character includes information on both the dorsoventral height and mediolateral width of the ascending process. Sometimes these measurements are given separate characters, but we here hold that they are generally correlated, in the sense that taxa with a very tall ascending process always have a mediolaterally broad process as well, and taxa such as *Guanlong* and *Falcarius* with a short ascending process also have a mediolaterally restricted process. We recognize, however, that future authors may want to break this character into separate characters referring to length and width, but we hesitate to do so because we do not wish to introduce a second character that is likely correlated with the first.

The ascending processes of compsognathids have been described as somewhat reduced, in that they only cover approximately 70% of the anterior surface of the tibia (Ostrom 1978; Currie and Chen 2001; Hwang et al. 2004). We consider this condition equivalent to state 0 here, but recognize that future authors may want to subdivide state 0 into separate characters for a broad ascending process that covers most of the anterior surface of the tibia and a reduced process that covers approximately 70% of the surface. These could be associated with state 1 here (a process covering half the tibial surface) to create an

ordered multistate, and state 2 here (which is unique to some alvarezsaurids) could be made into its own character. We do not do this, however, because we hesitate to divide state 0 into additional states because the "reduced" ascending processes of compsognathids are all based on slab specimens, which are difficult to measure and often prone to misinterpretation. It is clear, however, that these compsognathids do not possess the reduced and laterally restricted ascending processes of some basal coelurosaurs (e.g., *Guanlong*, *Coelurus*, *Tugulusaurus*) and the ougroups (*Allosaurus*, *Sinraptor*), which are denoted by state 1. This character is an equivalent, but expanded, version of Brusatte et al. (2010a, character 297).

Character 194: Astragalus, ascending process, articulation with condyles distally:

0: confluent with condylar region of astragalus

1: separated from condylar region by transverse groove or fossa across base

Character 195: Astragalus and calcaneum, fusion: (ORDERED)

0: absent, astragalus and calcaneum unfused to each other or to tibia in adults

1: present, astragalus and calcaneum fused to each other, unfused to tibia

2: present, astragalus and calcaneum completely fused to each other and to tibia

Character 196: Distal tarsals, fusion with metatarsals:

0: absent, distal tarsals and metatarsals separate

1: present, distal tarsals and metatarsals fused into tarsometatarsus

Character 197: Metatarsals, fusion: (ORDERED)

0: absent, metatarsals not co-ossified

1: present, metatarsals co-ossified proximally

2: present, metatarsals co-ossified proximally and distally

3: present, metatarsals co-ossified proximally and distally, with distal fusion extreme and distal vascular foramen closed

Character 198: Metatarsal II, morphology of distal end:

0: smooth, not ginglymoid

1: with developed ginglymus that extends onto extensor surface, giving the distal end a strongly concave profile in extensor (anterior) view

Character 199: Metatarsal III, morphology of distal end:

0: smooth, not ginglymoid

1: with developed ginglymus: smooth articular region extends proximally onto extensor surface and is broadly exposed

Note (via Brusatte et al., 2014): We here consider the condition in some basal coelurosaurs (*Zuolong*, *Tugulusaurus*, *Dilong*, *Guanlong*) to represent a well developed ginglymus, as defined for character state 1. Xu et al. (2004) recognized that *Dilong* was unusual among tyrannosauroids in possessing a gingylmus on metatarsal III (although it is absent on metatarsal II), a character score that was followed by Turner et al. (2012). Later discoveries showed that this character state is also present in some other basal coelurosaurs: Rauhut and Xu (2005) later noted that one was present on *Tugulusaurus* and Choiniere et al. (2010b) described and figured it for *Zuolong*. Our personal observations of *Guanlong* show that it is present in this taxon as well. *Bicentenaria* is described by Novas et al. (2012) as lacking a ginglymoid distal end of metatarsal III, but we score this taxon here as "?" because we have not been able to observe it, and therefore cannot assess if it has the basal coelurosaur-style ginglymoid condition of *Guanlong*, *Tugulusaurus*, and *Zuolong*.

Character 200: Metatarsal III, exposure of proximal shaft in extensor view: (ORDERED)

0: prominently exposed between MT II and MT IV along entire metapodium

1: MT III proximal shaft constricted and much narrower than either II or IV, but still exposed along most of metapodium, subarctometatarsal

2: very pinched, not exposed along proximal section of metapodium, arctometatarsal

3: proximal part of MT III lost entirely

Note (via Brusatte et al., 2014): We here consider this character ordered, because we hypothesize nested homology between the subarctometatarsal-arctometatarsal-mt III lost states (i.e., these form an ordered, progressive sequence of metatarsal III pinching and eventual loss). This character is equivalent to Brusatte et al. (2010a, Character 299).

Character 201: Pedal digit II, ungual and penultimate phalanx, morphology:

0: similar to those of III

1: penultimate phalanx highly modified for extreme hyper-extension, ungual more strongly curved and significantly larger than that of digit III

Character 202: Metatarsal II, site of articular surface for metatarsal I: (UNORDERED)

0: the middle of the medial surface of metatarsal II

1: the posterior surface of distal quarter

2: the medial surface near the proximal end

3: the medial surface at or near the distal end

Character 203: Metatarsal I, form of proximal end:

0: attenuates proximally, articular surface for metatarsal II is a simple butt joint lying against the shaft of metatarsal II

1: large and robust, similar to those of metatarsals II-IV

Note (via Brusatte et al., 2014): Although character states could probably be inferred by the form and position of the metatarsal I articular scar on metatarsal II, we conservatively score taxa for an affirmative score only if we can observe metatarsal I itself.

Character 204: Metatarsal IV, shaft thickness:

0: round or thicker dorsoventrally (extensor-flexor direction) than wide mediolaterally in cross section

1: mediolaterally widened and flat in cross section

Character 205: Foot, symmetry:

0: symmetrical

1: asymmetrical with slender MTII and very robust MT IV, excluding flange

Character 206: Dorsal vertebrae, neural spines on posterior dorsals, shape in lateral view:

0: rectangular or square

1: fan-shaped, anteroposterior length of spine expanding dorsally

Note (via Brusatte et al., 2014): State 1 refers specifically to the fan-shaped neural spines of compsognathids and *Pelecanimimus*, whose dorsal extremity is substantially longer anteroposteriorly than is the base of the spine. This is due to a “funneling outwards) in anteroposterior length dorsally across the length of the spine. Some theropods (e.g., *Tanycolagreus*, some ornithomimosaurs in intermediate phylogenetic position between *Pelecanimimus* and derived taxa with rectangular neural spines) have some

dorsal vertebrae with neural spines that are moderately expanded at their dorsal tips, but these do not exhibit the striking fan shape of compsognathids, and are thus scored for state 0.

Character 207: Manual phalanx I-1, shaft diameter:

0: less than or approximately equal to the shaft diameter of radius.

1: greater than the shaft diameter of radius.

Note (via Brusatte et al., 2014): State 1 of this character refers to a phalanx that is substantially thicker in diameter than the radius. Several coelurosaurs (e.g., *Tanycolagreus*, tyrannosauroids, ornithomimosaurs) have a phalanx I-1 that is approximately the same diameter of the radius, and sometimes is slightly larger (~10%). These taxa are scored for state 0, however, because this condition differs from the condition in compsognathids in which the phalanx is dramatically larger than the radius (state 1).

Character 208: Angular, extent in lateral view:

0: widely exposed, suture between surangular and angular reaches or nearly reaches posterior end of mandible

1: reduced in exposure, suture between surangular and angular does not reach posterior end of the mandible

Character 209: Surangular, laterally inclined flange along dorsal edge of bone for articulation with the lateral process of lateral quadrate condyle:

0: absent

1: present

Character 210: Distal articular ends of metacarpals I + II: (UNORDERED)

0: ginglymoid

1: rounded, smooth

2: II ginglymoid and MC I shelf

Character 211: Radius and ulna, articulation with each other:

0: well separated

1: with distinct adherence or syndesmosis distally

Character 212: Upper and lower jaws, occlusion:

0: occlude for their full length

1: diverge anteriorly due to kink and downward deflection of the dentary (pronounced downward deflection of dentary buccal margin anteriorly)

Character 213: Quadrate, exposure of head in lateral view:

0: absent, covered by squamosal laterally

1: present, exposed because quadrate cotyle of squamosal opens laterally due to a wide notch between the ventral and posterior processes

Character 214: Ilium, brevis fossa, orientation and exposure in lateral view:

0: faces primarily ventrally, but it is widely visible in lateral view (especially anteriorly)

1: faces primarily ventrally and medially, and it is obscured in lateral view across its entire length by a well developed lateral lamina of the postacetabular process of the ilium

Note (via Brusatte et al., 2014): This character is equivalent to character 310 in the revised version of the Brusatte et al. (2010a) analysis published by Brusatte and Benson (2013). We have altered the wording here to reflect the character language of Brusatte and Benson (2013), which focuses specifically on whether the fossa is visible or obscured in lateral view.

Character 215: Lesser trochanter, vertical ridge on lateral surface:

0: present

1: absent

Character 216: Supratemporal fossa, extension onto the squamosal:

0: absent, fenestra bounded laterally and posteriorly by the squamosal, with no distinct fossa present on the dorsal surface of the squamosal

1: present, distinct fossa extends onto the dorsal surface of the squamosal

Character 217: Dentary, teeth: (ORDERED)

0: present across much of bone, dentary fully toothed

1: present but only at the far anterior tip

2: absent, dentary edentulous

Character 218: Coracoid, ventral (=anterior) margin of bone anterior (=ventral) to glenoid, form:

0: straight or shallowly indented by a notch

1: deeply indented by a pronounced notch separating the glenoid and postglenoid process, glenoid lip everted

Note (via Brusatte et al., 2014): State 1 refers to the deep notch of ornithomimosaurs, which separates the glenoid from a pronounced and hook-shaped postglenoid process anteriorly. Many other coelurosaurs have a shallow notch in this region, but only ornithomimosaurs have a discrete, deep notch, which helps to define the characteristic hook-like postglenoid process in these taxa.

Character 219: Articular, retroarticular process, orientation:

0: points posteriorly

1: curves gently posterodorsally

Character 220: Scapula, flange on supraglenoid buttress:

0: absent

1: present

Character 221: Laterosphenoid, depression (possibly pneumatic) on ventral surface of postorbital process of bone:

0: absent

1: present

Character 222: Braincase, basal tubera, mediolateral width of conjoined tubera relative to occipital condyle: (ORDERED)

0: as wide as or wider than the occipital condyle

1: narrower than occipital condyle, reduced to a set of small processes directly below the condyle and separated by a narrow notch

2: tubera absent

Note (via Brusatte et al., 2014): This character is not equivalent to characters 154 and 155 in Brusatte et al. (2010a), which refer to the dorsoventral depth of the basal tubera and the depth of the notch between the tubera, respectively. This character refers solely to the mediolateral width of the tubera, and is considered an ordered character because we hypothesize a nested set of homologies (large-small-absent). The highly modified basal tubera of derived therizinosauroids are here regarded as possessing state 0 and

not state 2 (absent), as remnants of the tubera are still present despite their incorporation into the basisphenoidal bulla.

Character 223: Ilium, postacetabular process, morphology of dorsal edge:

0: convex or straight

1: deeply concave, ventral portion of postacetabular process (housing brevis shelf medially) extending posterior to dorsal portion of the postacetabular process as a tab-like structure

Note (via Brusatte et al., 2014): State 1 refers to the condition in some dromaeosaurids (e.g., *Unenlagia*), in which the dorsal margin of the postacetabular process is deeply concave, due to the extension of the ventral portion of the postacetabular process posteriorly as a discrete, rectangular, tab-like structure. This tab-like structure is ornamented by the brevis shelf medially, and therefore houses an extension of the brevis fossa itself. Some ornithomimosaurs have a subtly concave dorsal border of the postacetabular process, but this is not considered equivalent to the marked condition of some dromaeosaurids.

Character 224: Ilium, posterior end of postacetabular process, morphology in dorsal view:

0: terminating in rounded or square end, due to a brevis shelf that extends no farther than the posterior margin of the lateral lamina (lateral surface) of the postacetabular process

1: with lobate brevis shelf projecting beyond posterior end of lateral lamina of postacetabular process

Character 225: Pedal phalanx II-2, flexor heel, form:

0: small and asymmetrically developed only on medial side of vertical ridge subdividing proximal articulation

1: heel long and lobate, with extension of midline ridge extending onto its dorsal surface

Note (via Brusatte et al., 2014): Taxa without a discrete flexor heel are scored as inapplicable (“?”).

Character 226: Metatarsal IV, large, longitudinal flange along posterior or lateral surface of bone:

0: absent

1: present

Character 227: Ischium, proximodorsal process, form:

0: small, tab-like or pointed process along posterior edge of ischium

1: large, proximodorsally hooked, and separated from iliac peduncle of the ischium by a notch

Note (via Brusatte et al., 2014): Taxa without a discrete proximodorsal process are scored as inapplicable ("?").

Character 228: Pubis, prominent tubercle on lateral surface of shaft, at approximately the midpoint of the shaft:

0: absent, shaft smooth

1: present

Character 229: Ischium, distally placed dorsal process along posterior edge of shaft:

0: absent

1: present

Character 230: Ischium, obturator process, shape:

0: tab-like, with distal end separated from shaft by a discrete notch

1: triangular, with distal end confluent with shaft

Character 231: Ischium, morphology of triangular obturator process (only in those taxa with a triangular process as defined in character 233):

0: longer proximodistally than wide anteroposteriorly at the center of the process

1: shorter proximodistally than wide anteroposteriorly at the center of the process, resembles an elongate triangle extending anteriorly

Character 232: Metatarsal II, tuber along extensor surface (associated with the insertion of the tendon of the m. tibialis cranialis in Aves): (ORDERED)

0: absent

1: present, on approximately the center of the proximodorsal surface of metatarsal II

2: present, developed on lateral surface of metatarsal II, at contact with metatarsal III or on lateral edge of metatarsal III

Character 233: Ulna: femur length ratio:

0: substantially less than one

1: equal to or greater than one

Character 234: Maxilla, position of maxillary fenestra relative to ventral margin of antorbital fossa: (ORDERED)

0: abuts ventral margin

1: dorsal to ventral margin, at approximately the dorsoventral midpoint of the antorbital fossa

2: far dorsal to the ventral margin, dorsal to the dorsoventral midpoint of the antorbital fossa

Note (via Brusatte et al., 2014): This character has been modified to take into account variation in the position of the maxillary fenestra in tyrannosauroids, as noted by Brusatte et al. (2010a, character 18). State 0 here refers to the condition in *Daspletosaurus*, *Tarbosaurus*, and *Tyrannosaurus*, in which the maxillary fenestra abuts the ventral margin of the antorbital fossa.

Character 235: Maxilla, dorsoventral depth of main body of bone (=jugal process) at the midpoint of the antorbital fenestra compared to the depth of the entire skull at this measuring point: (ORDERED)

0: shallow, less than 16% of the depth of the entire skull

1: between 16-22% of skull depth

2: deep, greater than 22% of skull depth

Note (via Brusatte et al., 2014): This character has been modified into an ordered multistate to take into account additional variation noted by Brusatte et al. (2010a, character 23).

Character 236: Maxilla, maxillary fenestra, recessed within a shallow, posteriorly or posterodorsally open fossa, which is itself located within the maxillary antorbital fossa:

0: absent

1: present

Character 237: Maxilla, ascending process (=dorsal ramus or nasal process), form:

0: prominent, exposed medially and laterally

1: absent or reduced to a process with slight medial and no lateral exposure

Character 238: Maxilla, participation of the ventral ramus of the nasal process (=ascending process) in the anterior margin of the antorbital fenestra (as seen in lateral view): (ORDERED)

0: present extensively

1: small dorsal projection of the maxilla participates in the anterior margin

2: no dorsal projection of maxilla participates in the anterior margin

Character 239: Antorbital fenestra, composition of the dorsal border (as seen in lateral view):

0: lacrimal and maxilla

1: lacrimal and nasal

Character 240: Antorbital fossa, composition of the dorsal border (as seen in lateral view): (UNORDERED)

0: lacrimal and maxilla

1: lacrimal and nasal

2: maxilla, premaxilla, and lacrimal

Character 241: Maxilla, lateral lamina of the ascending ramus:

0: present and broadly exposed in lateral view

1: present but reduced to small triangular exposure in lateral view

Character 242: Frontal, supratemporal fossa, anteroposterior length compared to overall length of exposed portion of frontal on skull roof: (ORDERED)

0: less than 30%

1: between 30-60%

2: greater than 60%

Note (via Brusatte et al., 2014): This character has been modified and transformed into an ordered multistate using the quantitative character descriptions of Brusatte et al. (2010a, character 115). As worded here, this character now refers solely to the extent of the supratemporal fossa onto the frontal, unlike the previous TWiG character that referred to the qualitative extent (minor, extensive) of the fossa onto the frontal and postorbital. We note, however, that taxa with an extensive fossa on the frontal usually also have an extensive fossa on the dorsal surface of the postorbital.

Character 243: Jugal, contribution to antorbital fenestra margin (in lateral view):

0: absent

1: present

Character 244: Maxillary and dentary teeth, size of mesial (=anterior) and distal (=posterior) denticles:

0: not substantially different in size

1: mesial denticles, when present, substantially smaller than distal denticles

Character 245: Maxillary teeth, orientation relative to the long axis of the lower jaw:

0: almost perpendicular to jaw margin

1: inclined strongly posteroventrally

Character 246: Maxillary teeth, apicobasal height of teeth along tooth row:

0: highly variable with gaps evident for replacement

1: almost isodont with no replacement gaps

Character 247: Splenial, posterior margin (anterior margin of internal mandibular fenestra):

0: smooth

1: with distinct notch

Character 248: Premaxillary teeth, cross sectional size of first tooth crown compared with crowns of premaxillary teeth 2 and 3: (UNORDERED)

0: slightly smaller or same size

1: much smaller

2: much larger

Character 249: Maxilla, promaxillary fenestra in adults, visibility in lateral view:

0: visible (either as complete fenestra or portion of the fenestra)

1: completely obscured by ascending ramus of maxilla

Note (via Brusatte et al., 2014): This character is equivalent to character 15 in Brusatte et al. (2010).

Character 250: Nasal, shape of dorsal surface: (ORDERED)

0: flat or slightly convex

1: convex (vaulted) anteriorly, above and immediately posterior to the external naris

2: vaulted across most of their length

Note (via Brusatte et al., 2014): This character has been modified following Brusatte et al. (2010, character 38). Tyrannosauroids with a midline nasal crest are scored as inapplicable (“?”).

Character 251: Nasal, fusion between left and right nasals:

0: absent

1: present

Character 252: Quadratojugal and squamosal, constriction of lateral temporal fenestra: (ORDERED)

0: absent, anterior margins of both bones are approximately vertical

1: present, convex kink along the suture between the two bones that projects into the fenestra, constricting it to approximately one half or less of its maximum anteroposterior length

2: present, dorsal region of quadratojugal moderately expanded anteroposteriorly relative to the remainder of the bone, constricting fenestra to approximately one half of its maximum anteroposterior length

3: present, dorsal region of quadratojugal expanded anteroposteriorly by at least twice the minimum anteroposterior dimension of the bone, forming a flange that meets the ventral ramus of the squamosal to nearly divide the fenestra

Note (via Brusatte et al., 2014): This character is expanded into an ordered multistate using the character state definitions of Brusatte et al. (2010a, character 98).

Character 253: Supraoccipital, pronounced and strongly demarcated median ridge on posterior (occipital) surface:

0: absent

1: present

Character 254: Surangular, anteroventral extension divides external mandibular fenestra by contacting angular anteriorly

0: absent

1: present

Character 255: Ilium, vertical ridge on lateral surface of iliac blade above acetabulum: (ORDERED)

0: absent or poorly developed

1: present as a well-developed, linear structure extending vertically or anterodorsally

2: present as a well-developed, linear structure extending posterodorsally

Note (via Brusatte et al., 2014): This character is modified into an ordered multistate to take into account variation within tyrannosauroids as noted by Brusatte et al. (2010a, character 258).

Character 256: Premaxilla, main body, dorsoventral depth compared to anteroposterior length: (ORDERED)

0: less than or equal to

1: between 1.0-1.9 times larger

2: greater than 2 times larger

Note (via Brusatte et al., 2014): This character is modified into an ordered multistate to take into account variation within tyrannosauroids as noted by Brusatte et al. (2010a, character 8).

Character 257: Nasal, external texture of mid section of bone:

0: smooth to slightly rugose

1: pronounced rugosities and accessory vascular foramina present

Note (via Brusatte et al., 2014): This character is equivalent to Brusatte et al. (2010a, character 40).

Character 258: Jugal, shape of anterior process underneath the lacrimal: (UNORDERED)

0: tapering

1: bluntly squared anteriorly

2: expanded

3: bifurcated

Character 259: Axis, neural spine, morphology in lateral view:

0: extensive and anteroposteriorly elongate, sheet-like

1: anteroposteriorly reduced, rod-like

Character 260: Cervical vertebrae, prezygapophyses in anterior postaxial cervicals, orientation:

0: straight across their lengths

1: anteroposteriorly convex, flexed ventrally anteriorly

Character 261: Dorsal vertebrae, extent of pneumaticity (pneumatic foramina on lateral surfaces): (ORDERED)

0: foramina absent

1: foramina present in anterior dorsals

2: present in all dorsals

Note (via Brusatte et al., 2014): This character is equivalent to Brusatte et al. (2010, character 218).

Character 262: Femur/humerus length ratio: (ORDERED)

0: more than 3.3

1: between 3.3-2.5

2: between 1.2 and 2.5

3: less than 1

Note (via Brusatte et al., 2014): This character has been modified to include additional variation within tyrannosauroids, following Brusatte et al. (2010a, character 239).

Character 263: Humerus, shape in lateral view:

0: sigmoidal

1: straight

Note (via Brusatte et al., 2014): We consider this character to be equivalent to character 242 in Brusatte et al. (2010a), which scores for the presence/absence of humeral shaft rotation (quantified by the angle between the long axes of the proximal and distal ends). Brusatte et al. (2010a) scored *Dryptosaurus* for such rotation, but this taxon has a straight humerus in lateral view and is thus scored for state 1 here. It is possible that there is a subtle amount of variation within this character, such that taxa like *Dryptosaurus* have a straight humerus in lateral view but still retain some degree of rotation (a 30-45 degree angle between the long axes of the proximal and distal ends). However, we do not want to subdivide this character too finely, because it makes recognizing homologies across a wide swath of taxa more difficult. Therefore, we retain a simple binary character here.

Character 264: Radius, length compared to that of humerus:

0: more than half the length of humerus

1: less than half the length of humerus

Character 265: Premaxilla, fusion of opposing premaxillae at the symphysis: (ORDERED)

0: unfused in adults

1: fused anteriorly in adults, posterior nasal [frontal] processes not fused to each other

2: fused anteriorly and opposing frontal processes completely fused

Character 266: Dentary, form of articulation between opposing dentaries at the symphysis:

0: joined proximally by ligaments

1: joined by bone

Character 267: Dentary, symphysis region, two strong grooves forming an anteriorly opening 'v' in ventral view:

0: absent

1: present

Character 268: Cranium, facial margin, composition: (ORDERED)

0: primarily formed by the maxilla, with the maxillary process of the premaxilla restricted to the anterior tip

1: maxillary process of the premaxilla extending along half of facial margin

2: maxillary process of the premaxilla extending more than half of facial margin

Character 269: Premaxilla, nasal (frontal) process, length:

0: short

1: long, closely approaching frontal

Character 270: External naris, size compared to the antorbital fenestra: (ORDERED)

0: considerably smaller

1: long axis approximately same length as long axis of antorbital fenestra

2: larger

Note (via Brusatte et al., 2014): This character has been expanded into an ordered multistate to take into account variation within tyrannosauroids noted by Brusatte et al. (2010a, character 4). *Sinotyrannus* is here scored for state 1; although the antorbital fenestra is not preserved in this taxon, state 1 is hypothesized because the external naris long axis is as long as the first seven teeth of the maxillary tooth row, similar to the proportions in *Guanlong* and *Proceratosaurus*, which are scored for state 1, but differing from the state in other tyrannosauroids (and most other coelurosaurs) in which the naris is no longer than the first 4-5 teeth.

Character 271: Ectopterygoid:

0: present

1: absent

Character 272: Vomer and pterygoid, form of articulation between bones: (UNORDERED)

0: present, well developed

1: reduced, narrow process of pterygoid passes dorsally over palatine to contact vomer

2: absent, pterygoid and vomer do not contact

Note (via Brusatte et al., 2014): This character is left unordered because it is unclear if states 1 and 2 form a nested set of homologies.

Character 273: Palatine and pterygoid, form of articulation between bones:

0: long, anteroposteriorly overlapping contact

1: short, primarily dorsoventral contact

Character 274: Palatine, contacts with bones of the facial region:

0: contacts maxillae only

1: contacts premaxillae and maxillae

Character 275: Vomer, contact with premaxilla:

0: present

1: absent

Character 276: Basisphenoid, projecting articulation with pterygoid via discrete basipterygoid processes:

0: present

1: absent

Character 277: Basisphenoid, location of pterygoid articular surface:

0: located basal on basisphenoid

1: located markedly anterior on basisphenoid (parasphenoid rostrum) such that the articulations are subadjacent on the narrow rostrum

Character 278: Basisphenoid and pterygoid articulation, orientation of contact: (UNORDERED)

0: anteroventral

1: mediolateral

2: entirely dorsoventral

Character 279: Pterygoid, articular surface for basisphenoid, morphology: (ORDERED)

0: concave ‘socket’ or short groove enclosed by dorsal and ventral flanges

1: flat to convex

2: flat to convex facet, stalked, variably projected

Character 280: Pterygoid, shape of bone:

0: kinked, surface for basisphenoid articulation at high angle to axis of palatal process of pterygoid

1: straight, basisphenoid articulation in line with axis of palatal process

Character 281: Ossified interorbital septum, sphenethmoid:

0: absent

1: present

Note (via Brusatte et al., 2014): This character is equivalent to Brusatte et al. (2010a, character 167), which recognizes the presence of an ossified mesethmoid and sphenethmoid in derived tyrannosauroids. In the absence of developmental data, it is not clear if the ossification referred to as the sphenethmoid in tyrannosauroids is homologous to that in birds. However, we retain a single character scoring for the presence/absence of an ossified sphenethmoid for simplicity. The Brusatte et al. (2010a) character refers to both an ossified sphenethmoid and mesethmoid, but here we consider only the sphenethmoid in defining this character, so that it is equivalent to the original character in Turner et al. (2012). All known tyrannosauroids with an ossisfied sphenethmoid also possess an ossified mesethmoid, so their presence is probably correlated.

Character 282: Ossified interorbital septum, mesethmoid:

0: unossified or small, restricted to the ventral surface of the frontal or marginally extends anteriorly past the premaxillae/frontal contact but does not surpass posterior edge of external nares

1: large, extends anterior to the posterior extent of the frontal processes of premaxillae and anterior to the posterior edge of the external nares

Character 283: Eustachian tubes, morphology: (UNORDERED)

0: paired and lateral

1: paired, close to cranial midline

2: paired and adjacent on midline or single anterior opening

Character 284: Eustachian tubes, ossification:

0: absent

1: present

Character 285: Squamosal, ventral (=zygomatic) process, morphology:

0: large and elongate, dorsally encloses otic process of the quadrate and extends anteroventrally along shaft of quadrate, dorsal head of quadrate not visible in lateral view

1: short, head of quadrate exposed in lateral view

Note (via Brusatte et al., 2014): This character is not redundant with Turner et al. (2012, character 216), because this character refers specifically to the size of the ventral process. A reduced ventral process is one means of exposing the quadrate head in lateral view, as is a wide notch between the ventral and posterior processes (which is what is scored in character 216).

Character 286: Quadrate, orbital process (=pterygoid flange) of quadrate, form of articulation with pterygoid:

0: pterygoid broadly overlapping medial surface of orbital process (i.e., 'pterygoid ramus' present)

1: pterygoid contact restricted to anteromedial edge of orbital process

Character 287: Quadrate, form of pterygoid articulation with orbital process in those taxa where the contact is restricted to the anteromedial edge of the process: (ORDERED)

0: pterygoid articulates with anterior-most tip

1: pterygoid articulation does not reach tip

2: pterygoid articulation with no extent up orbital process, restricted to quadrate corpus

Note (via Brusatte et al., 2014): All taxa scored for state 0 in the previous character are here scored as inapplicable ("?").

Character 288: Quadrate/pterygoid contact, morphology in those taxa where the contact is restricted to the anteromedial edge of the orbital process:

0: as a facet, variably with slight anteromedial projection cradling base

1: condylar, with a well-projected tubercle on the quadrate

Note (via Brusatte et al., 2014): All taxa scored for state 0 for character 286 are here scored as inapplicable (“?”).

Character 289: Quadrate, well-developed tubercle on anterior surface of dorsal process:

0: absent

1: present

Character 290: Quadrate, form of articulation with quadratojugal:

0: overlapping

1: peg and socket articulation

Character 291: Quadrate, dorsal process, articulation with other bones:

0: with squamosal only

1: with squamosal and prootic

Character 292: Quadrate, dorsal process, development of intercotylar incisure between prootic and squamosal cotylae: (ORDERED)

0: absent, articular surfaces not differentiated (usually meaning prootic contact was absent)

1: two distinct articular facets, incisure not developed

2: incisure present, ‘double headed’

Character 293: Quadrate, mandibular articulation, form:

0: divided into two condyles

1: divided into three condyles, due to additional posterior condyle or broad surface

Character 294: Quadrate, pneumaticity:

0: absent

1: present

Note (via Brusatte et al., 2014): This character scores specifically for the presence or absence of an internal pneumatic recess in the quadrate. Many coelurosaurs have an internally pneumatic quadrate, but this recess communicates with the external surface of the bone in two distinct ways (Tahara and Larsson 2011). Most coeluorsaurs have a small foramen, usually on the posterior surface of the quadrate, that leads into the recess. Derived tyrannosauroids, on the other hand, possess a large, deep, funnel-like pneumatic opening on the anterior surface of the quadrate where the pterygoid wing and mandibular condyles meet.

Both conditions are considered homologous at the level of absence/presence of quadrate pneumaticity here, but we utilize separate characters below for the absence/presence of the posterior foramen and the absence/presence of the anterior funnel. The homologous nature of both conditions is supported by the morphology of *Dilong*, which clearly has an anterior funnel and also a posterior foramen (IVPP V14243; Xu et al. 2004).

Character 295: Quadrate, cluster of pneumatic foramina on the posterior surface of the tip of the dorsal process:

0: absent

1: present

Note (via Brusatte et al., 2014): Because we are uncertain whether this condition may be homologous to the broader condition of quadrate pneumaticity, we score taxa lacking this character for state 0, even if they are also scored for state 0 (absence) for the general character of quadrate pneumaticity.

Character 296: Quadrate, form of pneumatization, large single pneumatic foramen on the posterior or posteromedial surface of the shaft:

0: absent

1: present

Note (via Brusatte et al., 2014): See discussion regarding quadrate pneumaticity above. All taxa without quadrate pneumaticity are scored as inapplicable (“?”) for this character.

Character 297: Articular, pneumaticity:

0: absent

1: present

Character 298: Dentary, morphology of posterior end:

0: approximately straight or with a weakly developed separation into dorsal and ventral forks (i.e., small dorsal ramus)

1: strongly forked with the dorsal and ventral rami approximately equal in posterior extent

Character 299: Splenial, anterior extent: (ORDERED)

0: limited, stops well posterior to mandibular symphysis

1: elongate, extends to mandibular symphysis but does not participate in symphysis

2: elongate, extends to anterior tip of mandible to contacting on midline and participate in symphysis

Character 300: Mandibular symphysis, anteroposteriorly extensive, flat to convex, dorsal-facing surface:

0: absent, symphysis concave

1: present

Character 301: Mandibular symphysis, symphyseal foramina:

0: absent

1: present

Character 302: Mandibular symphysis, symphyseal foramina, number:

0: single

1: paired

Character 303: Mandibular symphysis, symphyseal foramina, location:

0: opening on posterior edge of symphysis

1: opening on dorsal surface of symphysis

Character 304: Dentary, Meckelian groove, exposure in medial view:

0: exposed as deep and conspicuous groove, not completely covered by splenial

1: not exposed, covered by splenial

Character 305: Lower jaw, anterior external mandibular fenestra:

0: absent

1: present

Character 306: Jugal, contact with postorbital:

0: present

1: absent

Character 307: Frontal/parietal suture

0: open

1: fused

Character 308: Thoracic vertebrae (with ribs articulating with the sternum, or in the middle-posterior dorsal series of theropods without preserved sterna), one or more with prominent hypapophyses:

0: absent

1: present

Character 309: Thoracic vertebrae, number: (ORDERED)

0: 12 or more

1: 11

2: 10 or fewer

Character 310: Thoracic vertebrae, form of articular surfaces:

0: at least part of series with round or ovoid articular surfaces (e.g. amphicoelous/ opisthocoelous) that lack the dorsoventral compression seen in heterocoelous vertebrae

1: series completely heterocoelous

Character 311: Thoracic vertebrae, parapophysis, position:

0: anterior to transverse process

1: directly ventral to transverse process (close to midpoint of vertebra)

Character 312: Thoracic vertebrae, centrum, proportions:

0: approximately equal in anteroposterior length and midpoint mediolateral width

1: length markedly greater than midpoint width

Character 313: Thoracic vertebrae, morphology of lateral surfaces of centra: (UNORDERED)

0: flat to slightly depressed

1: deep, emarginated fossae

2: central ovoid foramina

Note (via Brusatte et al., 2014): Turner et al. (2012) scored some taxa with large pneumatic foramina in the dorsals for character state 2 (e.g., *Balaur*). Because the size and shape of pneumatic foramina are variable, and because dorsal pneumaticity is already scored in other characters, we restrict state 2 to representing the large, ovoid, central foramina of some avialans (and also *Linhenykus*). Turner et al. (2012) also scored *Tyrannosaurus* for state 1, possibly because of the pneumatic foramen and

surrounding fossa on the dorsal centra. We do not consider this homologous to the deeply emarginated fossae on the lateral surfaces of some avialan and oviraptorosaur vertebrae, and therefore consider *Tyrannosaurus* (as well as all other tyrannosauroids and non-maniraptoran coelurosaurs) to be scored for state 0.

Character 314: Thoracic vertebrae with ossified connective tissue bridging transverse processes:

0: absent

1: present

Character 315: Notarium:

0: absent

1: present

Character 316: Sacral vertebrae, series of short vertebrae, with dorsally directed parapophyses just anterior to the acetabulum: (ORDERED)

0: absent

1: present, three such vertebrae

2: present, four such vertebrae

Character 317: Caudal vertebrae, anterior free caudals prior to transition point, length of transverse processes:

0: subequal to width of centrum

1: substantially shorter than centrum width

Character 318: Caudal vertebrae, fusion of distal caudals:

0: unfused to each other

1: fused to each other

Character 319: Caudal vertebrae, extent of fused distal caudals: (ORDERED)

0: fused element length equal or greater than 4 free caudal vertebrae

1: length less than 4 caudal vertebrae

2: less than 2 caudal vertebrae in length

Character 320: Gastralia:

0: present

1: absent

Character 321: Sternum, carina or midline ridge, morphology: (ORDERED)

0: absent

1: slightly raised

2: distinctly projected

Character 322: Sternum, position of carina or midline ridge: (UNORDERED)

0: restricted to posterior half of sternum

1: approaches anterior limit of sternum

2: restricted to the anterior half of the sternum

Character 323: Sternum, dorsal surface, pneumatic foramen (or foramina):

0: absent

1: present

Character 324: Sternum, pneumatic foramina in the depressions (loculi costalis) between rib articulations (processi articularis sternocostalis):

0: absent

1: present

Character 325: Sternum, coracoidal sulci spacing on anterior edge: (UNORDERED)

0: widely separated mediolaterally

1: adjacent

2: crossed on midline

Character 326: Sternum, number of processes for articulation with the sternal ribs: (ORDERED)

0: three

1: four

2: five

3: six

4: seven or more

Character 327: Sternum, raised, paired intermuscular ridges (linea intermuscularis) parallel to sternal midline:

0: absent

1: present

Character 328: Sternum, posterior margin, distinct posteriorly projected medial and/or lateral processes, morphology: (ORDERED)

0: absent

1: with distinct posterior processes

2: midpoint of posterior sternal margin connected to medial posterior processes to enclose paired fenestrae

Character 329: Clavicles, fusion:

0: left and right bones fused together

1: left and right bones unfused

Note (via Brusatte et al., 2014): We here score *Juravenator* as polymorphic "[01]". Chiappe and Göhlich (2010) described an unfused furcula in *Juravenator*, but because this specimen is such a young individual we cannot rule out that the two clavicles may have later fused in adults. Therefore, we conservatively score it as polymorphic for both conditions.

Character 330: Clavicles, interclavicular angle between left and right clavicles:

0: greater than, or equal, to 90 degrees

1: less than 90 degrees

Character 331: Furcula, lateral excavation:

0: absent

1: present

Character 332: Furcula, dorsal (omal) tip, form:

0: flat or blunt

1: with a pronounced posteriorly pointed tip

Character 333: Furcula, ventral margin of apophysis, form:

0: curved, angling

1: with a truncated or squared base

Character 334: Scapula and coracoid, form of articulation: (UNORDERED)

0: pit-shaped scapular cotyla developed on the coracoid, and coracoidal tubercle developed on the scapula (‘ball and socket’ articulation)

1: scapular articular surface of coracoid convex

2: flat

Character 335: Coracoid, procoracoid process:

0: absent

1: present

Character 336: Coracoid, lateral margin, form:

0: straight to slightly concave

1: convex

Character 337: Coracoid, dorsal surface (= posterior surface of taxa less derived than Paraves), form:

0: strongly concave

1: flat to convex

Note (via Brusatte et al., 2014): This character can be scored by examining the trace of the scapula-coracoid suture in lateral view. Those taxa with state 0 have a strong concavity on the coracoid, whereas those scored for state 1 have either a flat suture or one with a convexity on the coracoid.

Character 338: Coracoid, pneumaticity:

0: absent

1: present

Character 339: Coracoid, position of pneumatic foramen:

0: proximal

1: distal

Character 340: Coracoid, lateral process:

0: absent

1: present

Character 341: Coracoid, ventral surface (=anterior surface of taxa less derived than Paraves), lateral intermuscular line or ridge:

0: absent

1: present

Character 342: Coracoid, position of glenoid facet:

0: dorsal to, or at approximately same level as, acrocoracoid process/'biceps tubercle' (or estimated position of biceps tubercle is a discrete tubercle is absent)

1: far ventral to acrocoracoid process

Character 343: Coracoid, acrocoracoid process, shape:

0: straight

1: hooked medially

Character 344: Coracoid, n. supracoracoideus passes through coracoid (usually via coracoid foramen):

0: present

1: absent

Note (via Brusatte et al., 2014): This character is not equivalent to the presence or absence of a large, discrete coracoid foramen (Turner et al. 2012, character 440). Although a noticeable foramen is sometimes absent in non-avialan coelurosaurs, the overall morphology of the coracoid (and pectoral region in general) suggests that the n. supracoracoideus still passes through the coracoid, unlike the condition in some derived avialans. In the absence of soft tissue information in fossil taxa, we consider all non-paravian theropods with a traditional plate-like coracoid to possess state 0.

Character 345: Coracoid, medial surface, area of the foramen n. supracoracoideus (when developed)

0: strongly depressed

1: flat to convex

Note (via Brusatte et al., 2014): Turner et al. (2012) scored the outgroup *Allosaurus* and derived tyrannosauroids for state 1, but in these taxa this surface of the coracoid is indeed depressed (the condition in these taxa is basically identical to the ornithomimosaur *Gallimimus*, for instance, which was scored for state 0). Therefore, we score these taxa for state 0. State 1 is now scored only in some avialans, some alvarezsaurids, and *Microvenator*.

Character 346: Coracoid and scapula, angle between bones at glenoid:

0: greater than 90 degrees

1: 90 degrees or less

Character 347: Scapula, ratio of dorsoventral depth of distal end to minimum dorsoventral depth of blade: (ORDERED)

0: greater than 2.5

1: slightly taller or approximately the same depth as proximal dorsoventral shaft width, ratio between 1.0 and 2.5

2: tapering distally, ratio less than 1.0

Note (via Brusatte et al., 2014): This character has been modified into an ordered multistate to take into account variation within tyrannosauroids noted by Brusatte et al. (2010a, character 235).

Character 348: Scapula, curvature in lateral view:

0: absent, bone straight

1: present, bone dorsoventrally curved

Note (via Brusatte et al., 2014): Turner et al. (2012) scored some basal coelurosaurs (*Haplocheirus*, *Mononykus*, *Archaeornithomimus*, *Gallimimus*) for the curved condition, but these taxa have straight scapular blades that are extremely similar in overall shape to closely related that that were scored for the straight condition. We score them for the straight condition here, meaning that the curved condition is restricted to some derived maniraptorans.

Character 349: Scapula, position of acromion process:

0: extends anteriorly to surpass the articular surface for coracoid (facies articularis coracoidea)

1: does not extend further anteriorly than the articular surface for coracoid

Character 350: Scapula, orientation of acromion process:

0: straight

1: laterally hooked tip

Note (via Brusatte et al., 2014): Turner et al. (2012) scored tyrannosauroids for state 1, which is otherwise present only in some derived avialans. We do not recognize any discrete difference in the orientation of the acromion process of derived tyrannosauroids relative to other basal coelurosaurs, and therefore score all tyrannosauroids with well-preserved scapulae for state 0.

Character 351: Humerus and ulna, length comparison: (ORDERED)

0: humerus longer than ulna

1: ulna and humerus approximately the same length (within ~10% of each other)

2: ulna substantially longer than humerus

Character 352: Humerus, head, shape in anterior or posterior view

0: strap-like, articular surface flat or weakly convex, no proximal midline convexity

1: prominent and highly convex (domed) proximally

Note (via Brusatte et al., 2014): We consider this character equivalent to character 240 in Brusatte et al. (2010a), which scores for the absence/presence of an enlarged head that occupies the majority of the proximal end, is bulbous in roximal view, and overhangs both anterior and posterior surfaces. This feature, which is seen in some derived tyrannosauroids, is here considered equivalent to state 1.

Character 353: Humerus, proximal end, shape of proximal projection:

0: dorsal edge projected farthest

1: midline projected farthest

Character 354: Humerus, ventral tubercle and capital incisure:

0: absent

1: present

Character 355: Humerus, capital incisure, morphology:

0: an open groove

1: closed by tubercle associated with a muscle insertion just distal to humeral head

Character 356: Humerus, anterior surface, well-developed fossa on midline making proximal articular surface appear v-shaped in proximal view:

0: absent

1: present

Character 357: Humerus, 'transverse groove':

0: absent

1: present, developed as a discrete, depressed scar on the proximal surface of the bicipital crest or as a slight transverse groove

Character 358: Humerus, deltopectoral crest, orientation:

0: projected laterally or dorsally, such that it is in line with the long axis of humeral head

1: projected anteriorly, such that it is approximately perpendicular to the long axis of the head

Character 359: Humerus, deltopectoral crest, thickness compared with thickness of shaft: (ORDERED)

0: less

1: same width

2: dorsoventral thickness greater than shaft thickness

Character 360: Humerus, deltopectoral crest, proximoposterior surface (=proximolateral surface in most non-avialan theropods), form:

0: flat to convex

1: concave

Character 361: Humerus, deltopectoral crest, large fenestra:

0: absent, crest not perforate

1: present, crest perforate

Character 362: Humerus, bicipital crest, pit-shaped scar/fossa for muscular attachment on anterodistal, distal, or posterodistal surface of crest:

0: absent

1: present

Character 363: Humerus, bicipital crest, position of pit-shaped fossa for muscular attachment: (UNORDERED)

0: anterodistal on bicipital crest

1: directly ventrodistal at tip of bicipital crest

2: posterodistal, variably developed as a fossa

Character 364: Humerus, bicipital crest, anterior projection: (ORDERED)

0: absent or subtle

1: present, developed as an anterior projection relative to shaft surface in ventral view

2: present as a hypertrophied, rounded tumescence

Character 365: Humerus, proximal end, one or more pneumatic foramina:

0: absent

1: present

Character 366: Humerus, distal articular condyles, position:

0: on distal surface of bone

1: on anterior surface of bone

Character 367: Humerus, long axis of dorsal (lateral) distal condyle, orientation:

0: at low angle to humeral axis, proximodistally orientated

1: at high angle to humeral axis, almost transversely orientated

Character 368: Humerus, distal condyles, form:

0: prominent, subround and bulbous

1: weakly defined, 'strap-like'

Character 369: Humerus, distal margin, orientation:

0: approximately perpendicular to long axis of humeral shaft

1: oblique (angling strongly ventrally) to long axis of humeral shaft, ventrodistal margin projected significantly distal to dorsodistal margin (sometimes described as a well-projected flexor process)

Character 370: Humerus, distal end, compressed anteroposteriorly and flared dorsoventrally:

0: absent

1: present

Character 371: Humerus, brachial fossa:

0: absent

1: present, developed as a flat scar or as a scar-impressed fossa

Character 372: Humerus, ventral (medial) distal condyle, length of long axis:

0: less than the long axis of the dorsal (lateral) condyle

1: the same or greater than the long axis of the dorsal (lateral) condyle

Character 373: Humerus, demarcation of muscle origins (e.g. m. extensor metacarpi radialis in Aves) on the dorsal (=lateral in non-avialan theropods) edge of the distal humerus:

0: no indication of origin as a scar, a pit, or a tubercle

1: indication as a pit-shaped scar or as a variably projected scar-bearing tubercle or facet

Character 374: Humerus, groove for passage of m. scapulotriceps on distal end of posterior surface:

0: absent

1: present

Character 375: Humerus, m. humerotricipitalis groove:

0: absent

1: present as a ventral depression contiguous with the olecranon fossa

Character 376: Ulna, cotylae, orientation of dorsal (lateral) and ventral (medial) cotylae:

0: dorsoventrally adjacent

1: widely separated by a deep groove

Character 377: Ulna, dorsal cotyla, form: convex

0: flat or non-distinct

1: convex

Character 378: Ulna, distal end, dorsal (lateral) condyle, dorsal trochlear surface, extent along posterior margin:

0: less than transverse measure of dorsal trochlear surface

1: approximately equal in extent

Character 379: Ulna, bicipital scar: (ORDERED)

0: absent

1: present, developed as a slightly raised scar

2: present, developed as a conspicuous tubercle

Character 380: Ulna, brachial scar

0: absent

1: present

Character 381: Radius, posteroventral surface (=posteromedial surface of non-avialans), texture: (UNORDERED)

0: smooth

1: with muscle impression along most of surface

2: deep longitudinal groove

Character 382: Ulnare:

0: absent

1: present

Character 383: Ulnare, shape:

0: circular, triangular, or ‘heart-shaped’, with no or minimal differentiation into short dorsal and ventral rami

1: V-shaped, well- developed dorsal and ventral rami

Character 384: Ulnare, ventral ramus (crus longus), length: (ORDERED)

0: shorter than dorsal ramus (crus brevis)

1: same length as dorsal ramus

2: longer than dorsal ramus

Character 385: Semilunate carpal and metacarpals, fusion: (ORDERED)

0: separate from each other, no fusion

1: incomplete proximal fusion

2: complete proximal fusion

3: complete proximal and distal fusion

Character 386: Metacarpal III, anteroposterior (extensor-flexor) diameter at midshaft compared to anteroposterior diameter of metacarpal II:

0: approximately equal or greater than 50%

1: less than 50%

Character 387: Metacarpal I, anteroproximally projected muscular process, form: (ORDERED)

0: absent, no distinct process visible

1: present as small knob at anteroproximal tip of metacarpal

2: present, tip of process marginally surpasses the distal articular facet for phalanx 1 in anterior extent

3: present, tip of process conspicuously surpasses articular facet by approximately half the width of the facet, producing a pronounced knob

4: present, tip of process conspicuously surpasses articular facet by approximately the entire width of the facet, producing a pronounced knob

Character 388: Metacarpal I, anterior (extensor) surface, shape:

0: roughly hourglass-shaped proximally, at least moderately expanded anteroposteriorly, and constricted just before flare of articulation for phalanx 1

1: anterior surface broadly convex

Character 389: Carpometacarpus, pisiform process:

0: absent

1: present

Character 390: Carpometacarpus, ventral surface, supratrochlear fossa deeply excavating proximal surface of pisiform process or adjacent region:

0: absent

1: present

Character 391: Carpometacarpus, intermetacarpal space (between metacarpals II and III):

0: reaches proximally as far as the distal end of metacarpal I

1: terminates distal to end of metacarpal I

Note (via Brusatte et al., 2014): All taxa without a carpometacarpus are scored as inapplicable ("?").

Character 392: Metacarpals II and III, distal ends, position of articular surfaces for digits:

0: articular surface on metacarpal II located at same distal level as, or surpasses distally, articular surface on metacarpal III

1: articular surface on metacarpal III extends further distally than articular surface on metacarpal II

Character 393: Metacarpals, intermetacarpal process or tubercle: (ORDERED)

0: absent

1: present as scar

2: present as tubercle or flange

Character 394: Manual digit II, phalanx 1, shape:

0: subcylindrical to subtriangular in cross section

1: strongly dorsoventrally compressed, flat posterior surface

Character 395: Manual digit II, length of phalanx II-1 compared to that of II-2:

0: less than or equal to

1: longer

Character 396: Manual digit II, phalanx 2, internal index process on posterodistal edge:

0: absent

1: present

Character 397: Pelvis, ilium, ischium, and pubis, proximal contact between bones in adult individuals: (ORDERED)

0: unfused to each other

1: partially fused to each other (pubis not ankylosed)

2: completely fused to each other

Note (via Brusatte et al., 2014): State 0 includes the condition in derived therizinosauroids in which the pubis and obturator process of the ischium meet and fuse ventral to the acetabulum, which does not include fusion between the ilium and other pelvic bones.

Character 398: Ilium/ischium, distal co-ossification to completely enclose the ilioischiadic fenestra:

0: absent

1: present

Character 399: Ischium, dorsal process

0: does not contact ilium

1: contacts ilium

Note (via Brusatte et al., 2014): All taxa without a dosrsal process are scored as inapplicable (“?”).

Character 400: Ilium, antitrochanter, position:

0: directly posterior to acetabulum

1: posterodorsal to acetabulum

Character 401: Ilium, preacetabular pectineal process: (ORDERED)

0: absent

1: present as a small flange

2: present as a well-projected flange

Character 402: Ilium, preacetabular processes, orientation of left and right processes:

0: approach on midline but do not contact (open space between them, possibly a cartilaginous connection)

1: make contact with each other on midline and fuse, dorsal closure of 'iliosynsacral canals'

Character 403: Ilium, preacetabular process, morphology of process as it extends anterior to first sacral vertebra:

0: no free ribs overlapped

1: one or more ribs overlapped

Character 404: Ilium, postacetabular process, orientation:

0: dorsoventrally orientated, such that broad external surface faces laterally

1: mediolaterally orientated, such that broad external surface faces dorsally

Character 405: Ilium, postacetabular process, ventral surface, renal fossa:

0: absent

1: present

Character 406: Ilium, cuppedicus fossa, form:

0: broad, mediolaterally oriented surface directly anteroventral to acetabulum

1: small and entirely laterally facing fossa anterior to the acetabulum

Note (via Brusatte et al., 2014): All taxa without a cuppedicus fossa (scored as absent for the character denoting the absence/presence of the fossa above) are scored here as inapplicable ("?").

Character 407: Pubis, cross section:

0: suboval

1: compressed mediolaterally

Character 408: Pubes, contact of distal ends of left and right pubes:

0: present, variably co-ossified into symphysis

1: absent, pubes non-contacting

Character 409: Femur, posterior trochanter: (ORDERED)

0: present, developed as a slightly projected tubercle or flange

1: present, hypertrophied into a ‘shelf-like’ conformation (in combination with development of the trochanteric shelf)

2: absent

Character 410: Femur, patellar groove

0: absent

1: present

Note (via Brusatte et al., 2014): Turner et al. (2012) scored derived tyrannosauroids for possessing a patellar groove, probably because these taxa have a deep extensor groove for muscle attachment (a feature that is variably developed in tetanurans). We do not consider this feature homologous to the distinct patellar groove of birds, which houses a patella ossification (which is not known for tyrannosauroids or any other non-avialan theropod). Instead, we utilize a separate character (below) to refer to the extensor groove.

Character 411: Femur, ectocondylar tubercle and lateral condyle, separation:

0: present, separated by deep notch

1: absent, form single trochlear surface

Character 412: Femur, posterior projection of the lateral border of the distal end, continuous with lateral condyle:

0: absent

1: present

Character 413: Femur, laterally projected fibular trochlea: (ORDERED)

0: absent

1: present, developed as small notch

2: present, developed as a shelf-like projection

Character 414: Tibia/tarsals, condyles, extent of medial and lateral condyles:

0: medial condyle projecting further anteriorly than lateral

1: equal in anterior projection

Character 415: Tibia/tarsals, condyles, extensor canal: (ORDERED)

0: absent

1: present as an emarginated groove

2: present as a groove bridged by an ossified supratendoneal bridge

Character 416: Tibia/tarsals, condyles, tuberositas retinaculi extensoris indicated by short medial ridge or tubercle proximal to the condyles close to the midline and a more proximal second ridge on the medial edge:

0: absent

1: present

Character 417: Tibia/tarsals, condyles, mediolateral widths of lateral and medial condyles: (ORDERED)

0: medial condyle wider

1: approximately equal

2: lateral condyle wider

Character 418: Tibia/tarsals, condyles, medial constriction of lateral and medial condyles:

0: present, gradual sloping medial constriction of condyles

1: absent, no medial tapering of either condyle

Character 419: Tibia/tarsals, condyles, intercondylar groove, mediolateral width:

0: broad, approximately 1/3 width of anterior surface

1: narrow, less than 1/3 width of total anterior surface

Character 420: Tibia/tarsals, position of articular surface for distal tarsals/tarsometatarsus: (ORDERED)

0: on distal surface or restricted to distal-most edge of posterior surface, no broad extension of trochlear surface onto posterior surface of bone

1: well-developed articular surface extending up the posterior surface of the tibiotarsus (sulcus cartilaginis tibialis of Aves)

2: well-developed articular surface extending up the posterior surface of the tibiotarsus, with well-developed and posteriorly projecting medial and lateral crests

Character 421: Tibia, mediolateral width of distal end:

0: wider than midpoint of shaft, giving distal profile a weakly developed triangular form

1: approximately equal to midshaft width, no distal expansion of whole shaft (although condyles may be variably splayed mediolaterally)

Character 422: Metatarsal V:

0: present

1: absent

Character 423: Metatarsal III, position of proximal end:

0: proximally in same plane with II and IV

1: proximally displaced plantarly (to the extensor surface), relative to metatarsals II and IV

Character 424: Tarsometatarsus or metatarsals, intercotylar eminence:

0: absent

1: well developed, globose

Character 425: Tarsometatarsus or metatarsals, projected surface or grooves on proximoposterior surface (associated with the passage of tendons of the pes flexors in Aves; hypotarsus): (ORDERED)

0: absent

1: developed as posterior projection with flat posterior surface

2: developed as a posterior projection, with distinct crests and grooves

3: developed as a posterior project with distinct crests and grooves, at least one groove enclosed by bone posteriorly

Character 426: Tarsometatarsus or metatarsals, proximal vascular foramina: (ORDERED)

0: absent

1: single foramen present, between metatarsals III and IV

2: two foramina present

Character 427: Metatarsal I, shape: (ORDERED)

0: straight

1: curved or distally deflected but not twisted, ventral surface convex (‘J shaped’)

2: distally deflected and twisted, such that the ventromedial surface is concave proximal to trochlear surface for phalanx I

Character 428: Metatarsal II, distal extensor surface, fossa for metatarsal I: (ORDERED)

0: absent

1: present as a shallow notch

2: present as a conspicuous ovoid fossa

Character 429: Metatarsal IV, mediolateral width at midshaft: (UNORDERED)

0: metatarsal IV approximately the same width as metatarsals II and III

1: metatarsal IV narrower than MII and MIII

2: metatarsal IV greater in width than either metatarsal II or III

Character 430: Metatarsals, comparative trochlear width (UNORDERED):

0: II approximately the same size as III and/or IV (this includes taxa in which metatarsal III is slightly wider than the other metatarsals disally)

1: II markedly wider than III and/or IV

2: II markedly narrower than III and/or IV

3: IV markedly narrowest.

Character 431: Metatarsus, distal vascular foramen, form:

0: simple, with one exit

1: forked, two exits (plantar and distal) between metatarsals III and IV.

Character 432: Metatarsal III, distal trochlea in extensor view, proximal extent of lateral and medial edges:

0: equally extended proximally

1: lateral edge extends further proximally

Character 433: Metatarsal II, distal extent of metatarsal II relative to metatarsal IV: (ORDERED)

0: approximately equal in distal extent

1: metatarsal II shorter than metatarsal IV, but reaching distally further than base of metatarsal IV trochlea

2: metatarsal II shorter than metatarsal IV, reaching distally only as far as base of metatarsal IV trochlea

Character 434: Caudal vertebrae, middle to posterior caudals, anteroposterior length: (ORDERED)

0: shortened, less than 1.5x length of dorsal vertebrae (where known) and anteroposterior length of centrum less than twice its maximum mediolateral width

1: 1.5x-2x or less the length of dorsal vertebrae

2: 3x-4x length of dorsal vertebrae

Note (via Brusatte et al., 2014): This is a modified version of the original Turner et al. (2012) character, which takes into account an additional state (denoted as state 0) noted by Zanno et al. (2009).

Character 435: Coracoid, coracoid fenestra:

0: absent

1: present

Character 436: Metatarsal V length:

0: less than 40% of metatarsal III's length

1: more than 40% of metatarsal III's length

Note (via Cau et al., 2015): The character did not previously quantitatively define metatarsal V's elongation, with the derived state present exclusively in microraptorine and eudromaeosaurian dromaeosaurids among Coelurosauria, and *Balaur bondoc* showing the plesiomorphic state.

Character 437: Chevrons, degree of posterior extension of posterior chevrons:

0: not substantially elongated

1: very elongated

Character 438: Radius, width at midshaft:

0: roughly half or greater than width of ulna

1: less than half width of ulna

Character 439: Manus, combined length of metacarpal I and phalanx I-1:

0: greater than length of MC II

1: equal to or less than length of MC II

Character 440: Metacarpal III, shape:

0: straight

1: bowed

Character 441: Metatarsal I, position of distal trochlea:

0: proximally placed relative to trochleae of other metatarsals

1: inline distally with others

Character 442: Metatarsal I:

0: present

1: absent

Character 443: Braincase, preotic pendant, form: (ORDERED)

0: absent

1: present but small

2: present and robust

Character 444: Braincase, metotic strut, shape:

0: short and robust

1: long and narrow

Character 445: Prootic, pneumatic excavation on lateral surface leading into anterior tympanic recess: (ORDERED)

0: absent

1: present and shallow

2: present and deep

Note (via Brusatte et al., 2014): Witmer (1997), Makovicky and Norell (1998), Bever et al. (2013) and others have described lateral pneumatic excavations on the prootic, which are present in some coelurosaurs, to be associated with the anterior tympanic recess, which is present in most theropods. The character here does not code for the absence/presence of the recess, but rather for the absence/presence of discrete external pneumatic openings on the lateral surface of the prootic that lead into the recess. Contra Turner et al. (2012), tyrannosauroids are scored for state 1, as they possess a distinct fossa on the lateral surface of the prootic that includes the external foramina of the trigeminal and facial nerves, along with pneumatic openings that lead into the anterior tympanic recess (Bever et al. 2013). This fossa itself is a distinct tyrannosauroid character, which is listed as a separate character below.

Character 446: Braincase, anterior tympanic recess (ATR), manifestation on external surface of basisphenoid: (ORDERED)

0: subtle, external fossa not deeply impressed into the lateral surface of the basisphenoid

1: deeply impressed into the lateral surface of the basisphenoid in juveniles but not adults

2: deeply impressed into the lateral surface of the basisphenoid in adults

Note (via Brusatte et al., 2014): Turner et al. (2012) originally scored for the absence/presence of an anterior tympanic recess. Witmer (1997), Bever et al. (2013), and others have shown that this recess is present in most theropods, so we have modified this character to refer specifically to the external manifestation of the recess on the basisphenoid. This character scores for a shallow/deep lateral fossa or fossae on the basisphenoid. We have also subdivided the “deeply impressed” condition into two states, to take into account the recognized loss of a deeply impressed recess during ontogeny in some tyrannosauroids (Bever et al. 2013). This character is therefore equivalent to Brusatte et al. (2010a, character 157).

Character 447: Braincase, anterior tympanic recess and anterior tympanic crista (crest marking the posterior and dorsal border of the ATR), location:

0: below the exit foramen of cranial nerve VII exit and just proximal to the otic recess

1: anteriorly, with little or no development of the recess posterior to the basipterygoid processes

Character 448: Braincase, anterior tympanic recess confluent with the subotic recess:

0: absent

1: present, forming the Lateral Depression

Note (via Brusatte et al., 2014): All taxa without a subotic recess are scored as inapplicable (“?”).

Character 449: Braincase, V-shaped opening between basal tubera (or remnants of tubera):

0: absent

1: present

Character 450: Braincase, small tubera medial to basal tubera (or remnants of basal tubera) and ventral to occipital condyle:

0: absent

1: present

Character 451: Pedal phalanx II-2, size of distal articular surface relative to proximal articular surface:

0: approximately equal in size or distal surface slightly smaller than proximal surface

1: distal surface less than half the size of proximal surface

Character 452: Sternum, ossification of sternal plates:

0: absent, plates unossified

1: present, plates ossified

Character 453: Ulna, size of proximal cotylae:

0: unequal in size, lateral (=dorsal in avialans) condyle smaller

1: equal in size

Character 454: Braincase, middle ear resides within the Lateral Depression (formed by merged anterior tympanic recess and subotic recess):

0: absent

1: present

Character 455: Feathers, filamentous integumentary structures (Stage 1 feathers):

0: absent

1: present

Character 456: Feathers, vaned feathers (Stage 4 feathers):

0: absent

1: present

Character 457: Quadratojugal, size:

0: large

1: greatly reduced

Character 458: Frontal, notch for postorbital contact on postorbital process of frontal:

0: absent, process smooth or facet small

1: large notch present

Character 459: Frontal and parietal, position of fronto-parietal suture relative to postorbital processes of frontal: (ORDERED)

0: well posterior to the postorbital processes

1: at the level of the postorbital processes

2: anterior to postorbital processes

Character 460: Cervical vertebrae, orientation of articular surfaces between cervical vertebrae:

0: surfaces vertical to subvertical

1: strongly slanted anteroventrally

Character 461: Skull roof, accessory depression in supratemporal fossa:

0: absent

1: present

Character 462: Ilium, relative ventral extension of pubic and ischiadic peduncles: (ORDERED)

0: equal

1: pubic peduncle extends farther ventrally

2: pubic peduncle hyperelongate, approximately 2.5-3 times the proximodistal length of the ischial peduncle

Note (via Brusatte et al., 2014): This is a modified version of the original Turner et al. (2012) character, which incorporates a second derived state referred to the hyperelongate condition of some derived therizinosauroids (see Zanno et al. 2009: character ZCD 311). We also consider this character to be equivalent to Zanno et al. (2009: character ZCD 309).

Character 463: Parasphenoid, ala parasphenoidalis:

0: absent

1: present, well-developed and crest-shaped forming anterior edge of enlarged pneumatic recess with the ala continuous with the anterior tympanic crista

Character 464: Furcula, cross-section:

0: nearly circular

1: anteroposteriorly compressed near the symphysis

Character 465: Furcula, shape:

0: V-shaped

1: U-shaped

Character 466: Furcula, epicledial processes, form:

0: unexpanded

1: expanded

Character 467: Furcula, lateral expansion of the rami between the hypocledium and the epicledial process:

0: absent

1: present

Character 468: Furcula, hypocledium, shape:

0: rounded

1: keeled

Character 469: Furcula, symmetry:

0: asymmetrical

1: nearly symmetrical

Character 470: Furcula, thickness of rami:

0: thin

1: thick

Character 471: Metatarsal III, extensor (=anterior or dorsal) surface, shape:

0: relatively narrow and flat

1: transversely expanded and slightly concave

Character 472: Metatarsal IV, accessory longitudinal ridge on anterolateral side of the distal end of the bone:

0: absent

1: present

NEW CHARACTERS ADDED TO THE TWiG DATASET BY BRUSATTE ET AL. (2014):

***Expansions of Turner et al. (2012) Characters:**

Character 473: Metacarpal I, width of proximal articular surface compared to proximodistal length of entire metacarpal:

0: shorter

1: wider

Note (via Brusatte et al., 2014): This character is a new character, originally part of Turner et al. (2012, character 149), which distinguishes the extremely short and wide metacarpals I of some alvarezsaurids.

Character 474: Manual unguals:

0: present

1: absent

Note (via Brusatte et al., 2014): This character is a new character, originally part of Turner et al. (2012, character 151), which distinguishes the absent manual unguals of derived avialans.

Character 475: Manual unguals, form of flexor tubercle: (ORDERED)

0: large, robust, rugose, conical structure

1: reduced to a small convexity

2: absent

Note (via Brusatte et al., 2014): This character is a new character, originally part of Turner et al. (2012, character 151), which distinguishes the large flexor tubercles of most coelurosaurs from the small flexor tubercles of some tyrannosauroids, ornithomimosaurs, and other taxa. Some alvarezsauroids have completely absent flexor tubercles, which is denoted by state 2. This character is equivalent to Brusatte et al. (2010a, character 255).

***Characters Relevant to Tyrannosauroidea (from Brusatte et al. 2010a):**

Character 476: Skull, occipital region, orientation:

0: posteriorly

1: posteroventrally

Character 477: Skull, general shape:

0: long and low, length: height ratio greater than 3.2

1: deep, length: height ratio less than 3.2

Note (via Brusatte et al., 2014): Length is premaxilla-quadrate cotyle length; height is maximum height of the upper jaw, not counting any cranial crests.

Character 478: Skull, anteroposterior length:

0: less than 40% trunk length

1: greater than 40% trunk length

Note (via Brusatte et al., 2014): Trunk length is the anterior extremity of the pectoral girdle to the posterior extremity of the pelvic girdle, as defined by Sereno et al. (2009).

Character 479: Lateral temporal fenestra, orientation of long axis relative to long axis of orbit:

0: posterodorsal

1: approximately parallel

Character 480: Premaxilla, nasal process of opposing premaxillae, orientation:

0: divergent from each other, with small process of nasals fitting in between them

1: closely appressed to each other

Character 481: Premaxilla, deep foramen or fossa on the lateral surface of the base of the nasal process, within the anteroventral corner of the narial fossa:

0: absent

1: present

Character 482: Premaxilla, maxillary process orientation: (ORDERED)

0: mostly laterally (and resultantly widely visible in lateral view)

1: dorsolaterally (facing almost equally dorsally and laterally)

2: dorsally (and resultantly mostly hidden in lateral view)

Character 483: Premaxilla, form of narial fossa ventral to external naris:

0: shallowly excavated

1: deeply excavated, anterior margin invaginated as a deep groove

Character 484: Premaxilla, extent of narial fossa:

0: limited to region immediately ventral to external naris

1: extensive, covers most of main body of premaxilla

Character 485: Premaxilla, orientation and shape of anterior margin: (ORDERED)

0: smoothly curved and projecting posterodorsally, angle between ventral margin of premaxilla and anterior margin is less than 90 degrees;

1: smoothly curved and projecting vertically or slightly anterodorsally, angle between ventral margin of premaxilla and anterior margin is equal to or greater than 90 degrees

2: projecting vertically or slightly anterodorsally, with a discrete inflection point between a more vertical ventral portion and a more horizontal dorsal portion

Note (via Brusatte et al., 2014): We have transformed the original character of Brusatte et al. (2010a: character 13) into an ordered multistate by adding a new intermediate state referring to an approximately vertical (or slightly anterodorsally inclined) anterior margin of the ventral portion of the premaxilla. State 2 now refers to a special condition of this state, in which the anterior margin is vertical (or anterodorsally inclined) and there is also a discrete inflection point between the more vertical ventral portion of the anterior margin and the more horizontal dorsal portion of the margin. Taxa with this condition, therefore, do not possess a smoothly curved anterior margin of the premaxilla. Tyrannosauroids are characterized by either state 1 or state 2, with state 2 referring to a subset of basal tyrannosauroids (*Dilong*, *Guanlong*, *Kileskus*, *Proceratosaurus*, *Sinotyrannus*).

Character 486: Premaxilla, position of palatal process:

0: immediately above interdental plates

1: separated from interdental plates by deep lingual surface of premaxilla

Character 487: Maxilla, promaxillary fenestra, position:

0: anterior margin of antorbital fossa

1: extreme anteroventral corner of antorbital fossa

Character 488: Maxilla, maxillary fenestra, anteroposterior length compared to the distance between the anterior margins of the antorbital fossa and fenestra: (ORDERED)

0: less than half

1: greater than half

2: greater than half and also greater than half of the length of the eyeball-bearing portion of the orbit

Character 489: Maxilla, maxillary fenestra, position within maxillary antrum:

0: does not abut dorsal border of the antrum in medial view

1: abuts dorsal border of the antrum in medial view

Character 490: Maxilla, antorbital fossa, extent:

0: reaches nasal suture

1: does not reach nasal suture

Character 491: Maxilla, interfenestral strut, anteroposterior length:

0: greater than 50% of long axis of maxillary fenestra

1: less than 50% of long axis of maxillary fenestra

Note (via Brusatte et al., 2014): In oviraptorosaurs, the size of the interfenestral strut is measured relative to the "accessory antorbital fenestra," whose homology with the maxillary and/or premaxillary fenestrae of other theropods is not clear (e.g., Balanoff et al. 2009).

Character 492: Maxilla, antorbital fossa, trend of dorsoventral depth across main body:

0: uniform

1: diminishes

Character 493: Maxilla, subcutaneous flange bordering the antorbital fossa laterally on the posterior end of the main body, resulting in the fossa forming a channel between the flange and the main body:

0: absent

1: present

Character 494: Maxilla, dorsolateral process, coverage by antorbital fossa: (ORDERED)

0: process absent

1: process covered by subcutaneous surface only

2: ventral half of process covered by antorbital fossa

3: antorbital fossa completely excluded

Character 495: Maxilla, narrow region of smooth surface texture between anterior margin of antorbital fossa and the subcutaneous surface:

0: absent

1: present

Character 496: Maxilla, ventral margin of the anterior region of the bone, profile:

0: straight

1: convex

Character 497: Maxilla, joint surface for palatine, depth:

0: shallow, does not obscure the tooth root bulges from view

1: deep, obscures tooth root bulges from view

Character 498: Maxilla, anterior ramus (demarcated by concave step in anterior margin of maxilla):

0: absent

1: present

Character 499: Maxilla, form of contact with nasal in subadult to adult specimens: (ORDERED)

0: smooth

1: weakly scalloped

2: deeply scalloped with interlocking transverse ridges on both elements

Character 500: Maxilla, form of external subcutaneous surface texturing:

0: random foramina and shallow grooves and ridges

1: deep, prominent, dorsoventrally trending grooves and ridges

Character 501: Maxilla, swollen rim separating antorbital fossa and subcutaneous surface:

0: present

1: absent

Character 502: Maxilla, size of ascending ramus, anteroposterior chord directly above maxillary fenestra compared to dorsoventral depth of maxilla below anterior edge of antorbital fenestra:

0: greater than 1.75 times (ascending ramus large)

1: less than 1.60 times (ascending ramus small)

Character 503: Maxilla, posterior region of the main body (portion including the final 3-5 teeth and anterior to the jugal process), shape:

0: maintains a relatively constant dorsoventral depth

1: tapers in depth posteriorly

Character 504: Maxilla, primary row of neurovascular foramina, form:

0: continues as a row posteriorly

1: transitions posteriorly into a sharp groove, paralleling the antorbital fossa rim

Character 505: Maxilla, antorbital fossa, extent on main body:

0: covers more than half of the depth of the main body beneath the anterior margion of the antorbital fenestra

1: covers less than half of this depth

Character 506: Nasals, midline crest on dorsal surface:

0: absent

1: present

Character 507: Nasal, shape in dorsal view: (UNORDERED)

0: expands in width posteriorly

1: relatively constaint width across the length of the bone, due to subparallel lateral sides

2: tapers in width posteriorly

Character 508: Nasal, frontal process, mediolateral width:

0: unconstricted

1: constricted, less than ½ width of widest point of nasal

Character 509: Nasal, posterolateral process that overlaps the lateral surface of the lacrimal:

0: absent

1: present

Character 510: Nasal, extent of narial fossa on premaxillary process:

0: limited to ventral margin of process

1: covers entire process, and thus meets opposite fossa on dorsal midline

Character 511: Nasal, medial processes of frontal articulation, shape: (UNORDERED)

0: processes absent or very subtle

1: lanceolate

2: tapered

Character 512: Nasal, thin, low, and laterally projecting crest at the corner where lateral and dorsal surfaces meet:

0: absent

1: present

Character 513: Lacrimal, angle between anterior and ventral rami

0: 90 degrees (=inverted L shaped) or greater

1: approximately 70-80 degrees (=7 shaped)

Character 514: Lacrimal, cornual process on dorsal surface, form: (UNORDERED)

0: barely perceptible ridge across entire length of anterior ramus (equivalent to the state "cornual process absent" in tyrannosauroid-specific datasets)

1: broad, shallow, dorsally convex, laterally overhanging swelling across most of the length of the anterior ramus

2: discrete conical projection

3: small, conical, smooth projection that rises 2-3 millimeters from skull roof

Note (via Brusatte et al., 2014): All taxa without a cornual process (those scored as absent for character 37) are here scored as inapplicable ("?").

Character 515: Lacrimal, cornual process, form:

0: smoothly rounded

1: discrete apex present

Note (via Brusatte et al., 2014): All taxa without a discrete cornual process (those scored as absent for character 37 and those scored as 0 for character 514) are here scored as inapplicable ("?").

Character 516: Lacrimal, cornual process, position of apex:

0: dorsal to ventral ramus

1: anterior to ventral ramus

Note (via Brusatte et al., 2014): All taxa without a discrete cornual process (those scored as absent for character 37 and those scored as 0 for character 514 and 515) are here scored as inapplicable ("?").

Character 517: Lacrimal, anterior ramus, pneumaticity:

0: not inflated

1: inflated by pneumatic recess

Character 518: Lacrimal, size of primary external opening for lacrimal recess:

0: small, anterior end located approximately at the same level as the anterior end of the ventral ramus

1: large, anterior end located far anterior to the ventral ramus

Character 519: Lacrimal, interaction of primary external opening for lacrimal recess and antorbital fossa:

0: separate

1: blend

Character 520: Lacrimal, accessory external openings for lacrimal recess on the anterior ramus: (ORDERED)

0: absent

1: present and proximally located (i.e., near to primary recess)

2: present and distally located

Character 521: Lacrimal, pneumatic recess opening internally onto medial surface of bone as a discrete pneumatic fenestra (pneumatopore):

0: absent

1: present

Character 522: Lacrimal, dorsal prong of anterior ramus for articulation with maxilla ("anterodorsal process"), size:

0: absent or small

1: present and elongate

Note (via Brusatte et al., 2014): We have combined "absent" and "small" into a single character statement, because the subtle process can easily be mistaken for absent if it is broken or poorly preserved.

Character 523: Lacrimal, ventral ramus, extent of medial lamina:

0: greater than half of the dorsoventral depth of the ramus

1: half or less of the dorsoventral depth of the ramus

Character 524: Lacrimal, orbitonasal ridge on medial surface, position:

0: anterior to posterior margin of ventral ramus

1: adjacent to or contacting posterior margin of ventral ramus

Character 525: Lacrimal, articulation with frontal, form:

0: squamous

1: conical lacrimal process set into deep pit in frontal

Character 526: Lacrimal, posterior process for articulation with frontal, inflated by pneumatic recess:

0: no

1: yes

Character 527: Lacrimal, extent of antorbital fossa on ventral ramus:

0: covers greater than 60% of anteroposterior length along the contact with the jugal

1: covers less than this measure

Character 528: Lacrimal, maxillary process of anterior ramus, visibility in lateral view:

0: both dorsal and ventral margins visible

1: dorsal margin concealed by subcutaneous surface abouve antorbital fossa and only ventral margin visible

Note (via Brusatte et al., 2014): Those taxa without a maxillary process ("dorsal prong" in character 522) are here scored as inapplicable ("?").

Character 529: Jugal, maxillary ramus, depth:

0: shallow, not expanded relative to subortibal portion of bone

1: deep, expanded relative to suborbital portion of bone

Character 530: Jugal, antorbital fossa, extent on maxillary ramus:

0: edge of fossa undercut and continues posterodorsal to jugal recess

1: fossa edge does not extend past the jugal recess

Note (via Brusatte et al., 2014): Those taxa without a jugal recess (e.g., most ornithomimosaurs) are here scored as inapplicable ("?") for this character.

Character 531: Jugal, pneumatic recess, location relative to ventral ramus of lacrimal:

0: ventral

1: anterior

Note (via Brusatte et al., 2014): Those taxa without a jugal recess (e.g., most ornithomimosaurs) are here scored as inapplicable (“?”) for this character.

Character 532: Jugal, pneumatic recess, orientation of long axis:

0: approximately horizontal

1: inclined at approximately 45 degrees relative to the ventral skull margin

Note (via Brusatte et al., 2014): Those taxa without a jugal recess (e.g., most ornithomimosaurs) are here scored as inapplicable (“?”) for this character.

Character 533: Jugal, secondary fossa for pneumatic recess, position relative to recess:

0: ventral

1: dorsal

Note (via Brusatte et al., 2014): Those taxa without a jugal recess (e.g., most ornithomimosaurs) are here scored as inapplicable (“?”) for this character.

Character 534: Jugal, suture with lacrimal, angle of the posterior half of the contact

0: low

1: steep

Character 535: Jugal, fossa on lateral surface of postorbital ramus, depth inset into bone:

0: shallow

1: deep

Character 536: Jugal, articulation with postorbital, form of ventral extremity of suture:

0: tapering scarf joint

1: interlocking notch for postorbital

Character 537: Jugal, articulation with postorbital, extent of scarf joint on lateral surface of postorbital ramus:

0: limited, occupies less than 50% of anteroposterior length of the process

1: extensive, occupies approximately 50-75% of the anteroposterior length of the process

Character 538: Jugal, articulation with postorbital, braced by a pronounced ridge on the lateral surface of the postorbital ramus, which borders the postorbital posteriorly:

0: no

1: yes

Character 539: Jugal, postorbital ramus, orientation relative to ventral margin of jugal:

0: approximately perpendicular

1: posterodorsal (obtuse angle between the long axis of the process and the ventral margin)

Character 540: Jugal, cornual process: (ORDERED)

0: absent

1: present

2: present and distinctive (mediolaterally wide and heavily rugose)

Character 541: Jugal, dorsal prong of quadratojugal ramus, slope in lateral view:

0: horizontal

1: posterodorsal

Character 542: Jugal, ventral prong of quadratojugal ramus, slope of joint surface in lateral view:

0: approximately anteroposteriorly oriented, angled less than 45 degrees from horizontal

1: angled anterodorsally at greater than 45 degrees from horizontal

Character 543: Jugal, shape of orbital margin:

0: weakly concave, approximately level with lacrimal-jugal suture

1: U-shaped, extends ventral to lacrimal-jugal suture

Character 544: Jugal, raised rim on the lateral surface, paralleling the ventral margin of the bone and anteriorly confluent with the antorbital fossa rim of the maxilla:

0: absent

1: present

Character 545: Postorbital, cornual process: (ORDERED)

0: absent

1: limited to rugose rim at posterodorsal corner of orbit

2: present as a rugose, convex boss

Character 546: Postorbital, cornual process, position:

0: separated from dorsal margin of postorbital by a smooth, convex region

1: approaches or extends past dorsal margin of bone

Note (via Brusatte et al., 2014): Those taxa without a cornual process are scored as inapplicable (“?”).

Character 547: Postorbital, cornual process, position:

0: located at orbital margin

1: located posterodorsal to orbital margin

Note (via Brusatte et al., 2014): Those taxa without a cornual process are scored as inapplicable (“?”).

Character 548: Postorbital, squamosal ramus, form of posterodorsal margin:

0: uninterrupted convex arc

1: emarginated by squamosal (discrete concave notch within the margin)

Character 549: Postorbital, squamosal ramus, extent relative to posterior margin of lateral temporal fenestra:

0: reaches or extends posterior to

1: terminates anterior to

Character 550: Postorbital, suborbital process, position:

0: at ventral end of ventral process

1: flange-like, separated from ventral tip of the ventral process by a notch

Note (via Brusatte et al., 2014): Taxa without a suborbital process are here scored as inapplicable (“?”).

Character 551: Postorbital, anterior ramus, form:

0: short and stout, long axis is approximately half the length of the ventral ramus and the thickness at the base is approximately the same as the thickness of the midpoint of the ventral ramus

1: long and slender, long axis is greater than 60% of the length of the ventral ramus and the thickness at the base is approximately half that of the midpoint of the ventral ramus

Character 552: Postorbital, ventral ramus, anteroposterior width at midpoint: (UNORDERED)

0: approximately the same width as ventral ramus of the lacrimal

1: substantially wider than ventral ramus of lacrimal

2: substantially narrower than ventral ramus of lacrimal

Character 553: Squamosal, lateral ridge delimiting supratemporal fossa, form:

0: ridge unpronounced or undivided

1: divided

Character 554: Squamosal, supratemporal fossa, surface morphology:

0: flat or concave

1: convex

Character 555: Squamosal, quadratojugal process, morphology of anterior tip in those taxa with horizontal processes:

0: tapered point

1: squared off

Character 556: Squamosal, quadratojugal process, flange that is covered laterally by the quadratojugal, dorsoventral depth of entire process compared to portion of process that is exposed in lateral view when in articulation with quadratojugal: (ORDERED)

0: flange absent

1: thinner

2: substantially thicker

Character 557: Squamosal, pneumaticity: posterior process, inflated by squamosal recess: (ORDERED)

0: absent

1: present as a deep, concave depression on the ventral surface of the main body

2: present as a deep, concave depression on the ventral surface of the main body, and extending posteriorly to inflate the squamosal posterior process

Note (via Brusatte et al., 2014): This is a modified version of the original character in Brusatte et al. (2010a, character 95) which includes a second derived state referring to squamosal pneumaticity in general. This character is ordered, as state 2 is a special condition of state 1 in which the pneumatic recess that invades the ventral surface of the main body of the squamosal also extends posteriorly into the posterior process of the squamosal.

Character 558: Squamosal, posterior process, length of the long axis:

0: long, approximately 1/3-1/2 length of quadratojugal process

1: short, approximately 1/6 length of quadratojugal process

Character 559: Squamosal, anterior process, flange that extends dorsal to the postorbital posterior process:

0: absent

1: present

Character 560: Quadratojugal, dorsal process, ridge along anterior margin of lateral surface: (ORDERED)

0: absent

1: present, subtle and fades in strength dorsally

2: present, robust and extends to the dorsal margin of the bone

Character 561: Quadratojugal, form of jugal articulation, dorsal prong of posterior process of jugal approaching the base of the quadratojugal (the corner where the anterior and dorsal processes of the quadratojugal meet):

0: absent

1: present

Character 562: Quadratojugal, anterior process for articulation with jugal, form of anterior region: (UNORDERED)

0: tapered

1: rounded

2: squared off or double pronged

Character 563: Quadratojugal, anterior process, extent related to anterior margin of lateral temporal fenestra:

0: terminates posterior to fenestra margin

1: level with or anterior to fenestra margin

Character 564: Quadratojugal, curvature of bone:

0: mediolaterally compressed and flat

1: posterior region flexed so that it curves posteriorly, thus delimiting the lateral edge of a deep pocket that borders the quadrate foramen laterally in posterior view

Character 565: Quadratojugal, posterior process, length and orientation:

0: short, oriented mostly laterally

1: elongate, wraps onto the posterior surface of the quadrate cotyles

Character 566: Quadrate foramen, size:

0: small, long axis approximately 10% of the dorsoventral depth of the quadrate shaft

1: large, long axis greater than 20% of the dorsoventral depth of the quadrate shaft

Character 567: Quadrate, form of pneumatization, deep recess on the anterior surface where the psterygoid wing and condyles meet:

0: absent

1: present

Character 568: Quadrate, condyles, position relative to occipital condyle when skull is in articulation:

0: aligned (i.e., quadrate condyles approximately ventral to occipital condyle, or slightly anterior to condyle)

1: completely posterior

Character 569: Quadrate, quadratojugal articulation, extent on lateral surface of lateral condyle:

0: limited, occupies only part of the surface

1: extensive, covers entire lateral surface and extends dorsally to partially enclose quadrate foramen laterally

Character 570: Quadrate, articular surface for quadratojugal on quadrate lateral condyle, orientation of medial margin as seen in posterior view where quadratojugal wraps around quadrate:

0: vertical or dorsomedial

1: dorsolateral

Character 571: Prefrontal, contacts nasal:

0: yes

1: no, excluded by frontal-lacrimal contact

Character 572: Prefrontal, ventral process, extent:

0: large, extends 1/2-1/4 of the way down the ventral ramus of the lacrimal to make an extensive contribution to the preorbital bar

1: reduced or absent, ventral process is a thin flange that is continuous with the crista cranii of the frontal, and does not extend more than approximately ¼ of the length of the preorbital bar

Character 573: Frontal, shape (in those taxa with frontals that narrow anteriorly as a wedge between the nasals):

0: triangular

1: posterior end expanded into a rectangular shape, with a small anterior triangle

Note (via Brusatte et al., 2014): Taxa with frontals that do not narrow anteriorly between the nasals (scored in character 41) are scored here as inapplicable ("?").

Character 574: Frontal, size of single frontal, ratio of anteroposterior length of exposed portion on skull roof to mediolateral width at midpoint:

0: greater than 2.0 (usually greater than 2.5)

1: less than 2.0

Character 575: Frontal, supratemporal fossa, medial extension:

0: fossa restricted to posterolateral corner of frontal

1: meets opposing fossa at the midline

Character 576: Frontal, sagittal crest: (UNORDERED)

0: absent or subtle, only discernable as a slight midline bulge

1: present and pronounced (dorsoventrally tall), single structure

2: present and pronounced (dorsoventrally tall), paired structure

Note (via Brusatte et al., 2014): This character is not equivalent to a parietal sagittal crest. Rather, it refers specifically to raised crests on the dorsal surface of the frontal, which may or may not be continuous with a sagittal crest on the parietal.

Character 577: Frontal, sagittal crest, anteroposterior length:

0: short, less than 15% length of the frontal

1: extensive, approximately 25% of the length of the frontal

Note (via Brusatte et al., 2014): Those taxa without a frontal sagittal crest are scored as inapplicable (“?”).

Character 578: Frontal, postorbital suture: (ORDERED)

0: dorsoventrally shallow and undifferentiated

1: dorsoventrally shallow (approximately 6 times longer than deep) and differentiated into a vertical region anteriorly and a horizontal region posteriorly

2: dorsoventraly deep (approximately twice as long as deep) and subtly differentiated into vertical and horizontal regions

Character 579: Frontal, contribution to orbital rim: (ORDERED)

0: extensive

1: present but limited to a small notch

2: excluded by postorbital-lacrimal contact in large specimens

3: exclueed by postorbital-lacrimal articulation and oval “palpebral” ossification

Character 580: Parietal-frontal suture, form:

0: transversely smooth

1: tab-like wedge from parietal extends anteriorly to overlie frontal on mindline

Character 581: Parietal, sagittal crest, form:

0: comprised of two parallel crests

1: comprised of a single midline crest

Note (via Brusatte et al., 2014): Those taxa without a parietal sagittal crest are here scored as inapplicable (“?”).

Character 582: Parietal, skull table between supratemporal fossae, width:

0: broad, more than 10% of the mediolateral width of the fossa

1: extremely reduced, sagittal crest or crests (if present) pinched between opposing fossae

Character 583: Parietal, sagittal crest, dorsoventral depth:

0: consistent across length of crest

1: peaked anteriorly at frontal-parietal suture

Note (via Brusatte et al., 2014): Those taxa without a parietal sagittal crest are here scored as inapplicable (“?”).

Character 584: Parietal, nuchal crest, dorsoventral depth:

0: as low as or lower than dorsal surface of the interorbital region

1: extends higher than the dorsal surface of the interorbital region

Character 585: Vomer, shape of anterior end:

0: lanceolate (lateral margins parallel-sided)

1: expanded into a diamond

Character 586: Ectopterygoid, extent of internal recess:

0: does not inflate body of the bone and the pterygoid process

1: inflates body of the bone and the pterygoid process

Character 587: Ectopterygoid, jugal process, external pneumatic foramina leading into ectopterygoid recess:

0: absent

1: present

Character 588: Ectopterygoid, jugal process, pneumaticity:

0: is not inflated by the internal recess

1: is visibly inflated by the internal recess

Character 589: Ectopterygoid, external opening of pneumatic recess, shape:

0: thin ovoid slot

1: large, round or triangular

Character 590: Ectopterygoid, surface posteriorly adjacent to external opening of pneumatic recess, form:

0: flat, recess grade smoothly into the floor of the lateral temporal fenestra (=subtemporal fenestra)

1: lip, recess separated from lateral temporal fenestra (=subtemporal fenestra)

Character 591: Palatine, vomeropterygoid process, ratio of anteroposterior length of dorsal margin to length of greatest constriction of process neck:

0: greater than 2.0

1: less than 2.0

Character 592: Palatine, vomeropterygoid process, orientation of neck:

0: inclined anterodorsally

1: vertical

Character 593: Palatine, pneumaticity:

0: absent

1: present

Character 594: Palatine, pneumatic recess, number of external pneumatic openings:

0: one

1: two

Character 595: Palatine, primary external opening of palatine recess, location of posterior margin:

0: level with or extends posterior to posterior margin of the vomeropterygoid process neck

1: located far anterior to posterior margin of the vomeropterygoid process neck

Character 596: Palatine, primary opening of palatine recess, location of anterior margin:

0: level with or extends posterior to anterior margin of the vomeropterygoid process neck

1: located far anterior to anterior margin of the vomeropterygoid process neck

Character 597: Palatine, jugal process, location of contact surface for lacrimal:

0: posterior (“distal”), separated from opening of palatine recess by wide margin

1: anterior (“proximal’), closely approaches opening of palatine recess

Character 598: Palatine, maxillary process, form of maxillary articulation:

0: flat

1: deeply excavated as a slot, demarcated dorsally by a pronounced lip of bone

Character 599: Palatine, extension of pneumatic recess into jugal process:

0: no

1: yes, process visibly inflated

Character 600: Palatine, maxillary articulation, form:

0: maxilla abuts lateral surface of maxillary process and anterior region of jugal process

1: contact reinforced by a “brace” at the anteroventral corner of the jugal process, which sits within internal antorbital fossa

Character 601: Palatine, morphology of maxillary articulation brace:

0: projects ventrally due to a jugal process that extends further ventrally than the maxillary process, such that there is a discrete corner between the two processes in lateral view

1: projects laterally, with no discrete corner between the smoothly confluent jugal and maxillary processes in lateral view

Character 602: Internal choana, shape:

0: anteroposteriorly elongate oval

1: nearly circular

Character 603: Suborbital fenestra, shape:

0: anteroposteriorly elongate oval

1: nearly circular

Character 604: Braincase, orientation of occipital surface:

0: faces posteriorly

1: faces posteroventrally

Character 605: Supraoccipital, contribution to dorsal rim of foramen magnum (ORDERED)

0: forms entire rim

1: makes limited contribution to rim via triangular ventral process

2: completely excluded from rim

Character 606: Supraoccipital, form of dorsal margin

0: smoothly convex and undivided

1: divided into two processes (“forked”)

Character 607: Exoccipital-opisthotic, paroccipital process, ventral flange at distal end:

0: absent

1: present

Character 608: Exoccipital-opisthotic, paroccipital processes, deep fossa on posterior surface dorsolateral to the foramen magnum:

0: present

1: absent

Character 609: Exoccipital-opisthotic, crista tuberalis (=metotic strut), extent in posterior view:

0: limited, mediolateral width across opposing cristae less than one half the dorsoventral depth of the braincase from the dorsal tip of the supraoccipital to the ventral tip of the basal tubera

1: extensive, width greater than one half braincase depth

Character 610: Basioccipital, basal tubera, dorsoventral depth:

0: less than depth of occipital condyle

1: greater than depth of occipital condyle

Character 611: Basioccipital, basal tubera, concave notch ventrally between opposing tubera, dorsoventral depth:

0: shallow, less than 40% depth of tubera

1: deep, approximately 50% depth of tubera

Character 612: Basioccipital, subcondylar recess, depth of pneumatic fossae on posterior surface of basal tubera:

0: absent or shallow

1: deep

Character 613: Basisphenoid, basisphenoid recess, orientation of central axis: (ORDERED)

0: vertical, recess obscured in posterior view

1: posteroventral, recess partially visible in posterior view

2: extremely posteroventral, recess compressed anteroposteirorly and widely visible in posterior view, and basipterygoid processes located beneath the basal tubera

Character 614: Basisphenoid, basisphenoid recess, inflation of the ceiling of the recess:

0: absent

1: present

Character 615: Basisphenoid, basisphenoid recess, shape in ventral view:

0: funnel-like, expands in mediolateral width posteriorly

1: ovoid or circular, no posterior expansion

Character 616: Basisphenoid, shape of basicranium (rectangle defined by positions of both basal tubera and both basipterygoid processes):

0: anteroposteriorly longer than mediolaterally wide

1: wider than long

Character 617: Parasphenoid, shape of rostrum:

0: anteroposteriorly expanded, ventral margin is a smooth concave arch

1: dorsoventrally expanded, ventral margin is nearly vertical posteriorly and then abruptly transitions to horizontal trend anteriorly

Character 618: Laterosphenoid, antotic crest separating lateral wall of braincase from orbital and temporal spaces:

0: absent or indistinct

1: present, robust and rugose

Character 619: Laterosphenoid, antotic crest, form:

0: single structure

1: bifurcates ventrally

Character 620: Laterosphenoid, fossa on lateral surface that houses head of epipterygoid:

0: absent or shallow

1: present, deep and rugose

Character 621: Mandibular ramus, dorsoventral depth of dentary at level of dentary-surangular contact on the dorsal margin of the lower jaw:

0: less than 18% of the total anteroposterior length of the lower jaw

1: greater than 18% of the total anteroposterior length of the lower jaw

Character 622: External mandibular fenestra, dorsoventral depth relative to depth of mandible at midpoint of fenestra: (ORDERED)

0: greater than 25% depth of mandible

1: less than 25% depth of mandible

2: absent

Character 623: Lower jaw, articulation, glenoid position relative to level of alveolar margin of dentary: (UNORDERED)

0: approximately level with

1: dorsal to

2: strongly ventral to

Character 624: Dentary, position of the transition point between the ventral and anterior margins of the bone in lateral view:

0: below alveoli 1-3, anterior margin of bone rounded (or in some cases nearly straight)

1: below alveolus 4 (or more posteriorly), anterior margin nearly straight and projects posteroventrally

Character 625: Dentary, ventrally projecting rugose process ("chin") where the anterior and ventral margins of the dentary meet:

0: absent

1: present, visible as a pointed projection in lateral view and convex in medial view, braces dentary symphysis

Character 626: Dentary, symphysis, texture:

0: generally smooth

1: strongly rugose and beveled, with interlocking ridges and convexities for articulation with the opposing symphysis

Character 627: Dentary, articular surface for splenial along ventral region of dentary ramus below the Meckelian fossa, form:

0: dorsoventrally shallow and smooth

1: dorsoventrally deep (nearly as deep as anterior depth of fossa) and rugose

Character 628: Dentary, anterior alveoli, size in comparison to alveoli in middle of tooth row: (ORDERED)

0: approximately same size

1: first two alveoli substantially smaller

2: first alveolus substantially smaller

Note (via Brusatte et al., 2014): This character is ordered here, because we hypothesize that state 2 (which is seen only in a small subset of derived tyrannosaurids: *Tarbosaurus* and *Tyrannosaurus*) is a special condition of state 1 (which is present in all score-able tyrannosauroids except for the derived subset).

Character 629: Dentary, dorsal margin of bone in lateral view, profile: (UNORDERED)

0: straight

1: strongly concave

2: strongly convex

Character 630: Dentary, Meckelian groove, form:

0: dorsoventrally deep and shallowly inset into medial surface of bone

1: dorsoventrally shallow and deeply inset into bone, groove appears as a thin, sharp structure

Character 631: Surangular, surangular shelf on lateral surface, form: (ORDERED)

0: low ridge or absent

1: prominent ridge that is offset laterally from the bone but dorsoventrally thin

2: prominent shelf that is dorsoventrally deep

Note (via Brusatte et al., 2014): The "low ridge" and "absent" conditions are considered primarily homologous here, because a very low ridge may appear absent on a specimen due to poor preservation. Taxa that genuinely appear to lack a ridge (e.g., *Shuvuuia*) are scored as inapplicable ("?") for the following characters related to the form and the position of the ridge.

Character 632: Surangular, surangular shelf on lateral surface, position and form: (ORDERED)

0: placed far dorsal to posterior surangular foramen

1: foramen abuts shelf but shelf projects laterally and does not overhang foramen

2: shelf projects ventrolaterally to overhang foramen

Character 633: Surangular, surangular shelf on lateral surface, orientation relative to the long axis of the lower jaw: (UNORDERED)

0: anterodorsal

1: anteroventral

2: straight anteroposteriorly

Character 634: Surangular, pneumatic fossa posterodorsal to posterior surangular foramen:

0: absent

1: present

Character 635: Surangular, adductor muscle attachment site dorsal to surangular shelf, orientation: (ORDERED)

0: faces primarily dorsally

1: faces almost equally dorsally and laterally

2: faces primarily laterally

Character 636: Surangular, triangular fossa on the lateral surface of the surangular shelf immediately anteroventral to glenoid:

0: absent

1: present

Character 637: Surangular, fossa on the lateral surface of the bone immediately ventral to, and separated from, the glenoid:

0: absent

1: present

Character 638: Surangular, anteroposterior length of anterior flange (region anterior to anterior margin of external mandibular fenestra) compared to overall length of surangular:

0: less than 30%

1: greater than 30%

Character 639: Angular, ventral margin, form:

0: smoothly convex

1: anterior region "flexed" relative to posterior region, such that there is a discrete step between them

Character 640: Articular, mediolateral width of jaw muscle attachment site:

0: less than width of glenoid for articulation with quadrate

1: approximateily equal to width of glenoid

Character 641: Articular, smooth non-articular region between glenoid and attachment site for depressor mandibular muscles:

0: present

1: absent

Character 642: Splenial, anterior mylohoid foramen, shape and size: (ORDERED)

0: small circular or ovoid opening, or absent

1: large, anteroposteriorly ovoid shape

2: extremely large, approximately as deep dorsoventrally as the anterior process of the splenial

Note (via Brusatte et al., 2014): The small and absent conditions are here treated as a single character state, because poor preservation often makes a very small foramen appear absent.

Character 643: Splenial, dorsal region overlapped medially by prearticular:

0: absent

1: present

Character 644: Prearticular, ventral bar, series of ridges on lateral surface to strengthen articulation with angular:

0: absent

1: present

Character 645: Supradentary ossification, shape:

0: elongate, shallow strip

1: deep, crescentic shape

Character 646: Supradentary and coronoid ossifications, form of contat at their zone of fusion:

0: ossifications smoothly confluent

1: ossifications offset by a concave notch

Character 647: Premaxillary tooth crown 4, apicobasal height relative to largest maxillary crown:

0: subequal

1: approximately 50%

Character 648: Premaxillary teeth, median vertical ridge on lingual surface: (ORDERED)

0: absent

1: present as a subtle structure in anterior (mesial) premaxillary teeth

2: present as pronounced structure in all premaxillary teeth

Character 649: Premaxillary teeth, curvature of distal (posterior) teeth:

0: recurved

1: straight

Character 650: Maxillary teeth, number:

0: 13 or more

1: less than 13 (in the largest adult specimens when growth series are known)

Character 651: Maxillary and dentary teeth, form: (ORDERED)

0: ziphodont, transverse width of base less than 60% of mesiodistal length

1: incrassate, width greater than 60% of length

2: incrassate, width nearly equal to length

Note (via Brusatte et al., 2014): Those taxa with conical or leaf-like teeth are scored as inapplicable ("?") for this character (e.g., therizinosauroids, most alvarezsauroids).

Character 652: Axis and postaxial cervicals, anteroposterior length of centrum compared to dorsoventral height of posterior centrum face:

0: greater

1: less than or equal to

Character 653: Axis, pneumatic foramen (pleurocoel), position:

0: near midheight of entrum

1: dorsally located, directly underneath neurocentral suture and directly posterior to diapophysis

Character 654: Axis, pneumatic foramen (pleurocoel), extent of surrounding fossa:

0: limited to margins of foramen

1: extensive, occupies most of lateral surface of centrum

Character 655: Axis, ridge on ventral surface of centrum:

0: absent

1: present

Character 656: Axis, pneumatic foramina and fossae on each side of the anterior ridge on the neural spine:

0: absent

1: present

Character 657: Axis, neural spine, texture of dorsal region of anterior surface:

0: generally smooth or with subtle texture

1: highly rugose, with series of grooves, ridges, and eminences

Character 658: Axis, dorsal region of neural spine, number of projections on "crown" region:

0: two lateral projections, dorsal surface of spine smoothly concave

1: two lateral projections and one dorsal projection on the midline

Note (via Brusatte et al., 2014): Only those taxa scored for a mediolaterally broad ("transversely flared") neural spine for character 92 are relevant to this character. All taxa with a mediolaterally compressed neural spine are scored as inapplicable ("?").

Character 659: Axis, supradiapophyseal fossa (fossa posterodorsal to diapophysis), form:

0: absent or shallow

1: deeply excavated and funnel-like

Character 660: Cervical vertebrae, neural spines in middle-posterior cervicals, dorsoventral height:

0: substantially shorter than height of posterior centrum face

1: approximately same length as or longer than height of posterior centrum face

Character 661: Cervical vertebrae, morphology of posterior centrodiapophyseal laminae in anterior-middle cervicals:

0: absent or present as a weak ridge

1: present as a thick, laterally offset lamina that demarcates a deep infradiapophyseal fossa anteriorly

Character 662: Cervical vertebrae, hypapophysis on anterior region of ventral surface:

0: absent

1: present

Character 663: Cervical vertebrae, position of prezygapophysis in middle cervicals:

0: slightly overhangs centrum laterally

1: strongly overhangs centrum laterally, entire prezygapophyseal facet placed lateral to centrum

Character 664: Cervical vertebrae, orientation of posterior centrodiapophyseal lamina in anterior-middle cervicals:

0: projects posteroventrally, infrapostzygapophyseal fossa located primarily posterior to lamina

1: nearly horizontal, fossa located primarily dorsal to lamina

Character 665: Cervical and dorsal vertebrae, rugose ligament attachment scars in pre- and postspinal fossae: (ORDERED)

0: absent or weakly developed

1: present as prominent, rectangular flanges that extend outside of the fossae and are visible in posterior view, but on the dorsal vertebrae only

2: prominent in dorsals and cervicals

Character 666: Dorsal vertebrae, neural spine, level of posterior termination:

0: at approximately the same level as the posterior centrum face

1: far posterior to the posterior centrum face

Character 667: Dorsal vertebrae, anteroposterior length of middle-posterior dorsal centra compared to dorsoventral height of posterior centrum face:

0: greater

1: less than or equal

Character 668: Dorsal vertebrae, middle-posterior dorsals, position of postzygapophysis relative to prezygapophysis:

0: at same level

1: elevated dorsally

Character 669: Dorsal vertebrae, middle-posterior dorsals, form of anterior and posterior centrodiapophyseal laminae: (ORDERED)

0: discrete laminae absent, or laminae present but do not demarcate a deep infradiapophyseal fossa between them

1: present and make contact on ventral surface of transverse process, demarcating a triangular infradiapophyseal fossa

2: present and do not make contact but roughly parallel each other, infraprezygapophyseal and infradiapophyseal fossa merged into a single fossa

Character 670: Sacral vertebrae, fenestrae between fused neural spines: (ORDERED)

0: neural spines unfused

1: spines fused but fenestrae absent

2: spines fused and fenestrae present

Character 671: Sacral ribs, position of central ribs on sacrum:

0: span two sacrals

1: limited to a single sacral

Character 672: Sacral ribs, position of rib attachment for central ribs on individual sacrals:

0: span centrum and neural arch

1: limited to neural arch only

Character 673: Sacral vertebra five, position of ventral margin of posterior articular face in lateral view:

0: at same level as ventral margin of anterior articular face

1: positioned ventral to ventral margin of anterior articular face

Character 674: Sacral vertebrae, form of hyposphene in posteriormost sacral:

0: absent or present as a single midline structure

1: present and comprised of two parallel-sided sheets

Character 675: Caudal vertebrae, anterior caudals, position of base of neural spine:

0: anterior to posterior surface of centrum

1: level with or posterior to posterior surface of centrum

Character 676: Caudal vertebrae, anterior caudals, shape of transverse processes in dorsal view:

0: rectangular, with parallel anterior and posterior sides, or slightly ovoid with a gradual expansion in width distally

1: distal end expanded into a spatulate bulb

Character 677: Caudal vertebrae, anterior caudals, two laminae linking prezygapophysis and transverse process, between which is a deep, triangular fossa:

0: absent

1: present

Character 678: Scapula, angle between posterior margin of glenoid and dorsal margin of blade:

0: greater than 90 degrees

1: approximately 90 degrees

Character 679: Scapula, acromion, dorsoventral depth:

0: less than 3.0 times minimum dorsoventral depth of blade

1: greater than 3.0 times minimum dorsoventral depth of blade

Character 680: Scapula, ratio of anteroposterior length of bone to minimum dorsoventral depth of blade:

0: less than 10.0

1: greater than 10.0

Character 681: Scapula and coracoid, glenoid, position relative to posteroventral margin of blade:

0: offset posteroventrally (by a distance equivalent to the width of the neck of the blade to ½ of the width of neck of the blade)

1: offset only slightly posteroventrally (less than 50% the width of the neck of the blade)

Character 682: Coracoid, anteroposterior length at midpoint:

0: approximately 100-150% of the length of the scapular acromion at midheight

1: 200% or greater than the length of the scapular acromion at midheight

Character 683: Coracoid, coracoid foramen:

0: present

1: absent or extremely small

Character 684: Humerus, apex of deltopectoral crest, location from proximal end: (ORDERED)

0: 35-50% of the length of the humerus

1: 25-35% of the length of the humerus

2: less than 25% of the length of the humerus

Character 685: Humerus, additional muscle attachment tubera at the corner of the anterior and lateral surfaces distal to the deltopectoral crest:

0: absent

1: present

Character 686: Humerus, concave notch between external tuberosity and deltopectoral crest:

0: present, two structures clearly separated

1: absent, two structures smoothly confluent

Character 687: Humerus, form of distal condyles:

0: lateral and medial condyles expanded equally (offset from shaft in anterior or posterior view is approximately equal)

1: medial condyle expanded further medially than the lateral condyle is laterally

Character 688: Ulna, shaft axis, form:

0: bowed

1: straight

Character 689: Principal distal carpal, shape:

0: semilunate in lateral view with trochlear proximal surface

1: discoid with flat proximal surface

Character 690: Metacarpal I, medial distal condyle, form:

0: well formed and large

1: rudimentary

Character 691: Metacarpal I, medial margin, shape in proximal view:

0: concave

1: smoothly convex or straight

Character 692: Metacarpals, metacarpal II, mediolateral width at midpoint compared to midpoint width of metacarpal I:

0: equal to or narrower than

1: more robust than

Character 693: Manual phalanx II-1, length compared to that of metacarpal 1:

0: longer

1: subequal to

Character 694: Ilium, anteroposterior length compared to length of femur: (ORDERED)

0: 70-85%

1: 95-105%

2: 105-115% (or greater, in some birds)

Character 695: Ilium, dorsal margin of blade, position relative to sacral neural spines: (UNORDERED)

0: separated by a gap

1: lies against neural spines and opposing iliac blaces may make contact above neural spines in some individuals

2: separated by a wide gap

Note (via Brusatte et al., 2014): This is a modified version of the Brusatte et al. (2010a) character, which adds a second derived state ("separated by a wide gap") to refer to the condition of some derived therizinosauroids, as used by Zanno et al. (2009, character ZCD 306).

Character 696: Ilium, supraacetabular crest, maximum lateral projection relative to ischial peduncle:

0: significantly greater

1: subequal

Character 697: Ilium, supraacetabular crest, extent on pubic peduncle:

0: extensive, extends along most or all of the edge of the peduncle

1: limited, discretely offset from acetabular edge of pubic peduncle

Character 698: Ilium, pubic and ischial peduncles, anteroposterior lengths at dorsal base: (ORDERED)

0: pubic peduncle substantially longer than ischial peduncle

1: both peduncles approximately the same length

2: ischial peduncle longer than pubic peduncle

Character 699: Ilium, ventral margin of postacetabular process, shape:

0: straight to slightly convex

1: hightly convex, forming a discrete “lobe”-like flange

Character 700: Ilium, dorsal margin, shape:

0: smoothly convex or straight across entire length

1: convex anteriorly and straightens out posteriorly

Note (via Brusatte et al., 2014): In dromaeosaurids such as *Unenlagia*, which possess a separate posterior tab-like process of the postacetabular process, this character refers to the shape of the remainder of the ilium (i.e., the entirety of the bone except the tab-like process). Therefore, these taxa are scored for state 0.

Character 701: Ilium, ratio of anteroposterior length to dorsoventral depth above acetabulum:

0: greater than 3.0, ilium is long and low

1: less than 2.8, ilium is subovoid in shape

Character 702: Pubis, pubic tubercle: (ORDERED)

0: absent

1: present as a convexity on the anterior margin of the pubis

2: present as a rugose flange that is discretely offset from the anterior margin of the pubis and is bordered posteriorly by heavy rugosities on the lateral surface on the obturator region of the pubis

Character 703: Pubis, pubic tubercle, position:

0: distally positioned, located ventral to the level of the obturator notch

1: proximally positioned, located level with or dorsal to the obturator notch

Note (via Brusatte et al., 2014): All taxa without a pubic tubercle are here scored as inapplicable (“?”).

Character 704: Pubis, pubic boot, anteroposterior length relative to total long axis length of pubis:

0: less than 60%

1: greater than 60%

Character 705: Pubis, pubic boot, position of anterior process relative to posterior process:

0: displaced dorsally, resulting in a highly convex ventral margin of the boot

1: placed at the same level, ventral margin of the boot essentially straight

Note (via Brusatte et al., 2014): Taxa without a discrete anterior process (e.g., compsognathids) are here scored as inapplicable (“?”).

Character 706: Pubis, anteroposterior expansion of proximal obturator plate region relative to the anterior edge of the pubis shaft at its midpoint:

0: less than twice the anteroposterior thickness of the shaft at its midpoint

1: greater than twice the anteroposterior thickness of the shaft at its midpoint

Character 707: Pubis, obturator notch, form:

0: discrete structure, demarcated ventrally by extensive obturator flange

1: essentially absent, no ventral flange

Note (via Brusatte et al., 2014): The enclosed obturator foramen of *Mirischia* is here scored as equivalent to state 0. The presence of a completely enclosed foramen is autapomorphic of *Mirischia* among coelurosaurs.

Character 708: Ischium, position of medial apron:

0: along posterior margin of shaft

1: along anterior margin of shaft

Character 709: Femur, circular scar on posterior surface of shaft distal to fourth trochanter, position:

0: absent, low, or positioned approximately centrally on the shaft

1: abuts medial edge of shaft

Character 710: Femur, lesser trochanter, height relative to greater trochanter:

0: shorter, terminates further distally

1: subequal or slightly taller, the two structures extend to approximately the same level proximally

Note (via Brusatte et al., 2014): This is equivalent to Zanno et al. (2009: character ZCD 325).

Character 711: Femur, proximal margin in anterior view: (ORDERED)

0: approximately straight and perpendicular to the long axis of the shaft

1: approximately straight and oriented at an obtuse angle to the long axis of the shaft (=dorsally or proximally inclined head)

2: concave and oriented at an obtuse angle to the long axis of the shaft, due to head that is proximally inclined and a greater trochanter that is elevated substantially relative to the central portion of the proximal surface of the femur

Note (via Brusatte et al., 2014): Characters relating to the inclination of the femoral head are often used in theropod phylogenetic datasets, but it is often difficult to develop a standard system of measurement for quantifying the orientation of the head. We use the character as defined here, which concerns the angle between the long axis of the shaft and the long axis of the proximal end, as measured in anterior view (note that many taxa with obtuse angles between the head and shaft in anterior view, such as *Tyrannosaurus*, appear to have perpendicular angles when measured in posterior view). Usually, taxa with an obtuse angle between head and shaft (i.e., a dorsally inclined femoral head) have a proximomedially projecting proximal surface of the femur when seen in anterior view, and this may indicate the presence of an inclined head in specimens that do not preserve the shaft. Furthermore, taxa with an obtuse angle between head and shaft (a dorsally inclined femoral head) possess a head that is elevated proximally relative to the greater trochanter, which is another means for recognizing the inclined condition in specimens that do not preserve the shaft. We also recognize a second derived state, encoded by Zanno et al. (2009: character oZCD 339) and Brusatte et al. (2010a: character 285), referring to the deeply concave proximal margins of the derived tyrannosaurids *Tyrannosaurus* and *Tarbosaurus* and some derived therizinosauroids, which have dorsally inclined heads but also greater trochanters that are raised substantially relative to the remainder of the proximal femur (except for the head), giving the proximal margin a deeply concave profile in posterior view. This character is equivalent to Zanno et al. (2009, character oZCD 320), which refers to the distinction between a medially ("perpendicular to shaft") and dorsally inclined femoral head. Brusatte et al. (2012a) stated that *Tanycolagreus* possesses a dorsally inclined (elevated) femoral head, but we here note that this is not correct and that this taxon possesses a head that is perpendicular to the shaft (state 0).

Character 712: Femur, trochanteric fossa on the posterior surface of the head, lateral to the ligament sulcus (for the capital ligament), form: (ORDERED)

0: absent or shallow

1: deep fossa

2: deep, extensive triangular depression that covers most of the posterior surface of the femur proximally and is demarcated medially and ventrally by a pronounced, curving, swollen ridge

Character 713: Femur, fourth trochanter, position, measurement from proximal margin of head to distal termination of trochanter relative to total length of the femur:

0: 40% or less

1: greater than 40%

Character 714: Femur, lateral condyle, shape in distal view:

0: circular or ovoid

1: ovoid, but with an anterior bulge that is slightly separated from the remainder of the condyle

Character 715: Femur, extensor groove on anterior surface of distal end, form: (ORDERED)

0: absent or extremely shallow, anterior surface flat between the condyles in distal view (extensor groove may be present but does not manifest itself as a groove on the anterior margin in distal view)

1: groove present but shallow, expressed as a broad concave margin in distal view but present as an extensive depression on the anterior surface of the femur

2: groove present and deep, expressed as a deep, U-shaped cleft in distal view and present as an extensive depression on the anterior surface of the femur

Character 716: Femur, mesiodistal crest, form:

0: single structure

1: bifurcates distally to enclose fossa on the medial surface of the medial condyle

Character 717: Tibia, length relative to the femur:

0: 1.05 or greater

1: less than 1.00

Character 718: Tibia, lateral condyle of proximal end, anterior process:

0: absent

1: present

Character 719: Tibia, lateral malleolus, lateral extent:

0: limited, mediolateral measure is less than 40% of mediolateral width of adjacent shaft

1: extensive, mediolateral measure greater than 40% of mediolateral width of adjacent shaft

Character 720: Tibia, lateral malleolus, position relative to medial malleolus:

0: extent to approximately the same level distally

1: lateral malleolus extends substantially further distally than medial malleolus

Character 721: Fibula, iliofibularis tubercle, form:

0: single crest

1: large, rugose, and formed by two crests separated by a depressed fossa ("bipartite" condition)

Character 722: Astragalus, fossa on anterior surface of ascending process, form:

0: shallow concavity that covers most of the ventral region of the ascending process

1: deep, triangular or ovoid fossa immediately above midpoint of condyles, set within a broad fossa that covers most of the ventral region of the ascending process

Character 723: Pes, metatarsal III, form of medial surface in anterior or posterior view:

0: straight or subtly convex

1: with medial convex expansion forming a bulge along the distal part of the shaft

Note (via Brusatte et al., 2014): This character is equivalent to L. Xu et al. (2011: character 48), who utilized it as a character relevant to ornithomimosaurs.

Character 724: Pes, metatarsal III, ventral nonarticular surface (on the flexor surface) immediately proximal to the distal condyles, form:

0: concave

1: raised subtriangular platform

Character 725: Pes, metatarsals II-IV, distal separation when in articulation:

0: metatarsals closely appressed and distance between II-III and III-IV is approximately equal

1: distal ends of II and IV diverge from III, and distance between III-IV greater than that between II-III

Character 726: Pes, metatarsal II, articular scar for metatarsal III on distal portion of lateral surface of shaft, form:

0: subtle or absent

1: enlarged as a rugose fossa that occupies more than half of the proximodistal length of the shaft and expands in anteroposterior width distally

Character 727: Pes, metatarsal II, lateral surface in proximal view, shape: (ORDERED)

0: flat or weakly concave

1: moderately concave

2: strongly concave (deep concave notch is present)

Note (via Brusatte et al., 2014): This is an expanded version of the original Brusatte et al. (2010a, character 304) character, which adds a new derived state to take into account the subtly concave condition in some ornithomimosaurs, which differs from the flat condition in outgroups and many basal coelurosaurs (e.g., *Ornitholestes*) and the deeply concave notched condition of derived tyrannosaurids.

Character 728: Pes, metatarsal IV, distal end, ratio between anteroposterior long axis (measured from midpoint of condyles posteriorly to anterior surface of bone) and mediolateral width (measured at midpoint: (ORDERED)

0: greater than 1.40, distal surface is elongate anteroposteriorly

1: between 1.40 and 1.20

2: less than 1.20, distal surface nearly square shaped with nearly flat anterior surface

Character 729: Pes, proximal pedal phalanges of digits II and III, ratio of length to midshaft width:

0: greater than 3.0

1: less than 3.0

Character 730: Pes, pedal unguals, lip overhanging proximal articular surface dorsally (on extensor surface):

0: present

1: absent or reduced to a subtle tuber

***Characters Relevant to Ornithomimosauria (from L. Xu et al. 2011):**

Character 731: Maxilla, series of discrete foramina along ventral edge of lateral surface:

0: present

1: absent

Character 732: Lacrimal, prominence on lateral surface of bone:

0: absent

1: present

Character 733: Dentary, morphology of dorsal border in transverse cross section:

0: rounded and lacks “cutting edge”

1: sharp with a “cutting edge”

Character 734: Surangular, foramen on dorsal edge of bone dorsal to mandibular fenestra (anterior surangular foramen):

0: present

1: absent

Character 735: Neck, length compared to that of skull:

0: less than twice skull length

1: greater than twice skull length

Character 736: Coracoid, biceps tubercle, position:

0: positioned close to base of posterior process (closer to coracoid-scapula suture than to anterior edge of coracoid)

1: positioned more anterior than the base of the posterior process (closer to anterior edge of coracoid than to coracoid-scapula suture)

Character 737: Coracoid, position of infraglenoid buttress relative to that of posterior process when bone is seen in dorsal view:

0: two structures extend to same level laterally

1: buttress offset laterally from posterior process

Character 738: Metacarpal II, length compared to metacarpal III:

0: shorter

1: equal to or longer

Character 739: Manual digit I, length of phalanx I-1 compared to metacarpal II:

0: shorter

1: longer

Character 740: Manual unguals, flexor tubercles, position:

0: near proximal end

1: distal to proximal end

Note (via Brusatte et al., 2014): Those alvarezsauroids without discrete flexor tubercles are scored as inapplicable ("?").

Character 741: Pubis, shape of ventral margin of pubic boot:

0: straight or slightly convex

1: strongly convex with ventral expansion

Character 742: Pes, pedal digit I:

0: present

1: absent

Character 743: Pes, phalanx II-2, length:

0: more than 60% of length of pedal phalanx II-1

1: less than 60% of length of pedal phalanx II-1

Character 744: Pubis, angle between anterior process of pubic boot and shaft:

0: greater than 90 degrees

1: approximately 90 degrees

Character 745: Pubis, position of anterior edge of anterior process of pubic boot:

0: approximately same level as anterior margin of pubic shaft

1: markedly anterior to the anterior margin of pubic shaft

Character 746: Pes, pedal unguals, shape:

0: curved in lateral view

1: straight

***Characters Relevant to Basal Coelurosauria (from Li et al. 2010):**

Character 747: Premaxillary teeth, size compared to mesial (anterior) maxillary teeth:

0: approximately same size, slightly smaller, or larger

1: considerably smaller

Character 748: Ischium, form of articulation with ilium on proximal surface of iliac peduncle:

0: approximately flat or slightly concave

1: deeply concave as a deep socket to receive a peg-like ischial peduncle of the ilium

Note (via Brusatte et al., 2014): Some tyrannosauroids and ornithomimosaurs have been described as possessing an accessory peg-like structure on the distal edge of the ischial peduncle of the ilium, which fits into a deep socket on the ischium (e.g.., *Alioramus*, *Gallimimus*: Brusatte et al. 2012a). Because it is often difficult to determine the presence or absence of this subtle peg-like process, we here consider the peg-like process and a peg-like ischial peduncle itself as primarily homologous. The presence of either of these processes can be clearly determined by the presence of a funnel-like socket on the iliac peduncle of the ischium. Because both structures leave a similar mark on the ischium, this supports the hypothesis of their primary homology (at least at some level).

Character 749: Scapula, acromion process, shape:

0: much deeper dorsoventrally than long anteroposteriorly, generally tapering or triangular in shape

1: approximately as long as or longer anteroposteriorly than deep dorsoventrally, with short reach beyond scapular blade and squared-off profile

Character 750: Basisphenoid, pronounced muscle scars flanking basisphenoid recess:

0: absent

1: present

Character 751: Ilium, form of distal articular surface of pubic peduncle:

0: convex

1: flat or concave (notched)

***Characters Relevant to Alvarezsauroidea (from Choiniere et al. 2010a,b, 2012 and Choiniere, pers. comm.):**

Character 752: Lacrimal, orientation of ventral ramus relative to the long axis of the alveolar margin of the upper jaw when the articulated skull is seen in lateral view

0: approximately vertical or inclinded slightly anteroventrally

1: inclined strongly posteroventrally

Character 753: Exoccipital-opisthotic, position of ventral edge of the base of the paroccipital process:

0: level with or dorsal to the dorsal border of the occipital condyle

1: situated at mid-height of occipital condyle or further ventrally

Character 754: Maxillary teeth, shape: (UNORDERED)

0: mediolaterally thin and recurved

1: lanceolate (as in therizinosauroids)

2: conical (as in alvarezsauroids)

Character 755: Dorsal vertebrae, form of opisthocoely in taxa with opisthocoelous dorsals:

0: some dorsal vertebrae opisthocoelous

1: most or all dorsal vertebrae opisthocoelous

Note (via Brusatte et al., 2014): Only those taxa with strongly opisthocoelous dorsal vertebrae (character 99) are scored for an affirmative score here. All other taxa are scored as inapplicable ("?").

Character 756: Dorsal vertebrae, shape of neural spine in posterior dorsals:

0: approximately square-shaped or slightly rectangular, with a dorsoventral height that is equal to or slightly greater than the anteroposterior length at the base

1: rectangular, much higher dorsoventrally than long anteroposteriorly at the base

Character 757: Dorsal vertebrae, position of parapophysis in posterior dorsals:

0: distinctly ventral to transverse process

1: at approximately same dorsoventral level as transverse process

Character 758: Caudal vertebrae, morphology of anterior caudals:

0: amphiplatyan

1: procoelous

2: opisthocoelous

Character 759: Caudal vertebrae, position of transverse process on anterior caudals:

0: approximately centered (in the anteroposterior dimension) on the centrum

1: anteriorly displaced

Character 760: Coracoid, biceps tubercle:

0: absent or subtle

1: present as a discrete, mound-like structure

Character 761: Coracoid, strong lateral ridge on lateral surface, extending posteriorly from biceps tubercle along posteroventral process:

0: absent

1: present

Character 762: Scapula, tubercle on posterior surface of bone dorsal to glenoid:

0: absent

1: present

Character 763: Humerus, shape of internal tuberosity in anterior view: (ORDERED)

0: triangular or rounded, not discretely separated from remainder of humerus

1: rectangular, separated from the humeral head by a small but distinct notch

2: rectangular and hypertrophied, separated from the humeral head by a large notch

Note (via Brusatte et al., 2014): This character is modified into an ordered multistate with the addition of a second derived state, to distinguish between the condition in some therizinosauroids in which the internal tuberosity is rectangular and offset by a small notch (*Falcarius*, *Erliansaurus*) and that in which the internal tuberosity is rectangular but also proportionally hypertrophied and set off by a larger notch (*Segnosaurus*, *Erlikosaurus*, *Neimongosaurus*, *Suzhousaurus*, *Nothronychus*). This distinction follows Zanno et al. (2009, character ZCD 285).

Character 764: Ulna, shape of olecranon process:

0: transversely broad

1: mediolaterally thin and blade-like

Character 765: Ulna, position of distal articular surface:

0: limited to distal end

1: trochlear articular surface extends onto dorsal surface of ulna, bulbous in shape

Character 766: Metacarpal III, form of proximal articular surface:

0: flat or slightly convex

1: deeply concave and cup-like

Character 767: Metacarpal III, length:

0: considerably longer than length of metacarpal I

1: approximately same length as, or slightly longer than, metacarpal I

Character 768: Manual digits, paired flexor processes on proximal portion of ventral surfaces of proximal-most phalanges:

0: absent

1: present

Character 769: Manus, shape of proximal articular surface of ungual of first digit:

0: ovoid, dorsoventrally taller than mediolaterally wide

1: approximately square shaped, as mediolaterally wide as dorsoventrally tall

Character 770: Manus, form of lateral groove on ungual of first digit:

0: unenclosed or partially enclosed proximally by small flange

1: proximal end of grooves passes through foramena on ventral surface of ungual

Character 771: Femur, interaction of head and greater trochanter:

0: confluent

1: separated by a cleft

Character 772: Femur, shape of lateral distal condyle:

0: distally rounded

1: distally conical, projecting substantially further distally than medial condyle

Character 773: Tibia, shape of medial posterior condyle of proximal end in proximal view:

0: posteriorly rounded

1: posteriorly conical, projecting substantially further posteriorly than lateral condyle

Character 774: Fibula, orientation of proximal margin (as seen in lateral view):

0: horizontal or nearly horizontal

1: anterior portion of proximal margin extends substantially further proximally than posterior portion

Note (via Brusatte et al., 2014): This character is equivalent to Zanno et al. (2009: character ZCD 329).

Character 775: Fibula, shape of proximal surface in proximal view:

0: anterior and posterior portions of surface with nearly equal mediolateral widths

1: anterior portion markedly wider mediolaterally than posterior portion

Note (via Brusatte et al., 2014): This character is equivalent to Zanno et al. (2009: character ZCD 331).

Character 776: Astragalus, fossa on anterior surface of lateral portion of base of ascending process, sometimes bearing accessory fenestrations:

0: absent

1: present

Character 777: Astragalus, horizontal groove on proximal portion of anterior surface of condyles, separating condyles from ascending process:

0: present

1: absent

Character 778: Pes, phalanges of pedal digit IV, shape:

0: anteroposteriorly long, proximal and distal articular surfaces well separated

1: anteroposteriorly short, with proximal and distal articular surfaces very close together, particularly in distal elements

***Characters Relevant to Therizinosauroidea (from Zanno et al. 2009 and Zanno 2010):**

Character 779: Basisphenoid, inflated basisphenoidal bulla:

0: absent

1: present

Character 780: Braincase, foramen magnum, size (area): (ORDERED)

0: smaller than size of occipital condyle

1: approximately equal in size with occipital condyle

2: larger than size of occipital condyle

Character 781: Ectopterygoid, position:

0: posterior to palatine

1: lateral to palatine

Character 782: Dentary, mediolateral width of symphyseal region of conjoined dentaries:

0: narrower than width of post-symphyseal region

1: broader than width of post-symphyseal region

Character 783: Premaxillary teeth, serrations:

0: present

1: absent

Note (via Brusatte et al., 2014): It has been shown that unserrated premaxillary teeth are present in some juvenile tyrannosaurids, but serrations are gained during ontogeny (Carr and Williamson 2004). Taxa in which this ontogenetic shift occurs are scored for the present condition (state 0). Any taxa with any trace of serrations on any premaxillary tooth (whether all teeth are serrated or not) are also scored for the present condition. Only those taxa in which all known premaxillary teeth are lacking serrations are scored for the absent condition (state 1). It is likely that there is positional variation in serration presence/absence along the premaxillary tooth row, but this is difficult to parse out because complete dentitions are not known for most taxa. We recognize that future authors may wish to divide this character into additional states referring to serration presence/absence on individual premaxillary teeth or regions of the premaxillary dentition.

Character 784: Dentary teeth, shape of mesial (anterior) teeth:

0: not conical (i.e., ziphodont or lanceolate)

1: conical

Note (via Brusatte et al., 2014): Although the first one or two dentary teeth of derived tyrannosauroids such as *Alioramus* and *Tyrannosaurus* are small and somewhat conical (in comparison to the remainder of the dentary tooth row), these taxa are here scored for state 0. State 1 refers to taxa in which the first several teeth of the dentary (at least the first four, where visible) are distinctly conical compared to the more posterior (distal) dentary teeth, but are of approximately the same size as the more

distal premaxillary teeth. Oftentimes this condition is referred to in the literature as "anterior dentary teeth that are similar to the premaxillary teeth in shape".

Character 785: Dentary, extent of tooth row:

0: teeth present at anterior (mesial) tip of dentary

1: teeth absent at anterior dentary but present further posteriorly (distally)

Note (via Brusatte et al., 2014): This character encapsulates Zanno et al.'s (2009) revision of the original TWiG character number 220, which is character number 217 in the current analysis. We retain character 217 in the original language of Turner et al. (2012) here, as it refers to an ordered progression of fully toothed-posteriorly edentulous-completely edentulous, and therefore the addition of a fourth state for an anteriorly edentulous dentary (which is unique to some derived therizinosauroids) would disrupt this ordered sequence. Our preference is to include the therizinosauroid condition as a separate character, with those taxa that are completely edentulous scored as inapplicable ("?").

Character 786: Cervical vertebrae, length of anterior centra:

0: up to three times longer anteroposteriorly than the minimum transverse centrum width in ventral view (or the height of the anterior articular surface in lateral view, if the centrum cannot be viewed ventrally)

1: hyperelongate, approximately five times longer than wide

Note (via Brusatte et al., 2014): Zanno et al. (2009) presented a three-state character, but we do not want to over divide a ratio character such as this, and therefore recognize the major distinction among coelurosaurs as between a centrum that is fairly anteroposteriorly short (somewhere between 1-3 times longer than wide) and the so-called "hyperelongate" condition in which the centrum is approximately five times longer than wide. Those taxa that do not preserve anterior cervical, but have middle cervical that are hyperelongate, are scored for state 1.

Character 787: Cervical vertebrae, morphology of ventral surface of centrum:

0: smoothly flat or convex

1: with distinct depression anteriorly (at the level of the parapophyses)

Character 788: Cervical vertebrae, posterolateral margins of ventral surface of centrum, form:

0: unpronounced

1: developed into prominent crests (ventrally projecting “fins”)

Note (via Brusatte et al., 2014): Zanno (2010) notes that less pronounced crests, potentially homologous to state 1, are present on the cervical vertebrae of some ornithomimosaurs, but Zanno et al. (2009) scores all score-able ornithomimosaurs for state 0. We agree that ornithomimosaurs should be scored for state 0, as taxa such as *Gallimimus* (ZPAL MgD/I-94) clearly do not possess the prominent “fin-like” crests of therizinosauroids and some basal oviraptorosaurs.

Character 789: Dorsal vertebrae, anterior dorsals, dorsoventral depth of neural arch compared to that of centrum:

0: depth of region between prezygadiapophyseal lamina and base of neural spine less than or equal to depth of anterior and/or posterior articular surface of centrum

1: depth of region between prezygadiapophyseal lamina and base of neural spine greater than depth of anterior and/or posterior articular surface of centrum (neural arch hypaxially inflated relative to centrum)

Character 790: Dorsal vertebrae, size of parapophyses on anterior dorsals:

0: moderate in size, articular facet of parapophysis less than half dorsoventral depth of anterior articular surface of centrum

1: hypertrophied, articular facet greater than two thirds of the dorsoventral depth of the anterior articular surface of centrum

Character 791: Caudal vertebrae, pneumatic foramina on the centra of anterior caudals:

0: absent

1: present

Character 792: Scapula, dorsal flange on dorsal margin of scapular blade:

0: absent

1: present

Character 793: Humerus, crest on posteromedial surface of humeral shaft:

0: absent

1: present

Character 794: Humerus, tuberosity on anterior surface of distal humerus, proximal to entepicondyle:

0: absent

1: present

Character 795: Humerus, entepicondyle:

0: present and prominent as a spherical, conical, or crest-like structure in cranial view

1: extremely reduced or absent

Note (via Brusatte et al., 2014): This is a modified version of Zanno et al. (2009: character ZCD 291). We find it difficult to distinguish between Zanno et al.'s discrete conditions of "spherical" and "crest-like," which are given separate states in the original analysis. Therefore, we combine these conditions into a single "present and prominent" state, and recognize the major difference between coelurosaurs as the presence or absence of a prominent entepicondyle. Taxa with a prominent entepicondyle usually, if not always, exhibit a prominent groove proximal to the entepicondyle, whereas those that have a reduced or absent condyle do not. Therefore, we do not utilize Zanno et al.'s (2009: character oZCD 292) separate character for the groove.

Character 796: Humerus, mediolateral width of distal end:

0: greater than 2x minimum width of shaft

1: less than 2x minimum width of shaft

Note (via Brusatte et al., 2014): This is a modified version of Zanno et al. (2009: character oZCD 293), which combines two of the original states ("moderately expanded" and "significantly expanded") into a single state of "greater than 2x minimum width of shaft. Therefore, the character as worded here distinguishes the aberrantly unexpanded distal humeri of ornithomimosaurs and some tyrannosauroids from the standard condition among theropods.

Character 797: Humerus, morphology of distal humerus in anterior view:

0: medial aspect of distal humerus unexpanded, entepicondyle situated proximal to ulnar condyle

1: medial aspect of distal humerus expanded and subtriangular in anterior view, entepicondyle located well medial to ulnar condyle

Character 798: Metacarpal I, rectangular buttress on ventrolateral aspect of proximal surface that underlies ventromedial surface of metacarpal II:

0: absent

1: present

Character 799: Manus, ratio of the proximodistal length of metacarpal II to that of the combined proximodistal lengths of phalanges II-1 and II-2:

0: less than or equal to 1.0

1: greater than 1.0 (i.e., metacarpal II longer than combined lengths of phalanges II-1 and II-2)

Character 800: Manus, ligament pits on manual phalanges:

0: strongly developed

1: weakly developed or absent

Character 801: Manus, manual unguals, proximodistal length:

0: shorter to, equal to, or slightly longer than length of penultimate phalanx

1: elongate, twice or more as long as penultimate phalanx

Character 802: Ilium, orientation of ventral portion of preacetabular process:

0: parasagittal, in line with dorsal portion of process

1: laterally deflected, extends nearly perpendicular from sagittal plane of ilium

Character 803: Ilium, size of preacetabular process:

0: moderately developed, anteroposterior length of process subequal with dorsoventral height of ilium directly dorsal to the center of the acetabulum

1: hyperelongate, length of process at least twice the height of ilium above acetabulum

Character 804: Ilium, orientation of blade:

0: parallel or gently inclined relative to dorsoventral plane passing through the pubic and ischial peduncles (the ventral portion of the ilium)

1: angled laterally relative to the ventral portion of the ilium, rising steeply at at least a 30 degree angle from the dorsoventral plane passing through the peduncles

Character 805: Ilium, morphology of dorsal surface of postacetabular process in dorsal view: (ORDERED)

0: smooth and non-rugose

1: with rugosity causing transverse expansion of the posterior dorsal margin

2: hyperrugose, with hypertrophied posterior tuberosity

Character 806: Ilium, orientation of pubic peduncle: (ORDERED)

0: straight

1: posteriorly recurved, articular face posteroventrally directed

2: posteriorly recurved, articular face posteriorly directed

Character 807: Ilium, morphology of antitrochanter: (ORDERED)

0: separated from ischial peduncle

1: merged with ischial peduncle, both structures together form enlarged ventrolaterally flattened boss

2: merged with ischial peduncle as a boss, which is hypertrophied and spherical

Character 808: Ischium, position of distally placed dorsal process along posterior edge of shaft: (UNORDERED)

0: entirely proximal to obturator process

1: opposite obturator process

2: extending distal to obturator process

Note (via Brusatte et al., 2014): This character refers to the position of the dorsal process of the ischium, whose presence or absence is scored in character 226. Those taxa without a process are scored as inapplicable ("?").

Character 809: Ischium, shape of obturator process in those taxa in which the process contacts the pubis:

0: approximately square-shaped

1: elongate anteroposteriorly, approximately twice as long as dorsoventrally deep

Note (via Brusatte et al., 2014): The presence/absence of an obturator process contacting the pubis is scored in character 164. Those taxa scored for the absence of this feature in character 164 are scored as inapplicable ("?") for the current character.

Character 810: Ischium and pubis, morphology of contact surfaces between the two bones:

0: flat

1: markedly sinuous

Character 811: Pubis, shape of shaft:

0: rod-like

1: mediolaterally flattened

Character 812: Pubis, morphology of distal shaft:

0: distal portion of shaft approximately equal (or less than) in anteroposterior length to proximal portion of shaft

1: distal portion of shaft greatly enlarged, more than twice the anteroposterior length of the proximal portion of shaft

Character 813: Femur, anteroposterior width of region bridging femoral head and greater trochanter in proximal view:

0: smoothly confluent with head and greater trochanter

1: anteroposteriorly constricted relative to head and greater trochanter

Character 814: Femur, hook-like process at distal edge of femoral head, demarcating a notch between the head and the shaft

0: present

1: absent, shaft and head smoothly confluent

Note (via Brusatte et al., 2014): This is equivalent to Zanno et al. (2009: character oZCD 323), which refers to the presence or absence of a "raised ventral rim" that separates the femoral head from the femoral neck (essentially the shaft). The "ventral rim" is equivalent to the hook-like process of the femoral head, which extends further distally relative to the remainder of the head to demarcate a notch between the head and shaft in anterior and posterior views.

Character 815: Tibia, morphology of incisura tibialis (lateral fossa on the tibia in proximal view):

0: deeply inset

1: wide and shallow, nearly absent

Character 816: Tibia, shape of tibia in proximal view:

0: anteroposteriorly longer than mediolaterally wide (measurements taken through midpoint of proximal surface)

1: mediolaterally wider than anteroposteriorly long

Character 817: Tibia, length of fibular crest:

0: short and proximally positioned, extends up to approximately 1/3 of the length of the tibia

1: long and distally extensive, extends to approximately the midshaft of the tibia

Character 818: Tibia, exposure and morphology of the anteromedial region of the distal tibia: (ORDERED)

0: covered anteriorly by the astragalus

1: not covered anteriorly by the astragalus, exposed

2: exposed and developed into an anterior tuberosity

Character 819: Fibula, iliofibularis tubercle, position:

0: proximal to midshaft of fibula

1: approximately at midshaft of fibula

Note (via Brusatte et al., 2014): In those alvarezsauroids with an apomorphically shortened fibula (e.g., *Shuvuuia*, *Xixianykus*, *Parvicursor*), the position of the tubercle is measured relative to the length of the shaft of the tibiotarsus. Therefore, these taxa are scored for state 0 although their tubercles are located near the distal end of the shortened fibula.

Character 820: Astragalus, ascending process position:

0: lateral edge terminates approximately at the lateral edge of the tibia, or medial to the lateral edge of the tibia

1: lateral edge extends lateral to the tibial shaft to contact and overlap the fibula

Character 821: Astragalus, lateral condyle of distal end, morphology:

0: well developed

1: strongly reduced, lateral tibia exposed on distal and anterior surfaces of tibiotarsal region when tibia and astragalus are in articulation

Character 822: Metatarsus, overall shape:

0: elongate, unit comprised of metatarsals II-IV much longer proximodistally than wide mediolaterally

1: short and broad, unit comprised of metatarsals II-IV less than twice as long as wide

Character 823: Metatarsus, proximodistal length: (ORDERED)

0: greater than 45% length of tibia

1: between 44-38% length of tibia

2: less than 36% length of tibia

Note (via Brusatte et al., 2014): For those taxa in which only isolated metatarsals are present (i.e., not a complete metatarsus), the length of metatarsals II, III, or IV is taken as a proxy for the length of the metatarsus as a whole.

Character 824: Metatarsus, orientation of individual metatarsals II-IV:

0: not closely appressed, divergent from each other

1: appressed throughout most of metatarsus

Note (via Brusatte et al., 2014): The outgroups *Allosaurus* and *Sinraptor* are scored for state 0, as in these taxa metatarsals II and III are closely appressed but metatarsal IV is divergent. *Zuolong* has closely appressed metatarsals II and III, but because only a fragment of metatarsal IV is known it cannot be determined if IV was appressed or divergent, and therefore this taxon is scored as "?". Within coelurosaurs, all therizinosauroids are scored for a divergent metatarsus, following Zanno et al. (2009: character ZCD 335). Taxa without a complete metatarsus can be scored based on the shape of metatarsal IV (a laterally divergent kink along the shaft is indicative of state 0, whereas a straight shaft is indicative of state 1).

Character 825: Pes, pedal unguals of digits III-IV, proximodistal length:

0: approximately the same length as the penultimate phalanx (ungual may be slightly shorter or longer than the penultimate phalanx)

1: twice as long or more than the penultimate phalanx

***New Characters Relevant to Basal Coelurosaurs Established in Brusatte's Dissertation (Note that some are inspired by published literature, as described in the main text, and others may have been added to published analyses during the time Brusatte's dissertation was written).**

Character 826: Premaxilla, anteroposterior length compared to that of maxilla:

0: length of ventral (alveolar) margin greater than 10% total anteroposterior length of maxilla

1: length of ventral (alveolar) margin less than 10% total anteroposterior length of maxilla

Character 827: Supraoccipital, tab-like processes on left and right sides of dorsal margin of bone:

0: absent

1: present

Note (via Brusatte et al., 2014): This character has been included in previous phylogenetic analyses of tyrannosauroid interrelationships (e.g., Carr and Williamson 2010), but Brusatte et al. (2010a) combined this and the absence/presense of a dorsal bifurcation of the supraoccipital into a single character, due to a presumed equivalency between the characters. As outlined by Bever et al. (2013), however, the tabs are separate from the bifurcation, as many tyrannosauroids have tabs but only a few taxa exhibit a bifurcation. The tabs themselves are created by midline depression of supraoccipital relative to left and right sides, as explained by Bever et al. (2013).

Character 828: Nasal, premaxillary processes (=supranarial processes), extent of their apposition to each other on the midline in dorsal view:

0: apposed for nearly their entire length (may abruptly separate from each other at their tips)

1: not apposed for most of their length, and therefore do not abruptly separate from each other at their tips

Character 829: Dentary, Meckelian groove, position:

0: approximately centered at dorsoventral midheight of dentary

1: positioned ventrally, located closer to the ventral margin than the dorsal margin

Note (via Brusatte et al., 2014): This character was originally used by Carr and Williamson (2010: character 211), in which state 0 was scored for all score-able tyrannosauroids. We here note that this condition is also present in outgroups, *Compsognathus*, and derived therizinosauroids (*Erlikosaurus*), whereas state 1 is present in *Ornitholestes*, ornithomimosaurs (*Harpymimus*), basal therizinosauroids (*Falcarius*, *Beipiaosaurus*, *Alxasaurus*), and paravians (e.g.., *Deinonychus*, *Zanabazar*).

Character 830: Dentary, row of foramina on lateral surface paralleling ventral margin:

0: absent or limited to a small series of foramina at the anterior end of the dentary (at the level of the first 1-4 alveoli)

1: present as a distinct row that extends along most of the lateral surface of the dentary (across the entire length of the tooth row at the very least)

Character 831: Splenial, notch along dorsal margin of anterior process, where the splenial contacts the supradentary (if present):

0: absent

1: present

Character 832: Cervical vertebrae, position of prezygapophyses in anterior-middle cervicals:

0: prezygapophyseal facet approximately level with anterior face of centrum

1: prezygapophyseal facet entirely anterior to anterior face of centrum

Character 833: Caudal vertebrae, morphology of dorsal portion of neural spines of anterior caudals:

0: unexpanded

1: expanded anteroposteriorly relative to the remainder of the neural spine (and often mediolaterally as well)

Character 834: Ulna, morphology of distal end:

0: expanded mediolaterally at least 1.5x relative to midshaft mediolateral width

1: expanded less than 1.5x midshaft mediolateral width

Note (via Brusatte et al., 2014): The denoted measurements are taken in anterior (extensor) and posteror (flexor) views.

Character 835: Ilium, medial surface of preacetabular process, pronounced horizontal shelf continuing from anterior margin of pubic peduncle to demarcate the cuppedicus fossa dorsally:

0: absent

1: present

Character 836: Metatarsal III, mediolateral width of distal end compared to mediolateral widths of distal ends of metatarsals II or IV (whichever of the latter is greater):

0: less than 1.3 times as wide

1: greater than 1.3 times as wide

Character 837: Lacrimal, extent of antorbital fossa on region where anterior and ventral rami meet:

0: extensive, fossa excavates nearly entire region and nearly extends to the posterodorsal corner of the lacrimal (the lacrimal angle), leaving only a thin region of bone at the posterodorsal corner of the lacrimal

1: reduced, fossa stops well short of the posterodorsal corner of the lacrimal

Character 838: Scapula and coracoid, deep fossa on lateral surfaces of both bones in the region of their suture (covering the posterior half of the coracoid and anterior portion of the acromion plate region of the scapula):

0: present

1: absent

Character 839: Scapula and coracoid, contribution of each bone to the glenoid: (UNORDERED)

0: both bones contribute approximately equally in the anteroposterior dimension

1: scapula contribution markedly anteroposteriorly longer than coracoid contribution

2: coracoid contribution markedly anteroposteriorly longer than scapula contribution

Character 840: Femur, shape of anterior margin in proximal view:

0: strongly convex, due to a midline tubercle

1: essentially straight or slightly concave

Character 841: Femur, horizontal ridge on the anterior surface of the head and neck, demarcating a deep fossa ventrally:

0: absent

1: present

Character 842: Femur, extent of crista tibiofibularis in distal view: (ORDERED)

0: projects further posteriorly than medial condyle

1: extends to the same approximate posterior level as medial condyle

2: terminates well short of the posterior level of the medial condyle (medial condyle projects substantially further posteriorly)

Character 843: Tibia, position of medial ridge on posterior surface of distal end: (ORDERED)

0: displaced laterally, positioned lateral to the medial edge of the distal tibia by approximately 25-33% of the mediolateral width of the distal tibia

1: positioned lateral to the medial edge of the distal tibia approximately 10-20% of the mediolateral width of the distal tibia

2: positioned medially, positioned at approximately the posteromedial corner of the distal tibia in distal view

Character 844: Scapula, fossa on lateral surface of bone immediately above glenoid, which is demarcated dorsally by a convex bulge or ridge spanning the scapula-coracoid suture:

0: absent

1: present

Character 845: Humerus, deltopectoral crest, orientation relative to mediolateral long axis of proximal end of humerus in proximal view:

0: straight, approximately perpendicular to long axis of proximal end of humerus

1: curves strongly medially as it continues anteriorly, such that anterior end of crest is oblique to long axis of proximal end of humerus

Note (via Brusatte et al., 2014): Those taxa scored for state 0 for character 358 (a deltopectoral crest that is projected laterally or dorsally, such that it is in line with the long axis of proximal end of the humerus) are here scored as inapplicable ("?").

Character 846: Femur, position of fourth trochanter: (UNORDERED)

0: along posteromedial corner of shaft along its entire length

1: positioned near center of posterior surface of shaft distally and extending proximomedially to become confluent with posteromedial corner of shaft proximally

2: positioned near center of posterior surface of shaft distally and extending proximolaterally to bcome confluent with the greater trochanter

Character 847: Fibula, anteroposterior width of the minimum point of the midshaft compared to the maximum anteroposterior width of the proximal end:

0: 20% or greater

1: less than 18%, fibula shaft exceptionally gracile

Character 848: Maxillary teeth, posterior (distal) extent of tooth row:

0: extensive, extends posterior to the level of the maxillary ascending ramus and underneath the antorbital fenestra (if present)

1: limited, terminates posteriorly at the level of the maxillary ascending ramus

Character 849: Dentary, shape of ventral margin in lateral view:

0: approximately straight or slightly convex

1: broadly concave

Character 850: Maxilla, posterior extent of the ascending ramus relative to that of the main body (jugal ramus):

0: the two rami extend to approximately the same level posteriorly, distance between posterior tip of ascending ramus and posterior tip of main body no more than 1/3 of the anteroposterior length of the entire maxilla

1: main body extends considerably further posteriorly relative to the ascending ramus, distance between the posterior tips of the two rami is greater than 1/3 of the length of the maxilla

Character 851: Tibia, proximal surface, proximal extent of cnemial crest relative to the proximal extent of the posterior condyles:

0: cnemial crest extends further proximally than condyles

1: cnemial crest and condyles extend to same approximate level proximally

Character 852: Prootic, prominent fossa on the lateral surface of the bone, anterior to the otic recess and posterior to the preotic pendant, that houses the external foramina of the trigeminal and facial nerves and pneumatic openings:

0: absent

1: present

Character 853: Palatine, form of pneumaticity in those taxa with pneumatic palatines:

0: pneumatic fossa on the external surface of the bone

1: large internal chamber that opens externally via a window-like pneumatic opening

<u>CHARACTERS ADDED TO THE TWiG DATASET BY CAU ET AL. (2015):</u>

Character 854: Metacarpal I, proximal half:

0: mediolaterally expanded, width comparable to rest of bone (0);

1: narrower than distal half, medial margin sloping proximolaterally (1)

Character 855: Distally closed intermetacarpal space between metacarpals II and III:

0: absent, metacarpals not contacting distally;

1: present

Note (via Cau et al., 2015): This character is not redundant with character 391, which describes the extent of the intermetacarpal space among the taxa bearing the closed intermetacarpal space and is scored as 'inapplicable' among the other taxa.

Character 856: Metacarpal III, distal end:

(0: bicondylar;

(1: simple convexity.

Character 857: Dorsal margin of manual unguals:

(0: does not;

1: does arch dorsally above level of articular facet (Senter 2007; Agnolín and Novas 2013).

Character 858: Interpubic space, width between conjoined pubes:

0: gradually narrowing distally;

1: wide pubic canal and laterally bowed pubis, followed by an abrupt narrowing at the symphysis.

Character 859: Length of pedal phalanx I-1:

0: < 66% III-1;

1: > 66% III-1.

Note (via Cau et al., 2015): The derived state is present exclusively in a subset of avialans among Coelurosauria. *Balaur* shows the apomorphic state.

Character 860: Metatarsal II, distal condyles, plantar projection:

0: medial and lateral condyles with comparable projection;

1: medial condyle much further projected ventrally than lateral. (O'Connor et al. 2014)

Note (via Cau et al., 2015): This character describes the marked medial projection of the distal condyles of metatarsal II present in *Balaur* and some avialans.

XV. Character-taxon matrix for second data set. Character-taxon matrix including 860 total characters and 157 taxa, based on the Theropod Working Group dataset, mainly off the working dataset of Brusatte et al. (2014) and Cau et al. (2015, 2017).

Allosaurus_fragilis
?10000?0000000100011001000101200111011001 0??00000000000010000000000100010000000000101
01001000000010010100000000000100000000????000000000000000?100000000000100001001010000
001000100000110000001000000000000000000001000000000000?0?000?001100001000000100001110002
1011000000000020000000000?0000??0000000?00000??0000000010000[01]0?0????????0000020?00?0000
000101000?0?0010000?000000000000001000010?01000??0000000?000?000002000000000000?00000?00
?00100000000020000?000100??001100000[01]0?00000000100000000110000000000100011000000002112
100100100010 0?1????000000000100??10?0000?000000000000011000?100?000?0?1000000000????000?0
0010010000101000000000000000001020000000000000000000001000000000000020101[01]0000100000000000
0000000100000010?0001000000102011000000000110000000010000000000000010010001000000000000
0000000000000000000000000000000000??0000000000000000000000?000001000000000000000?00000
00

Sinraptor_dongi
?10000?000?00010001000000010120000101100 10?00?00000000000010000?00000100?10?0000000001010
1001000000010010100000000000001??????0??1?10?0?0??00??0??????000000000?010??010010100000001?
0?1000001100000010000000000000?000?000?00?000000?0?000?001100001001000100000100002101??
?00000000000000000?0000??0000000?00000??000000010000??00?00??00?????2????????????1010???
??????????????????????????????1??????0??000??00000000200000000000000000?00?00??0???00020
000?0001?0??0011000?????????0001000000000000000000001000001100000002102100000?010?00?1000
0000000000100??01?0000?000000000000?11000?100?010?0?100?00?001000?0010000100100001010?0?
000000000000112000000?00????000000010000?00000020101??0??1000000???????????000010010?0000?
0000102011010 10?0001100000 0??1?00000000000001001000??0???000000000100?000000000000000???
????00000000??0000000000000000000000000?0010?00000?00000001????0?0

Dromaeosaurus_albertensis
?0??00100000000000010??0???0?01??01110????1111????101?1001100??00000010011111000?000010100
1??1??????????????????????????????
????????????11?1?0????00??????00?0?00??1?????????1?00??011000100?0??????????????0???0000?000
00??0000??0000000?00000??00???
??????????????00??????????????????????????????????????0????0?????????010121?000??0???00?0??????
???0????0??0??0???0??????0?0100?00?00????????????????????????0????00000100000???????0???????0
001001?0?0????00??00?????0?0?10????????????000?1000000010????000000000001120100000000?0?0001
0??0??000?0?????????????
0??0?010????????????????????????0200000?????????????????????????????????????0?1?0??110??????????
??????000?0????????

Deinonychus_antirrhopus
?0010????1???????1??0000?011100011100011111100?1??00001110100000?0100111?1000?010010100
0?110001100?1121011????0011011112 1??????1?110111001001110000010022111010102 0220?0111121
1201?11110000000000100011010010000000001100000?00010?00100021100000111101 10000??0000012
2000000000?0????000????0???????????00000??000000000000?[01]0?0?????????????20000?000?000111
000?0?0010000?00000000000000101?0???00000??0100000?000?0000000000000000000000000000?00100

000000????????0?1????00111?????????0000?????00001?10?0010001?000001010??00?0???????00??0?0?
?0?????00001000000??01?00?0?00000001??????????????????????0?0?1001000?000?00????????????????
?000000000001120100?0000?0?????00000000?00001000100?????00000?11010100001 00??1?0000???????
?????????00000?000000000?00?100?00??0001?1??0?000010?100000000010?010??00?0000000000000
0000000000??0????000000000100??1101?000110???200?10001?00001?00

Velociraptor_mongoliensis
?00100100100001201120000101110001110001211 1?10?0101000011101000000010011111000101 00101
0000110001100111210111110001101111211110101111111100100111000001002211101110202202 0111
12212011111?000000000111011010010000000001100000000011?0010102110000011100110000000000
0112000000000000?000000?0000??0000000??0000??0000?00000000[01]0?00??000?00000020000?000?
000111000?0?001000???0000000000000010100???00000??0100000?000000000000000000000000000000
?0110000000010[12]21?0001101100011100000?100000000?0100001?10?0010001?0?00010101000000??
?0??0?01??00?0????000001000000??01?0000?0000000 1001?0?0???000??00110100?0???01000?000?000
001001000?00000000000??00?112010000??????00000?????????????01001000?000001110101?0?0?00
?010000011 0?01?010000000000000000000000?000100000?000?101010?000010010000000010?01002
000000000000000000000000000??00001000000000010000?1?10?0011010020001000100000100?

Balaur_bondoc
??
???1?2??1????0???10?????????0????111[01]11101001?1?00001???2???0111?202?02?0?1?22122????????
????????21100010010 00??00??1???????????0?00??0??????????????????????1?????2?00????????????????
??????????????????????????00000000?[01]???0????????????20??????0???0111000?0?001000???010?00
?????010?00???21??0?00000020?100??0000??????00???0?000000000?00??0?00000????????010????????
1????????00000??
??
???????????0100?0?0??0?00?1101010000?00?01000?0110?01?????????????000?00??00?????00100000?0
0??1??????0000100100?0000?????0?0???????????00000000000000000??000????000000210????????00
0?10???200?1??????1110111

Tsaagan_mangas
?00100100100001201120000101010001?1000121111100010110000100??000000100?11110001010 0101
00001100??1??????0??????????????????1????????11?111???
??????????????????????????00??00??00?0000???????????211000011?00?0000000?00001????0000000??
??000000?0000??0000000?00000???000??????????????????????????20000???0?0?0??10???????????????
??0????????0121?00???0??0001
1?0????????????00?0100001?10?0010001?0?0001010100 0?00???0??0?01??00?0????000001000000??0??
0000?0000000 1001?000???000??001101?0?0???????????????000100100000000000000??00?112010000
000????000000???00?00001??????????????00?110??000
?000?????????0?10?010?????10??????????????????02?0000000??????????????????????????????????????00
0?1?1????110????0???000?0????????

Bambiraptor_feinbergi
?0010??001000012010[02]00?010111000?110001211 1?1000101?00011?0?0?000?0100?1111000?010010
100??1110?1100?1??100?0?1100[01]1011??2????0101011011110?001?100000111?211?0101?202202?11
1022100???1110000000000 10001101?000000000000110000000011?0010?021100??11?110??0000?00000
0122000?00000?0??00000??0000??0000000?1?000??000000000100??0??0?0?0?0?000002 0000?00000?0
11?000?0?0?1000??000000000?00010??????00000??0000000?000?0000000000000000000000000?00?011
?100000000121?000110??00011101100?100000000 10100001?10?0110001?0000010001000000???0??0??
1??00?0????000001000000??01?0000?00000001??1?0?????000??001101???????????????????0??????????
??????0?000??00?1120100?????????????0000000?00001000101100 0?0000011101010 00010?0010000011
0?01?010000000?00000?000000000?0001?000??00001?10?0?100010?100????????????????0?000?????00
??00????000000??00001000000000100?0???10000110100200?10001????01?0?

Tianyuraptor_ostromi
??01?????????????????0????111?????????????0??1??????????????????????????????0?0?0?1010??0???0?
????????0?0?0?1??01?0112???????1??00?011110?001?1100001112?1??0?1102223?21111022?0???1?110
?????????00?11????000??0???1??00?0??01???1011?0???0??????1?0??????????0????2?000?0?????????
???????????????????????????0??????????0?00?00????00000?0?00?000?0?0????????????????????????
???????????????????????00?1?0????10??????????????0?0????????20?101????????????1???????????0000
?10??000??1???????????????????????????0??
??0???????????????????????????0?????????
?????????????????01?1?1???00??0?0????000??????????????0?????0?0???00????0????0?0???0???????0
???0?????0??0??????????????????0?????0???0????????????????????????0010????????0?0???????????0?
0??????????

Sinornithosaurus_millenii
0001??????0??????????00???1110????1000111100?1????1??0????????00?00100??1????010100??100????
????1??1???????0???00?1??1???????01?1?11011110?0?????0000?00?201?01?112023?2?111022?2?1???1
?????????110001?1100?0?00010001??0000???01101111002000000111100?00?00???000???20000?000??
???0??000????0????00000??1000??00?00?0?????0?0??0???????00?0?[12]0000???0?000100?00?0??010?0
??00?????????????????????0000??0000?0000??0?0?00?????000??0?0000?00?0???0111?11000???????01?
?10000111?1100?1000000?010100000000?0000000?0?00000001000200???0??0??1??00?0????00000000
0000??00?0000?00000001??1????0??000??001101?????????0??????????????????????????001000?010112
010000???????100?0???????????????????????????0011101????00?000?????????0?01????????????????0?0?
??00000??0??00000?000?1??0?0?????10?????0?00?????0?0???0100??????0????00000????001?000?????0
??000100?011?????0110????0???000????????0?

Microraptor_zhaoianus
0??????????????????100???0??010?0?1????0?01000000?????00
1?1??01?2100?01???0110?1212111?01?1011111111010??1110000110121111??0112023?2?1110221201?11
111??00?0001110111110?100??000?11?000????1010111100?0?0???1?110???0?00??0?00???20??0?000??
?????????????????????????0?0???????00001000?[01]0?00?0000?001000200???0?000?01???10?0???1010
0?000??1?0?000?????????000???????0?00?0000000000????000?00??000000000??1211111??00?021???0
11011???1?1?1100?1000000??????????????????????????????0??
??00???1????????????
?????????00?????????????00001????????01???1?10000?000???0000??0?01??10?????0?00000?0???00???
?0????0?00??0????1????0000???10??0?00???0?010???????0???0?00??000??0?000000?000?10?00000001
0??????????0?1??0?????1???1??00?100?

Graciliraptor_lujiatunensis
???020???1??????????
????????????????0???11?12?????????????????01011?1100001???????????????????????????????????????0??
0?010?[01]11?1??0??0??00?????????????0???????????????????00???????????????200?????????????????
??0??????10?0???000??0????????
???10?0000??00?000???10?????????????????????????????????0
00??
??0????????????????
????????????????1????00?00?????????????????????????????0000????00??????100??0??0???????0????????
?00?0?00?????01???????????????????0???00????????????????0?000?10????????0?0?????????????????????
???

Hesperonychus_elizabethae
??
???21??010??????????????221211??????????????
????????1?????????????1??????????0??1??
??

???0???????????????????????????????
???
???
??????????????????01000?0110?01?????????????????????????????????0???0?????1????????????????????
????????????????????????????????000???0??

Pyroraptor_olympius
???
????????0????????1????????????????????????001???
???01??1?????????0?????????????0??????0???
??010??0????
??0??
???
???0?
0??????????0???0???????????????0?????????????????0????????
??

Rahonavis_ostromi
??0??????????????
?011121???1?01?011112?12???????????0??11??011??????0?01111?10111120?3?2012101?121??2111000
1000?011101101?0100????0??1?????0??1000101111???
?????????????????????????????0011000[01]0???????????????2????????????1100??????????????????????
???010100???????????????000000?000000000100000000?00000000?001??01???0????????0?1?11?????1?
??????00???
???
????????100?1??0?0??0011??????0?????0?100000????01??10???0?0????00?0???00???????????00??0?00?
1????0000??0?0???????0???010???????????000????????0000000?000?10??00?0001????????00?0?11?0?
?0??????????????00

Buitreraptor_gonzalozorum
?0010???????????????00001011?00??????????100?10??01?0?????????00??001?????????0210??100?001
0111100111?110???100011012[012]121??????01101111010?1?1??0??????11?101??12[02]23??01?1?1?
120??11?0??0??1?011?0?1110000?????0??1????????1101000110011000???0??????0000??0?00?10200?
00?0???????????0???????0?000101???0?????00000000000[01]0??????????01000100?0?0?00??0?10000?0
?001100??00000100000001?100???????????????000???00???0?00??????????0?00?000?0010?00???????
???????1????0010??1100?0000???00?0?????????1?11?00?0???010?0101?0??0????00000??1100????????00
00???0?0??0??0??????????????100?????000??00?0????????????????????0????????????????0?000???0?1
?2????????????????000???????0001001000??0??00000111010100???0?001??000???????01000??00??0?
????????0?0?000??????????00????0?1000100?0???????01000?????0?0001000000000????000000??0??0
1000?0??001???????10????11100?00010001???0?0???

Neuquenraptor+Unenlagia
???
???11211111111???1????1????????????0??1001???????????01111?1011112022020111?121202011?1000
0??0?010??111100000????0??11????0??110110?110??????????????????????0?????220?????????????????
???????????????????????????0?00000?0?????????????????1?????????????1100?0?0?0011000?00000??????
??????0???????????????00000?000000000000000000?00000000?00?????????????????????????????????
???
???
?????11011?0?????0011???01???????0010000110?01?010000000?00?0100??00??????????000?00?00?1
????10????01???????00???010??????????????00???0????000000??000010??0?000010?????????0??111001
000?????????????0?

Austroraptor
?0000????????????????????2?1?0????????12?0001????????????????????0?011???????????210??10?????00
11?001????11????????????????????????????????00????????0???????????????????????????????????1?????
?0?0?0?0??[12]1??????????0????0????????????????11?00??01??00?????????????1[12]20??0?0?????????
???????????????????????????0??00?00????????????????????????????????????0?0?001100???00??0000
000???
?????00?0??????????0?10100?0000010??1???????1?????????????????????????????0??01?01?????????
??????????0?10?00????????????00000?000?00?????????????????0??0010000??????????????????000????
???0001001101??????????????101????????????????????????????????000???????00?00????????????0????
?0?0???????????????????????????0?0001000?????0???????0000???????00??????0?????01?1????1?????1?
0??0001?????????

Shanag_ashile
???????????????????1??1010110????????????????????????????????????00??011??????????0100101l???????
???
???????????????????0????0????????????????2100001???00??????????????????00??0????????????????
???
???
???????1?10???00?0?000000???
??0??00??00????????????????????10????????????????????
???0?0???????????????????0?????????????
????????????????00??1?????????????????00???????????

Halszkaraptor_escuilliei
?0010?????0????????000?0??110?11??002?12100101??0?1??10???????00??0100?[01]0??0?000210??1?0
0001?0??0?001??000?[12]110?01?010?1?1???0???????????0??0?????000??0?2?11???1?????????????????
?0???100?0???0?????1101300010???000??00?0?????????????0?0?0?00001?00?0???00?00?[01]?1020020?
00?0??????????0??00???????0?????????0?000000?000?0???????????????????????????????????0?????????0
??????????????????????00???????00??????0?0?1?????1??????????0??00?00?010??001000???????01???
???1?0?????????00?00100010?000????10????0?0?0100?????0????????????0??0???0???????0000?0??????
??00?????????00??1?0????0000??00?0?0???????????????????0?0??????????0???00000??0001???????0??
?????0??000????????00???????1????100??????1???1?????00?????0???????0???????0?????0?0???000?0?1
???00?01??00????110?0??0?????000?0????????0?2?010010?000?0???????01?0?????????????????001000
?00?00010??????????1000???00?0?0?

Mahakala_omnogovae
???????????????101???????????????????????1?00???????0?0011??????????????????????02????1???00??0?
11?011?211??1?10001101221O????????????????00??1110000?100?211101?2??????????????????0111100
????0?011001101?00000??00??11?0?????0000?????00????????0????????????0????1[01]2?0??????0?????
????????????0??1?00???????????00000100000[01]0?????????????????????????????10??[01]0???0?0000???
0?????????????100????0??0???000000??0??001??0??1000000000?00000000?001??00?000????1???0????
??011?0????????000000??????????????????0???0??
?????????????????0?????000?00????????0????????????????00010?????????????????????0???????????????
?????0000?????0?01?000???????000??????????00????00100000???????0100000?0?000?100?0?000?0????
0?0?0???0????1?10???00???????????0????010?20????0?0??0???????000000000??0??0100000000010?0??
??1?000???10????01????0??00???0

Atrociraptor_marshalli
???????????????????[02]00?0??1010????????????????????????????????00??010????????0?0000101001??
???
??????????????????????0????0????????????????21?00??0??111?????????1???????????0??????????????
???????????????0????0??
???

???00001?1????1???0?0?000?01???
???0?????0?00???????????????????0?????????????????
???0?0?????????????0??????0??????????
???????????????????000??0??1??????????????????00???????????

Utahraptor
???????????????????[02]00?0??????????0?001?????????????????????????????????????0????0101??1???
?0?1100?1??1????????0??011?????????????101?1???????????0?????????0?01????????????11?????0111?0
0??0?001000100???10????????11??0???????0?????0???????????1???0???????0???10???0???0???????????
?????????????????????????????00000???????????????????20000?000000???????????????????????????????
?????????????????????00???????0??0000000000000?0000?????0?10?????????????????????????????????
????00?????000001??????????????????????????1???0??00010?0??????????????????????????????????????
???
??01?????????0?0?????????????0?????0?????
???????????00????????????0????????????????????????????????0?0??????????????????1????????????????
???????

Adasaurus_mongoliensis
?0010?????0????2??????????????0?????0?1??11?1?0?101??001??0?????0????0?1???0??????????????0110
0?11?0111?10?111[01]100??011?1?1??????1?111111??????????????10221110101 02?2202?1?11221001?
111000000?000?11010010010??00??00110?0?0?0001??00100?????????11????????00?0????1??????????0
?????????00??00???000?0???????????000?0010000[01]0???????????????200?0???00??01110???????????
?????????????????????????????????00?000000000000000000000000?002??11?00???00???21?000?
?0??0001?1?????????0???0??0?????????????????????????????????0?????????1??00??????000010??000?
?0??00?0?0?00????????0??????????0?????????????????????????0???????????????????0???????11201??????
??100000??0?01???????????????0?0???00?0????
????000?00????10???????????????????????????00??????0??00???????????000000??000010000??00010????
??????01?????????????????????0?

Achillobator_giganticus
?????????????????????????01?1??0000101??????0?0
1100?11210???????0??011?11??????????101????????????00??10220??11010102102?011011?001?21110?
0????00?000?101????0???????1???0????001??00100021100??0??100????????0???????????????????????
??????????????????????????????0000????????????????????20000?0?0?0?0??10????????????????????????
??????????????????????00???0?00000??000???????0?0000????????0???????????????0??????????0??????
?????00????????????00?001???0?0?000??1??
??10?????????
?????0100???????????????????????????100000??0101?0100000?1000??????????0?????????0??01?????1??
0??0????????????????01??0??????????0??00?????????0000000???0000100????????1????????????????1?1?????
0?00?????????

Saurornitholestes_langstoni
???111?????????????11????0?????????????????100101?00?1100
0110011121011011100?1011?1?1?????????????111?????????000111221?1?1?1?2?0?????????????????11?
?0????00??001101??00???00?0?1??00?0000010?001000????????1110011??????0????1220????????0?????
???????????????0000????????????0???????????????????????????????????0???00??1???????????????????????
????????????????????????0????????????????????????????????????0???00??????????0??????00111??????
??00?00??????????????00?01??0?0?0?000?00??
??????????000??00?1???0?000???????????????????????????????0??0000000???????????????????0??????
?????????1?0???0??????0????????????
???0?????????????????1??01?????????????0?????????00???????????????????????????????11???????????????????0
???????????

Dineobellator_notohesperis
?0?????????00?1??????????????0?0????001?????????????0?????????????????????????01?0101?????????
???????????????????101???????????????????01001????00?1?????????????????????????????????1?0????
??????0110??0??????00???????????????????00??????????10????00?????0????20????????????0?0???????
??????????????????????????????[01]1???????????????????????????????00?0?001000??00???????0??01
??0?0000??????????000??????????1?11??????????????0?
?00?????????1?????????0????0?????01????00???0??00?1???0???????0???????0????????????????????????
???????????????????????????????????0???????????0???????????????????????????????????0???????????
??????????000??????1?1?0?????????????????????0???????????????0??0?????????0?????0???????0???2
0???10?0????0???????????????????0?00?????????????????01?????????1????????0???1???0???0????????
???1??0

Dakotaraptor_steini
??00??101??????????
??????????????????0?1??????????????????01001???1???????????????????????????????01111000???0
0011001??1??10????00???1??????????0??????0??????????10????????????????200????????????????????
???????????????????????????????0???????????????????????????????[01]0?0?001000??0000000?00000
10??0?????0????????????????????0000????????0?0000??00??2????0??????????????1?11??????????????
?0000??
??0???????????
???????????????????20100??10????????????????010000000000?0???000?0????????00?????0???????0??
??????100?????001??0????????????????000[01]0??0????????????01??00?00001?????????0?????10???00?
???1??0?????0

Acheroraptor_termertyorum
???????????????????????10111??????????????????????????????????000?010??????????010010100??????
??
???????????????????0????0????????????????1100000??000??1???????????????00??????????????????
???????????00??0??
??
???????10?001?001?0?0?0?0??
??010?010??????????????????10??????????????????
??0?0???????????????????0????????????
???????????????0?00???11?????????????????00???????????

Boreonykus_certekorum
?0??????????????????????????????????????01?1???????????????????????????????????01??101??????????
??????????????????0??????????????????????????????0?1???
??1?10??
??
??00??????????????00??????
??1000?
?00??0??????????????????????
???0?????????0?????0???????0???????????????
??1???

Saurornithoides_mongoliensis
?00??1?1??1101???0?110001?1000????????2?????????????????1?010?100?0010??1????0001110101?????
????1???0??1?????100??1???02012020?010??10[01]1?111
10???????????0???10?01??????0??1?0???????????010??11000??0?1?01?0?????0?000??????0??0000????0
?1000?0??0??????????0000???0????00100?0???
???????????????????????00????????0?0????0000000000000?20?0210000000000201001?10???1110?
1???????????00?0100000??2??11?0?0?1?0100010100??00???0??0?????0??0?????????????00??????????

????????????????????????????0???????1????????000????????????????0????????????????0???????0000
0??????????????0????????0?0????????????????????????????1?010???????????00????00000????????00??
00????0?0???00???????????1??????0??000?0?????0??????????????????0?00???????0?101?0????????1??
?0??????0?0?00???????

Zanabazar_junior
?00101?12?110100?001?000??100000????2022000?21??0??11100??????100?001???1????000111010100
?????????????????1?1000?1020?1???2????????????????????
????????011????2???????????00??00???11????????0??1100000001000000????1??00???????00?000?????0
01??0?00?????????????0000??0?00????????0[01]0???
??00010000??000??2????1??0???00000201001?11???1110
?1????????????00??100000??2??11?0?0?1?0100010100?000???0??0?????0??0?????????10??000??00?0??
???????????????????000?00011010?????????????????000?00000????0000???000?001???????????0????
00000???????????????????0?????????????????????????????????0?????????????0?0????????000???????
?????0?????00???0?????????????????01?00?0000??????????????????????????????0?00????100100????
???????1????000?0????????

Xixiasaurus
??????????????????010001011 10??????2?22????????????????????0?00??001????????000201??01?0????
???00?1???
?????????????????0???00????????????????????1?000000???01?0000????00???????000000???00????????
???????????????0??
?????0??????0???????????????????????????????????????0?0?????????????????????????????????00?
???110100??2?01100?0?1?01000101000000???0??0???
????000??0??010?000????????????????00000?????????????
???000??????0??????0????0?0??????
????????0?????????????0100????????????????0????????????????????????1?010??????1??????????000?????
?????

Byronosaurus_jaffei
?????101???101?1100110001011?0??????20220???????????1?100??????0000001??11????000211??01?0?0
???????010121????????0??02???1????
0?0??0???????21??????????0????0????1???????????11000000?1?00????0??0?000??0???000000?????????
???0???????????????0?0????0????0010???
??????????????????????????????????000????????????????????????????00020001 1??0????????1???????
?????00??100000??2?011?0?0?1?0100010100??00???0??0?????0??0??????????????00??????????????????
?????????0???????????0?????????????????00?000000????????0??00??00?????????0???????0?0000?????
?????1??000?????????????0????00
0????????????????????????00?0100??????????????????????????????00?????????1?0?0?1????1??????????0
00?0????????

Sinornithoides_youngi
?0??01??????????????1?000??1??0????00???2???????1???????????????00??0010???????0001110?01??????
11??001???????????0011?10212101 1???1?00?1?101?0???100000????21??01??02??30???11?0??001??111
0?????0???1?000110001?0??0?0?1??00?0???0???0010?0??00000????0??0??0???00???0?2000000?0?????
???????????????00?????1?00??000??????????[01]0?0?????????0??0?20??0?00000?0??1000???0?1000???0
0?0??????????0??010?0000???000000??00??0??000????000000000000000020?0210000000????????101??
????1?010100??1??000????????????????????????????????????0??????????????????????????????????????
????????????????????????000??0??
0?0?00?????????0???????????????00?110101?000?000???????????????1??????0?????000???00?0???0???
0?00??0??1????0?????100????0?0?????????0???000?000???00??10?000?????0??00???0000000010?????0?
1?0?0101????0????0?????0?0???

Mei_long
?0????????0????????11010?????0?11?002012100001?1010??100??????00010010??1?0000?021???01?001
1011100011?2110?01???0110222111?1?????111?11100?0?1?1000001?012111?1??1200302?1110221?[01
]?0111100?0101001000??110011?0?0000???00000??000100?1??0?1?000000?001?0?0000?00000102000
0?000?????????00?0??0??00??0101????????0?0000010000[01]0?0????????0100020?00?00000?0111000?
0?0000000?0000000?000?01?1?0???00000????00000?00000010000??00000000000?000021?01100000??
0???20???1010??101100?1100?10?0000?110??0??0??????????0?1?01????0?????00???????????????0????
?????????00???????????????????1???0??????0????0????0????????????????????????????????????0?000???
??????????0???????0?0000?????????0????????????????????1?1?0?????????????????????????????0??????
00???00?00?0??????0????0????1?0?000?1001??00?0001?0?010???0???0?0000000000?000????00???0???
00?000000100?????????01??????????0?0????0?????

Jinfengopteryx_elegans
?0??????????????????1?01???2000????????1??00?0????1???100??????00?00??0??1??0?000?1???0???1???
?????????????????????02????2?200???????1101110??????100000?????1?????????????????????2??0??????????
0??????????????????0?0??00????00???????????????01?000001???0??0?0??????0????200?0?000??????????
??????????????????0??????0?000???0?0??0?0???????????????00?0???0?0?01???0?????????????????????????
??10?0000????????????0??11?01????????????
?000?110?????0?01??1????0?????????0?????00??????????????????????????????0??????????????????????????
???????0????0????0???0??00?????????????????????????????0??????????
???0?????????0????1??0????00????0???????????????????????0????????????????0?0??00??????00????1?0???
0????????0??????????????????000????????????????0?0???????00?????????001?0??????????????????????000??
??0?????

Anchiornis_huxleyi
000100??????????????11010??11100?????0?2?000??1???11????????????000??01??1?????00020???01??0?1
01???00?0??010?01???01101220??01?????01111110000??100000010?21?10011020?202?1?1122121???
1?1?000??1?01?000121100010?1000?1??00?0???????0?11?011000001???0??00?????000???030000?000?
?????????0??????0????0??????0??????00000?0000[01]0?0????????0000020000?0?00??0?10000?????10?
0???0???????????????????10?01000??0?00?00??00?00000?????????????00000?0?00??0100000000????????00
??1110?1?1?1100010??000?110??0??0?01?010?0?0?1??101?00??0??00??????????????????0?????????1???00
0??0???2??????????1?????????00????0????0???????????????????????????????????????0?000??01?????????0
???????0?0000?????????0???000??????000??????1???00??0000100000??0?01?????????0?????0?0???00?0
0?0??100?00??00???11?0?0000???????0???????????0????1000????0????0??0?0000000???00?0????????001
00???0???0?01??????????000???00?0?0?

Xiaotingia
?00?????????????????1?1???111000????0?2???????????11?????????????001001??1???00??020???01??0??0
???1?0?????10?01?????1??????10??????01?01111?10???1000001011?1110?1102122?????102?12[01]????
??????10????????1?010001010000?1??0?10??00???0010?011000??1???00??0?????000???22000??000???
????????????????????0000?????????0??000?10000???0????????010002?????????????11?000?0?00???0???0?
0?0????0???????????0100??1?00000???0?0000???????????????????????0??0?02????10000????????00??1????
??1?1100?10??000?110??0??0?01??1????0?????0???0?????0??????????????????0??????????1???00?????????
?????????????????????0????0????0??0??00???1?????????0??????????
?00??????????0???????????????????0011??????0???000010?000?????????????????0????????0???00000?0??100?0
0??00?1??1?0????????0????0???????????0?????000?????0???0??000?000?????0???????????0?100???0?????
0101????0???000???01?0?0?

Aurornis
?0010????????????????0101???11100???000?1?0???????????????????????????00?001????????0?020????1??0???
?????0?????????0????0??0?221?00??????01?01111??0???10000??001?011????02122020??10?????????????
???????????0??100000?000000?1??0??0??00????111?011?0000????0???000???0000???20000?000??????
???????????????????????0??????000??0???000?0???????????00???2??0?????????0?0?010?????????0??????????

???????????10?0??00??0?00?00?0000000???????????????0???0??????1??010000????????0???1?1?1??1?
?1000100?000?110??0??0?01??10???0?1??101?001???000???????????????0?????????10??000??0??02???
????00????????????00????0???0???0???1?????????0??????????0
0???????????????????????0???01???1???00??0000100000???????????????0???????0???00??0?0??100?00
??00????1?0???0????????0??????????0?????00??????0??????0?000000???????????????00100??????????
0?????????00????00???0?

Eosinopteryx
00010??????????????1?01??????000????0?1?0?????0??1??????????????00?001????????00020????1??0???
?????0??????????????0??0?231?10???????1101111??0???100000?001?1?1??1?02002?20??10????????????
???????????00?1??0001000000?1??0??0??00????011?0???00??????00?0?00???0000???20000?0000??????
????????????????????0??????0000?0?10000?0?0????????00???2??0????0???0?1?010????????0??????????
?????????????0??00??0?00?00?000?000???????????????0?????00??01???000??????????0???11??1??1??
1??0??0?000?11???0??0????????????????????0?????00???????????????0?????????10??0????????????????
??????????????0????0???0???0???1?????????0???????????0????
??????????000??????0??0011??1???0???000?100000???????????????0?????0?0???00??0?0??100??0??0??
1????0?0?0????????0??????????0????100??????0??????00000000???????????????00100?????????0?01???
?0????0????00?0?0?

Troodon_formosus
???1?1112?1101000001???0?011?0??????2022000210?0??01100????0?10??001??????????0111010100?
??1111100101211111?1000?1020??11??????????????1?010?????000010??????????0?0?2?20?11001?0??01
111000???00010000021??01????000??1?0??010???1??0??0?1?00000?0?0???????00???????????????????
??001??0?000?????0??1?1?????????00??
??0?????0000201001??0???1110?1
?????????0000??
???????0?????0000??00110????????????????????0000001000??000000??0000000????????????????????0
0???????0?0??00??????????????????????0?????????????????????????????????00000?000??0????????00??0
???0??0????00??01??0000?0?1??01?01?00?0?????????????0?0?????????????????0?000?10?0?10?0?0?0???
?????????0??0????????

Sinovenator_changii
?0???0002?0000111100101 01?1110?011?02??2??0011???1?10100????0?000?001??????000001 1110?1??
???11?101000 12110?001000110222?1?????????110111???????100?0???0?211?010112[02]0302?1110221
201?11110000??1001100011 1??110?000000???00001100010001100 11000??1??011??0000?00000??0??0
0000000000?000000?00?0??0000010100[12]00??00000?0010000?????????????????20000??00?0101?10?
???00?100000010000000000000?0?00?020?0110?0000000
0020?010?10??1011000???????000000?????0000?02?011??00?1?0100010?????0??????????????????????
??????????????????????????????0????????0?00????1????????????????????0????????0???00000??00???0?
???????????????????00??????????????00????0??????00111??????????????0000?0??0?01???????1??0????0?
0?0???0?0????????????0?0?1?1??0?????100?????????0????0??01?01?0?0000?00??????0?00??0???0000?00
00000001?1???0?????0??1??1?????000?0????????

EK_troodontid_IGM_100/44
?????0012??0???????????????1??11???????????1??????0?????
??1?00000???
?????01?1?001???00??????0?0??1?????????0??
??????1?1??
??0?????????000?????
???0??????????
??
???0??
????????1????0?????????????0000????????????????????0?1??????????????????????????????000???

IGM_100/1126
?0????002?0000?11[12]01?010??2000?011?020121000110?01010100??0??000000000?11?000000211??0
0?0???????????????????0????01102????????????????????????0??1000001???211?010?02002020001013101
2??111000????0??2?001210001?00000011?00?0?020000?00100?1?00000111?01?0?00?000003??????000
000?????00??00?00?0??0??0000?00?00???0?0?????????0???
????????????????0?00???0000???0100000?00??0???0?00?00?????00?1?000?20?02????0000001020?111?
?0??101?001???????0?000???
??
??
??
??????????????????????

IGM_100/1323
?00???????0???11???1?000??20000?1??020121?00?1??0?0??????00?0?000?0????1??00000021???0000??
?????????????????????011????????????????????????????????????00??1??????020?20200[12]102?1011???
????00???0????0??2???01??00??0????0?0???00?0?00100?1?000000?1?01?0?00?00?003??????0000000??
??00?000?00?0?????0000?0?000??0?00???
????????????????????????????00?????00?00?0?00?????????1000??2?????????????????????????????01???
??
??
??
??
??????????

Archaeopteryx_lithographica
100?0000??000??112010010??1110?011000012100?10?0000??100111?0?000001000002?00000200??001
00?1?1???00?0??1?0?0???002101231100????1011111111000?1100000001121110 10?12003020121022?
2?2?111100000?0001000000030?00000000011000000??00?00011001110000001001?01000000???0000?030
000?0000000?00110??00?0??000?00??000???00?000000000[01]000?0????????00000?0000?0000000100
00000?0011000?000??000?0000100000??00000?000000000000?0?00000000?00?0?[01]1000000?00???0
0000100001020?00001011101?0101100?100000001 10?00000?01?01?0000?0?0100?10100?000???0000?0
1??00?0????00???10??000?????????000000?0?0????????000??0011000?????0??????????00000001 1????
??00000?000000000??00???0???0???0?0100???????000?0000????????0?0011101?10000?000?100000??0
?01?01??????0????00?0???00?00?00?100000?000?1?1000?0?0010010??000000?0?010020?1000?000000
??0?000000?0000?000?????0?0000100001001???0111????00?100010?000000?

Confuciusornis_sanctus
10010???????????????1?000?00??0001???0??2??0??0??00??01???????000010000?10?0001?1?????????0??
????????1021?0?2???0?2????4??111?11010??13111000?11110000001121?1?1??12000?2?111023?2??12?
?10??11??1202110100 30?0000000001102000??00?0101??11?100011001??????0?????000?123001[01]00
120?????????10??00??011200??01[12]011111000000110000100100012000 0?00?0000?0??0??010000000
?000211??001000000001010210101 1[01]?10?0000001010000000000101010010001100001 1000?0000000[
01]000???200000110111 0?00??1100?10?0000?110?00000??1?00?0000?0?01?0?10100?00??????????1??0
0?0????0000000??000??01?02???00100000?0????????000??00???0?????????????????????????????????????
?00010???0?0??00??10?????????????0???????0001?000???0????0?00111?0?1?000?100??????0??0??1????
?????0????00?0???00?00?0??100000??0??1??0?????0?????0??00???????010???????0?????0?0000?0?0???
???0??0??????0?0000100?0?0?????01????????????1????1110111

Jeholornis_prima
10????0?????????1???0???0?????001???????????????????0????????????0000?1???00?00?1?1??????????0????
????0????1?0?11???02111031??01?0???01103111 0001??1000001011?1??0??01?0???2???1?221?10??????
?01????01011010030000000000??0??1000??00?010????1???000?1?????0???0??0??0??12300?10010??????
????????????0????00?00000??00?000000100000?0??001???0000?00000?0?1000120001000?0010000?00

0?0000?000010000101100000?000000000000?00000?01000000000100000100?00000001100????????01
1?1110???1?1100?10?0000?110?00000??????????0?0???????0?????????????????????0??????????0??00?
????????????????????????????000??00??0??00???0????????????
??????????????????0?0??0000???????0?0011101?1?0???000???0000??0?01?????????0????00?0???00?0??
0??100000?00??1?10???0?00???????00???????010???????0?????0???0??0?000?00???000?????0?0000100
???0?????0??????????1?1?1??11?0?0?

Jixiangornis_orientalis
100??????????????????0010?????001????0?0??00000??????0100??????000001????0?0??1??0????????00?0
???1?00????1??20???02??1031??11?1?00011031110001101000001111210101021200002010 1?221?10?2
?11?001??1?01[12]1101003000?00?000?0??1000??00?0100??111?001?1?????????00?000000?12300?000
10??????????????????????0?????000??00?0000?11?0?10?11?0?????0000000000?00100112100 10?0?0000
000?000???0??0????0??010?00100??0000?001000000?00??0000??00000100000?00?0000?001100??????
??00??11????01?1100?10?0000?11??00000??????????0?0???????0??????????????????????0???????????0?
?0???????????????????????????????????0??00010???0????????
??????????????0??????????????????????????????0?1?00??00???????????????????????0?????0?0???00?0
??0??100?00??0?????0???????????????00?????????0??????????????????0??0?0???????????????????00100
???0?????0????????????1????11?010?

Yanornis_martini
?00??????????????????1?010?????0?11???0??2??0??????00????????????000011???0????000?1???00??0???
?1???0????????4?1???????04??????110?011031110?011?130010?????????????????????????23?010?2??10
??1??0?0121[23]0000300000??0001???00?0???????0???21?1?0???????????????????0?????300100012????
??????10???????1??????????????????0?0011?00???0210?[12]??10100001010?1?11?112100111??0101?010
1?10000?0?????021[01]101311[01]1100[01]100?0?1?0??0?00??????0??00?1110[01]?1?00??1?0??01100?
???????01??1????????1100?11??001?11??????0??????????0?0???????0?????0????????????????0????????
??0??0???0???0???0????
???????????????????0???????0?0??0?0???????????00111?0?1?00??100???0000??0?01?????????0?????0?0?
??00?0??0??100000??00?1??0???????????????00?????????0?????000?????0????????000?00??????????????
?00100?????????0??1?????????0?1??11?0100

Apsaravis_ukhaana
???????????????????????0??0002?1?0??0??????????????????1??11?
?10200??1???5?100021??24??????1?01011031100001111 3003??000?1???1?201000?2?021?23?03?12?01
0?????1??12130100?00000??20???1?20?0?????0?00??21???????????????????????????300?10??????????
???????????0?1[01]?????1?0????????20010?[01]001??21??1????????00000?0?10001210111111?01011010
[12]0111110000101021110?[23]120100?11??20?1[01]0010?11?110?1012112111111?0000?1?0??01101??
??????011??????0?1????????0001??
???
??????????????0?????????01???0?????0??0?0011100010001?1?0?1?0000????01?010???????????0000??00
0????????????0??0??1????????00?00100?????0??????0???????0????0000000????00000????000????0?000
?10????????0?0?11????0???????????1????1

Yixianornis
100?????2?021??1?????0?01????0?1?1?0?????????????????0100??????000?010???0?00?00120???10?01??
?1???0000??1?0?40??0???0024???11?11000110311 10?00101300001000 21?101?21200302000 1023?23?0
211?0??1??????2130100100000??000?0??1000?20??0?000001????????????0??0??0???0??0?12300100 01??
?????01??1?????????11[01]?????1?0?????10?20011000?11021??[12]??10100001010?111101121001 11100
?0110???01?000???0?01021[01]10?31201100[01]100?00100?00?002?101??????11110[01]?1?00??1?0?00
1100??????00011?11?01????0100?10??000??1???
???????0???????????????????????????????????0???
0??????????????????????0??????????????00?0????????00111?0???000?100?1??000????01??????????0?????

0?0???00????0??100?00??0??1????????0???0????00?????0???0???????0?????0???0????000?00????00???
???0??00100?????????0?11????0??1??????1110100

Sapeornis
?001???????????????0?010??11000????00??21?0?00????0??????0????0000010??10?00000020??????00??
??1???000????0?211??0210004??100?????211011110100111000001011 21??00?212000?201[01]0022121
0????1??01??1001210?1003000000000????2000??0??0100??0111?000???1???00??0???0000??23000000
000?????????????????????????0?00???000000001000010 0????????0000020000?00000?010001000?0?01
010?0010000?0???010?1[01]10?11100??0[01]00?0000?0000?00?000??00?00??000001?00??0?00001000?
???????001?1?1011?1?110001000000??1?000000??????????0?0???????010??00?????????01??00?0??????
???00??0??????????0?????000??0????????000??00???00000???0
?????0???0???????0?0100???????000??000?0??????0?00111?1?1?000?000???0000??0?01?0???????0????
?0?0???00?0??0??100000?000?1?10?0?0?0??0?1???000000?0???0????1??0???0?0??????0?000?000??000
?????000000100?0?0?1???00?1?????????1?1??1?1011?

Neuquenornis_volans
???????12????????20?????????????????????????00?????00???
???00???????????????????????1?11011?1031110?01111000???????????????????????????????????010?????
??????[01]010030??0????0??????0?00????????????1???????????????????0???????0200?????????????????
?????????????????????????0110???????21?????10110??0100?001?001?0??[12]??1???01?0???0?????[01
]?????????2???20?1??01????????????????10?1????????1?0???1011???????0?000???200000110?????????
????????0??0??
?????????????00???0????????????????????????000??0100???
?????????????????????0111?????0??????????????????????????????????????0?0???00???????????00??0??1???
0??????????????????????????2????????????????0??????????????????????????0010?0?????????0????????????
??????1????1?

Patagopteryx_deferrariisi
?0???0010??
??2???????????????????????????????????????0?1????????????211101??????????????????????2?010001??1
00121301001?000???????1????????00?0?????1??????????????????????0?????0??????????????00?1??????
00??111110000?????????010000000????????0????????000????0101?1210[01]00???[01]?10?0???0100000
1[01]0001?[12]00???30???????00?2000000?010120????0?000[12]11001100000?[01]?0??0?100?1?200??0
1?0???0?001????????00???0?1??
????????????????????0??????0?0??0?110?????????????????????0?????????????????????????????0020000??00?
?????????0?0?00??0001?00000????????00111?00100????????1?0000???????0100??0?0???0?000???00????
0??????00??0??1??????0?10????????????00???010???????00000000??0?????000000???00?1???00000010?
?????1???0????0???0?1???10??1???0?

Cathayornis
?0??????????????????1?010?????0?1???????????0???????????????????00??????????????00001????????0?????
???[02]?????????3??????2????4???0??11?02?1031110?011?1300?0?01111?0?00?12000?201?1022?23?????
????1???0??[12]1[01]1?0?3000?0???00????0??1??0??0100???111??????????0??0???????0????2300[01]0?0
12????????????????????????????0??????0????0111??0010?20??1??10110?101????010?012000100101 10[0
1]?010201111 10?[01]?1?????210120?11?01?0101011?0?000001???1?0?0?1[01]1?0????????????0??01110
????????01????????01???????????0???110?00000??????????0?0?0100?10100?000????????????????0??????
????0??00???????????????????????????????000??00?0?0???????????????????????????????????????0??00??00?
????????????????0?0100???????0???????????????????00111?0???0?????????0000????????????????0??????0?
0???00?0??0??????00??00?1?1??0?????????????????????????0????100??????0???0?????00?00?????????????
???00100?????????0???????????000???11?0?1?

Concornis
??
???00?????????????????????0??11?02?1031110?0?1???0030?????????????120?102??11?23??3?0???1???1??

?0?1[02]11??0?3000?0???0????????????????001??1???????????????????????????2300?????????????????
???????????????????????????0111????10?20??1?0101100101?0?0010001?0??[01]001?110??0102?1???10
???1[01]1?[12]??????????????[01]????????????00?????0?100??1?00??1?[01]1??0??????110?????????????
????????1100110??01??
???
?????????????????0??????????00111?0?1?0????????????????????????????0?????0?0???00???????100?0
0??0??1?????????????????????????????0??????????????0????????0?????????????????0??0010???????????0?
???????????????11?011?

Gobipteryx
?0?????????????????1?01010?0?0??????????????????????????????11?000?0??????????1?1???????????????
??????????????????????????????????2??
??11???????????????????????????????????1????0??????????????????0????????1100100?00110?10?????0
0?????0???0??0???1?????????????10??????????11??10000?0?100012000[12]001?1?0110???0???????????
?1?121??[012]0?????1?0??1????????000?0???10?001111000?1?11?0???????????????????????????????????
??????????????00000??????????0?1?01????0???0?0???
??????????????????????0????????????????0??????????????????0?01000?000??00???000???????????????
???0???????????????????
????????????????????????????0???0???0??????????????????0
???????????

Vorona
???
??12?0100111010
01112110???00????????????????????0?????2??
???
?????????????????????????????1010111100001?000000????????????????????????????????????00??????
???
???
?????????????????????????????????????1???????00000000?00???????????????????????????????????????
???0?10001????????????????????????????????????110?00000010??????????0????0?????1???1????????0

Songlingornis
????????????????????0?0??00?????????????000?0????0?????????
?????????????????????????????11??[01]???3???
?????????0????0?????0?????00?????????????????????????????0???????0?????????0?01????????????????
??????????????????0???????????????21??[12]?000100?010???1?11011?????????????????????????????????
???0??0??????????????1??1??????????????????
??????????0??
??0??00??00?????????????????0?0?0????????????????
????????????1?1?????0??1??????????????
???????????????????00????????????????????????????????????0?1????????????????????????0??????????
?

Pengornis_houi
?0010???????????????10010??0?00??????0?12??0??????0?????0??????000?001????????0?0?00??00???01?
?????20???0???2??????????4????1?????2?1031111?0??????03??001?1??????????????????2??2??????????
1??????211???0?000??0??000???0??0??00???0???11?1?000??????????00????0001230000?000?????????
???????????????????0?????0???0011??00100????????01[01]00?00??????0??1??012111[01]0?011010001
[01][01]1?????1??1???10[12][12]0??1??1?11??????0????00?0????0??0[01]?1?00??1?11??0?0??0?100????
????01?????00?01?1001011???1??110?00000????0??0??0?0?0100??0?????00???????????????0??????????
0??000???????2?????????????????????0?0??0??0?0?0???0?0??0
0???0????????0?0100??0?0??0??1?0?????????????00111?0?1000????0???0000???????????????0?????0?0??

?00?0??0??????00??00?1??0?0???0????1??????????????0????1?00?????????0??????0?0?????00?1???????
00100???????????1?1????????000??????0?1?

Hesperornis
?0??????????????????00010100?00??????00021?001????00???00????1?000?001???1?0001?021???00??10
101110?2100??11?50100?210?24??11??10??00103111??????????3??0202100?1??01000???021023?03?1
2???0001??11?1213110010??0?00??01?1?0000?000???00??2??1?1111????0???????10000??10?1?10?112
1200110?1010??1110011100??00?0???10101210110000012?0?000100100?0???10?0???00011??0????????
?0???0????0??????????????????????????20?1100001112110211110021111122122002?0?????00??????00
01?0???0?000?0100?11???1??01??00000??????????0?1?0100??0?????00????????0???0??0??????????0??
00??????????????????00??0????????0?0??0????0????????????????????????????????????0?000???0????????
????????????000???????0?0??000???????0???01?????????????201??000????01?????????0????00?0???0??
00?0??????00?????1??0?0?0?0???0????????????????0?????000?????????0?????0000?????00?????????001?
0??????0???1??????????000?????????0

Baptornis
??
?2?????????5?????????24??
?212???????????????????????????????????2????????????????????????????????[12]????????????????????1
1001??00??1??????????12101100000012????012?0?????01?10?0???000????0?????????0???0??????????01?[
01]1[01]????????????????20?1100001112110211110021111?21020?2??????????????????????????????????
??
???0???????0
?0??000???????0???01???????0?????201??000????01?????????0????0000???0????????????00?????1?????
?0?0???0????????????????0???????0?????????0?????0000?????00?????????0210???????0????????????????
???????????

Ichthyornis
?????00????????1220?????????????????????000111????0????????????000??1?????1000??0210??10???1?0
1111?2100?1???5?000?21??34???01?100?001031110?011113003??0102?1??10212000????21023??3?12?
?10011??11?1213110???00?0??20?????00????0??00????21???????????0?????????????012300[12]0?11???
????????10[01]1?1110111010010 20???101?1200111?101[12]?2110220101000010 10?1111011211011110
0101101110100000101[01]101121111231201100 11?12001[01]0010?11211021111002 1111[12]2?100002?
0??011?100?20???0110??1??00?00100?11??01??01???????????????????????????0?00?00???????????????
?????????????????????????????????????1?0?????00????????????????????????????0????0100?????0?00?
0000?00?????????000?0???????00??000??0?01?0000??????0??00111?00100?0?10201??000????01?0????
???0????0000?0?0???0?0??1???00??0??1??????000??00100?0???001??01??2?0?00000?00000000????000
0?????00?111?0?00001????10?1?0?0?11?0??0????0??0?11?????

Iaceornis_marshii
??
?????????????????????????????1101?010311??????113?03??????????????????????????????????2??1??1?????
??21??????????????20???1??0???
????????????????????????????????21?02[23]11???1101010?11110112101????????????????????????????
??11131401110111020010??1?1?1211021211002 1???0100?11?
??1???
??
????????????00111???????0?10????????????????0??0??0??????0????????????????1??????????1???????????
00????0???00???01???????????????0??????1???????????????1???0?00?????????????????11?0??0???????????
?????

Limenavis_patagonica
??
??011?13???

????????????????20??
??10000[01]01010101?1
1[12]313011?011??
??
??
????????????????0?0???
??????????????????????????0000??

Lithornis
100????????????????01010100?00?111??2?02?00?0???????????????10000?00????02?101?1?????????1??
??????2????????[67]?1????????????[12]??11000010311110011?13003??000210101?20200102??2102????
?12?01000???11?12130100?????0??200?01?2010???0?0?0???21???12???????????00???00000??300?1121
2100?00011111010110?11[01]1101?1111?01?10121012?0111[12]1211122110100?01011011110112101 1
11100110001[12]1110000101010101111113130110[01]11102[01]0120110?1121102011000211112221 00
10[01]?000011?1????????011?111??00???????????01???1??
??0???????????????0????????????????
????????????????????????????????0???????0?0???????????????????????????????1??000??????????????
??????????????????0??0???????????????0000??????
???11???0?

Hongshanornis_longicresta
101?????????????????11010?????0?11???0????00???????????????????000?001??00????1?1?10???0??0??0
1??1?0????????[234]??????????4???1??11?01110311100011113002 0?0112???01?20????????2??23?23??2
??100?1??????21[23]01000000000?000?01?1????????0?0????1?1?0????????????0????0000?1?300???012
???????????????????????????00??????1?????0?????110????????01?0??11???0?10???21000111???110???
2????0?????????????1??2000??000100?0??0?0??000????????1???1100??[01]?00??2?0???1??????????????
??????????0100?11??001?11??????0???????????0?????????0??????0???????????????0??????????0??0?????
??????????????????????????????0??0?0?0???0?????????0?????
???????0???????0?????????????????01?1?0?000?0?10??????????????????????0?????0?0???00????0??1
00?00??0?????0??????????1???00?????????0?????????????????0??0?0?????????0?????????00100??????
???0?????????????1????11?010?

Liaoningornis_longidigitris
??
???????????????????????????????????3??1??0???100?1????0
?21211003000?????0??????????????????????????????????????0????????????????????????????????????
???????????????????????????????011?01??????????0???0???????????????????????????0?????01???2??
????????????????????????01?1?0??001111110201000?00???
??
??
????????????????0????????????????????????????0?????0?0???00???????????00??0???????????????????
??????????0???0010??????????0??????????????1?????????

Crypturellus_undulatus
10????012?020002?2001010100?00?111?12002100001011100010???1?10000?000??102?001?1?????????
1110111122100?01107?110??12?24??121?110100103110110111130 03??001210001?202201120021023?
23?12?01000100110121301?0???01000200001?20000200?0?001021???120??01??????0????00000103002
112121[01]0100011111010110011011111111100101012101011211212111 12?1010002101101011111210
0111110110001201100001110101011111031401011111020?120110?11211020211002111122210010110
??011?101020000011111100 00??1100?10??01??11??
?????????????????????????????????????0??0????
??2?????????????????????0?????

???0??????????
???0??????????????????????????????1110?0?

Gallus_gallus
100???102?021002?2000010100?00?111?120021?0001?1000001????1?1001000000000021101?1?????????
101[01]1111221?0?01007?120??12?24??1[12]1?1101201031102001111 3003??001211111?2?2001120021
023?23?12?0100011011??2130100100010002001 00?20100200?0?0??21???121??01???0??00???0000010
3001102121211111221101111111111110110001101011121010112011121111211010002101111111112
1001111101100012011000010111010111111314010012110 21?121111?11211020211002111132220 0111
?00?0110001020?000111111?000??0010110??01??11??
???0???0?
???2??????????????????????0??
???0???????
??????0?????????????????????????????1110?00

Crax_pauxi
1001011?2?021000??001010100?00?111?120021000011100000100??1?100000000000021101?1????????
?10001111 22100?111?7?120??12?24??121?1100201031112101111 3003??0002100?1?2?2001120021023?
03?12?0100011011?12130100100010002001?1?20100200?0?0??21???121??01?????00???00000103002
10212121111122110111111111111011000110101112101011201112111121101000210111111111121001
1111011000120110000101110101111113140101121102 1?111111?11211020211002111132220 01101000
011000?020?0001111110000?1100011??01??11??
??????????????????????????????????????0???0??????
???2?????????????????????0???????
??0???????????
?0?????????????????????????????1110?0?

Anas_platyrhynchus
100???102?021002?2101001100?00?111?10002??00?1?100010?0???1?10200000100?021101?1?????????
101?1111221?0?011?7?11???10?2410121?1101111031111101111 3003??0012000?1?2?2001120021023?1
3?12?0100011011?121311?0???010002101?1?20100?00?0?0?1021???121?????????00???000001130021
0212111111122110211121111111011000111101112101010201112111131201001010 10?1[01]111112100
0111001100012011000010111011111112314010101110 21?111110?11111010211002111 1[23]221001021
0?0011?10?020?000111111?000??0100?11??01??11???
??0???0???
??2??????????????????????0????
???0?????????
????0?????????????????????????????1110?0?

Chauna_torquata
000??1012?0210002?101010100?00?111?100021?00010100010100??1?100000000??0021101?1????????
?1011101112100?111?7?11???10124121 11?10000010311111011113103??0012101?1?2?200102?021023?
23?12?0100011011??21301001000000002001 01?20100000?0?0?1021???121??01?????00???0000012300
1102121[12]11110221101111211111110110001111011121012102011121111401010010101111 11011121
1021110011000120110000101110101111123140101111102 1?111111?11211020212002111 12222001001
000011000102000101111110000?11101?11??01??11??
???0???0??
??2?????????????????????0???
??
?????0?????????????????????????????1110?0?

Pedopenna
??
???0?

0?0??0010??
??
?????????????????????????????????00?000?????0??0????00????????0???11???????????????0??????????
??
??
??00????0???00???????????00??0?????????????????????????
?????0???0?10??????????????????????????????????0?

Epidendrosaurus
??0??1????????????????10???????????01?????0???????0??????
1?????????????????????????????????001??1??0??????001?????????????????????????????????????0????
????0000030?0?0???0?????1???????????????1?????????????????????????????3?0??????????????????????
??????????????????0???????0???????????????????0??0?0??????1???0?????????0???0???0??????????????
??0??????000??????????????????????????100?00000??0?0??0?010????????00??????1???????????0??00??
???0
????0???
??????0011??????0???????????????????????????0?????0?0???00?????????0?00??0????????????????????
???????????0??????????????0????????0?????????????????????0010??????????0?????????????????00?0?1?

Epidexipteryx
?01????????????????????1??????00?????0??2??0011?????????????????100?0?0????0001020???10000???
????????????0?[01]????1?1?123??10??0????0101?010?0??????00?1???????????022?????011?03?0??????
????0????011?0??0?10?000???00??1?1?0???00?0?00???0?0??????????1??2?????0???????2?0?????0??????
????0????????????????????????000?0????00??0?0???????????????0??0?0??????1???0??????????????00?[01]
0??10?0?0?0????10?00???0??????????1??11??1????
????????0?00?100?????0???????????0??????????0?????0??
??????????????0????0???00200??02?????????0???????0?1?????
????????????????????????0011??1???0?????00??????????01?????????0?????0?0?????????0????0?0????0??
??????0????????????????????0???????00??????0???0????0?????????0??????????001?00?????????????????
????0???????????

Incisivosaurus_gauthieri
?00?00?01?001??1??1000111010010001?010?1200001100010101101011121010000100?1?000210??00?
0???
??????????????????????00??00??01?0??0?0?????????10000000100010 2?00010?100??????000000000010
0001??0000???0000101?1100??0000??
??01020?00???0??000?0?0??
??????????11?1100000????1000000?0?0000?000?0?000???1000??1??00?0????000?110??010??01?0000?
00000???00?00????0100??0111000????????????????0010000000010000????00200??02?1?000000000????
?00110??100????????
?????0??0?001?????????????????????????0201100???0?0?0??????
0?????????000?0????????

Citipati_osmolskae
?001001001001??2210001011111?01011?00010210001100001000100011012l1120?01000101111?1????????
?1011101100101211001??201??0022001111?01112001101001000?1100000100021001??02012020??1011
1111?2101000000000?0000000000000000000011020000000?0?001000?000002011???0???[01]000020001
22000100000000110 2?1??0000??000001000101 0??000002001 1000[01]0?00?001000010102 0??????0????
111000?0?0010000?000000000000?00100?0??0?0???0000000????00000?20000000000000000000002?000
?00000000002 0?000110??00[01]10?011011000000011?1100001????1000001?0?0001?11000?001???100
2??0???0?0????000?000??000??01?0000?000000000000100?????100??0111000????????????????10100000
0010000????10201???2?1?0000000???0???10???00?00????????

??????????????????0?0?0???00100????000?00??0???0?00?????????0?0?0?00?0???0?00211??????????0??
00?000?0???????0??????0?0000100?0100????10?????????1?00?0????0?0?

Oviraptor_philoceratops
?00?0??????01?1????0??1??111?1011???0???1?0?11???01?0???0?11?121120?01?00?01?1?1????????????
??????????????????0????????????????12??1?????00???10??00?00??1??0????????????????????????????0
????0????0??0?????00?00000???2?00?0????????????10000?0?0???????0??0???0????00???0?00????????1
0?0?00??0??00?00?1?1????00???00?100????0????????01010?0?00?0?0????1?100??????1100???0???????
?00?????????000?0??00000??????????????????????????????????????0??000?????20????1?0???02?0??110
1100???0011?1?????1????10????1???0?01????????01???????????????0?????0???00??0?0??0??00?????00
??????????????????0??1020????2?1?0000000??????????????
????????????????????????1???1???0???100????000?????????
?0????????????????0??0???????211?????????00??????0??0??????????????????????00??0??????0????????
???00??????0???

Microvenator_celer
??21?20?0????????????????????011?0?
11000121100???0?1?1002????????????00?000?11000?????0010002???0000?????????????1110??0101100
?0000010???????0??0???00??11?20?0??00???0?1??0??????????????????????0???0?2200000?????????????
??????????????????????????0?0001????????????????????20000?000?01?????00?0??010?00?0000000000
00010000?????00????????0???0??000002000000000000????????????10??0??????????????1????????0???
????????00??
??0???00?
?0001?01001?????????????010000?0?????100???????01?01??0000????00??????????????0?????0?????????1
????000?10?000???0010?0001????1??????00??00?10??00000000??000?0??00000??????????1?01??11100
??001?????????0???

Caudipteryx_zoui
00010??????????????0?111??10?0001?10???21000???0?0?????????????2?1120?0?????????0?1??0??????00??
??0??00????1???0???01?????30?????0???0??0???01?0???100000?000?1??????0201202???1????101?11??
???0????01000??1?00?0?000000?1102000??00?0?001000100000?00????0???00?00000?1[01]2000000000
??????????0??????0000????1000???0000?00?0000?0?0??????????????20??0?0?000?11??00???????0?0???
00?00?????????????1??0000???0000000?000000000??0?0?00????0000000??0??2000000??0????????01101
100??01?110???0??000?10???0000?????0????0?0?0001?00?????0????????????????0????00???00??0?0??
0??0?????????000?0?????????1????0??00201???2?????????0???
??????0??0????????????????????????????1???????00??000???0000???????????????0?00??0?0???001?0?0?
??00?0???0????10?????0???000??0?00?0???0?0???11??0????00????0?000?0000???0?0?????0?0?00100??
?0?????????????????1?10???0??0?0?

Ingenia_yanshani
?00?0??????????????????1?1?????1?????????????????????????????????2?1120?01000?0111?1???????????????
?????????????1???01????2?00???101112001?1??00000??10000010002?1??????0201202001?1011?11101101
?0000?00011?000000000??0000??11?2000?000?0?001000???????????????????0002?0??2200?1000000??
1?????0?0??0??0000?00??1?10???000??0??1??0[01]0??02??????0101??0??0???0?0?0101000?????1100??
?00?0??????????????????00????0?00?00?000?0?000??0?0?000?0?0?0??00?0????00?000000????????0110?
?00?1?0?1101000??000?1??
???
?????????????????????????????0011000?001?0?01??101000??0101??11??0??????00000???00?????1?00
0000010??1?1?0??????10???0???0??0?000????11???0???0100000001000000000??000100?000000010????
??????0??1?0??00?1???1??0??0???

Rinchenia_mongoliensis
?00?0????0??????????0111?1???1?11?00010??00???0000?????00??1?12112??01000??111?1????????????
????1???????????????0?????220??????????2??1???00?0???1??0001000?1?????1???????????????1?????????

0????0??0?0???????0??00??001??2?00??????????????0??????00????????00??210??????2??0120????????
10?0?10??0???0????????????0??
?????????????????????00???0?00???002?0??????????
??00?1?1?00001????1??0001?0?0001?11??0??01???1???????????0????000?000??000??01?00???0?00000
0?0????????100??0????0????????????????????????????????0??
????????????????????????????????????1?1001?????????????????????????????0???????????????????0??
??????????????0???????????11????????????????????0??????????????????????0??????????0????????????0
??????????

Conchoraptor_gracilis
?0010???????????1???00111?1???1?11?000??21000110?00????1?0????121120?010?0?0111?1???????????0
1010110010??1?012?110??10????01?1?????20011000010???????0010002100010102002020??101101[01
]1?11010000??00010000010?000??000?001102?0?0000?0?0010001000002001???????0?0?0200012?????
?000000000????????10???0100100????????000??0?010???
???????????????????????????????????00?00??000002000000000000?0000?002?01?????????0?020?000?
?0??002?00???????????0011?1?00001????1??00?1?0?0?01?11??0??01????0?2????????0????0?0??00??0?
0??01?00???0?00?000?0?0??????100??0???00???????????????????10???0???????0????????????????????
???0?0??????????????????????????????0?0????010??
???010?????0?????0???????10???????00?0???????21?????????????????0?000000???????????????0?1?00?
???????00?????????????0?0????0???

Chirostenotes_pergracilis
?????1??01?01101??0???1?110?0??????????????????????01010??????21120?00000201???1?????????????
???1101?12????1?12???0????????????????101?1??????????00?100021?0010102212020110?111[01]??01
??100???00?100000200000??000?0?1??20?0?000?0?00100???????????????????000?????[12]????10????
????001????00????????????01?10???0????0011??00?????????????????20000?00000????????????????????
???????????????????????????00000?00??00000?00?0????????000000?0???0000?????001020?00???0???
?????1????????0?001???????????????????1?0?00???0??
??1000001000???????00200???2?1?000??0000??????
????0???????0?0??00???1000?0???????????????0??0??101000?????1?0100?0??0?00??00000?001?0??1?1?
000?01?????1?0??????10?????000??0??????02?1???0110?1???????000000000???00????00??00010?0??0?
????1?0??0???????0?1???000??0

Avimimus_portentosus
?00?0???10011?00??0??1?1???????1???1?????00?11??00100110??????2?1???00?0??0111?????????????01
1010110101?1?00??100????????????????????????0100??????????00?211?01??0201202010?011?0?10001
00?0???00011110020???00??0?0?01?02?000000?0?001000????????0?????????00?01??0?02001?????????
???????????0??0???00??0??????????02001000?????????????????????????????0?????0?0?0010000?0000
00000??????????????2???????????[02]0?000?0000020?01000000000000??00?01??0??????0?????????????
???????????????????1?0???
????????????????????100??0?1100????????????????????0000001?0?00?????????????????????????????????
???0???????0001000001??????????????00001???????101001??0101?0100?0?000??001000??00???????0?
?0?1001??101????0000100000?????1001101102?????101010000000????00?000???0010000000000010?0?
???1??01????0??00?1???1??????0??

Falcarius
??01?1110000111101 0?????1????00??????????00??????0000000??????100?000??0???????001010001?1?
?00011101012101000120001 00??0000??????0000010001000 10?0000100?0110010102203010101001000
1000000000000011000000000000??000?10?00?0?000?0?001000????????0?001???00?0?0?0??11?0??00
0???????000??00???0??00?00100?0?00??0000??0010000[01]0??????????0100020000?00000001110?1?0
?0010000?001000000000010000???0100???0000000?000000000200000000000?00000000?0000??0000
0?0?200000??0???00000?1100?00000000?????????????????000?00?00100????0???????????????????
???????????0?????0???????????????01?00????00?00????????????????????????000001010010????0??000

00001???0??????????????0?0???????0001000001000??1000??1010000000100000000000?0101?0100000
00001?0000001000?0??00101000010?1?01?11?1000100100000000011101002?0?1011110000001010001
00000??000001000?000000?0?10?10000?011011002?00?10?0000???

Beipiaosaurus
0??1???10?10?????????????1001001?100???
?1??????????????000??0220?????????0000??0????0?1000000?0000??001???20220??0??0????0??00?0??0
0??????0000????0???0??00111???0?0??0????0010?0??????????0??????????0???0??????0??????????????
???????????????????????00????001000??????????????00?00????????????????????????????????10000??????
?????1??0100???000000???0??0??0?????0???????0000000000???0?0?00000????????????10?????1?1100?
?0??000?1?0?????0????????????????????0???
??0??0?00???????????????????????0???????0
??????????????????????1???0?00??00???00?1????????10?????0?0?????????00???????1?1?0????????1??1
?????10???000?0?00????????????????????0??0?01000?01010???0?11??10??0?1?0???1?????????????????
?1????00?0?0?

Segnosaurus_galbinensis
??21??10?0000?000???0100?001???????
?1??????????1?0?0??????????????????0?10?1?00000????0???20011100102020221??0110201101?11001?
00?00?1100000021?0??00?01?1??0?0???00?0?010?00??????????0?????????00???????0??????????????
???????????????????0?0????0???????????????????????????????0??????0????1????1???0010000?00100000?
?????????????????????????00?00000??10??????00?????000000000??21?0????00????????????????????2????
??????0??
??
?????????10?????????????0?00???????21020010?0101??02????????????0?00???????????????0??01??0??1??
1????????2???????0??110????????1???????10100??1?111222101111111112?111201??????????0???1?????
??????????????1?

Erlikosaurus_andrewsi
?0012???2?0?1?1??1010011100??0001?1000001000010000000000??1111210010000002000l?001001001
???0??
?????????0000?021?0??00??11??00?0?00????????????0?00000010010??00000?000????0?000002000001
???10?0100???0000?0?10000??0000???1?0?0010000?00100
00000???00000000????????0000?0????000?????000?0?
????????0????01?0001100????1?10000?0?00?0?100000000???0??0?00??00?0????000001000000??00?02
00?00000101000?00001?000??00?0?00?????????????????0000001101????0???0100100020000000000000
00??????0???????????????????????????????????0?00???0000???
????00??0???0?011????????2???????????????11210?11???????1?0?1????????????????????????1?0100100
0????01??????????010?0???????1?

Alxasaurus_elesitaiensis
??210?100????????????1001001???????
???0?01010000?1?0101002?0?1????????????0?000??0?10?0000120?11??0??1?20?21???11??????????1????
0?????????00000?10000??001?1??00?0??00?0?0???00???????????0??????????0?????0????0??????????????
??????????????????????0??00????0??00??[01]0?????????????????0?????????0??11??01??????0?0???010000
???????????10?0100???000000???0??0??????????????????????????????????1000000?0?????????0??????????????
????????000???
??0??0020??????????????????????0????
????????11101?????1??0?11??0?00??010?0?????01??????????2????????????????200??0????1?1??0??0???
?1??1?100???????000000??1??0???0?00???11?0????01100??11?1?????1111?????1?00???10?1???0?????
???????1???????0???

Neimongosaurus
???21????0??????????????0??001?1??000

?110101?1?0?10?2?1?1?02200???????0001010000?????????????11001?2????????????????????0??00
????0??00000021?00????0??1??00?0??00?0?????0??????????0??????????????1[12]200?0???????????
????????????????????????0?????00?0000?0?????????00000200?0?00000?0?11001?0?0?10?0???010000
?0?00????????????????????0???????????20?00???????0?00000000?0210??????0????????0?????????2?1
100?000??
???0????2????????????????????0????
???0??1??11???????0??01?10?0?00???????21?2?????????????2?1???1?00??00000?1???0??00????00????00
??????100010?2???????00?????1???0?0111?111001011????1?1121?????111111????10????1??11??0?0??
0?100???1?1?????????

Erliansaurus
??
??10??1??????????1???????????????0?0???10?0?0???00000????1?0?1??????????????2??1????10?00?0?0
001?0??00??????????00????????????????????0????????????????????0??????200????????????????????
???????????????????????0????0??????????????????????????????????01?0?0?10?0???010000???00010?
??????100???000000???????????20?00???????0????????????????0??????????????????????????????????00
0???
???0????????????????
??????????????0?0?1?0?01????????????????02?0???1?000??????????????00100???????0?????????00???
100?????00?11????????????????011?111111??11?1???0?1?11111011?0?????????1??????????0?1??????0
0?0???

Suzhousaurus
??
?0101??01000??0??1????0?????????001010000???????????200111001?2020?2?100100201101011100?00
?????????????????0???????1????????00???010???????????????????????0?????0?0??????????????????
????????????????????????20010000[01]???????????????20000?00000?01010?1?0?0010?0???0100000?
?00?????????????????????00?000?00?1020000????????????????????0???????????????????????????2?????
??
??
???10111100100000?11010?00??????021020010?0101?00201010???????????????????0???0??01??00?1?
???1000?0?2???????00???????????????11?100011????1112212111110??????????????????1?0??01102?0
?2????????????1??

Nothronychus
?????1112??11?1?0?0????????????????????????????????00000???????????????????????????1??????????
??10101?10?01?1?00?100220?001?????00000?100?00????00000200??10?1?2020221100100201101?1100
100?10001100000021?00???00??????0?0?000?0?010?00???????????????????0????1[12]200?????????
??1????????????????????????????????0010000[01]0??????????00000200?0?00000?0211001?0?0?10?0??
?010000???00010??????000???00???00?000000?1020??000000000?00000000?0210??0?000?0????000??
??????0???1100?000?000???
???000?01100???????????????????????????????
???????0???????0?0??1010110???0000?110?00001?0?0?021?20010?1101??02???1?10000?0?000?10?????
0?1?0000010?0??1?11?1?0010020000?00001110?1?2??????1?1110?0001???11112221011111?1210112
0??0????1?1?0???10?????1???10????0???

Enigmosaurus
??
??111?01?2020221100??0201101???????????
?????????????????????1????????00???010??
??
???????????00?000000?10???2????????????????
??

???1??1
?????????????????????2?020?10?1101??????????????????????????????????0??01?????1?????????????????
?????????????????????????????????1?122121100??

Nanshiungosaurus_brevispinus
??1???????
101?121???0??
???0?????1????????????????????????????
??????????????????200??0??
??????????0???2????????????????????
???
???0??????????1???1??0??????
??????????????????21?2??10???01????????????????????????????????????01??0??1???????????????????
????????????????1???????????????11222??11???

Therizinosaurus
???
?????????????????????????????????0010?0000????0?00200?????????????????????????????????????0??0?
1100000021?0????00???00??????????????????????
??????????????????????????0??????????????????2000??00000?01?1001?0?0?10?0???010000?????010??
?????100???00000??????????????????????????????????????0??0100????????????????????????????????00
???
???
????????00?1010?00100101???001?0????????0??????????10??
0000?????????????????????????1110111111?????????????????2?111?0?????????1???01????0????????????0
???

Haplocheirus
?0010???00010?0?120110111011112001?000020100?0?1010??0001?00100000010110110?1000001101010
100??10??0?0111201??0?00001?0???0?00???????100210001210?0?00010???001?00?101002010010?011
000?00000000?0000?0000000??00?1110001100010001?0???0010?01000000000??0000001?00000?10200
0000000????00???00???0???000000??0??0??0000?000?000????0?????????????20000?000000010101????
??100???000?00???????????0???00000??0000000?00?000100200000???????000?0??0???0100?000???0?0
??????????00?0??????????0?0000001?00001??0?0000000?0?01000000?0??11???0??0?01??01?0????0000
01000000??00??100?00000000000?000????00??0??0?0??????001????????0?0??0?0000????0???00000??0
0?00000??00??????????000???????0??1???????????00?1010100??0100?00?0????????0?01??0???00?0???
??0?????00000?00?101000??00?0??010?11001001000100000110??00??0?101???00000???0000?????????
000????????00101???0?1?1?010?????0??0000??10000???

Alvarezsaurus_calvoi
??01
000???0???0?20?0?2012????????????000?00????????0??1?0000101?00?2??????????????????01?000??0?
?0??11000000??00????????1???0?0??00?0?????0??????????????????????0????10??????????????????????
?????????????????????????00100???0????????????????2?000?????0?0?0?????????????????????????????
??????????????????0??000?00???2????00000000?000???00?02???0??????????????0????????0??????????0
0??
??0???????0001?
00????????00?10?0?0????????????000?000?????????????????0??00?????00?????????????0??0??0????????1
000???????10?????101????????1????00?????????00000???????1???0?000?10??????1???0?0??0??0???????
?????????

Patagonykus_puertai
???1?
01?112????012010?2?2???????????????00210???210?????1?0???1????1010?????20????21202??11000000

000001210??0????001??0???????0?????????0????0?????????????????????0?????01???????????????????
?????????????????????????0?00100???????????????????20000?000?00??????0?0?0000??0?0010000???0?
?????????1???????????0???0????100200000000000??00?0???????10??0??????????????????????????????
????0???
??
?00001?01100?0?????000?1100??????11???0?0?01?01?1?000?10??1????0?00?????0????00?000?0??1???
0??1001?101??1?0?1111111????????????0?00000??0?????00??0000?00?0?00??1?????????10??1???0???0
????1??0???0??

Achillesaurus
??
???????????020???2??????????????????????????????????????10??01?2????????????????????1??????0??1?
01000??0???00????????1???????????0?????0??????????????????????0??????????????????????????????
??
????????????0???????????2????00000000?0??0??0???
??
??
??????????????????????00?0??????????0????????00?0????0??????????????????????????????11?????????
??????11?????????????0????????????000???????1???0?00?????????????0?????0???????????????????

Mononykus_olecranus
??????00???????112????????????????????????????????????100????????????????????????2????00???????1
?1?11102001???2?10?2??????1???1000?000200030210?02201100??1????1?201000?2?????3??0??021010
01110112120000300000001??00???1??0?0??00?0?0???00???????????????????????????00011?????????????
??????????????????????????????0?0010?0?????210???00?????2?010?0?0?0?010?011?0?0001000?001000
000000??0000???30000???0???0??00???????200000000000??0000??0?00?0??0??000???????01?0?????0
?0?????????11102??
???0?
??????1001001000????????1010100000100?????011???0???01?010000010101?100000100????0??????00
??1??0???021?111001011111111101011???????010000000000?000?0000??000010000?000?10???????
?100?10100?0?01???1??0??0?00

Shuvuuia_deserti
?00101002?01011112011000101 1?01111?100201000010100100 10???1110?0010100100210 0??0211??00
0??0111111011102100?0?201012012110100?1000?00020003021??02201100001010 01?201000?2002203
??03?02101001110112120000300000011000001100000010000?00??001000000001?010??000000000?00
0100000000?101000?10000110?0111000??0[12]0????0100?0010000[01]0?0210?0?00?????20010?000?0
10101011?0?0001000?00100000?000?????????30000??000??00?000?00?1020000000000000?0000000?0
0100000?0000020?0001?01?1010001???????1110200?000?000??0?1??0000?0?00000?00000100???0??0?
00??01?0????0?????0??000??00?0200?000000??0?0?0??00?000?001?0?0???????????????????000000000
????????00000?000?0??000000??00??????0?0???????0001001000101 1?0001010100000100??0?0111000
0???01??1??????????0???0????00001????1??00??1??00110210?110010?1111111??0?01002?0??00??0?00
00000?000000000??000?????0000001010 1?0??0?00110????00?1101?0????????

Parvicursor
??
???10?????????1??2??1????2?1000???02203??03???2??10001 1?1
1211000030??00?????????1??????????0?00??0??????????????????????0?????0???????????????????????
??????????????????????0?00?00???
????????????????0?????????1?200000000000??000?000?00?0???????????????0???????????????????11??
??
???10
00?????00??????????????????????????0???01??10??00?0?01??000??100???????????00??1?????????1?111?

??????????11100010????????????0????????????0?????000??0000000010??????????0???1?0????1???1??
???????

Albertonykus
???
??211?????1?0???
?????030?????????0??
???
??0??02???????
???
???
???????????1??????????????????????????????0???00????0??????????????????????????????????11???11??
1???0????0???????????1??????????????????????????

Albinykus
???
???0????????????????????????????????1101??
121100300000?????????????????0000?????0??
???
????????????0??????0????????000000000?0000000?00??0???00?????????0???????????????????11???????
???
???
???0?010000??00???????????00??1??????????????????????
???00000?10??????????0??????????1?????????????

Bonapartenykus
???
???112??0????????????????????????000210???????????????????????01????????????212?2??1???????????
?????????????0???????????0?0???????0????????????????????????????????0???????????????????????????
?????????????????????00100???????????????????20000?000?000??10?????????????????????????????????
??????????????????????100??????????????????????????0??
???
??0?1000??1???
??0?010?010??????????????????0?0?01?????????????????????00?????00???????????0??????1???011????
?????????????????????????0??????????????????0?????0????????????????????10????0????????????????

Ceratonykus
?0010???2??10?????????????????11???10?201?0?0?0100??0??????????????01?0???????????????????????
??1?1??????????????????????????10????0?2???0???1??????
??212?0003???00????????????????????0?????0???????????????????0????????????????????????00???????1
????111????????????????????????????21?????0??????0000?0??01????????????????????0???????????????
?????????????????????????????????0000000??000?000?00?0?????????????????1????10?0??????????????
?0??0?????????????????????????????????0?????????????1?0????0?????0??000??0??02???0?0000??0?0?0
??00?000??0????0???????????????????0??0?00??????????0?0????????????????????????????0???????0001
???0?0??1000???????0??????????????????1?????1?
00?0????????????1??????????????????????????????????????00?00001????????????01??????????????????
??????

Linhenykus
??111?
101?1??1?0???2010020?2???????10?0???1??????????0?21110??????????2??????????????????0?1010001?
???2?2?0?030?0000???0????1??????????0?????0???????????????????????????01???????????????????????
???????????????????????0?0012?0?????210????0?????20??0???0?0?0???????????????????10000???0????
??????31?????00??0??????????1?2000000000000??000?000?001?????0?0?????????1???????0???????????

??02??
??0???????0001?
01000?????0??????1???????????????????????????0?0??00?0?0???000???00?0??????1??0?????????????1011
???????1111?1???0???????????????0????????00???????????01??00?00001???????????0?1?110?0????????
?0??0???

Xixianykus
??
???102?00?21201??0????????????????????????????????????0?01?1??1?200000?20022?3??03?02?0100111
01121211??3???000???????11???????00?0?00??0??????????????????????0????????????????????????????
?????????????????????????0?0010000???
??????????????????00?000000?1?200000000000??000?000?0???????????????????????????????0?????????
??
???1
000?0???????????????????????000110000???01?010??00?0?00010?0????????????????????????0??1???1011?
???????????111??11???????????????0???????????0?0000??00001000000001??????????0????100????1???1?
????????

Nqwebasaurus
?0?????????????????02????2010????????0100?00??1?????0?00?????????????????????????02????1??????00
?11001???1???????????????????????????0102?000??0?00?10110?????????????????????????????00?????0?00
00100010000000?000?1??00??????0?0?0?????0????01?00???00?????????????????10200???????????????
???????????????00???????????????????????????0??????????????20????0?0???0101?0??????10?0??????????????
??????????00000??00000??????????00??????????????00?0????00??0?0??00000????????0??????0?0???????
???0?001??????????????0?0??????0???0????????????0???0??????0?????????????????????????????1????????????
????????????000??0??0?0?????????????????????????00??0??????
????????????????????000??01?0?1?0?0??????????????????????????0????00?0???00?????0?111?0???1??0??0
12?????100??0001000010000 1???????0???????????????0?????????????????0?000010??????????0?????????
??1?1???00?0???

Shenzhousaurus_orientalis
???0?????????????????21000??10?0??1???000??00?000?0??????????????0?00000000??0??0?1?12?0??1??????
??????000??1???0???00000???0???????????????????????????????010000000??11000100101?000001100??000
01?0?????????????????????0??1??1?0011?????00???0010??10000010????????00??0?00???0???00?000?????
????????????????00?????0??????0?00?0?100?0?0?0??
?????????????????0?????0?00000?00000?0002?000??????????????????????????00????????????????????00000??
?????????01?00??00000??0?0000?00?0?0000000000??00???0??0??1???1??????????????????????????????????
??10???????0????????????????????00?00??00?00?0????0??????????????
?????????????000???????00??????????????????010100000?0001?00??0????????????????????01?????0?10??00
??1??00?2?0000????????100?0?????????00?10????00????????00000000??00??????????????0???0??0????1?
??????????1???0???0???

Ornithomimus_edmonticus
?00010?1101101?101021000?010101011000000000000000001000000??????00000000010?1001?1???????0
?001?1011000011100010100000000100100??????011200120000000?20200100000001100011010110000001
1001000001100010001000000 20??00?0111111101211110000?0?001000100000000 1?????0001?000000002
100000000?????????0?0?10???000010110??????000000010000[01]0?0???????????????0??0?0?0?0?01?10
01?0?00?000???0000000??00???????1??00000??0000020?000?0000020000000000000?0??0?????0??0??00
0?1????????0???0??0000000????????00010000?00000?00?0000?01?0?0100000??0??00???0??0001??01?0
????000001000000??01?00????0000101?0000??000000??0????0?????????1??????????1????????????????0
0210???0?00000?010?????????????010?00?00001000011????????????????????10?00???1?????????????????
????????1???????0111100111111111?11000???????100??0??00?01????101?0???1??0?0000?1000000000
0????00000000000000100?0?????????1????????????10?01???0???

Archaeornithomimus_asiati
???00?101
100001110001?100000000?0?????????010200?20000?0110[12]0???000?011000110101100000102010000
001100??000?0000020??0000?10??10??1?1??00??0?001000??
????????????????????????????????0?001000?[01]0???????????????20?00?00000?0?0??01?0?0010000?0
00000000000010000???00000??000??00???00000002000000000000?0000??00?0?10?000?????????????0
??????0?0????????00001??
???
?????0???????0??1?00001?00??00000?00020010?010?010100000?01011?00000000000?000001000?????
001?101?001?11?0????000010000001100001??011???????110?0000011000000000000??000000000?000?
10??????101?0?1?000?000????1?????????

Anserimimus_planinychus
???
?????1?????????????????0??????????1?200?2????00?202001000000110001101011????001?001??????????
??????00002???00????11??10??1?1??00?0?001000???
??0????????0??1??????????????????????????????
?????01000??00000????????????????????????10000??00??0??0??00?1????????????????????????????0
01???
???
?????????????????????10?0??1?1?????????????????????????1?0??????????1101101100 1??1?0????????10
0???01?00????????????????????000?10?000????????????????????010????????????????????????????00
00???

Struthiomimus_altus
?00010?110??0??101021000201110 1?11000000000000000010??0001?01??00010000010200 01?1????????
?001?101100001110001010000000100100??????0112001200000012010010000001100011010 11000001
?0010000011000100010000020??000011111110121110000?0?001000100000000 1??????00?1?0000??221
0????0????????????0?0?1????000?????0?00???00?0000?0000[01]0?0??????????????200?0?0?000?0101001
?0?0010000??00000000000?100000??00000??0000000?000000000200?00000000000000??00?02100000
0?10022000000?0??0?00?00?????????0010000000000?00?0000?01?0?0100000000000???0??0?01??01?
0????0000010?0000??01?0000??0000101?00?0??000000??00?0?0????????????????????1000011010?00???
?00210???0?000000000????????????00??00?0000100001 1?????0000000002 00?110101?10100100?01011
?00000000000001000100000111001111111?1?110000??000010000001100001000110100???1??0?00001
100000000000???000?000000000100?0?0?101?1110000?0000?1010???00???

Gallimimus_bullatus
?00010?11011010101021000201 1?01011000000000000000010000001?010000000000001020001?1???????
??0011101100001110001010000000100100??????0112001200000??20100100000011000110101100000
110010000011000100010000020??00000111111012111 0000?0?001000100000000 1?????0001100000002
21000?00000001000000?0000??00000?0?10200??1000000010000[01]0?0??????????????20010?00000001
01001?0?0010000?000000000000 100001??00000??0000020?00000000020000000000000000?000?021
000000?1002??0000?00??0000000???????00001000000000 ??0?0000?01?0?0100000000000???0??0?01
??01?0????000001000000??01?0000?00000101000?000000000?000?0?000???????????????00100001111
00000???00210?0?000000000000000????????000000?00001000011[12]0000000000000020011?01010101
00100?010110000000000000000100?10001110111111011001?110001??000010000001100001000110100??
?11000000011000000000000??00000000000000010000?001010111000010000?1010?00000?0

Garudimimus_brevipes
?000?????01101????02?00020101000??000000000000000000?00001?010000000000000002?001?1???????0?
?0111????0001?10001?100??00?????10??????????????????????????????0000001100?????????????0??00100
0001?000?000?000001000000?01??1110?2?1?0?00?0?0??00010000000 0??????0000100000010???00?000
0?001000000?0000??00000101100000??000000000 10000???0???

?????????????????????????????????00?000000000200000000000000000000?021?0????0000????00???
0??001000????????00???0000000000002?0000?00?0?0100000000000???0??0?01??01?0????000001000
000??01?0000??00000000000000000000??00?0?000??00?010???????001000000010???0???00210???000
000000000000????????0?0?00?0000??0001110000000????????????????010100100?0101?0000000?0000
00100?10001111??????001000?1?0001??0000???????????000010110100??????000??????????000000??0
0000000000000010000?0010??01??0011??00?111?0?0???00

Pelecanimimus_polydon
?00???????1????????2100?2?1??0000??000000?0000??????????????0?000?0000???????000211??0001??0
00???0?0?111?00????????????????0??0?00???120????00??0?2010?????????????????????????????????????
???????????????????1001010????0?????0?????0????10000??0?1?0????00????0000?0?10???0?0?????????
?0?0??????????????????????00???0?10??????0???????????????0??0?0?0???0?????????????????????????
???????1??00000??00000??0??000???????????1?????0?00???????
????001?00??00010??0?0000?00?000000000000??00???0??0?01??01?00?0?0000011??000??????????????
????????????00??0?000??????????0010000????0??????????????????0??00??00????????????????0000000
0?10???????0?0??????????????????????0?01?????????????????????????????????????010?1??111??????0?
???0?2?0?????000001100???????????0?1101???????????0?000???????????????????????????1???0??????1?????
?????111??10000???

Harpymimus_okladnikovi
?0??????????????????2100??????0??????000000000000??????????????000000?0000????1?1200??1???0??
?1???0?00??10001?100000?00?001?????????0?1?00120000000000010?10??00???????1????????????00?00??
????0??0?010000010??000001011????1?0???00?0?0???001??0000??1?0????00??0000??11?10000000???
?????????0??????????????0000??00?00000?0000[01]0????????????????????????????10??01?0?0010000?
000000000000???0?010?00000??0000000?0?0000?00?????00000000?00000?00??11??0000?0????????0?
??????000??????????0?001?000000000????0????0??0?0?????0000?000???0??0?01??0???????????????????0
???1?00??????0??????????????00000??00?0??????????????????????????????????????00210??000????????0
????????????0???????000??0000?1??0????0000??2001?00?01?1??0010??????????0??????????00?0??0001
100100011??0??0?11?00????000???000001000????011???0??01???00000110?000000000???0?00???0?00
0?100?01??1?1?01??00??00???1????0000???

Beishanlong
???
??????????????????????????????????010200120000????0100??????????????10???1??00??????000001100?1
0001000?01??000?0???1???0??1?1????????01000????????????????????????????210????????????????????
??????????????????????????????????0??0????????????????20010?0000000101001?0?0010?00?0000000000000
10?????????????????????0???????????2000000000000?00000?00????0??0?0?0?????????0?0????????????????
?00?01??
???
???????????????0000002011??????????????????????00??????0?00000?0???00?????00??1?0???0??1??????
????1000000?1??00?0?011??????????????000100??00??????????????0000000001?????????1???10?0??0??0
???1??????0???

Sinornithomimus
?0001?????1?0????1?21000??1010001?0000000000000000000?000??0???00000000?00????1?1?????????0
001?0?10?0???100?1?1??0?00?0?0?100??????010200120000?01101001000000110001101011000001?00
?0000011000100?000?010??00000101110121?1??00?0?0010?01000000001?????00001000000?021000
00000??????????????00??000001?1?0??????00000001?000?0?0??????????????20010?00?00?0101001?0?00
10000?0000000000??01000010?00000??0000000?0000000002000000000000000000??00??1100?0000????
?????000???000000????????0?0010000000100?00?0000?01?000100000??0?000???0??0?01??01?0????00
0??10??000??01?0000??000??????00000000000??00?0?0?0??????????????????10000????????????00210??
?0?000000000????????????0???00?00001?000?????0?0??000000200?100?010101001000?0?010000?000?0

0?0??1?0?1?00111?1001110?1001?11?00???00001000000110000100?11??00???110000000110?0000000
00???000000000000001000??0?101?011?000?0?00?101??00?0???

Qiupalong
???
??00??1100????????????01100?????????0??00
01000002???00????????1????????00?0?0???0???
??????????????????????????0??
????????????20?0?0?00000?????00000000?0000?000?02????????1????????0??????????0????????00?????
???
???
??????????????????????10100?00?0001??????????000?010001??0??????????1??110????0??????????????
???????1??01??????????????????????????0000??000??0000?00001??????????01??????1???????1????????
?

Kinnareemimus
???
???0??
?00001???00????????????????????0?????0??
???????????????????????????0???
?????????0?????????0??????0000000000000?000?02???????????????????????????????????00?????????
???
???
??????????????????????????0???????????????000??00000?00???
??00???1?????????????????????????????????0???0000???001???????????0??????????????1?????????

Huaxiagnathus_orientalis
?00?0???????????????0001???1???0?????00??0?????????????????????00?0010????????00010?1010?00??0
???1????????0??????00???0?002010????000000000?00??010000000020?0??000100201?000012?0???0??
????0??0?010000000?0?011??000????00?0??00?0?0010?0100000?0?1?00????0????0000??200???0000??
????????????????????????????????00?00?1??0?[01]0?0????????0000020?00?0?0?0?010100??????10?0??
?0????????????????????010????000??00?00??00000??????000?0??00??0??0????100000000????????000??
??00000?0?00??000000?01??00000??0?00?0?00?10000000000???1??????????????????????????00?00???
?????????????????????????????????0???02?00???0?????????????
????1?0?00???????0?0??0000??????0??0001100???1?0?000??00000??0????????????0?????0?0???0?0?0?
00?101000??01?0?00?0?00001000?000??0???????0?????10?????00????0?0?0?0000????0???0????000010
0??????????????????????000???00?0?0?

Sinosauropteryx_prima
000?0???????????????0001???1???0?????0000??????00??0????????????00??01???????1?0001001010?000?
01??100????1?0?0????00?110000201??????00000000?10??010000?00020??????0100201?000?11?0?1?00
?0??000?0?01000000000?011??0000??000?0??00?0?000??010000000?1?00?00?????000?0??20000?000?
?????????????0???????0101????????0000?0??0?00[01]0?0???????????????0?00?0?00??010?000?????10?0
???00?00?????????????1??0100???0000000?00000??0?2?????00??0??00??00?0???0100000000????????00
0?10???000?????????0000?01???0000??1??1????0?1?0?00??0??????0??????????????????????????00??0??
??????????????????????????????????0???02?00???0????????????
?????000100???????0?0??0010??????0??00111?0???110?000??00000??0?0???0??0???0??0?00?0???0?00
0?00?11100???0000?00?0?0?001000?0?0?0??????010????1100???0000???0?000?00000??000???????000
0100?????100??????????????000???0000?0?

Compsognathus_longipes
?00?0????????????????00101?1??00???00000????000????000???????0?00000100?1?0010000100101010 00
?01??1010???1?0?0???000?0120002010?????0?000000?10????0?000000??????0?01002010000012?0????
??????00?000100000000000001100?00????0?0???00?0?0010001100000001?00000000?0?000??020000?000

0??0?0??0??????0???0000????01?0??0000000010000[01]0?0????????00?0?20000?0?0?0?010100????0?1
000???0???00??????????????010????0?0??00??0?000?00??????00??0?0000?00000?0010000000????????
00????000000????????00000?001?001000?1?0100?0001000000000000??0???0??0?000?00?0????000000
0?00?0??01?12????????000???????11?000??????????????????????????0??????????????????02000?000000
20000?0????????1?0000???????000??0000?0????0????????01???1????00????0??0?0?0???0???????0??0000?
0???0?000000?101000??00?0??0?000?0010???0?0????0????10????1100??0000????0???0?00???????00???
????0000100?0010100??1?0????????001???00?0?0?

Juravenator_starki
0000????????????????00010??11000?1???00000?0000?0?????00?????0?0000010???0????000100101??00?
?0???1????????0???????00??10000201O????000000000000????0000000000?01100????????????????????0??
???00??????0?0??000000?10?000?0??0??0??00?0??????010000000?1?00?0?000??0000???20000?000????
??????????0?????????????0??????000?00???00??0?0?????????[01]00002???0????????0101000?0?0010000??
000?000?000???0?0????100???0000?0???00?000??2??00??????000??0?0000?0010000000????????00???
00?0?00???00??0?0000?011000?00?01?0100?00?1?0000000000?00???0??0?00??00?0????0000000??00
0??01?10????000??00????????11?000??00?0????????00?????????0??????????????????02000???0??????0??
?0????????0?010??????????????????????????0000??00??1?0?000??????0?????????0???????0?????0?0???000
00?0??101?00??00?0??0?0???0????0?0?0??0?0?????0????0?0?????000???0?000?0????????0??????????001
00?0?????00?1??????0???00????00?0?0?

Sinocalliopteryx
0000????????????????0001??0???000???00?000?000??0??????????0?0?000001????????1?00010010101000
?01??10?????1?0???????00??1000020?0?????0000000??00??0100000003?0?0??000110201?000012?0????
?????00???0??000??0000?011??000?1??0000??00????010?01?000000???00?0000???00000??20000?0000
?00?????0????00?????0?????????????00000001000?[01]0?0????????00?0?20000?0?000?010100??????10?
0????0??00????????????1??010????0000000?0000000002??00?0????00?0??00?00??0100000000?????????0
0??10?00000???00??00?000?01???0?010?0?0000?000??0000000000???0???0??0?00?????0????0????00?0
0?0??0???2?????00????????????11?0????0?????????????0?????????0????????????????????2?00??00?00????
????0?????1???0000??0??000?00010??????0??000010????1?0?000??00000??0?0???0??????0??0??0?0???
0?000?00?100000??01?0?00?0?0?0010?0?0?0????0?0???0??0?0?00??0??0???00?000?0000????00???????
?000101?????10???1??????????000???00?0?0?

Mirischia
??
????????0??0?0??0????????0001?02000???01210010001011 0???
?0??????????????1??????????????????????0010???
?????????????????????????0?1?0??
??????????????0???00?0?000???
???
???0???00
0???????????????????????1??????0?0?00?000?1??0?0????????????????????????0??????0??0????0????0?????
??????1?????????????????????????????000?0????00??00??????????????????0??????????0????1???????0??

Ornitholestes_hermanni
?0002???0?0?00?1???0?010?01110001?100101?0??1000001?01011????000000100 0?0?000010?00101001
?????011?0101210000??000?0010??1????????????????01?00?????????00001??1?0?0100101000001??0?1?
??????0????????00?00????00000?0010?00?0?0000?0?0000?0100000000 1?01?000010000 02?1120000000
10?0??000000?0?00???0000?0?0000???0000000010000[01]0???????????????????????????????????0?0?001
0000?000000000000??0??0????1000??0000000?000?00000?0000?????????0000?000?001??00?0????????
00??0???0001?0?????????00??00?1?000000000000000?000000000??00??0???0000?000?00?0???00000
00?0000??01?0000?00?0000100??00001?000?0001100?00000?01000?????000000000000???0???020000
00000020000?00000?00000000???????0001000002?10?00?0??????10010?01??00000000???0100????00

0????0?00?0000?0000???????????001??1010?0000???0???0????0?00??001000000?00???000000???00000
0??000?????????0?1000?1001?0001????0??0?1010?01???????

Coelurus_fragilis
??0??0100
10000121000?????0?0002????????????0?0??0?10001????0??????????????????????????01100?000001?0?
0000110?0?0????0?00??00???0??????????0?0????0??????????????????????????11200?????????????????
??????????????????????????001000??????????????????2?????????????01000?0?0010000?000000000000
010000???0????????0000?????????0020000000000000??000???????????0000???????????0??????0???????
????0??
???0???????0
000100002?????0?000?0??100100????????????0?0001?000000000?0000?????0????????????1??00???????
???0000??000???0??00?0100????????11000000000?00?????????00000??00000001?????????0?1?0?1122
1111?????????????

Tanycolagreus
?000?????????????????00?0??????0???00110?????????0?00??????????????????????10?00??????1????????
?????0011?000?????0????0?0??0???????0000000000011?1000000??????????????????????110010000011
0000000100000000000000??00???0??000?????0?0???00?????????????????1???1????[01]2000???0??????
?????????????00??0?0???????????0?00001000??0?0?????????????20000?0000000101000?0?0010000?000
0000000000100000??01000??00000??????????002000000000000000000000?00100?00000????????0?0??
??????????????00000?????001000??????????????????0??????021000000?000?0?????????????????0??0
??00???????0?0?000??00??0??
???????????????????00001????????0011001001000000?????????00???000000000000000000?000?????
00100000000??0??0?0?100?1000000000000101000????????????0000000000?????????0?000000000001
0?????0??0?100101221011???1??0??1???

Tugulusaurus
???
???????????????0??????????????????????????????????0?0????????????????????????????000?0?100??00
110?0?1????0?????0??
???
???????????????????????2000000000000???01?????
???
???
???????????????00?????????????????000000000000?0???????0?????????0??????????????????????????0
00001???1?????????????????????????????????????0?????00?????????????????0001??1????1??0??????

Zuolong
?0000??????????????000001011 1?0???000?0000001100000?????1000????????????????00010?101??1?0
10001000????1???000000?0000??????????????????000?0??????0?????00?0100?????????????0110011000
0110?00?0???00010?0?00?????0?0100??????00??0???0011000000??0??00??1???0??0002000??0000???
??????????00??0000000??????????010?0?10??0?0???????????????????????????????????0??????10000?0000
00000000010000???????????????00?0?0?00?0020000?????????0000?000?0?1???0??????????????0???000
100????????0??00?????000000000000?0000000 1000???????1???0??00?0000?????????????????0?????0
000?00000?0?000??10??0010?0001101?00000??0?????
?????100???????00000??0???000???0??????00000?????0?00000?0?0?0??0000000000????000?000?00??0
????0?????000??00?0???0???????????000011?????0?0??0000?0?00000??00???0?0???0000000????00??0?
????1?0?10??000??01?0?01?????????

Bicentenaria
???????????00?1??????0?0??????00??00??????????????00????????????????????????0?00000001????????
????100?????????0?????????????????????0?00???????????0?1????0??10????????????????0???00000?10?
??0?110???????0??????????1????0?????????????????00??0???0???????????????1???0??????????00??????

??0??????00???????????0???0010????0???????????????2???0???0???0?????0?0?001??????00000000?0??
????????????????????0??0???0????2000?00000000?????????????0????????????????????0??????????????
??00????????0???????????0???00??0??????????????????????????????0?0000000???????????????000000
00?1??1?2?00??000??0??????0?????????
??????????????1?00??0????00????????0??0?????????0000?02???00?0???????00????????0?????00??????0
???00???0???????00???00?????0??????00??00?0??????0??0?????00?????00???????????0?????0?0??0?1?0
????????????

Kileskus
???????????????????0?01120111???00000?101?????????
??
???00?00??0??????????????????????????????1?0000?0???0??00??????1????????0??001???????????????
??
??
1011201???000?0000?0000?0???
??100010?????????????00??????????????????
??00?????00???????????????1??????0???????????
????????????????0???1?????????????????????0????????????

Guanlong
?00020?000000?010[01]10001120111200?001100000010000000000?10????00000010?00??2000000010
10?100?000000000111000000000000?0???00???????000000100000?0100000003000010000100200000
0111001000001100?0011000010000000000000000000000000?0?001000110000000110?00?11101102?
1120000?0010????0000?000?00??0000000??0?0????000000010000?0??????????????20000?0?????0101
?00?0?0010000?0000000000000010000???01000??0000000?000000000200000000000000000000?00100
?0000020?20?000?00??0001000???????0000000000000112010?1000?00?100000101000000?003??0000??0?
?00?10001000001100010??01?1000?0000000000000?1000011?000000?00000????????????020?01000
100000000000????0?000000010???????010100???????00001000021000?0?0000010001000000000000000
01000000?011000000100000?0000000?0001100000100?010010?1000100000000000010000000000?001
0010000000000000000??00000000000000010100???1?0011?1001??1000001??00?0?0?

Sinotyrannus
???????????????????0?01???101???????????????????????????????????00??0?1????????0?0000101?????????
?????0??0??00??0?0?000??????????????????????????????
??????????????????????0??0?????00?????????1?0000?0??100??0?????11????????00?001??????????????
??????????????0??
??????????????00?000?0?0???0???????????00???
???01?2?1??10???0??1?00????1???
???0??????????????????????????0??????????????????
???????????????????????????0001?????????????????????????????0?0??????0?????????????0?1??????????
?????????????????0??00???????????

Proceratosaurus_bradleyi
?00?2??????????????00011?01112000?00??????????00?00???????000?00000010?00?120000000101011??
??
???????????????????00??0????0?0??0???????????110000?0?1100?00?11?0?1?2??????00000100000?????
????00???0??00??10?0????00??
???0???????0???????????????
??00?0010112?10?1000?00?1?0000101???0????????????????0?1000100000110001???????0???0???00000
000??0?0?????????????0000??000?????0??0?0??????00100?????0001???10?00000001000?????11000?????
???0?00?????????????1????0
?0????????????????????????0??0010??1?0?1?????????????????0
00??????????

Dilong_paradoxus
?0002??000000???0?100011??1112000?00110?0000100000000100???????00000000?00??200000001010?
1?0000?0001?0??1000?????0??0?0?0??0???????0000001100?????0000000?00??1??00110201?0?0001?00
?000?0??00??00010000100?0000?00000?0?00000?000?0?0010001000000001100?001111?110201?20?00
0000????00000???0?0???000001011000 0???000??00??00????0?????????????20000?0?0?0?0101000?0?00
10000?0000000000000?0??0????1000??0000000???0?000002000000000000000000??00?0?1000?0000?0?
20?00???010000100????????000000000010002?11?1000100?0?000000000000103??0100?00??00010111
10000110001 0??00?1100??00000000010????00000?000100???????????????????020?010?010?0????010
01??10?10002?00?11?????0111000000001000001 00001??????00000110001????10000?0001??000???011
000000000000??010000000??11000????1?01?01001?????00??00???00???00000?00100?00?0000010?0?0
00000????00??0?000?0001?100???1??10112????0??0000??????????

Eotyrannus_lengi
????????????????????000120??12??0???110???????????0?????????0?000?0?0??1???20000000101012?00
?????01?0??1??????????????????????????00000010????????0?0????????????????????????????????????
????0???00000?0?00???00?0????00?0?????0?????0?1?000?00??10??0121???111?0?211?0000?0?????????
??????0??00?00100???00??0???0?0010?0??0???????????????200?0?0?000?0?0?0?0?0?0010000?0000000
?0000????????????00????????????????0??????????????????????????????0????????????????????????1??????
?????0?00?????1?00101???????00?0?01????01000?1010??1??0?00?00????????????????0???????????????
??????01?1??0???????????????????001??????0?00????????????????????000?100211010 0??????????2??00?
??00???00????0??????????00??011011?000???????????????????????????01??00?00100?000?0???0??????
1?0??0?00????1000??????0?00????????0000?00???0000100?00??0??????????0000???0?1????0??????011
??????1???0?1?10??0???

Juratyrant
???0?00?0
000001?10??00000??0???????????????????????????????????00?00?01000011010110000011?01???0??10
????0?????????????????????1????????00???0010???????????????????????2????11?????????????????????
?????????????????????????0?00100?0?0??
??????????????????00?000000000?0?00?000??00?????????????1??????????????????????????1?0?????????
??
???0????????00?10
0011?00100?0?????????????????10000001100100 0?????110000 1????????????????????0??01??1??1?????0
00?????????????0??????????????0000?0???????????000000??000??0000??????????????1??1??????11??0????
1?????????

Xiongguanlong
?0002???00000?????1?000220???0000?001101000?21000000?0001??000????????????????00000?101??20
0000000000011 1000???0?12?01000????????????????????000
0110????????????????????0??????0?00?????000?????????1?000000???00?0?1121?210201 1???0??0000?00?
000??????00???0??000????????????00?00100??
???????????????????????00?0000000??20000???????0???????????????????????????0???????000100??
???????????0010?10001????1?00?01?0?01?0000100??0010?0100??0??0??0?????0???01000?0???0?0100?
??001?111110010????100?1?11??00?100?????????????00100100001 0?1??????????????????????????????
2?000??000000001100001 1??????????????????????????111??001???????0111012????????????????00??0????
???????1??1?010?10??????????????00??????000?0??00001???????????000000?????000000???????10????1
?????????1???0?0?01?????????

Dryptosaurus
????????????????????????20???0?0?0??2???????0000101?1???????
??????????????????0???00???????????????????10???????10?0?????????????[01]?0?0?2????????101?010001
00010001000??2???0???????????0?0?????????0?0???????????????00?????????????????11????0????????????
?????????????????????????0??????????????0??????????????????????????????????0?0?0010000?00??????????

??????????????????????0????????0020000000000000??000??0??
?00?01?????????????????????????0???
???212?1?1????????????????????????
?????????????????????10????????????????????1?0???012?1?0111?????1????0??????0?????????????0??
?????????????000000???????????????00?1???00????????00??0000?00001????01?????????0?1??00?0
?0?????0???

Appalachiosaurus
???????????????????0?0?201110??????120????????????????????00000?000?0???????00001010?2????
???????????????????0?0?00???000010120?10???10??0100010
0010001000002?0?00??????0????0????????0?00100?11000000?1???1?021??????????????000??0??????0
00?????0???????????0000???0????????????0??
?????????????????????00????????0020000000000000?0000?000?00???????????????????????????????????
??00?????1????????000?000?01?010111?101??1?0020?01?10??0?0??????????????????????????????????
??????????????????????0000000101000010?00?????????????????11?110??1?????????0??1?????2?0????
????????????????????0??????????????????????????????1?01110???11111111021?0000????????????0??
???0?0???00???????????0000000???00??0?????0???????????????????00?0??00000001???0???????01????
?1??00000??1???????

Bistahieversor
?1102?????????10?2200002?01110000?001201000?2110000???????????00000000?20??2?000000101012
??0???0?
?????????????2???????10??00??00?0??0???????????120000011?00?00213?0?112??????0000000???????
??10??0????00001???0?0????00???????????????????????????????????????00?????????????????????????
???11??????????0????????????
???????0?1011001000?1010101?010111010101010020100010001???10000??0000002000021100001000?
?1111111?1?100101100121111?01000?01001111??????11?111101?10111111100?11212101100112?10
??2?00????111?????????????????0111?1????????????????????????????011?????01101111?120010000????
????????1??1?0?0????????????????????????????0??0??11001???
???1??????????000?11???????

Albertosaurus_sacrophagus
?1102??000000?1002200002201110000000120100002110000?000100000000000000020??20000000001010
120????0?10?001?100?0????000??0????0???????000000010000???0110010312001100000010120010001
10??0?00?1000??0010000020000000100000000000?000?0?0010?0120000001100?00213101112102011
00000000???00000100?00??000001001000??000000001000?0?0????????0000020000?00000?000100
1?0?0010000?00000000000000010??0?????000???00??00?00000000200000000000000000?00??0100000
??020100?000??0??001100?0110?0000001011101100100000[01]1001[01]1001011101020111002111[01]1[
01]1101?0010000000100010201002001100100011111011111111110101100111111000[01]000111[01][01
]111100001011011111111011111110011121211100?12?10?121011???????1????????????????1111102
011111?10211111102111011??1??1???1111111111220100000000??00000101?011010?100010?000??00?00
0000100?000000??0?00??110??00000000??0000?0000000001011001111010111????010000011??????

Gorgosaurus_libratus
?11020?0000001100220000220111000000001201000021100000100010000000000000002001200000000101
0120?0000010000101000000?000000000000?00001000000000100000010110010312001100000001012001
0001101001000100?00010000020000000100000000000000000?0?0010001200000011000100213101112
102011000000000000000001000000??0000010?10000??0000000010000[01]0?0????????000002000?0000
0?0001001?0?0010000?0000000000000001000010??1000??0000000?000000000200000000000000000000
?001000000000020100?000100??001100000110?00000010111011001000[01]100101001011101020112002
1101[02]1?01000100000001000102010020011001000111110111111111101011001111111000100011111111
1??001101101111111110111111100111212111100112?10?121011???????1????1101????1?0?0111110201

011111021111010211101?011?1?2?11111111?22010000000100000010110110 10?100010?00001000000
0001000000000000?0000110?000000000??0000?000000000101100111101011110??0?0000001100?000?

Alioramus
?01020?0000001100220 0?0?20111000000012010000211000001000100000000000000200?20??00001010
1?00000001000011100000 0?00?00?0??000?????????????????????????????120011000[01]001012?010??
????001000100??0?01000?02??000??10??000000?0?0000????010??110000001100 01?0213101?12102???
?000?0000000000001000 00??00000100?0000??00000?00100?0?0????????????????????????????????????
???00?0???000??2000000000000000000?0??0?1?0????0020110
?00???0??0011000???????0????00111?2????0011101111 10?0111010100120121001021110101110001 00
00102000010000001011011120111111110010110011 11111?10111001101110100011011011110110111 0
1111001112121111 10??10000???00000100001111 0?101221011111????????????????1111010??????1?1
111121??00?111?12?0100000??????0???0?1?1?010??000???????????0????01?0000?000000?0?????????
??0000??0??00???0?000?1??1101111?1?1??1011??0?000?11???????

Teratophoneus
?1?0???0???????0??2??????0111?00????1201???????0000???????????00??000??????????000010101??1??
??0????????????????????????????????0?????
?????????????????????0????0????????????????1?0000?01?????????3?????????0???000????????????10??0
???00?001???0?????0???1????????????????????????????
???100?????????????????????????????
??????????0????0?01??????????????2?021?0001??10??????????0???01?1??????0???01??1?1??21?111?10
0111101211?????????????????????????011?1?120?1????1??1100?11?????????11????????10????????????1
?????????????????1?01???1????????????????????011?1?1???????????????000??????????????????0?0????
???????????????????????0??0???1??????1??????????00??1??
??????

Daspletosaurus
?11020?000000?1002200000220101000000012010000211000001000100000000000000200120000000101
01201000001000 01?100?0????000??0????0???????0000000010000???11100103120011000000101200100
0110??0100?1000??0010000020000000100000000000000000?0?0010?00200000011000101213101112102
0110000000???00000100?00???000010010000??000000001 0000?0?0????????0000020000?00000?0001
001?0?0010000?000000000000010??0????1000??000??00?0000000002000000000000000000?00??01000
000?020110?000??0??0011000 0110?0000001011101200110201100211102111010210120121 0101[12]1
1111111111110110102000 0211001011[01]112112121111111100?111012211111110111001101111[01]10
0111110111101111111111100111222111101112?1001210110001110111102 1111???????111111?211?11
1?112111??1??111?1??1????2?11????11?12???00000??11000????1??1?010?1000???000?1000?0??????0?
000000000???0011000?0??????????????????????????11101111 0??1??????????000?11???????

Tyrannosaurus_rex
?12020?0110001100220000220101000000012010000211000001000100000000000000200120000000101
01201000001000010100000001000000000000?0????000000000010000???1110010312001100000010120010
0011010010001000100010000020000000100000000000000000?0?00100002000000210001012131012121
0201100000000000000000100000??000001001 0000??0000000010000[01]0?0????????0000020000?00000
00001001?0?0010000?000000000000010000????1000??000??00?00000000020000000000000000000?
0010000000020100?000?00??0011000011 0?000000111111200110 2101[01]0[13]01102111101021 11201
0??1012101111011[01]110111102101021100101111121 1[12]121111111001111212301111111111011
0111111111111110110201111111111111211222121101112111112112111111011111021111211111111
11021101111112111111021111111122112111111111111221100000001000000101101 1010?1000100000?
?0000000001100000000000100001100?10000000??000000000000001011101111010111101101000000 1
10010000

Tarbosaurus_baatar
??2020?011000?100220000220101000000012010000211000001000100000000000000200120000000101

0120100000100001010000?1000000000000??????000000010000?0111100103120011000000010120010
0011010010001000100010000020000000100000000000000000?0?00100002000000210001012131012121
020110000000000000000001000000??000001001000??00000000010000?0?0?????????????20000?00000?00
01001?0?0010000?0000000000000010000????1000??0000000?0000000002000000?00000000000000?001
000000020100?000?00??00110000??????0000111111120011021011130110211110102101201O??10121
111111011111011110210102110010111112112121111111000111212301111111111101101111111111111
10110201111111111111121122212?10111211111211211111111?111?1021112??1??1?111111021101111?112
1111110211111111122112111111111111221100000001000000101101101O?1000?0000001000000000011OO
00000000010000110??10000000??00000000000000101110111101011111011??00000011????0??

XVI. Phylogenetic Results. While Gondwanan dromaeosaurids are recovered as monophyletic, Laurasian dromaeosaurids are recovered in several different clades and several clades have both Asian and North American members (Fig. 3). *Dineobellator* and *Acheroraptor* are recovered within the mainly Asian Velociraptorinae. The mainly North American Dromaeosaurinae has one Asian taxon (the Baynshire Formation dromaeosaurid), with the large-bodied taxon *Dakotaraptor* as a more basal member. The Saurornitholestinae contains only two members, *Saurornitholestes langstoni* and *Atrociraptor marshalli*, with both being from North America. It is noted that *Balaur*, whose phylogenetic position has been somewhat contentious being originally considered a dromaeosaurid (Csiki et al., 2010), with others determining it to be basal among Avialae (e.g., Cau et al., 2015, 2017), was not included in the current data set. Large-bodied dromaeosaurid taxa were discussed by DePalma and coauthors (2015). While he felt they formed a monophyletic clade, current results suggest they are separated into multiple clades. *Utahraptor* and *Achillobator* are sister taxa and are part of a clade (with *Adasaurus*) sister to the velociraptorines. *Deinonychus* is then sister to both of these clades. *Dakotaraptor* is recovered as a more basal member of the Dromaeosaurinae. Contra to the findings of DePalma et al. (2015), this suggests large body size has evolved multiple times among dromaeosaurids and suggests changes in body size are easier to

accomplish evolutionarily for the group. Maastrichtian eudromaeosaurs are found in several clades. *Adasaurus* and *Atrociraptor* from the early Maastrichtian are in two distinct areas of the tree, with the former distinctly among the sister clade to the velociraptorines and the latter within the Saurornitholestinae and basal among the eudromaeosaurs. *Dineobellator* also allows us to better understand exactly what was happening in the evolution of these theropod dinosaurs in the late Maastrichtian of North America. The two late Maastrichtian Hell Creek Formation taxa (*Dakotaraptor* and *Acheroraptor*) are found within two distinct subfamilies of eudromaeosaurs, within *Acheroraptor* showing Asian affinities as part of the Velociraptorinae. *Dineobellator*, also from the late Maastrichtian, is also found within the Velociraptorinae. This suggests multiple lineages of dromaeosaurids were present during Campanian and Maastrichtian time, including those in the northern and southern portions of North America (and Laramidia). The presence of the two late Maastrichtian velociraptorines Dineobellator and Acheroraptor in North America suggests a migration event between Asia and North America during the Campanian-early Maastrichtian. With two distinct species of velociraptorines in North America during the late Maastrichtian, it suggests vicariance in North American velociraptorines during the Maastrichtian, with distinct species to the north and south. It is also noted that close morphological relationships between Asian taxa and those in the San Juan Basin during the Late Cretaceous have been found before, namely with the ankylosaurids (e.g., Sullivan, 1999), pachycephalosaurs (e.g., Sullivan, 2003, 2006b), and dromaeosaurids here suggests close connections between these two regions at or before this time, and potentially has further paleobiogeographical implications. This suggests movement of dinosaur species, with vicariance occurring

between geographical regions. Further information and fossil material of dromaeosaurids during the Late Cretaceous will allow further analysis on whether distinct lineages lived throughout this time. Preliminary analysis suggests potentially at least four lineages during the Campanian of North America, and at least two to three remaining into the Maastrichtian, specifically with vicariance occurring in different biogeographical regions.

XVII. Geologic ages used for operational taxonomic units in the phylogenetic analysis.

Acheroraptor temertyorum – Material referred to *Acheroraptor temertyorum* comes from the Upper Maastrichtian Hell Creek Formation and dates to the late Maastrichtian (Evans et al., 2013).

Achillobator giganticus – The type material of *Achillobator giganticus* was collected from the Bayanshiree Formation of Dornogovi Province, Mongolia (Perle et al., 1999) and is considered Cenomanian-Santonian in age based on the magnetostratigraphy (Hicks et al., 1999).

Adasaurus mongoliensis – Material of *Adasaurus mongoliensis* derives from the Nemegt Formation in the Gobi Desert of southern Mongolia (Barsbold, 1983). The presence of other Maastrichtian dinosaurs (e.g., *Saurolophus*) and the fact that it overlies the Djadokhta Formation (late Campanian to early Maastrichtian in age), suggests the Nemegt Formation to be early Maastrichtian in age (Funston et al., 2016).

Archaeopteryx spp. – The majority of *Archaeopteryx* specimens, and those used for scores in the phylogenetic analysis, are from the Altmühltal Formation and are of Tithonian age (Rauhut et al., 2018).

Atrociraptor marshalli – Material of Atrociraptor marshalli was collected from the top of Unit 4 of the Horseshoe Canyon Formation (Currie and Varricchio, 2004), and has given an early Maastrichtian age (Larson et al. 2010).

Austroraptor cabazai – Material of *Austroraptor cabazai* was collected from the Allen Formation in Río Negro, Argentina and is considered middle Campanian-early Maastrichtian in age (Novas et al., 2009).

Balaur bondoc – Material of *Balaur bondoc* was collected from the Sebeş Formation of Romania and is Maastrichtian in age (Cau et al., 2015).

Bambiraptor feinbergi – Material of *Bambiraptor feinbergi* was collected from the upper Two Medicine Formation near Bynum, Montana (Burnham et al., 2000) and is considered middle to late Campanian in age (Turner et al., 2012).

Bayanshiree Formation OTU – Material of this presumably new taxon was collected from the Bayanshiree Formation of Mongolia and is considered Cenomanian-Santonian in age based on the magnetostratigraphy (Hicks et al., 1999).

Boreonykus certekorum – Material referred to *Boreonykus certekorum* is known from the upper part of Unit 3 of the Wapiti Formation and is dated at 73.25 Ma in the middle late Campanian (Bell and Currie, 2016).

Buitreraptor gonzalezorum – Material of *Buitreraptor gonzalezorum* was collected from Candeleros Formation in Patagonia, Argentina, dating to near the Cenomanian-Turonian border (Makovicky et al., 2005).

Byronosaurus jaffei – Material of *Byronosaaurus jaffei* was collected from the Djadokhta Formation at Ukhaa Tolgod in southern Mongolia and is dated to the late Campanian (Norell et al., 2000).

Changyuraptor yangi – Type material of *Changyuraptor yangi* was collected from the Yixian Formation from Xijianchang, Jianchang County, Lioaning Province, China and is considered Aptian in age (Han et al., 2014; Chang et al., 2017).

Dakotaraptor steini – Material of *Dakotaraptor steini* was collected from the upper portion of the Hell Creek Formation in Harding County, South Dakota, and dates to the late Maastrichtian (Depalma et al., 2015).

Deinonychus antirrhopus – The majority of non-tooth fossils of *Deinonychus antirrhopus*, including the type material, was collected from the Cloverly Formation in Montana (Ostrom, 1969) and dated to the middle Aptian-early Albian (Ostrom, 1970).

Dineobellator notohesperis – The type material of *Dineobellator notohesperis* was collected near the base of the Naashoibito Member of the Ojo Alamo Formation in the San Juan Basin in northwestern New Mexico (Jasinski et al., 2011b). Age of this stratigraphic unit has varied, with some considering it to be from the Paleocene (e.g., Fassett and Steiner, 1997; Fassett and Lucas, 2000; Fassett et al., 2002, 2011; Fassett, 2009;), while others consider it Cretaceous (e.g., Lehman, 1981, 1984, 1985; Ford, 2000;

Sullivan et al., 2005, 2009, 2010a, 2010b, 2011a, 2011b, 2012, 2016; Lehman et al., 2006; Burns, 2008; Williamson and Weil, 2008; Jasinski et al., 2009, 2011a, 2011b, 2015, 2016; Lucas et al., 2009, 2016; Sullivan and Lucas, 2010, 2014, 2015; Fowler and Sullivan, 2011; Koenig et al., 2012; Sullivan and Jasinski, 2012; Jasinski, 2015; Jasinski and Dodson, 2015; Jasinski and Sullivan, 2016). Current understanding places the Naashoibito Member (and *Dineobellator notohesperis*) in the Maastrichtian, particularly the late Maastrichtian (probably early late to late Maastrichtian) based on radiometric dates (e.g., Heizler, 2013; Mason et al., 2013; Peppe et al., 2013; Williamson and Brusatte, 2014) and biostratigraphy (e.g., Lehman, 1981, 1984; Williamson and Weil, 2008; Jasinski et al., 2011b).

Dromaeosaurus albertensis – Non-tooth material of *Dromaeosaurus albertensis* was collected from the Dinosaur Park Formation in Dinosaur Provincial Park in Alberta, Canada (Currie, 1995; Turner et al., 2012). It was collected in the area of Little Sandhill Creek (Currie, 1995) and is considered to be middle to late Campanian in age (Turner et al., 2012; Jasinski and Dodson, 2015).

Graciliraptor lujiatensis – Material of *Graciliraptor lijuatensis* was collected from the Lujiatun Member of the Yixian Formation in Liaoning, China (Xu and Wang, 2004). Radiometric dates of this member places it in the early Aptian at around 123.2 Ma (Chang et al., 2009).

Halszkaraptor escuilliei – Type material of *Halskaraptor escuilliei* was collected form the Bayn Dzak Member of the Djadokhta Formation from Ukhaa Tolgod, Mongolia and is late Campanian in age (Cau et al., 2017).

Hesperonychus elizabethae – Material of *Hesperonychus elizabethae* was collected from the lower strata of the Dinosaur Park Formation in Dinosaur Provincial Park in Alberta, Canada and is of late Campanian age (Longrich and Currie, 2009).

Hulsanpes perlei – Type material of *Hulsanpes perlei* was collected from the Barun Goyat Formation at the Khulsan locality in the Nemegt Basin of Mongolia (Osmólska, 1982) and is considered late Campanian in age, potentially from around 77–72 Ma (Averianov and Sues, 2012).

Linheraptor exquisitus – Material of *Linheraptor exquisitus* was collected from Wulansuhai Formation of Bayan Mandahu, Inner Mongolia, China (Xu et al., 2010). It is considered late Campanian in age (Jerzykiewicz et al., 1993).

Mahakala omnogovae – Material of *Mahakala omnogovae* was collected Tugrugyin Member of the Djadokhta Formation in Ömnögov, Mongolia and is considered late Campanian in age (Turner et al., 2007b).

Microraptor spp. – Material referred to *Microraptor* has been collected from the Yixian and Jiufotang formations in Liaoning in northeastern China and are considered Aptian in age, namely middle Aptian (Xu et al., 2000).

Neuquenraptor argentinus – Material of *Neuquenraptor argentinus* was collected from the Portezuelo Formation in the Sierra del Portezuelo in Patagonia, Argentina and has been dated to Coniacian (Novas and Pol, 2005).

Rahonavis ostromi – Fossils of *Rahonavis ostromi* were collected from the Maevarano Formation in the Mahajanga Basin of Madagascar (Forster et al., 1998) and is considered to be from the Maastrichtian (Rogers et al., 2007).

Saurornitholestes langstoni – While material of *Saurornitholestes langstoni* has been collected from several formations throughout Canada and the United States, definitive non-tooth material comes from the Dinosaur Park Formation from Dinosaur Provincial Park near Steveville in Alberta, Canada (Sues, 1978; Currie, 2005). Restricting *S. langstoni* to this fossil material gives it a late Campanian age (Currie, 2005; Jasinski and Dodson, 2015; Currie and Evans, 2019).

Saurornitholestes sullivani – Holotype material of *Saurornitholestes sullivani* was collected from the De-na-zin Member of the Kirtland Formation in the San Juan Basin in northwestern New Mexico (Sullivan and Lucas, 2000; Sullivan, 2006; Jasinski, 2015). Fossil material from the De-na-zin Member, including S. sullivani, comes from the late Campanian (e.g., Lucas et al., 1987, 2000, 2010, 2011, 2016; Sullivan, 1999, 2006; Sullivan and Williamson, 1999; Sullivan and Lucas, 2000, 2003, 2006, 2014, 2015; Williamson and Carr, 2000; Lucas and Sullivan, 2003, 2006; Burns, 2008; Jasinski and Sullivan, 2010, 2011, 2016; Jasinski et al., 2011a, 2011b, 2015, 2016, 2018; Sullivan et al., 2011c, 2011d, 2011e, 2012, 2016; Sullivan and Jasinski, 2012; Jasinski, 2015; Jasinski and Sullivan, 2015; Robinson et al., 2015; Williamson and Brusatte, 2016) and the late Kirtlandian land vertebrate age (e.g., Sullivan and Lucas, 2003; 2006).

Shanag ashile – Material of *Shanag ashile* has been collected from the Öösh Formation in the Altai region of Central Mongolia (Turner et al., 2007a). This age of this strata has

not yet been well constrained and is currently considered to be from the Early Cretaceous (Berriasian–Berremian) (Turner et al., 2007a, 2012).

Sinornithosaurus millennii – Type material of *Sinornithosaurus millennii* was collected from the Jianshangou beds of the Yixian Formation and comes from right around the Barremian-Aptian border (Xu et al., 1999; Turner et al., 2012). Additional fossil specimens have been collected from the younger Dawangzhangzi bed of the Yixian Formation, which is from the early Aptian (Zhou, 2006).

Sinovenator changii – Fossil material of *Sinovenator changii* comes from the Lujiatun Beds of tbe Yixian Formation (Xu et al., 2002). Radiometric dates place these in the early Aptian (He et al., 2006).

Stenonychosaurus inequalis – Material of *Stenonychosaurus inequalis* has been collected from the lower part of the Dinosaur Park Formation (or Lower Dinosaur Park Formation in Dinosaur Provincial Park and along Red Deer River in Alberta, Canada and is lower upper Campanian in age, or around 77–75 Ma (van der Reest and Currie, 2017; Fowler, 2017).

Tianyuraptor ostromi –Type material of *Tianyuraptor ostromi* was collected from the Yixian Formation from Dawangzhangzi, Lingyuan, western Lioaning Province, China and is considered Aptian in age (Zheng et al., 2009; Chang et al., 2017).

Troodon spp. – A lot of fragmentary fossil material has been referred to *Troodon* since *Troodon formosus* was first named from an isolated tooth from the Judith River Formation of Montana (Leidy, 1856). A lot of *Troodon* material is also known from the

upper Two Medicine Formation, and considering this material, and the fact that a lot of material previously referred to *Troodon* is being re-evaluated to determine if it should belong to multiple taxa, the strata containing this genus is considered to be from the late Campanian (e.g., Rogers et al., 1993).

Tsaagan mangas – Type material of *Tsaagan mangas* was collected from the Djadokhta Formation near Xanadu in Ömnögov, Mongolia and is considered late Campanian in age (Norell et al., 2006).

Unenlagia comahuensis - Material of *Unenlagia comahuensis* was collected from the Portezuelo Formation in the Sierra del Portezuelo in Patagonia, Argentina and has been dated to Coniacian (Novas and Puerta, 1997).

Unenlagia paynemili - Material of *Unenlagia paynemili* was collected from the Portezuelo Formation near Neuquén in Patagonia, Argentina and has been dated to Coniacian (Calvo et al., 2004).

Utahraptor ostrommaysorum – Material of *Utahraptor ostrommaysorum* was collected from the upper Yellow Cat Member of the Cedar Mountain Formation of Utah and is dated to the Barremian (Kirkland et al., 1993; Jasinski and Dodson, 2015).

Velociraptor mongoliensis – Type material of *Velociraptor mongoliensis* was collected from the Djadokhta Formation in Ömnögov, Mongolia and is considered late Campanian in age (Osborn, 1924; Turner et al., 2012).

Velociraptor osmolskae - Type material of *Velociraptor osmolskae* was collected from the Bayan Mandahu Formation in Inner Mongolia, China and is considered late Campanian in age (Godefroit et al., 2008).

Zhenyuanlong suni – Type materual of *Zhenyuanlong suni* was collected from the Yixian Formation from Sihendang of Jianchang County, Lioaning Province, China and is considered Aptian in age (Lü and Brusatte, 2015; Chang et al., 2017).

XVIII. Phylopics sources

The sources for all silhouettes used in the phylogeny are provided. All silhouettes taken from phylopic.org and are freely available for reuse under a Public Domain or Creative Commons license (www.phylopic.org).

Achillobator giganticus – Matthew Martyniuk (vectorized by T. Michael Keesey), under the Creative Commons Attribution 3.0 Unported license (https://creativecommons.org/licenses/by/3.0/).

Archaeopteryx lithographica – Scott Hartman, under the Public Doman Dedication 1.0 license.

Balaur bondoc – Emily Willoughby, under the Creative Commons Attribution-ShareAlike 3.0 Unported license (https://creativecommons.org/licenses/by-sa/3.0/).

Byronosaurus jaffei (+ *Troodon*) – Danny Cicchetti (vectorized by T. Michael Keesey), under the Creative Commons Attribution-ShareAlike 3.0 Unported license (https://creativecommons.org/licenses/by-sa/3.0/).

Changyuraptor yangi – Emily Willoughby, under the Creative Commons Attribution-ShareAlike 3.0 Unported license (https://creativecommons.org/licenses/by-sa/3.0/).

Deinonychus antirrhopus – Emily Willoughby, under the Creative Commons Attribution-ShareAlike 3.0 Unported license (https://creativecommons.org/licenses/by-sa/3.0/).

Microraptor zhaoianus – Brad McFeeters (vectorized by T. Michael Keesey), under the Public Doman Dedication 1.0 license.

Rahonavis ostromi – T. Michael Keesey, under the Public Doman Dedication 1.0 license.

Saurornitholestes langstoni – Scott Hartman, under the Creative Commons Attribution 3.0 Unported license (https://creativecommons.org/licenses/by/3.0/).

Sinornithosaurus millenii – Scott Hartman (modified by T. Michael Keesey), under the Creative Commons Attribution-ShareAlike 3.0 Unported license (https://creativecommons.org/licenses/by-sa/3.0/).

Troodon formosus – Scott Hartman, Creative Commons Attribution-NonCommercial-ShareAlike 3.0 Unported license (https://creativecommons.org/licenses/by-nc/3.0/).

Utahraptor ostrommaysorum – Scott Hartman, Creative Commons Attribution-NonCommercial 3.0 Unported license (https://creativecommons.org/licenses/by-nc/3.0/).

Velociraptor mongoliensis – Scott Hartman, under the Creative Commons Attribution-ShareAlike 3.0 Unported license (https://creativecommons.org/licenses/by-sa/3.0/).

Zhenyuanlong suni – Emily Willoughby, under the Creative Commons Attribution-ShareAlike 3.0 Unported license (https://creativecommons.org/licenses/by-sa/3.0/).

Dineobellator notohesperis silhouette by Steven Jasinski and not currently on phylopics.org.

XIX. Summary. *Dineobellator* represents the most complete theropod skeleton recovered from the late Maastrichtian Naashoibito Member. It would have shared its environment with several other theropods, including caenagnathids, ornithomimids, troodontids, and tyrannosaurids (Lehman, 1981; Jasinski et al., 2011, 2016; Sullivan et al., 2011b). This dynamic ecosystem is similar to numerous other known ecosystems at the end of the Cretaceous, and presumably dromaeosaurids, or other small-sized theropod dinosaurs would be present in other North American ecosystems at this time as well. *Dineobellator* also allows us to better understand what was happening in the evolution of these theropod dinosaurs in the Maastrichtian of North America. The distinction between the two Hell Creek dromaeosaurids suggests at least two lineages of dromaeosaurids was still present in North America at the end of the Cretaceous. The presence of two velociraptorines in North America during the late Maastrichtian suggests vicariance in this clade. The close relationships between *Dineobellator* and Asian velociraptorines, along with the potential Asian affinities of some ankylosaurids and pachycephalosaurids from the San Juan Basin in the Late Cretaceous also suggests a close connection between these two biogeographical regions. Further information and fossil material of dromaeosaurids during the Late Cretaceous will allow further analysis whether distinct

lineages lived throughout this time. Preliminary analysis suggests potentially at least four lineages during the Campanian, and at least two to three present in the Maastrichtian, although whether this is due to evolutionary events among North American taxa, migration to and from Asia, or a combination of these factors, is not yet known.

XX. Supplemental Literature Cited

Arbour, V. M., L. E. Zanno, D. W. Larson, D. C. Evans, and H.-D. Sues. 2016 The furculae of the dromaeosaurid dinosaur *Dakotaraptor steini* are trionychid turtle entoplastra. *PeerJ* **4**:e1691. DOI:10.7717/peerj.1691

Averianov, A. and H.-D. Sues. 2012. Correlation of Late Cretaceous continental vertebrate assemblages in middle and central Asia. Journal of Stratigraphy 36:462–485.

Barsbold, R. 1983. Carnivorous dinosaurs from the Cretaceous of Mongolia. Transactions of the Joint Soviet-Mongolian Paleontological Expedition (in Russian) 19:5–119.

Bell, P. R., and P. J. Currie. 2016. A high-latitude dromaeosaurid, *Boreonykus certekorum*, gen. et sp. nov. (Theropoda), from the upper Campanian Wapiti Formation, west-central Alberta. Journal of Vertebrate Paleontology 36(1):e1034359. DOI:10.1080/02724634.2015.1034359

Brinkman, D. L., R. L. Cifelli, and N. J. Czaplewski. 1998. First occurrence of *Deinonychus antirrhopus* (Dinosauria: Theropoda) from the Antlers Formation

(Lower Cretaceous: Aptian–Albian) of Oklahoma. Oklahoma Geological Survey Bulletin 146:1–27.

Brusatte, S. L., G. T. Lloyd, S. C. Wang, and M. A. Norell. 2014 Gradual assembly of avian body plan culminated in rapid rates of evolution across the dinosaur-bird transition. Current Biology 24:2386–2392.

Burch, S. H. 2014. Complete forelimb myology of the basal theropod dinosaur *Tawa hallae* based on a novel robust muscle reconstruction method. Journal of Anatomy 225:271–297.

Burnham, D. A. 2004. New information on *Bambiraptor feinbergi* (Theropoda: Dromaeosauridae) from the Cretaceous of Montana. Pp. 67–111 *in* P. J. Currie, E. B. Koppelhus, M. A. Shugar and J. L. Wright, eds. Feathered Dragons. University of Indiana Press, Bloomington and Indianapolis.

Burnham, D. A., K. L. Derstler, P. J. Currie, R. T. Bakker, Z. –H. Zhou, and J. H. Ostrom. 2000. Remarkable new birdlike dinosaur (Theropoda: Maniraptora) from the Upper Cretaceous of Montana. University of Kansas Paleontological Contributions 13:1–14.

Burns, M. E., and R. M. Sullivan. 2011. A new ankylosaurid from the Upper Cretaceous Kirtland Formation, San Juan Basin, with comments on the diversity of ankylosaurids in New Mexico. New Mexico Museum of Natural History and Science, Bulletin 53:169–178.

Calvo, J. O., J. D. Porfiri, and A. W.A. Kellner. 2004. On a new maniraptoran dinosaur (Theropoda) from the Upper Cretaceous of Neuquén, Patagonia, Argentina. Arquivos do Museu Nacional, Rio de Janeiro 62(4):549–566.

Carr, T. D., and T. E. Williamson. 2010. *Bistahieversor sealeyi*, gen. et sp. nov., a new tyrannosauroid from New Mexico and the origin of deep snouts in Tyrannosauroidea. Journal of Vertebrate Paleontology 30:1–16.

Cau, A., V. Beyrand, D. Voeten, V. Fernandez, P. Tafforeau, K. Stein, R. Barsbold, K. Tsogtbaatar, P. Currie, and P. Godefroit. 2017. Synchrotron scanning reveals amphibious ecomorphology in a new clade of bird-like dinosaurs. Nature 552:395–399.

Cau, A. T. Brougham, and D. Naish. 2015. The phylogenetic affinities of the bizarre Late Cretaceous Romanian theropod *Balaur bondoc* (Dinosauria, Maniraptora): dromaeosaurid or flightless bird? PeerJ 3:e1032. DOI 10.7717/peerj.1032.

Chang, S. C., K. Q. Gao, C. F. Zhou, and F. Jourdan. 2017. New chronostratigraphic constraints on the Yixian Formation with implications for the Jehol Biota. Palaeogeography, Palaeoclimatology, Palaeoevology 487:399–406.

Chang, S. C., H. Zhang, P. R. Renne, and Y. Fang. 2009. High-precision 40Ar/39Ar age for the Jehol Biota. Palaeogeography, Palaeoclimatology, Palaeoecology 280:94–104.

Csiki, Z., M. Vremir, S. L. Brusatte, and M. A. Norell. 2010. An aberrant island-dwelling theropod dinosaur from the Late Cretaceous of Romania. Proceedings of the National Academy of Sciences of the United States of America 107:15357–15361

Currie, P. J. 1995. New information on the anatomy and relationships of *Dromaeosaurus albertensis* (Dinosauria: Theropoda). Journal of Vertebrate Paleontology 15(3):576–591.

Currie, P. J., and D. C. Evans. 2019. Cranial anatomy of new specimens of *Saurornitholestes langstoni* (Dinosauria, Theropoda, Dromaeosauridae) from the Dinosaur Park Formation (Campanian) of Alberta. The Anatomical Record. DOI:10.1001/ar.24241.

Currie, P. J., and D. D. Varricchio. 2004. A new dromaeosaurid from the Horseshoe Canyon Formation (Upper Cretaceous) of Alberta, Canada. Pp. 112–132 *in* P. J. Currie, E. B. Koppelhus, M. A. Shugar and J. L. Wright, eds. Feathered Dragons. University of Indiana Press, Bloomington and Indianapolis.

D'Emic, M. D., J. A. Wilson, and R. Thompson. 2010. The end of the sauropod dinosaur hiatus in North America. Palaeogeography, Palaeoclimatology, Palaeoecology 297:486–490.

DePalma, R. A., D. A. Burnham, L. D. Martin, P. L. Larson, and R. T. Bakker. 2015. The first giant raptor (Theropoda: Dromaeosauridae) from the Hell Creek Formation. Paleontological Contributions 14:1–16.

Elzanowski, A. 2002. Archaeopterygidae (Upper Jurassic of Germany); in L. M. Chiappe and L. M. Witmer, eds., Mesozoic Birds: Above the Heads of Dinosaurs; Berkeley, University of California Press, p. 129–159.

Evans, D. C., D. W. Larson, T. M. Cullen, and R. M. Sullivan. 2014. '*Saurornitholestes' robustus* is a troodontid (Dinosauria: Theropoda). Canadian Journal of Earth Sciences 51:730–734.

Evans, D. C., D. W. Larson, and P. J. Currie. 2013. A new dromaeosaurid (Dinosauria: Theropoda) with Asian affinities from the latest Cretaceous of North America. Naturwissenschaften 100:1041–1049.

Fassett, J. E. 2009. New geochronologic and stratigraphic evidence confirms the Paleocene age of the dinosaur-bearing Ojo Alamo Sandstone and Animas Formation in the San Juan Basin, New Mexico and Colorado. Palaeontologia Electronica 12:150 p.

Fassett, J. E., and S. G. Lucas. 2000. Evidence for Paleocene dinosaurs in the Ojo Alamo Sandstone, San Juan Basin, New Mexico. New Mexico Museum of Natural History and Science Bulletin 17:221–230.

Fassett, J. E., and M. B. Steiner. 1997. Precise age of C33N-C32R magnetic-polarity reversal, San Juan Basin, New Mexico and Colorado. New Mexico Geological Society Guidebook 48:239–247.

Fassett, J. E., L. M. Heaman, and A. Simonetti. 2011. Direct U-Pb dating of Cretaceous and Paleocene dinosaur bones, San Juan Basin, New Mexico. Geology 39:159–162. doi:10.1130/G31466.1

Fassett, J. E., R. A. Zielinski, and J. R. Budahn. 2002. Dinosaurs that did not die: Evidence for Paleocene dinosaurs in the Ojo Alamo Sandstone, San Juan Basin, New Mexico. Geological Society of America Special Paper 356:307–336.

Ford, T. L. 2000. A review of ankylosaur osteoderms from New Mexico and a preliminary review of ankylosaur armor. New Mexico Museum of Natural History and Science Bulletin 17:157–176.

Forster, C. A., S. D. Sampson, L. M. Chiappe, and D. W. Krause. 1998. The theropod ancestry of birds: new evidence from the Late Cretaceous of Madagascar. Science 279(5358):1915–1919.

Fowler, D. W., E. A. Freedman, J. B. Scannella, R. E. Kambic. 2011. The predatory ecology of *Deinonychus* and the origin of flapping in birds. PLoS ONE 6(12):e28964.

Funston, G. F., P. J. Currie, D. A. Eberth, M. J. Ryan, T. Chinzorig, D. Badamgarav, and N. R. Longrich. 2016. The first oviraptorosaur (Dinosauria: Theropoda) bonebed: evidence of gregarious behavior in a maniraptoran theropod. Scientific Reports 6:35782 (13 p.).

Godefroit, P., P. J. Currie, H. Li, C. Y. Shang, Z. Dong. 2008. A new species of *Velociraptor* (Dinosauria: Dromaeosauridae) from the Upper Cretaceous of northern China. Journal of Vertebrate Paleontology 28:432–438.

Goloboff, P. A., and S. A. Catalano. 2016. TNT version 1.5, including a full implementation of phylogenetic morphometrics. Cladistics 32:221–238.

Gong, E.-P., L. D. Martin, D. A. Burnham, A. R. Falk, and L. H. Hou. 2012. A new species of *Microraptor* from the Jehol Biota of northeastern China. Palaeoworld 21:81–91.

Han, G., L. M. Chiappe, S.-A. Ji, M. Habib, A. H. Turner, A. Chinsamy, X. Liu, and L. Han. 2014. A new raptorial dinosaur, with exceptionally long feathering provides insights into dromaeosaurid flight performance. Nature Communications 5:4382 (9 pp.).

He, H. Y., X. L. Wang, Z. H. Zhou, F. Jin, F. Wang, L. K. Yang, X. Ding, A. Boven, and R. X. Zhu. 2006. $^{40}Ar/^{39}Ar$ dating of Lujiatun Bed (Jehol Group) in Liaoning, northeastern China. Geophysical Research Letters 33:L04303 (4 p.).

Heizler, M. T., A. Mason, T. E. Williamson, D. J. Peppe, J. Ramezani, S. A. Bowring, and I. P. Mason. 2013. 40Ar/39Ar Chronostratigraphy of Cretaceous and Paleocene strata in the San Juan Basin, New Mexico: Accuracy limitations of high precision measurements. Geological Society of America, Abstracts with Programs 45:289.

Hicks, J. F., D. L. Brinkman, D. J. Nichols, M. Watabe. 1999. Paleomagnetic and palynological analyses of Albian to Santonian strata at Bayn Shireh, Burkhant, and Khuren Dukh, eastern Gobi Desert, Mongolia. Cretaceous Research 20(6):829–850.

Hunt, A. P., and S. G. Lucas. 1993. Cretaceous vertebrates of New Mexico. New Mexico Museum of Natural History and Science Bulletin 2:77–91.

Jasinski, S. E. 2011. Biomechanical modeling of *Coelophysis bauri*: possible feeding methods and behavior of a Late Triassic theropod. New Mexico Museum of Natural History and Science Bulletin 53:195–201.

Jasinski, S. E. 2015. A new dromaeosaurid (Theropoda: Dromaeosauridae) from the Late Cretaceous of New Mexico. New Mexico Museum of Natural History and Science Bulletin 67:79–87.

Jasinski, S. E., and P. Dodson. 2015. Biostratigraphy, paleobiogeography, and evolution of dromaeosaurids (Dinosauria: Dromaeosauridae) in North America. Geological Society of America Abstracts with Programs 47(7):paper no. 222-1.

Jasinski, S. E., and R. M. Sullivan. 2010. A new small pachycephalosaurid from the San Juan Basin, New Mexico and a re-evaluation of pachycephalosaurids from the Kirtlandian LVA (late Campanian). Journal of Vertebrate Paleontology 30(Supplement):111A.

Jasinski, S. E., and R. M. Sullivan. 2011. Re-evaluation of pachycephalosaurids from the Fruitland-Kirtland transition (Kirtlandian, late Campanian), San Juan Basin, New Mexico, with a description of a new species of *Stegoceras* and a reassessment of *Texacephale langstoni*. New Mexico Museum of Natural History and Science Bulletin 53:202–215.

Jasinski, S. E., and R. M. Sullivan. 2016. The validity of the Late Cretaceous pachycephalosaurid *Stegoceras novomexicanum* (Dinosauria: Pachycephalosauridae). New Mexico Museum of Natural History and Science Bulletin 74:107–115.

Jasinski, S. E., S. G. Lucas, and D. A. Moscato. 2011. Investigation into the turtles from the Late Cretaceous to Paleocene in the San Juan Basin, New Mexico. Journal of Vertebrate Paleontology 31(Supplement):131A.

Jasinski, S. E., R. M. Sullivan, and P. Dodson. 2015. Late Cretaceous dromaeosaurid theropod dinosaurs (Dinosauria: Dromaeosauridae) from southern Laramidia and implications for dinosaur faunal provinciality in North America. Journal of Vertebrate Paleontology 35(Supplement), Programs and Abstracts:150A.

Jasinski, S. E., R. M. Sullivan, A. J. Lichtig, S. G. Lucas, and P. Dodson. 2018. Baenid (Baenidae: Testudines) lower jaws from the Late Cretaceous and Paleocene of the San Juan Basin, New Mexico. New Mexico Museum of Natural History and Science Bulletin 79:311–318.

Jasinski, S. E., R. M. Sullivan, and S.G. Lucas. 2011. Taxonomic composition of the Alamo Wash local fauna from the Upper Cretaceous Ojo Alamo Formation (Naashoibito Member), San Juan Basin, New Mexico. New Mexico Museum of Natural History and Science Bulletin 53:216–271.

Jasinski, S. E., R. M. Sullivan, S. G. Lucas, and J. A. Spielmann. 2009. Taxonomic composition of the Alamo Wash local fauna from the Upper Cretaceous Ojo Alamo Formation (Naashoibito Member), San Juan Basin, New Mexico. Journal of Vertebrate Paleontology 29(Supplement):122A.

Jasinski, S. E., R. M. Sullivan, E. Snively, E. M. Morschhauser, S. G. Dalman, and P. Dodson. 2016. Theropods (Dinosauria: Theropoda) from the San Juan Basin, New Mexico, and implications for Late Cretaceous theropod faunas of Laramidia. Journal of Vertebrate Paleontology 36(Supplement), Programs and Abstracts:161A.

Jerzykiewicz, T., P. J. Currie, D. A. Eberth, P. A. Johnston, E. H. Koster, and J.-J. Zheng. 1993. Djadokhta Formation correlative strata in Chinese Inner Mongolia: an overview of the stratigraphy, sedimentary geology, and paleontology and comparisons with the type locality in the pre-Altai Gobi. Canadian Journal of Earth Sciences 30:2180–2190.

Kirkland, J. I., D. Burge, and R. Gaston. 1993. A large dromaeosaur (Theropoda) from the Lower Cretaceous of eastern Utah. Hunteria 2:1–16.

Koenig, A. E., S. G. Lucas, L. A. Neymark, A. B. Heckert, R. M. Sullivan, S. E. Jasinski, and D. W. Fowler. 2012. Direct U-Pb dating of Cretaceous and Paleocene dinosaur bones, San Juan Basin, New Mexico: COMMENT: Geology:e262.

Larson, D. W. 2008. Diversity and variation of theropod dinosaur teeth from the uppermost Santonian Milk River Formation (Upper Cretaceous), Alberta: a quantitative method supporting identification of the oldest dinosaur tooth assemblage in Canada. Canadian Journal of Earth Science 45:1455–1468.

Larson, D. W., D. B. Brinkman, and P. R. Bell. 2010. Faunal assemblages from the upper Horseshoe Canyon Formation, an early Maastrichtian cool-climate assemblage from Alberta, with special reference to the *Albertosaurus sarcophagus* bonebed. Canadian Journal of Earth Sciences 47:1159–1181.

Lehman, T. M. 1981. The Alamo Wash local fauna: A new look at the old Ojo Alamo fauna; *in* S. G. Lucas, J. K. Rigby Jr., and B. S. Kues, eds., Advances in San Juan Basin Paleontology; Albuquerque, University of New Mexico Press, p. 189–221.

Lehman, T. M. 1984. The multituberculate *Essonodon browni* from the Upper Cretaceous Naashoibito Member of the Kirtland Shale, San Juan Basin, New Mexico. Journal of Vertebrate Paleontology 4:602–603.

Lehman, T. M. 1985. Depositional environments of the Naashoibito Member of the Kirtland Shale, Upper Cretaceous, San Juan Basin, New Mexico. New Mexico Bureau of Mines and Mineral Resources, Circular 195: 55–79.

Lehman, T. M., F. W. McDowell, and J. N. Connelly. 2006. First isotopic (U-Pb) age for the Late Cretaceous *Alamosaurus* vertebrate fauna of West Texas, and its significance as a link between two faunal provinces. Journal of Vertebrate Paleontology 26:922–928.

Leidy, J. 1856. Notices of remains of extinct reptiles and fishes, discovered by Dr. F. V. Hayden in the bad lands of the Judith River, Nebraska Territory. Proceedings of the Academy of Natural Sciences of Philadelphia 8:72–73.

Liu, J., S. Ji, F. Tang, and C. Gao. 2004. A new species of dromaeosaurids [sic] from the Yixian Formation of western Liaoning. Geological Bulletin of China 23: 778–783.

Longrich, N. R. 2011. *Titanoceratops ouranos*, a giant horned dinosaur from the Late Campanian of New Mexico. Cretaceous Research 32(3):264–276.

Longrich, N. R., and P. J. Currie. 2009. A microraptorine (Dinosauria–Dromaeosauridae) from the Late Cretaceous of North America. Proceedings of the National Academy of Sciences of the United States of America (PNAS) 106:5002–5007.

Lü, J., and S. L. Brusatte. A large, short-armed, winged dromaeosaurid (Dinosauria: Theropoda) from the Early Cretaceous of China and its implications for feather evolution. Scientific Reports 5:11775. DOI:10.1038/srep11775.

Lucas, S. G. 1981. Dinosaur communities of the San Juan Basin: A case for lateral variations in the composition of Late Cretaceous dinosaur communities; *in* S. G.

Lucas, J. K. Rigby Jr., and B. S. Kues, eds., Advances in San Juan Basin Paleontology; Albuquerque, University of New Mexico Press, p. 337–393.

Lucas, S. G. 1993. Dinosaurs of New Mexico. New Mexico Academy of Science 32:130 p.

Lucas, S. G., and R. M. Sullivan. 2003. A new crocodilian from the Upper Cretaceous of the San Juan Basin, New Mexico. Neues Jahrbuch für Geologie und Paläontologie, Montashefte 2003:109–119.

Lucas, S. G., and R. M. Sullivan. 2006. *Denazinemys*, a new name for some Late Cretaceous turtles from the Upper Cretaceous of the San Juan Basin, New Mexico. New Mexico Museum of Natural History and Science Bulletin 35:223–227.

Lucas, S. G., A. B. Heckert, and R. M. Sullivan. 2000. Cretaceous dinosaurs of New Mexico. New Mexico Museum of Natural History and Science Bulletin 17:83–90.

Lucas, S. G., N. J. Mateer, A. P. Hunt, and F. M. O'Neill. 1987. Dinosaurs, the age of the Fruitland and Kirtland Formations, and the Cretaceous-Tertiary boundary in the San Juan Basin, New Mexico; *in* J. E. Fassett, and J. K. Rigby Jr., eds., The Cretaceous-Tertiary Boundary in the San Juan and Raton Basins, New Mexico and Colorado. Geological Society of America Special Paper 209, p. 35–50.

Lucas, S. G., R. M. Sullivan, S. M. Cather, S. E. Jasinski, D. W. Fowler, A. B. Heckert, J. A. Spielmann, and A. P. Hunt. 2009. No definitive evidence of Paleocene Dinosaurs in the San Juan Basin. Palaeontologia Electronica 12(2):8A(10 p.).

Lucas, S. G., R. M. Sullivan, and S. E. Jasinski. 2010. Giant hadrosaur footprints from the Upper Cretaceous Fruitland Formation, San Juan Basin, New Mexico. Journal of Vertebrate Paleontology 30(Supplement):124A.

Lucas, S. G., R. M. Sullivan, S. E. Jasinski, and T. L. Ford. 2011. Hadrosaur footprints from the Upper Cretaceous Fruitland Formation, San Juan Basin, New Mexico, and the ichnotaxonomy of large ornithopod footprints. New Mexico Museum of Natural History and Science Bulletin 53:357–362.

Lucas, S. G., R. M. Sullivan, A. J. Lichtig, S. G. Dalman, and S. E. Jasinski. 2016. Late Cretaceous dinosaur biogeography and endemism in the Western Interior Basin, North America: A critical re-evaluation. New Mexico Museum of Natural History and Science Bulletin 71:195–213.

Makovicky, P. J., S. Apesteguía, and F. L. Agnolín. 2005. The earliest dromaeosaurid theropod from South America. Nature 437:1007–1011.

Mason, I. P., M. T. Heizler, and T. E. Williamson. 2013. 40Ar/39Ar sanidine chronostratigraphy of K-Pg boundary sediments of the San Juan Basin, NM. New Mexico Geology 35:42–43.

Matthew, W. D., and B. Brown. 1922. The family Deinodontidae, with notice of a new genus from the Cretaceous of Alberta. Bulletin of the American Museum of Natural History 46:367–385.

Norell, M. A., J. M. Clark, A. H. Turner, P. J. Makovicky, R. Barsbold, and T. Rowe. 2006. A new dromaeosaurid theropod from Ukhaa Tolgod (Ömnögov, Mongolia). American Museum Novitates 3545:1–51.

Norell, M. A. and P. J. Makovicky. 2004. Dromaeosauridae. Pp. 196–209 *in* P. Dodson and H. Osmólska, eds. The Dinosauria, 2nd Edition. University of California Press, Berkeley.

Norell, M. A., P. J. Makovicky, and J. M. Clark. 2000. A new troodontid theropod from Ukhaa Tolgod, Mongolia. Journal of Vertebrate Paleontology 20:7–11.

Norell, M. A., P. J. Makovicky, and J. M. Clark. 2004. The Braincase of *Velociraptor*. Pp. 133–143 in P. J. Currie, E. B. Koppelhus, M. A. Shugar and J. L. Wright, eds. Feathered Dragons. University of Indiana Press, Bloomington and Indianapolis.

Novas, F. E., and D. Pol. 2005. New evidence on deinonychosaurian dinosaurs from the Late Cretaceous of Patagonia. Nature 433:858–861.

Novas, F. E. and P. F. Puerta. 1997. New evidence concerning avian origins from the Late Cretaceous of Patagonia. Nature 387:390–392.

Novas, F. E., D. Pol, J. I. Canale, J. D. Porfini, and J. O. Calvo. 2009. A bizarre Cretaceous theropod dinosaur from Patagonia and the evolution of Gondwanan dromaeosaurids. Proceedings of the Royal Society B 276:1101–1107.

Osborn, H. F. 1924. Three new Theropoda, *Protoceratops* zone, central Mongolia. American Museum Novitates 144:1–12.

Osmólska, H. *Hulsanpes perlei* n.g. n.sp. (Deinonychosauria, Saurischia, Dinosauria) from the Upper Cretaceous Barun Goyot Formation of Mongolia. Neues Jahrbuch für Geologie und Paläontologie, Monatshefte 7:440–448.

Ostrom, J. H. 1969. Osteology of *Deinonychus antirrhoppus*, an unusual theropod from the Lower Cretaceous of Montana. Peabody Museum of Natural History, Bulletin 30:1–165.

Ostrom, J. H. 1970. Stratigraphy and paleontology of the Cloverly Formation (Lower Cretaceous) of the Bighorn Basin area, Wyoming and Montana. Peabody Museum of Natural History, Bulletin 35:1–234.

Outamuro, D., L. Söderquist, F. Johansson, A. Ödeen, and K. Nordström. 2017. The price of looking sexy: visual ecology of a three-level predator–prey system. Functional Ecology 31(3):707–718.

Peppe, D. J., M. T. Heizler, T. E. Williamson, I. P. Mason, S. L. Brusatte, A. Weil, and R. Secord. 2013. New age constraints on Late Cretaceous through early Paleocene

age rocks in the San Juan Basin, New Mexico. Geological Society of America, Abstracts with Programs 45:290.

Perle, A., M. A. Norell, and J. Clark. 1999. A new maniraptoran theropod – *Achillobator giganticus* (Dromaeosauridae) – from the Upper Cretaceous of Burkhant, Mongolia. Contributions of the Mongolian-American Paleontological Project 101:1–105.

Rauhut, O. W. M, C. Foth, and H. Tischlinger. 2018. The oldest *Archaeopteryx* (Theropoda: Avialiae): a new specimen from the Kimmeridgian/Tithonian boundary of Schamhaupten, Bavaria. PeerJ 6:e4191. https://doi.org/10.7717/peerj.4191

Robinson, R. F., S. E. Jasinski, and R. M. Sullivan. 2015. Theropod bite marks on dinosaur bones: indications of a scavenger, predator or both?; and their taphonomic implications. New Mexico Museum of Natural History and Science Bulletin 68:275–282.

Rogers, R. R., D. W. Krause, K. Curry-Rogers, A. H. Rasoamiaramanana, and L. Rahantarisoa. 2007. Paleoenvironment and paleoecology of *Majungasaurus crenatissimus* (Theropoda: Abelisauridae) from the Late Cretaceous of Madagascar. Journal of Vertebrate Paleontology Memoir 8:21–31.

Rogers, R. R., C. C. Swisher III, and J. R. Horner. 1993. ^{40}Ar/^{39}Ar age and correlation of the nonmarine Two Medicine Formation (Upper Cretaceous), northwestern Montana, U.S.A. Canadian Journal of Earth Sciences 30:1066–1075.

Sankey, J. T. 2001. Late Campanian southern dinosaurs, Aguja Formation, Big Bend, Texas. Journal of Paleontology 75:208–215.

Sankey, J. T. 2008. Vertebrate paleoecology from microsites, Talley Mountain, upper Aguja Formation (Late Cretaceous), Big Bend National Park, Texas; *in* Sankey, J. T. and S. Baszio, eds. Vertebrate Microfossil Assemblages Their Role in Paleoecology and Paleobiogeography; Bloomington, Indiana University Press, p. 61–77.

Sankey, J. T. 2010. Faunal composition and significance of high-diversity, mixed bonebeds containing Agujaceratops mariscalensis and other dinosaurs, Aguja Formation (Upper Cretaceous), Big Bend, Texas; *in* Ryan, M.J., Chinnery-Allgeier, B.J. and Eberth, D.A., eds., New Perspectives on Horned Dinosaurs; Bloomington, Indiana University Press, p. 520–537.

Sankey, J. T., B. R. Standhardt, and J. A. Schiebout. 2005. Theropod teeth from the Upper Cretaceous (Campanian-Maastrichtian), Big Bend National Park, Texas; *in* Carpenter, K., ed., The Carnivorous Dinosaurs; Bloomington, Indiana University Press, p. 127–152.

Senter P. 2007. A method for distinguishing dromaeosaurid manual unguals from pedal “sickle claws”. Bulletin of the Gunma Museum of Natural History 11:1–6.

Senter P., J. I. Kirkland, D. D. DeBlieux, S. Madsen, and N. Toth. 2012. New dromaeosaurids (Dinosauria: Theropoda) from the Lower Cretaceous of Utah, and

the evolution of the dromaeosaurid tail. PLoS ONE 7(5): e36790 (20 pp.). doi:10.1371/journal.pone.0036790

Spielmann, J. A., S. G. Lucas, R. M. Sullivan, S. E. Jasinski, and P. L. Sealey. 2009. Giant sea turtle from the Cretaceous (upper Campanian) Pierre Shale, Raton Basin, Northeastern New Mexico. Turtle Symposium Abstracts and Program:171–175.

Sues, H.–D. 1978. A new small theropod dinosaur from the Judith River Formation (Campanian) of Alberta, Canada. Zoological Journal of the Linnean Society, London 62:381–400.

Sullivan, R. M. 1999. *Nodocephalosaurus kirtlandensis*, gen. et sp. nov., a new ankylosaurid dinosaur (Ornithischia: Ankylosauria) from the Upper Cretaceous Kirtland Formation (Upper Campanian), San Juan Basin, New Mexico. Journal of Vertebrate Paleontology 19:126–139.

Sullivan, R. M. 2003. Revision of the dinosaur *Stegoceras* Lambe (Ornithischia, Pachycephalosauridae). Journal of Vertebrate Paleontology 23:181–207.

Sullivan, R. M. 2006a. *Saurornitholestes robustus*, n. sp. (Theropoda: Dromaeosauridae) from the Upper Cretaceous Kirtland Formation (De-Na-Zin member), San Juan Basin, New Mexico. New Mexico Museum of Natural History and Science Bulletin 35:253–256.

Sullivan, R. M. 2006b. A taxonomic review of the Pachycephalosauridae (Dinosauria, Ornithischia). New Mexico Museum of Natural History and Science Bulletin 35:347–365.

Sullivan, R. M., and D. W. Fowler. 2011. *Navajodactylus boerei*, n. gen., n. sp. (Pterosauria, ?Azhdarchidae) from the Upper Cretaceous Kirtland Formation (Upper Campanian) of New Mexico. New Mexico Museum of Natural History and Science Bulletin 53:393–404.

Sullivan, R. M., and S. E. Jasinski. 2012. Coprolites from the Upper Cretaceous Fruitland, Kirtland and Ojo Alamo formations, San Juan Basin, New Mexico. New Mexico Museum of Natural History and Science Bulletin 57:255–262.

Sullivan, R. M., and S. G. Lucas. 2000. First occurrence of *Saurornitholestes* (Theropoda: Dromaeosauridae) from the Upper Cretaceous of New Mexico. New Mexico Museum of Natural History and Science Bulletin 35:105–108.

Sullivan, R. M., and S. G. Lucas. 2003. The Kirtlandian, a new land-vertebrate “age” for the Late Cretaceous of Western North America. New Mexico Geological Society, 54th Field Conference Guidebook: 369–377.

Sullivan, R. M., and S. G. Lucas. 2006. The Kirtlandian land-vertebrate “age”—faunal composition, temporal position and biostratigraphic correlation in the nonmarine Upper Cretaceous of western North America. New Mexico Museum of Natural History and Science Bulletin 35:78–79.

Sullivan, R. M., and S. G. Lucas. 2010. A new chasmosaurine (Ceratopsidae, Dinosauria) from the Upper Cretaceous Ojo Alamo Formation (Naashoibito Member), San Juan Basin, New Mexico; in M. J. Ryan, B. J. Chinnery-Allgeier, and D. A. Eberth, eds., New Perspectives on Horned Dinosaurs; Bloomington, Indiana University Press, p. 169–180.

Sullivan, R. M., and S. G. Lucas. 2014. Stratigraphic distribution of hadrosaurids in the Upper Cretaceous Fruitland, Kirtland and Ojo Alamo formations, San Juan Basin New Mexico; in D. A. Eberth, and D. C. Evans, eds., Hadrosaurs. Bloomington, Indiana University Press, p. 361–384.

Sullivan, R. M., and S. G. Lucas. 2015. Cretaceous vertebrates of New Mexico. New Mexico Museum of Natural History and Science Bulletin 68:105–129.

Sullivan, R. M., and T. E. Williamson. 1999. A new skull of *Parasaurolophus* (Dinosauria: Hadrosauridae) from the Kirtland Formation of New Mexico and a revision of the genus. New Mexico Museum of Natural History and Science Bulletin 15:1–52.

Sullivan, R. M., D. R. Braman, S. E. Jasinski, and S. G. Lucas. 2010a. New palynological data from Cretaceous strata in the San Juan Basin, New Mexico, do not indicate a Paleocene age for dinosaur fossils. New Mexico Geology, v. 32 (2):67.

Sullivan, R. M., S. E. Jasinski, M. Guenther, and S. G. Lucas. 2011a. The first lambeosaurin (Dinosauria, Hadrosauridae, Lambeosaurinae) from the Upper Cretaceous Ojo Alamo Formation (Naashoibito Member), San Juan Basin, New

Mexico. New Mexico Museum of Natural History and Science Bulletin 53:405–417.

Sullivan, R. M., S. E. Jasinski, and S. G. Lucas. 2010b. Late Cretaceous (Kirtlandian) turtles from the Fruitland and Kirtland formations, San Juan Basin, New Mexico. Journal of Vertebrate Paleontology 30(Supplement):172A.

Sullivan, R. M., S. E. Jasinski, and S. G. Lucas. 2012. Re-Assessment of Late Campanian (Kirtlandian) Turtles from the Upper Cretaceous Fruitland and Kirtland Formations, San Juan Basin, New Mexico, USA; pp. 337–387 *in* D. B. Brinkman, P. A. Holroyd, and J. D. Gardner (eds.), Morphology and Evolution of Turtles. Dordrecht: Springer.

Sullivan, R. M., S. E. Jasinski, S. G. Lucas, and J. A. Spielmann. 2009. The first "Lambeosaurin" (Dinosauria, Hadrosauridae, Lambeosaurinae) from the Upper Cretaceous Ojo Alamo Formation (Naashoibito Member), San Juan Basin, New Mexico: further implications for the age of the Alamo Wash local fauna. Journal of Vertebrate Paleontology 29(Supplement):188A.

Sullivan, R. M., S. E. Jasinski, and M. P. A. van Tomme. 2011b. A new caenagnathid *Ojoraptorsaurus boerei*, new gen., new sp. (Dinosauria, Oviraptorosauria) from the Upper Cretaceous Ojo Alamo Formation (Naashoibito Member), San Juan Basin, New Mexico. New Mexico Museum of Natural History and Science Bulletin 53:418–428.

Sullivan, R. M., S. G. Lucas, and D. R. Braman. 2005. Dinosaurs, pollen, and the Cretaceous-Tertiary boundary in the San Juan Basin, New Mexico; pp. 395–407 in S. G. Lucas (ed.), Geology of the Chama Basin: Fifty-sixth Annual Field Conference September 21–24, 2005 New Mexico Geological Society Guidebook 56.

Sullivan, R. M., S. G. Lucas, and S. E. Jasinski. 2011c. Preliminary observations on a skull of the amiid fish *Melvius*, from the Upper Kirtland Formation, San Juan Basin, New Mexico. New Mexico Museum of Natural History and Science Bulletin 53:475–483.

Sullivan, R. M., S. G. Lucas, and S. E. Jasinski. 2011d. The humerus of a hatchling lambeosaurine (Dinosauria, Hadrosauridae) referable to cf. *Parasaurolophus tubicen* from the Upper Cretaceous Kirtland Formation (De-na-zin Member), San Juan Basin, New Mexico. New Mexico Museum of Natural History and Science Bulletin 53:472–474.

Sullivan, R. M., S. G. Lucas, S. E. Jasinski, and D. H. Tanke. 2011e. An unusual sacral neural spine osteopathy of a chasmosaurine (Dinosauria: Ceratopsidae) from the Upper Cretaceous Kirtland Formation (Hunter Wash Member), San Juan Basin, New Mexico. New Mexico Museum of Natural History and Science Bulletin 53:484–488.

Sullivan, R. M., S. G. Lucas, A. J. Lichtig, S. Dalman, and S. E. Jasinski. 2016. A critique of Late Cretaceous dinosaur biogeography and endemism in the Western

Interior Basin, North America. Journal of Vertebrate Paleontology 36(Supplement), Programs and Abstracts:234A.

Turner, A. H., S. H. Hwang, and M. A. Norell. 2007a. A small derived theropod from Öösh, Early Cretaceous, Baykhangor Mongolia. American Museum Novitates 3557:1–27.

Turner, A. H., P. J. Makovicky, and M. A. Norell. 2007b. Feather quill knobs in the dinosaur *Velociraptor*. Science 317:1721.

Turner, A. H., P. J. Makovicky, and M. A. Norell. 2012. A review of dromaeosaurid systematics and Paravian phylogeny. Bulletin of the American Museum of Natural History 371:1–206.

Turner, A. H., D. Pol, J. A. Clarke, G. M. Erickson, and M. A. Norell. 2007c. A basal dromaeosaurid and size evolution preceding avian flight. Science 317:1378–1381.

van der Reest, A. J., and P. J. Currie. 2017. Troodontids (Theropoda) from the Dinosaur Park Formation, Alberta, with a description of a unique new taxon: implications for deinonychosaur diversity in North America. Canadian Journal of Earth Science 54:919–935.

Williamson, T. E., and S. L. Brusatte. 2014. Small theropod teeth from the Late Cretaceous of the San Juan Basin, northwestern New Mexico and their implications for understanding latest Cretaceous dinosaur evolution. PLoS ONE 9:e93190 (23 p.). doi:101371/journal.pone.0093190

Williamson, T. E., and S. L. Brusatte. 2016. Pachycephalosaurs (Dinosauria: Ornithischia) from the Upper Cretaceous (upper Campanian) of New Mexico: A reassessment of *Stegoceras novomexicanum*. Cretaceous Research 62:29–43.

Williamson, T. E., and T. D. Carr. 2002. A new genus of derived pachycephalosaurian from western North America. Journal of Vertebrate Paleontology 22:779–801.

Williamson T. E., and A. Weil. 2008. Metatherian mammals from the Naashoibito Member, Kirtland Formation, San Juan Basin, New Mexico and their biochronologic and paleobiogeographic significance. Journal of Vertebrate Paleontology 28:803–815.

Xu, X. and X.-L. Wang. 2004. A new dromaeosaur (Dinosauria: Theropoda) from the Early Cretaceous Yixian Formation of Western Liaoning. Vertebrata PalAsiatica 42:111-119.

Xu X., J. N. Choinere, M. Pittman, Q. W. Tan, D. Xiao, Z. Q. Li, L. Tan, J. M. Clark, M. A. Norell, D. W. E. Hone, and C. Sullivan. 2010. A new dromaeosaurid (Dinosauria: Theropoda) from the Upper Cretaceous Wulansuhai Formation of Inner Mongolia, China. Zootaxa 2403:1–9.

Xu, X., M. A. Norell, X.-L. Wang, P. J. Makovicky, and X.-C. Wu. 2002. A basal troodontid from the Early Cretaceous of China. Nature. 415:780–784.

Xu, X., Q. Tan, J. Wang, X. Zhao, and L. Tan. 2007. A gigantic bird-like dinosaur from the Late Cretaceous of China. Nature 447:844–847.

Xu, X., X.-L. Wang, and X.-C. Wu. 1999. A dromaeosaur dinosaur with filamentous integument from the Yixian Formation of China. Nature 401:262–266.

Xu, X., Z. Zhou, and X. Wang. 2000. The smallest known non-avian theropod dinosaur. Nature 408:705–707.

Xu, X., Z. Zhou, X. Wang, X. Kuang, F. Zhang, and X. Du. 2003. Four-winged dinosaurs from China. Nature 421:3335–340.

Zanno, L. E., M. A. Loewen, A. A. Farke, G. S. Kim, L. P.A.M. Claessens, and C. T. McGarrity. 2013. Late Cretaceous theropod dinosaurs of southern Utah; *in* A. Titus, and M. A. Loewen, eds., Advances in Late Cretaceous Western Interior Basin Paleontology and Geology; Bloomington, Indiana University Press, p. 504–525.

Zanno, L. E., S. D. Sampson, E. M. Roberts, and T. A. Gates. 2005. Late Campanian theropod diversity across the Western Interior Basin. Journal of Vertebrate Paleontology 25:133–134A.

Zanno, L. E., J. P. Wiersma, M. A. Loewen, S. D., Sampson, and M. A. Getty. 2010. A preliminary report on the theropod dinosaur fauna of the late Campanian Kaiparowits Formation, Grand Staircase–Escalante National Monument, Utah; *in* M. Eaton, ed., Learning from the Land, Grand Staircase–Escalante National Monument Science Symposium Proceedings. Grand Staircase–Escalante Partners, Kanab, Utah, pp. 173–186.

Zheng X., X. Xu, H. You, Q. Zhao, and Z. Dong. 2009. A short-armed dromaeosaurid from the Jehol Group of China with implications for early dromaeosaurid evolution. Proceedings of the Royal Society B 277:211–217.

Zhou, Z. 2006. Evolutionary radiation of the Jehol Biota: chronological and ecological perspectives. Geological Journal 41:377–393.
